# The Sirt2–Nur77 axis regulates muscle stem cell quiescence and senescence via epigenetic–metabolic synergy

**DOI:** 10.1038/s41419-026-08645-w

**Published:** 2026-03-28

**Authors:** Yanteng Wang, Yichen Yang, Wan Yu, Yue Liu, Wenwei Guan, Yingxi Wang, Na Li, Liu Cao, Difei Wang

**Affiliations:** 1 Department of Gerontology and Geriatrics, Shengjing Hospital of China Medical University, Shenyang, China; 2 Department of Breast Internal Medicine II, Cancer Hospital of China Medical University, Cancer Hospital of Dalian University of Technology, Liaoning Cancer Hospital & Institute, Shenyang, China; 3 The College of Basic Medical Science, Health Sciences Institute, China Medical University, Shenyang, China; 4 Key Laboratory of Cell Biology of Ministry of Public Health, Key Laboratory of Medical Cell Biology of Ministry of Education, Liaoning Province Collaborative Innovation Center of Aging Related Disease Diagnosis and Treatment and Prevention, China Medical University, Shenyang, China; 5 Clinical Translational Research Center, Shengjing Hospital, Health Sciences Institute, China Medical University, Shenyang, China; 6 Innovation Center of Aging Related Disease Diagnosis and Treatment and Prevention, Jinzhou Medical University, Jinzhou, China

**Keywords:** Ubiquitylation, Acetylation, Senescence, Muscle stem cells, Muscle stem cells

## Abstract

Nur77 expression decreases with age in multiple organs, including the liver, brain, heart, and kidney, whereas Sirt2 increases with age in the mouse cerebral cortex and hippocampus. We identified the central role of the Sirt2-P300/Nur77/K310 acetylation axis in regulating muscle homeostasis and regeneration and its age-related alterations. Consistently, we observed reduced Nur77 and elevated Sirt2 expression in aging skeletal muscle, particularly the anterior tibialis, which is enriched in type IIB and IIA fast-twitch fibers. Mechanistically, Sirt2 promoted Nur77 degradation via K310-specific deacetylation, weakening Myf5 transcriptional activity and altering satellite cell metabolic heterogeneity. Functional tests showed that Sirt2 inhibition (AGK2) or Nur77 activation (CSNB) improved muscle function in aged mice, whereas the K310R mutation led to muscle atrophy and impaired regeneration. These findings suggest the Sirt2-P300/Nur77 axis as a potential therapeutic target for skeletal muscle aging and anti-sarcopenia drug development.

## Introduction

Skeletal muscle is the largest metabolic organ in the human body. It shows a significant decline in homeostasis maintenance and regeneration capacity with age, leading to multiple degenerative diseases such as sarcopenia in older adults [1–3]. Progressive loss of muscle function is closely associated with the aging and depletion of muscle satellite cells (MuSCs). Studies have shown that quiescent satellite cells in aged individuals exhibit delayed activation and impaired regenerative capacity. Notably, even when transplanted into a young host environment, these functional deficits remain irreversible [4]. This confirms the presence of irreversible intrinsic damage in aging satellite cells. Such damage is related to compensatory adaptive mechanisms initiated by satellite cells to maintain the resting state during aging. However, in old age, this mechanism becomes dysregulated, making it difficult for satellite cells to properly exit the cell cycle and return to a resting state. This ultimately leads to the depletion of the stem cell pool and the loss of self-renewal capacity [5, 6].

Muscle aging is not an isolated event and may be regulated by systemic aging signals. Recent studies have found that the nuclear receptor Nur77 (NR4A1) exhibits an age-dependent decline in expression across multiple vital organs, including the liver, brain, adipose tissue, and kidneys [7], Concurrently, the expression levels of the deacetylase Sirt2 in the cerebral cortex and hippocampus of mice increase significantly with age [8, 9]. This pair of aging-related, cross-tissue expression changes, which operate in opposite directions, strongly suggests that the “Sirt2-Nur77 axis” may function as a conserved, systemic regulatory hub in aging. However, its specific role and mechanisms in the aging process of skeletal muscle remain unelucidated.

At the molecular mechanism level, Nur77, as a member of the NR4A nuclear receptor family, is involved in various physiological and pathological processes such as cellular metabolism and stress responses [10, 11], and its functions are precisely regulated by post-translational modifications [12, 13]. The epigenetic regulators P300 and Sirt2, serving as core acetyltransferases and deacetylases respectively, play significant roles in determining stem cell fate: P300 maintains the stemness and plasticity of various stem cells through acetylation modifications [14, 15], while Sirt2 participates in metabolic reprogramming and the regulation of aging-related pathways via deacetylation [16, 17]. However, how these two enzymes specifically regulate Nur77 through modifications to influence satellite cell function, particularly in the context of aging, remains to be elucidated.

Notably, the population of quiescent satellite cells exhibits significant metabolic and functional heterogeneity. Different subpopulations display varying activation potentials and differentiation fates during the aging process [18, 19]. Mounting evidence suggests a close interaction between cellular metabolic states and epigenetic modifications, which together form a core regulatory network governing stem cell fate decisions [20–22]. This leads us to propose the following key scientific questions: During skeletal muscle aging, does the Sirt2-Nur77 axis regulate the metabolic state and fate decisions of satellite cells through the interface of acetylation modification? What is the underlying molecular mechanism?

Therefore, this study focuses on elucidating the expression patterns of Sirt2 and Nur77 in aged skeletal muscle and satellite cells; clarifying how P300 and Sirt2 regulate Nur77 function through acetylation/deacetylation modifications at specific sites; and uncovering how this regulatory axis influences the metabolic heterogeneity, homeostasis maintenance, and regenerative capacity of satellite cells. By integrating multidisciplinary approaches, this research systematically reveals the core regulatory role of the Sirt2-P300/Nur77/K310 acetylation axis in muscle aging. This provides a new theoretical foundation for a deeper understanding of the molecular mechanisms of sarcopenia and the development of targeted intervention strategies.

## Results

### Sirt2 binds to the Nur77 DBD domain and induces nuclear Nur77 deacetylation

**Fig. 1:**
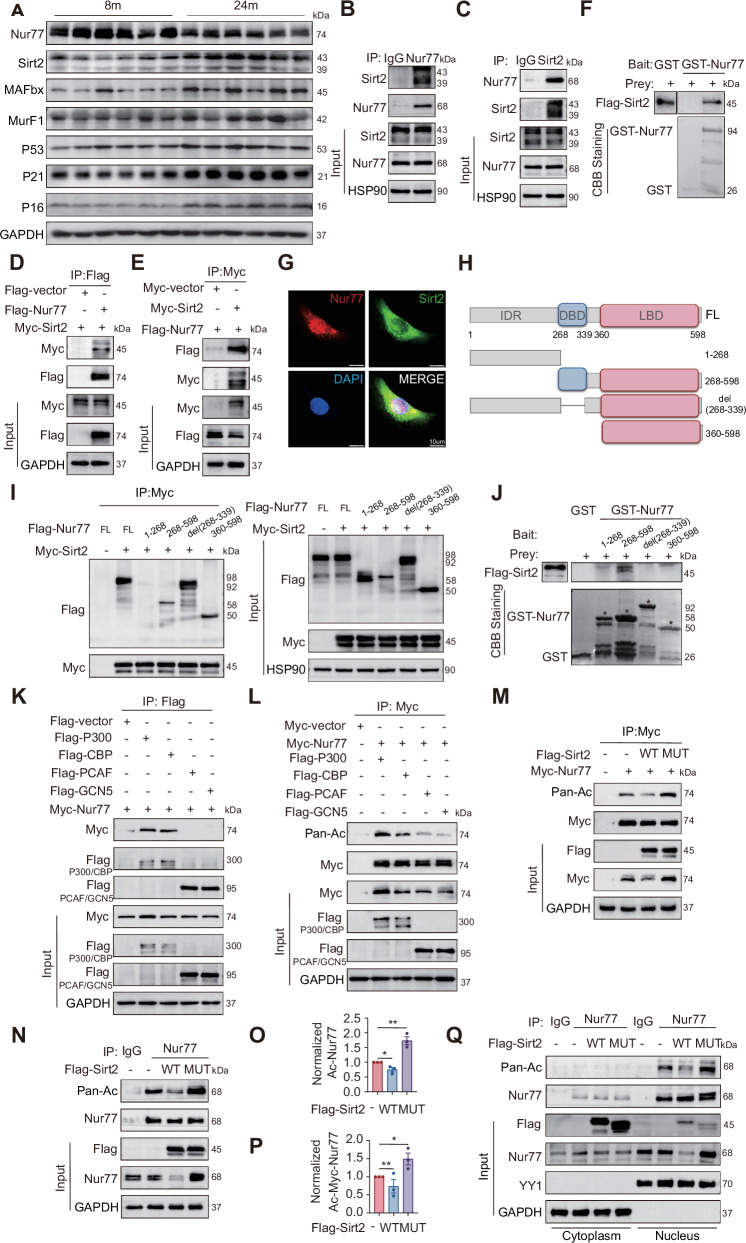
Sirt2 binds to the Nur77 DBD domain and induces nuclear Nur77 deacetylation. **A** Western blot (WB) analysis of relevant proteins in the anterior tibialis muscle of 8- and 24-month-old WT male mice (*n* = 6 mice). **B**–**E** Co-IP shows the interaction between endogenous and exogenous Nur77 and Sirt2 in HEK-293T cells. IgG, immunoglobulin. **F** GST pull-down assay was performed using bacterial-expressed GST-Nur77 and Flag-Sirt2 fusion protein. Coomassie Brilliant Blue (CBB) staining shows GST and GST-Nur77 expression. **G** Cell immunofluorescence shows Nur77–Sirt2 co-localization in C2C12 cells. Nur77, red; Sirt2, green; DAPI, blue. Scale bar: 10 μm. **H** Schematic diagram of the Nur77 598aa domain and recombinant fragment. **I** Co-IP of Flag-Nur77 fragment and Myc-Sirt2 in HEK-293T cells. **J** GST-Nur77 fragment expressed by bacteria was used for GST pull-down assay with Flag-Sirt2 fusion protein. CBB staining shows GST and GST-Nur77 fragment expression. **K** Co-IP shows interactions between Nur77 and P300/CBP/PCAF/GCN5 in HEK-293T cells. **L** Co-IP and WB analysis show Nur77 protein acetylation levels in HEK-293T cells transfected with indicated plasmids. **M**–**P** Co-IP and quantitative Flag-Sirt2-WT/MUT for endogenous and exogenous Nur77 acetylation in HEK-293T cells. *n* = 3 independent experiments. **Q** Nuclear-cytoplasmic separation and Co-IP detection of Flag-Sirt2-WT/MUT for Nur77 acetylation in HEK-293T cells. *p < 0.05, **p < 0.01. Error bars represent SEM. See also Fig. S1.

To further investigate whether Nur77 also experiences age-related decline in muscle tissue, we analyzed anterior tibial muscle tissues from 8-month-old (adult) and 24-month-old (elderly) mice, with equal numbers of males and females. The results showed that Nur77 protein expression exhibited a significant age-related decline, whereas the expression levels of the muscle atrophy-related genes MuRF1 and MAFbx significantly increased, suggesting that decreased Nur77 expression may be closely associated with age-related muscle atrophy. Additionally, we found that the protein expression of Sirt2 showed an age-related increase, suggesting that Sirt2 regulates molecular pathways involved in aging (Fig. 1A, Fig. S1A). To investigate the mechanism underlying the age-related decline of Nur77, we examined its molecular interaction with Sirt2 through various methods. Co-immunoprecipitation [12] assays demonstrated specific binding between Nur77 and Sirt2 in HEK-293T cells, primary MuSCs, and the anterior tibial muscle tissues of mice. In co-transfection experiments using Flag-Nur77 and Myc-Sirt2 constructs in HEK-293T cells, the Flag antibody successfully pulled down Myc-Sirt2 and vice versa. In vitro GST-pulldown assays further validated this binding. Additionally, immunofluorescence analysis in C2C12 cells demonstrated significant nuclear co-localization of Sirt2 and Nur77 (Fig. 1B–G, Fig. S1B–E). To clarify the key domains involved in the interaction between Nur77 and Sirt2, we generated truncated Flag-labeled constructs of Nur77 (containing its intrinsically disordered region [IDR], DNA-binding domain [DBD], and ligand-binding domain [LBD] [23]) and co-expressed them with Myc-Sirt2 in HEK-293T cells. Co-IP assays demonstrated that both the DBD and LBD truncations interacted with Myc-Sirt2, whereas the IDR truncation exhibited no significant binding. This suggests that DBD and LBD are the main interaction domains (Fig. 1H, I, Fig. S1F). Further confirmation through in vitro GST pull-down assays showed that only the purified GST-Nur77-DBD protein could specifically pull down Flag-Sirt2, clearly indicating that the DBD is the core domain for Sirt2 binding (Fig. 1J). However, overexpression or knockdown of Sirt2 does not affect the mRNA level of Nur77, suggesting that their regulatory relationship is mediated by protein–protein interactions and post-translational modifications rather than transcriptional regulation (Fig. S1G–J).

To elucidate the post-translational regulatory mechanism of Nur77, we systematically analyzed the dynamics of its acetylation. Screening four classic acetyltransferases (E1A-binding protein P300, CREB-binding protein CBP, P300/CBP-associated factor PCAF, and general regulatory factor GCN5) and transfecting them into HEK-293T cells showed that only P300 and CBP significantly enhanced Nur77 acetylation, with P300 having the strongest effect. Additionally, P300 increased Nur77 protein levels, and co-IP assays confirmed that Nur77 selectively interacts with either P300 or CBP (Fig. 1K, L, Fig. S1K). These findings identify P300 as the primary acetyltransferase targeting Nur77. Further investigation revealed that overexpression of Sirt2-WT significantly reduced both endogenous and exogenous Nur77 acetylation while concurrently decreasing Nur77 protein levels. In contrast, the enzyme-inactive mutant Sirt2-H187Y (Sirt2-MUT) lacked deacetylase activity, leading to the accumulation of acetylated Nur77 and an increase in Nur77 protein levels (Fig. 1M–P, Fig. S1L, M). These findings indicate that Sirt2 promotes the degradation of Nur77 through deacetylation. Co-IP assays confirmed that Nur77 undergoes acetylation at both its DBD and LBD (Fig. S1N, O). To elucidate the subcellular localization of Sirt2-mediated deacetylation of Nur77, we transfected HEK-293T cells with either a control vector, wild-type (Sirt2-WT), or the enzyme-inactive mutant (Sirt2-H187Y). Using nuclear-cytoplasmic separation combined with co-IP assays, we found that only Sirt2-WT significantly decreased Nur77 acetylation in the nucleus, whereas Sirt2-MUT increased nuclear acetylation of Nur77 (Fig. 1Q). To verify the pharmacological regulation of Nur77 acetylation by Sirt2, we treated HEK-293T cells stably expressing Flag-Nur77 with the Sirt2-specific inhibitor AGK2. Nuclear-cytoplasmic separation combined with Co-IP analysis revealed that AGK2 treatment significantly increased the acetylation level of exogenous Flag-Nur77 in the nuclear fraction, with no significant change observed in the cytoplasmic fraction (Fig. S1P). In summary, Sirt2 directly binds to Nur77 in a DBD-dependent manner and promotes its degradation through nuclear deacetylation, a process that can be reversed with AGK2 inhibition. In contrast, P300-mediated acetylation stabilizes Nur77. The age-related disruption of this dynamic balance (increased Sirt2 and decreased Nur77) may represent an important mechanism underlying impaired muscle regeneration during aging.

### Specific deacetylation of the K307 site of Nur77 by Sirt2

**Fig. 2:**
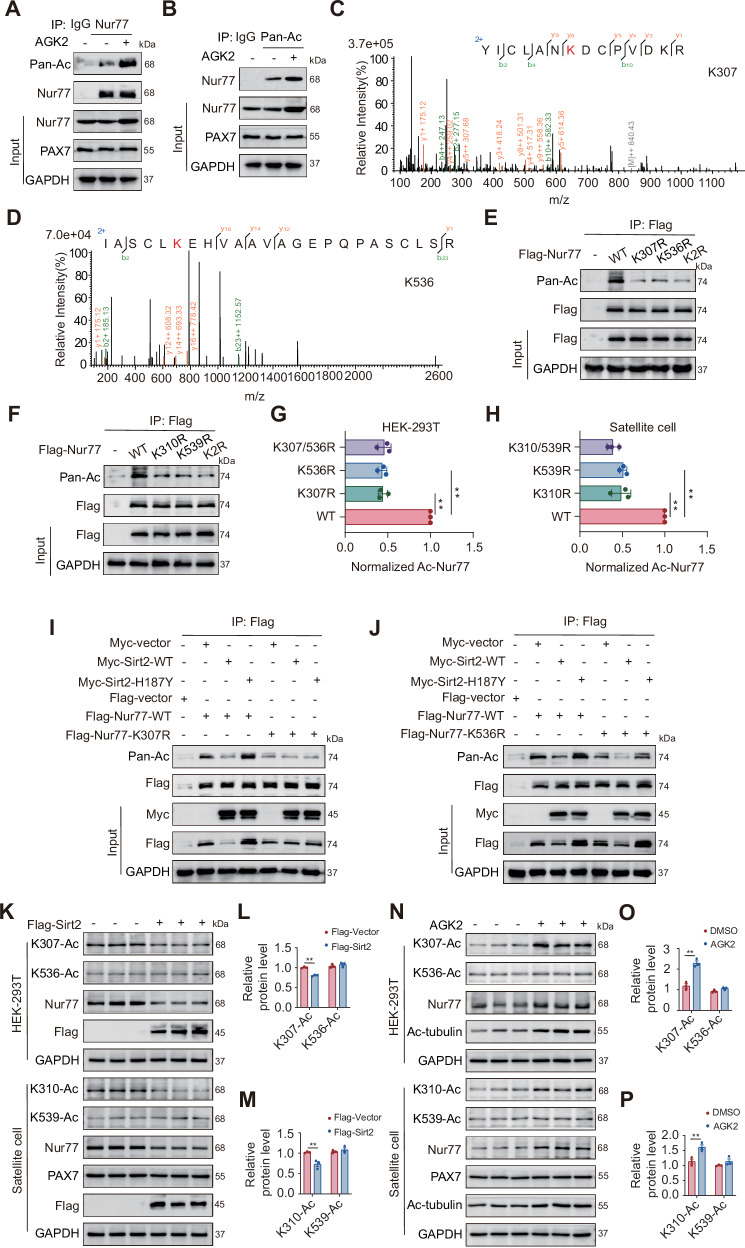
Specific deacetylation of the K307 site of Nur77 by Sirt2. **A**, **B** Co-IP shows that AGK2 affects Nur77 acetylation in primary MuSCs of the anterior tibial muscle of 8-month-old WT mice. **C**, **D** Mass spectrometry analysis detected the acetylation site peak map of the purified human Nur77 protein in vitro. **E**–**H** Co-IP and quantitative acetylation of Nur77 WT, K307(310)R, K536(539)R, and K307(310)/536(539)R in HEK-293T cells and 8-month-old WT mice primary MuSCs. *n* = 3 independent experiments. **I**, **J** Co-IP shows that Myc-Sirt2-WT/H187Y acetylates Flag-Nur77-WT/K307R/K536R in HEK-293T cells. *n* = 3 independent experiments. **K**–**P** WB analysis with quantification shows that Sirt2 overexpression and AGK2 treatment specifically regulate the acetylation levels of Nur77 at the K307/K310 and K536/K539 sites in HEK-293T cells and 8-month-old WT mouse primary MuSCs. *n* = 3 independent experiments. *p < 0.05, **p < 0.01. Error bars represent SEM.

Based on our previous findings in HEK-293T cells demonstrating that Sirt2 mediates the deacetylation of Nur77, we further validated this regulatory relationship in primary mouse MuSCs. Treatment with the Sirt2-specific inhibitor AGK2 and Co-IP assays showed that AGK2 significantly increased the acetylation level of Nur77 (Fig. 2A, B), which was consistent with the findings in HEK-293T cells, confirming that Sirt2 also regulates Nur77 through deacetylation in MuSCs. Furthermore, mass spectrometry analysis of the purified human Nur77 protein (598 amino acids) revealed two endogenous acetylation modification sites: K307 and K536 (Fig. 2C, D). Multiple sequence alignment showed that these two acetylation sites are highly conserved across various mammals (Table S1). Notably, although the mouse Nur77 protein is slightly longer than the human version (601 vs. 598 amino acids), the corresponding acetylation sites (K310 and K539 in mice) are identical in spatial position and surrounding sequences, suggesting their conservation and potential regulatory importance in Nur77 function across species. By constructing human (K307R, K536R, and K307/536 R double mutations) and mouse (K310R, K539R, and K310/539 R double mutations) Nur77 acetylation site mutants and transfecting them into HEK-293T cells and primary mouse MuSCs, Co-IP assays showed that, compared to WT Nur77, the acetylation levels of mutants (K307R or K536R) were significantly decreased. Mouse homologous site mutations (K310R or K539R) exhibited similar acetylation patterns to the human mutants (Fig. 2E–H), further confirming the functional conservation of these acetylation sites across species. To clarify the specificity of Sirt2 regulation at different acetylation sites in Nur77, we conducted two parallel experiments in HEK-293T cells. The first group was co-transfected with Flag-Nur77-WT or K307R and Myc-Sirt2-WT or MUT (H187Y), whereas the second group was co-transfected with Flag-Nur77-WT or K536R and Myc-Sirt2-WT or MUT (H187Y). Co-IP results showed that Myc-Sirt2-WT significantly reduced the acetylation levels of the K536R mutant, whereas the enzymatically inactive H187Y mutant increased acetylation levels. In contrast, neither Sirt2-WT nor MUT caused significant changes in the acetylation levels of the K307R mutant (Fig. 2I, J). These results indicate that Sirt2 mainly mediates the deacetylation of Nur77 at the K307 site rather than at K536. Previous GST pull-down assays have confirmed that Sirt2 binds to the DBD of Nur77, with K307 located precisely within this core domain. Subsequently, through site-specific acetylation antibody detection, we confirmed that Sirt2 overexpression significantly reduced acetylation at the K307 (human) and K310 (mouse) sites in HEK-293T cells and mouse primary MuSCs, without affecting the K536 or K539 sites. Conversely, treatment with the Sirt2 inhibitor AGK2 significantly increased acetylation levels at the K307 and K310 sites, whereas those at the K536 and K539 sites remained unchanged (Fig. 2K–P). These results directly demonstrate that Sirt2 regulates Nur77 function through specific deacetylation at the K307 and K310 sites, rather than at K536 or K539, providing a precise molecular target for understanding age-related muscle regeneration disorders.

### Sirt2 regulates Nur77 protein stability through a K307-site acetylation-ubiquitination cascade

**Fig. 3:**
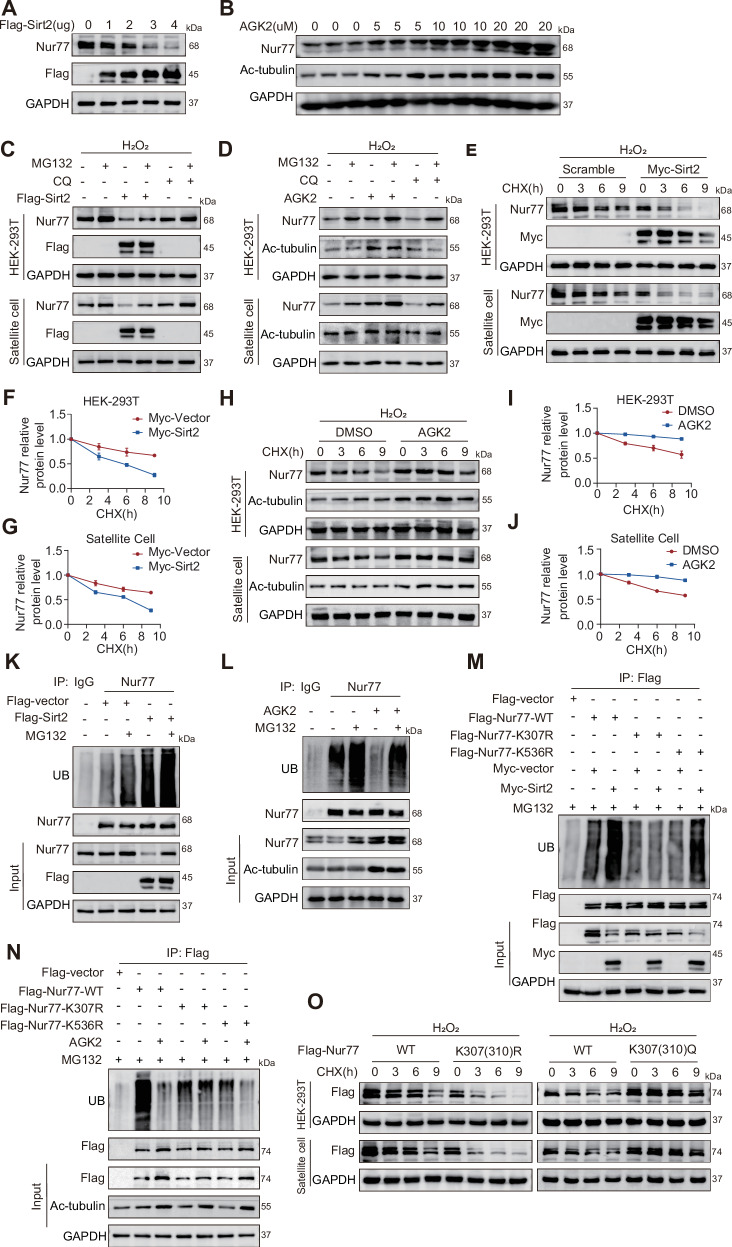
Sirt2 regulates Nur77 protein stability through a K307-site acetylation–ubiquitination cascade. **A**, **B** WB analysis shows Nur77 protein expression in HEK-293T cells overexpressing a gradient of Sirt2 and a concentration gradient of AGK2 (24 h). *n* = 3 independent experiments. **C**, **D** WB analysis shows Nur77 protein in HEK-293T cells and 8-month-old WT mice primary MuSCs under conditions of overexpressing Sirt2 (or 10 μM AGK2 for 24 h) and 50 μM Z-leucine aldehyde (MG 132) for 4 h or 20 μM chloroquine (CQ) for 10 h. *n* = 3 independent experiments. **E**–**J** WB analysis detecting and quantifying Nur77 protein in HEK-293T cells and 8-month-old WT mice primary MuSCs under conditions of Myc-Sirt2 (or 10 μM AGK2 for 24 h) and 100 μg/ml nystatin CHX. *n* = 3 independent experiments. **K**, **L** Co-IP shows endogenous Nur77 ubiquitination in HEK-293T cells under Myc-Sirt2 (or 10 μM AGK2 for 24 h). *n* = 3 independent experiments. **M**, **N** Co-IP shows Flag-Nur77-WT/K307R/K536R ubiquitination in HEK-293T cells under Myc-Sirt2 (or 10 μM AGK2 for 24 h). *n* = 3 independent experiments. **O** WB analysis shows Flag-Nur77-WT/K307(310)Q in HEK-293T cells and 8-month-old WT mice primary MuSCs under 100 μg/ml nystatin CHX. *n* = 3 independent experiments; *p < 0.05, **p < 0.01. Error bars represent SEM. See also Fig. S2.

To investigate the regulatory effect of Sirt2 on Nur77 protein levels, we conducted dose-response experiments in HEK-293T cells. Increasing amounts of the transfected Sirt2 plasmid resulted in a gradual decrease in Nur77 protein levels, confirming that Sirt2 promotes Nur77 degradation in a dose-dependent manner. Conversely, treatment with increasing concentrations of the Sirt2 inhibitor AGK2 led to a significant concentration-dependent increase in Nur77 protein levels, and this effect persisted in the H₂O₂-induced aging model in both HEK-293T cells and primary MuSCs (Fig. 3A, B, Fig. S2A). These results indicate that AGK2 can reverse the loss of Nur77 in aging cells by inhibiting Sirt2. To elucidate the specific mechanism by which Sirt2 mediates Nur77 protein degradation, we combined the proteasome inhibitor MG132 (50 μM, 4 h) and the autophagy inhibitor chloroquine (CQ, 20 μM, 10 h) in HEK-293T cells and MuSCs overexpressing Sirt2 and treated with AGK2. The results showed that in the H₂O₂-induced aging model, MG132 alone significantly restored Nur77 protein levels, whereas CQ alone had no significant effect. However, when MG132 and CQ were combined, the recovery effect of Nur77 was not further enhanced compared to MG132 alone (Fig. 3C, D). Additionally, the cycloheximide (CHX, 100 μg/ml) tracking experiment indicated that Sirt2 overexpression significantly shortened the half-life of Nur77 protein, whereas AGK2 treatment prolonged its half-life (Fig. 3E–J, Fig. S2B–G). Hence, Sirt2 promotes Nur77 degradation by activating the proteasome pathway, and AGK2 helps maintain the stability of Nur77 by inhibiting its proteasomal degradation. To clarify the molecular mechanism by which Sirt2 regulates the degradation of Nur77, we measured the ubiquitination level of Nur77 using co-IP in HEK-293T cells. Overexpression of Sirt2 significantly increased the polyubiquitination of both endogenous and exogenous Nur77 (Fig. 3K, L, Fig. S2H, I), confirming that Sirt2 promotes the ubiquitination of Nur77 for proteasomal degradation. Treatment with the Sirt2 inhibitor AGK2 significantly reduced the ubiquitination level of Nur77, consistent with its effect of enhancing Nur77 protein expression. This effect was dependent on the deacetylase activity of Sirt2, suggesting that the acetylation state of Nur77 directly influences its susceptibility to ubiquitination. Based on our previous finding that Sirt2 promotes Nur77 degradation through K307 deacetylation, we further investigated whether K307 directly participates in ubiquitination. Comparison of the ubiquitination levels of Flag-Nur77-WT, K307R, and K536R mutants in HEK-293T cells revealed that the overall ubiquitination levels of the K307R and K536R mutants were significantly lower than that of WT (Fig. S2J), suggesting that K307 and K536 may be direct sites of ubiquitination or affect other sites through conformational changes. To clarify the site-specific regulation of Sirt2 on Nur77 ubiquitination, we transfected Flag-Nur77-WT, K307R, and K536R plasmids into HEK-293T cells in combination with Sirt2 overexpression or AGK2 treatment. Co-IP assays showed that Sirt2 overexpression significantly increased the ubiquitination levels of WT and K536R Nur77 but had no effect on the K307R mutant. Conversely, AGK2 treatment reduced the ubiquitination levels of WT and K536R, with no change in the ubiquitination state of K307R (Fig. 3M, N). Hence, K307 is the target of Sirt2-mediated deacetylation and is a key site for regulating Nur77 ubiquitination and degradation. In contrast, K536 may indirectly affect this process through conformational changes, revealing a precisely coordinated regulatory mechanism of acetylation-ubiquitination at the K307 site. To clarify the direct effect of K307 (human) or K310 (mouse) site acetylation on the stability of Nur77, we generated Flag-Nur77-WT, K307 or 310 R (simulating deacetylation), and K307 or 310Q (simulating acetylation) lentiviruses in HEK-293T cells and primary MuSCs and assessed the protein half-life through ChX tracking experiments. The K307 or 310 R mutation significantly shortened the half-life of the Nur77 protein, whereas the K307 or 310Q mutation prolonged its half-life (Fig. 3O, Fig. S2K–N). These results directly confirm that the acetylation state of the K307 or K310 sites is a key molecular switch regulating Nur77 protein stability. Deacetylation (K307 or 310 R) promotes degradation, whereas acetylation (K307 or 310Q) enhances stability, providing evidence for Sirt2-mediated Nur77 degradation. Collectively, Sirt2 promotes Nur77 ubiquitination and degradation in a dose-dependent manner by triggering ubiquitination through specific deacetylation at the K307 site, ultimately shortening its protein half-life; inhibition of Sirt2 or simulation of K307 acetylation (K307Q) reverses this process, elucidating the core mechanism underlying the progressive decline of Nur77 in aging muscle.

### Knockout of Nur77 leads to sarcopenia phenotype

**Fig. 4:**
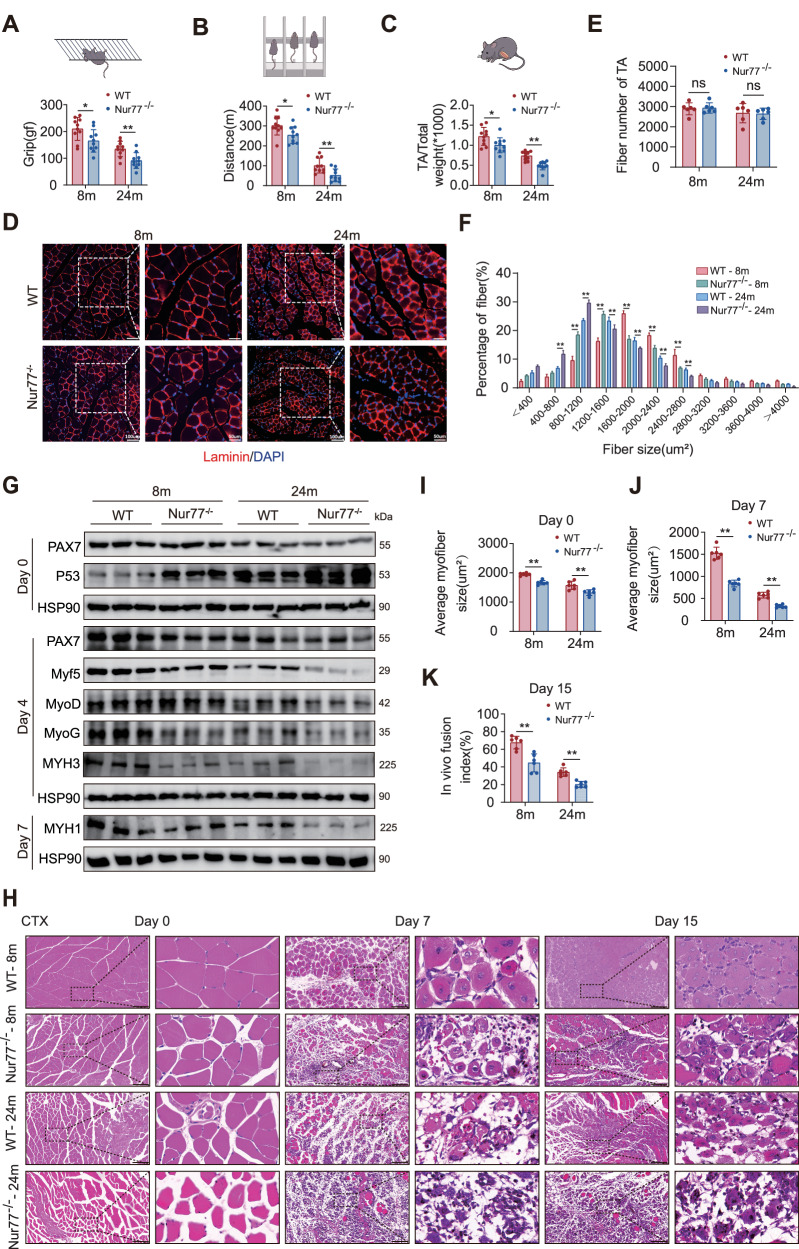
Nur77 knockout leads to sarcopenia phenotype. **A**–**C** Forelimb grip strength, running wheel distance, and comparison of anterior tibialis muscle or body weight in 8- and 24-month-old WT and Nur77^−/−^ mice (*n* = 10 mice); **D** Representative immunofluorescence staining images of anterior tibialis muscle cross-sections of 8- and 24-month-old WT and Nur77^−/−^ mice (laminin, red—basement membrane; DAPI, blue—cell nucleus). Scale: 100 μm (*n* = 6 mice). **E**, **F** Statistical analysis of the total muscle fiber number and percentage distribution of muscle fibers in different cross-sectional areas (μm²) in the anterior tibialis cross-sections of 8- and 24-month-old WT and Nur77^−/−^ mice (*n* = 6 mice). **G** WB detection of related protein expression in the anterior tibialis muscle of 8- and 24-month-old WT and Nur77^−/−^ mice 0 d, 4, and 7 d after CTX injection (50 μl 10 μM multi-site injection, *n* = 6 mice). **H** Representative HE staining of muscle cross-sections of the anterior tibialis muscles of 8- and 24-month-old WT and Nur77^−/−^ mice examined on days 0, 7, and 15 after CTX injection. Scale: 100 μm (50 μl 10 μM multi-site injection, *n* = 6 mice). **I**–**K** Quantitative analysis of the average muscle fiber cross-sectional area after CTX injection in the anterior tibialis muscle of 8- and 24-month-old WT and Nur77^−/−^ mice (0 d); comparison of the average muscle fiber cross-sectional area (7 d); muscle fiber fusion index (15 d). Fusion index (%) = Number of muscle fibers containing ≥ 3 central nuclei / Total number of central nucleus-positive muscle fibers × 100 (*n* = 6 mice); *p < 0.05, **p < 0.01. Error bars represent SEM. See also Fig. S3.

To explore the role of Nur77 in maintaining muscle function, we constructed a global Nur77 knockout mouse model (Nur77^−/−^) and conducted phenotypic analyses of 8- and 24-month-old WT and Nur77^−/−^ mice. The grip strength, running distance, and wet weight of the anterior tibial muscle in Nur77^−/−^ mice were significantly lower than those in WT mice of the same age. Aging further exacerbated these reductions (Fig. 4A–C, Fig. S3A), suggesting that Nur77 suppresses the decline in muscle function associated with aging. Hematoxylin-eosin (H&E) and Laminin immunofluorescence staining analyses of the anterior tibial muscles of 8- and 24-month-old WT and Nur77^−/−^ mice revealed no significant differences in the number of muscle fibers between age-matched WT and Nur77^−/−^ mice. However, the cross-sectional area of muscle fibers was significantly reduced in Nur77^−/−^ mice compared to WT mice of the same age (Fig. 4D–F, Fig. S3B). In 6-month-old mice with muscle-specific knockout of Nur77 (CKO), generated by crossing Myf5-cre mice with Nur77 ^flox/flox^ mice, H&E staining revealed that, compared to Nur77 ^flox/flox^ control mice, the CKO mice showed no significant change in the number of tibialis anterior muscle fibers but exhibited a significant reduction in fiber cross-sectional area (Fig. S3C–F). Therefore, the specific absence of Nur77 led to muscle fiber atrophy rather than a reduction in the number of muscle fibers.

To investigate the role of Nur77 in muscle injury repair, we performed cardiotoxin (CTX) injury experiments (50 μl of 10 μM CTX, multiple-site injection) on the anterior tibial muscles of 8- and 24-month-old WT and Nur77^−/−^ mice, based on methods described in previous studies [24–27]. Before injury, PAX7 expression was not significantly different between WT and Nur77^−/−^ mice within the same age group, although expression varied between age groups. Four days post-injury, the expression levels of PAX7, Myf5, MyoG, and MYH3 in the 8- and 24-month-old Nur77^−/−^ mice were significantly lower than those in the WT mice of the same age, whereas MyoD expression was reduced only in the 24-month-old Nur77^−/−^ mice. By day seven post-injury, the expression of mature myosin MYH1 was significantly downregulated in both age groups of Nur77^−/−^ mice (Fig. 4G). Hence, the absence of Nur77 impairs the expression of myogenic regulatory factors during muscle regeneration, with the impairment becoming more pronounced with age. H&E staining analysis at day seven post-injury revealed that an increased number of typical regenerative muscle fibers with a central nucleus was observed in the 8-month-old WT mice, indicative of active regeneration. In contrast, age-matched Nur77^−/−^ mice showed disordered muscle fiber arrangement, structural tissue destruction, and a significant reduction in the average cross-sectional area of muscle fibers. The 24-month-old WT mice already exhibited delayed regeneration characteristics; however, the degree of muscle fiber disorder in the Nur77^−/−^ mice was more pronounced. A total of 15 days post-injury, the muscle fiber fusion index of Nur77^−/−^ mice was significantly reduced (Fig. 4H–K), indicating that the absence of Nur77 delays muscle regeneration and also disrupts the proper maturation of regenerating muscle fibers. These regenerative defects increased with age. Hence, the absence of Nur77 leads to age-dependent impairment of muscle regeneration through dysregulation of myogenic regulatory factor expression.

### Nur77 regulates Myf5-mediated MuSC activation and improves aging-related muscle regeneration dysfunction

**Fig. 5:**
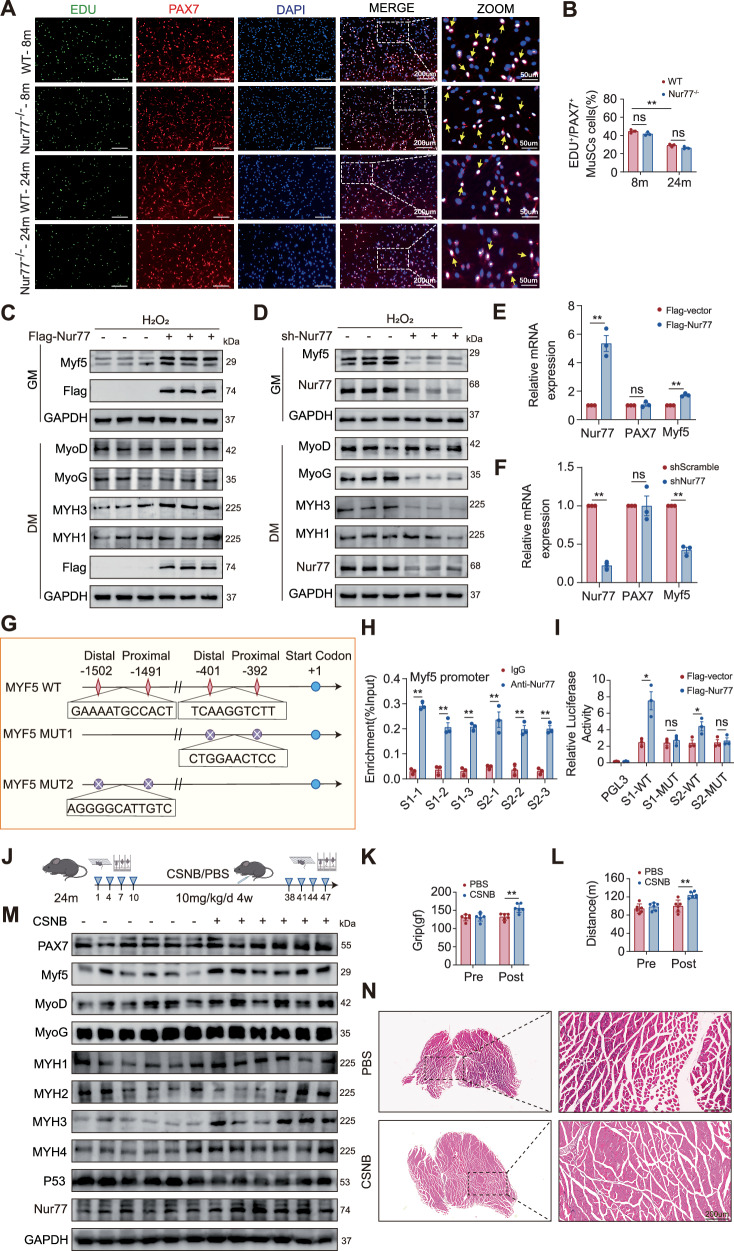
Nur77 regulates Myf5-mediated MuSC activation and improves aging-related muscle regeneration dysfunction. **A** Representative immunofluorescence staining images of primary MuSCs isolated from anterior tibialis muscles of 8- and 24-month-old WT and Nur77^−/−^ mice. EdU, green—proliferation; PAX7, red—MuSCs; DAPI, blue—cell nucleus. The yellow arrow indicates cells positive for both EdU and PAX7. Scale: 100 μm. *n* = 3 independent experiments. **B** Bar graph of the percentage of EdU^+^/PAX7^+^ MuSCs. **C**, **D** Primary MuSCs isolated from anterior tibialis muscles of 8-month-old WT mice were respectively treated with Nur77 overexpression (Flag-Nur77) and knockdown (shNur77) lentiviruses. WB was used to detect related protein expression after 1 h of H₂O₂-induced senescence stress. GM: proliferation medium, DM: differentiation medium. **E**, **F** Primary MuSCs isolated from tibialis anterior muscles of 8-month-old WT mice were treated with either Nur77 overexpression (Flag-Nur77) or knockdown (shNur77) lentiviruses, and RT-qPCR was used to detect related genes mRNA levels after 1 h of H₂O₂-induced senescence stress. *n* = 3 independent experiments. **G**, **H** ChIP was performed using IgG antibody as a control on HEK-293T cells to analyze Nur77 enrichment in the *Myf5* promoter region. *n* = 3 independent experiments. **I** Results of dual luciferase reporter gene assay of HEK-E293T cells expressing Nur77 and luciferase reporter gene containing WT or mutant *Myf5*. *n* = 3 independent experiments. **J**–**L** After 24-month-old WT mice were intraperitoneally injected with CSNB (10 mg/kg/d for 4 weeks), forelimb grip strength and running wheel distance were measured (*n* = 6 mice). **M** WB detection of related protein expression in 24-month-old WT mice after intraperitoneal injection of CSNB (*n* = 6 mice). **N** Representative HE-stained histological sections showing cross-sectional morphology of the tibialis anterior muscle from 24-month-old WT mice following PBS or CSNB administration. Scale: 200 μm (*n* = 6 mice). *p < 0.05, **p < 0.01. Error bars represent SEM. See also Fig. S4.

To verify the expression pattern of Nur77 during cellular aging, we established an H₂O₂-induced oxidative stress model by treating HEK-293T cells and primary MuSCs isolated from the anterior tibial muscle of mice with 0, 250, 500, and 750 μM H₂O₂ for 1 h, according to previously described methods [28]. As the H₂O₂ concentration increased, Nur77 expression levels decreased significantly in a dose-dependent manner, whereas the expression of the classical aging markers p53 and p21 increased correspondingly (Fig. S4A). This finding was consistent with the age-related decrease in Nur77 expression observed in the anterior tibial muscle of mice, further confirming that Nur77 expression is negatively correlated with aging. To explore the role of Nur77 in the proliferation of MuSCs, we isolated and cultured MuSCs from the anterior tibial muscle of 8- and 24-month-old WT and Nur77^−/−^ mice. The EdU staining proliferation experiment showed that, compared with WT mice, the satellite cells of the Nur77 gene-deficient mice of the same age exhibited a downward trend in proliferation, but no statistical difference was observed. However, the proliferation ability of satellite cells from 24-month-old mice was significantly lower than that of 8-month-old mice (Fig. 5A, B). The expression of the proliferation marker Ki67, as detected by WB, was consistent with the results of EdU staining (Fig. S4B). Therefore, although Nur77 significantly regulates muscle regeneration, it may not directly regulate satellite cell proliferation. However, Nur77 may affect regeneration through other pathways, such as modulating the cellular niche or by influencing satellite cell division, thereby affecting the maintenance or differentiation of the stem cell pool. To investigate the role of Nur77 in MuSC differentiation, we isolated and cultured primary satellite cells from the anterior tibial muscles of mice and simulated aging stress with H₂O₂. In the undifferentiated state (maintained with growth medium), Myf5 expression was significantly increased and decreased in the Flag-Nur77 and shNur77 cells, respectively, suggesting that Nur77 specifically promotes the expression of the myogenic activator Myf5. These findings indicate that Nur77 acts as a key regulatory switch in the transition of myogenic satellite cells from the quiescent state (PAX7^+^) to the activated state (Myf5^+^). Following induction with 5% horse serum and insulin (10 μg/ml) for 3 days to promote differentiation using differentiation medium, MyoD and MYH1 expression remained unchanged in both experimental groups, indicating that they are not directly affected by Nur77 during the activation or late maturation stages of myogenic differentiation. In contrast, MyoG expression decreased in the shNur77 group but remained unchanged in the Flag-Nur77 group, suggesting that Nur77 may maintain basal MyoG levels through an indirect pathway. Notably, MYH3 (embryonic myosin) expression increased in Flag-Nur77 cells and significantly decreased in shNur77 cells, clearly demonstrating that Nur77 promotes early myotube formation (Fig. 5C, D, Fig. S4C). Nuclear–cytoplasmic separation revealed that, in undifferentiated myogenic satellite cells, Nur77 was mainly localized in the nucleus, consistent with its function as a nuclear transcription factor, and was significantly transferred to the cytoplasm after 3 days of differentiation (Fig. S4D). This suggests a potential reduction in its transcriptional regulatory function during differentiation. Further overexpression and knockdown experiments showed that Nur77 did not significantly affect PAX7 mRNA levels but could significantly upregulate the transcription levels of Myf5 (Fig. 5E, F). Based on the observations we made previously regarding CTX-induced damage, the decreased level of PAX7 protein and the reduced proliferation trend of satellite cells in Nur77 knockout mice (Fig. 4G), we can infer that Nur77 may affect the expression of PAX7 at a non-transcriptional level. Based on JASPAR database predictions (Table S2, we identified two high-scoring binding sites (S1 and S2) for Nur77 in the Myf5 promoter region. ChIP-qPCR in HEK-293T cells confirmed the specific binding of Nur77 to these sites. A dual-luciferase reporter assay further confirmed that Nur77 overexpression significantly enhanced the activity of the WT Myf5 promoter, whereas the mutant [14] promoter showed no response, clearly demonstrating that Nur77 directly binds to the Myf5 promoter to positively regulate its transcription and promote the activation of myogenic satellite cells (Fig. 5G–I). To confirm that the pharmacological action of the Nur77 agonist CSNB strictly depends on the Nur77 protein, we performed a validation experiment using primary tibialis anterior MuSCs stimulated with H₂O₂ to mimic an aged state. Following specific knockdown of Nur77, the enhancing effect of CSNB on the expression of its downstream target Myf5 was significantly attenuated, confirming that the effect of CSNB is dependent on the Nur77 protein (Fig. S4E). Having clarified the mechanism of action of CSNB, we further evaluated its therapeutic potential at the whole-animal level. We conducted a 4-week intervention experiment in 24-month-old WT mice, following previously described methods [23, 29, 30]. Mice were administered cytosporone B (CSNB: 10 mg/kg/day, intraperitoneally) or PBS as a control. Behavioral and molecular analyses revealed that running distance and grip strength in the CSNB-treated group significantly improved after the intervention, whereas no changes were observed in the PBS control group, indicating that Nur77 activation can reverse age-related declines in muscle strength (Fig. 5J–L). WB analysis showed increased expression of the early activation factor Myf5 and embryonic myosin MYH3, consistent with the direct transcriptional regulation of Myf5 by Nur77. In contrast, the expression levels of MyoD, MyoG, and mature MYH1 did not show significant changes, suggesting that CSNB treatment mainly promotes the activation of myogenic satellite cells rather than terminal differentiation. Additionally, the expression of fast-twitch myosin MYH4 increased, whereas that of MYH2 (associated with slower muscle fibers) remained unchanged, indicating that CSNB may enhance muscle contractile function by optimizing the composition of muscle fiber types (Fig. 5M). H&E staining revealed a slight increase in the cross-sectional area of muscle fibers in the CSNB mouse group (Fig. 5N). In summary, Nur77 may activate myogenic satellite cells through the positive transcriptional regulation of Myf5.

### Nur77 regulates Myf5 transcription through a K307 acetylation-dependent mechanism

**Fig. 6:**
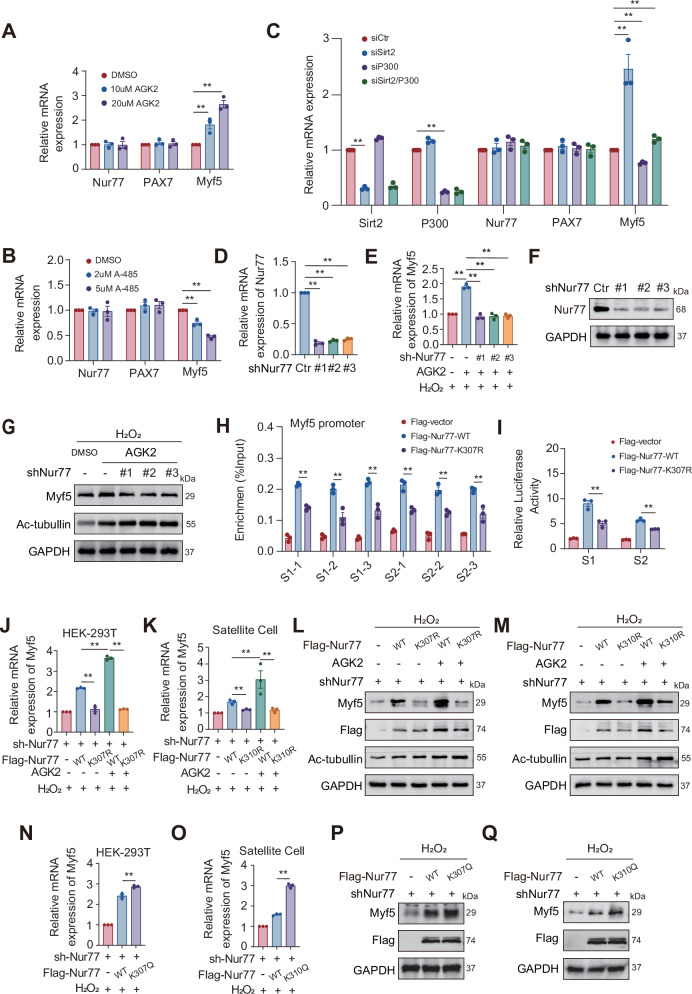
Nur77 regulates Myf5 transcription via a K307 acetylation-dependent mechanism. **A**, **B** RT-qPCR analysis of *Nur77, PAX7*, and *Myf5* mRNA levels in primary MuSCs of the tibialis anterior muscle of 8-month-old WT mice, under concentration gradients of AGK2 (24 h) and A-485 (24 h). *n* = 3 independent experiments. **C** RT-qPCR analysis of *Nur77, PAX7*, and *Myf5* mRNA levels in primary MuSCs of the tibialis anterior muscle of 8-month-old WT mice, after treatment with siSirt2 (50 µM), siP300 (50 µM), and siSirt2/P300 (30/30 µM). *n* = 3 independent experiments. **D**–**G** RT-qPCR and WB analysis of the effect of AGK2 on *Myf5* mRNA and protein levels in primary MuSCs in the presence or absence of Nur77 after H_2_O_2_ stimulation. *n* = 3 independent experiments. **H** ChIP experiment using Flag antibody on HEK-293T cells transfected with Flag-Nur77-WT/K307R to analyze Nur77 enrichment in the promoter region of target genes. *n* = 3 independent experiments. **I** Results of dual luciferase reporter gene assay of HEK-293T cells expressing Flag-Nur77-WT/K307R and WT Myf5. *n* = 3 independent experiments. **J**–**M** RT-qPCR and WB analysis of Myf5 mRNA and protein expression in HEK-293T cells and primary MuSCs of the tibialis anterior muscle of 8-month-old WT mice with or without AGK2 and Flag-Nur77-WT/K307R. **N**–**Q** RT-qPCR and WB analysis of Myf5 mRNA and protein expression in HEK-293T cells and primary MuSCs of the tibialis anterior muscle of 8-month-old WT mice with Flag-Nur77-WT/K307Q. *p < 0.05, **p < 0.01. Error bars represent SEM.

To elucidate the regulatory roles of Sirt2 and P300 on the downstream target gene Myf5 of Nur77, we conducted pharmacological and genetic intervention experiments, which revealed that in primary myoblasts, the Sirt2 inhibitor AGK2 (10 or 20 μM) dose-dependently upregulated Myf5 expression. In contrast, the P300 inhibitor A-485 (2/5 μM) significantly inhibited Myf5 expression. Similarly, in primary satellite cells, siRNA-mediated knockdown of Sirt2 increased Myf5 expression, whereas P300 knockdown decreased Myf5 expression. Co-transfection restored Myf5 levels, whereas Nur77 mRNA levels remained unchanged across all groups (Fig. 6A–C). Therefore, we concluded that Sirt2 and P300 regulate Myf5 expression in primary myoblasts. Subsequently, we performed Nur77 knockout experiments in H₂O₂-treated senescent primary myoblasts and subsequently administered the Sirt2 inhibitor AGK2. AGK2 failed to reverse the decrease in Myf5 expression after Nur77 knockout, indicating that Nur77 is a necessary mediator for AGK2 to regulate Myf5 expression (Fig. 6D–G). To elucidate the regulatory mechanism of K307 acetylation on the transcriptional activity of Nur77, we conducted ChIP-qPCR and dual-luciferase reporter assays in H₂O₂-treated senescent HEK-293T cells. ChIP-qPCR results showed that Flag-Nur77-WT specifically bound to the Myf5 promoter, whereas the binding ability of the K307R mutant was relatively reduced. Dual luciferase reporter results showed that Flag-Nur77-WT increased Myf5 promoter activity, whereas the K307R mutant decreased Myf5 promoter activity (Fig. 6H, I), confirming that K307 acetylation positively regulates the transcriptional activity of Nur77 by enhancing its binding to the Myf5 promoter. To clarify the necessity of K307 or 310 acetylation in the Nur77-mediated transcriptional regulation of Myf5, we conducted functional complementation experiments in Nur77 knockout HEK-293T cells and primary MuSCs. Compared to the complementation of Nur77 WT, the K307 or 310 R mutant exhibited a significantly reduced ability to restore *Myf5* mRNA and protein levels. Additionally, the Sirt2 inhibitor AGK2 only enhanced Myf5 expression in the WT group, with no significant effect on the K307 or 310 R groups. Hence, K307 or K310 acetylation serves as the molecular switch for activating Myf5 transcription by Nur77, and the efficacy of AGK2 depends on the acetylation state of this site (Fig. 6J–M). Subsequently, reintroducing the acetylation-mimetic Nur77-K307/310Q mutant into Nur77-depleted HEK-293T cells and primary MuSCs effectively restored Myf5 expression at both the mRNA and protein levels (Fig. 6N–Q). In conclusion, Sirt2-P300 precisely controls the transcriptional activity of Nur77 by dynamically regulating the K307 acetylation state of Nur77, with K307 acetylation being essential for Nur77 to bind and activate the Myf5 promoter. This modification is critical for the drug sensitivity and functional complementation effects of AGK2, revealing the “acetylation–DNA binding–transcription activation” cascade regulatory mechanism.

### Nur77-K310 acetylation regulates muscle fiber type conversion and fibrosis to maintain muscle homeostasis and regenerative functions

**Fig. 7:**
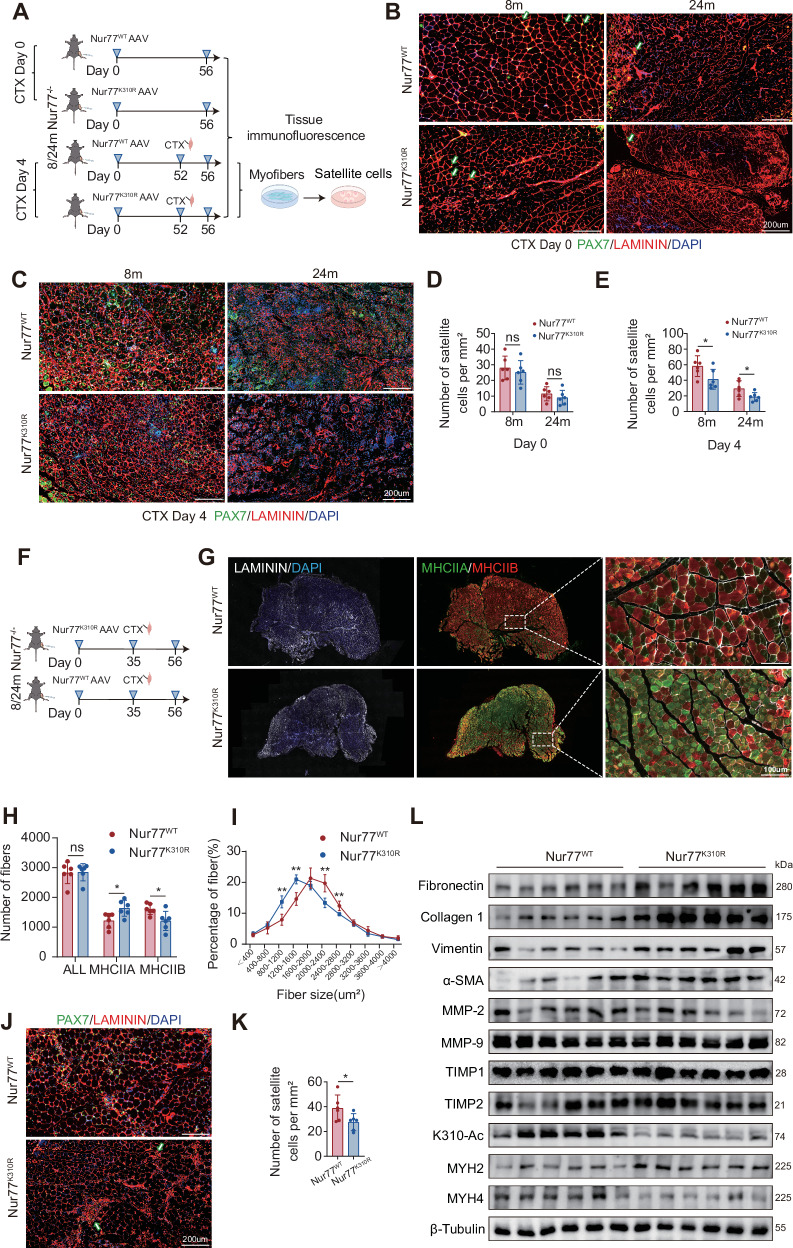
Acetylation of Nur77-K310 regulates muscle fiber type conversion and fibrosis to maintain muscle homeostasis and regenerative functions. **A**–**C** After injecting AAV-Nur77-WT/K310R into the tibialis anterior muscle of 8- and 24-month-old Nur77^−/−^ mice, a CTX injury model was established. Representative immunofluorescence images of tibialis anterior muscle cross-sections on days 0 and 4 after injury (PAX7, green—MuSCs; laminin, red—basement membrane; DAPI, blue—cell nuclei) are shown. Scale bar: 200 μm (*n* = 6 mice). **D**, **E** Quantitative analysis of MuSC number in the tibialis anterior muscle of 8- and 24-month-old Nur77^−/−^ mice after AAV-Nur77-WT/K310R injection and CTX injury at 0 and 4 d; **F**, **G** After injecting AAV-Nur77-WT/K310R into the tibialis anterior muscle of 8-month-old Nur77^−/−^ mice, a CTX injury model was established. Representative immunofluorescence images of tibialis anterior muscle cross-sections 21 d after injury (MHCIIA, green—fast oxidative fibers; MHCIIB, red—fast glycolytic muscle fibers; DAPI, blue—cell nucleus). Scale bar: 200 μm (*n* = 6 mice); **H** Quantitative analysis of the number of ALL/MHCIIA/MHCIIB muscle fibers. **I** Quantitative analysis of the percentage distribution of muscle fibers in different cross-sectional areas (μm²) of the two mouse groups in different intervals (*n* = 6 mice). **J** After injecting AAV-Nur77-WT/K310R into the anterior tibial muscle of 8-month-old Nur77^−/−^ mice, a CTX injury model was established. Representative immunofluorescence staining images of the transverse section of the TA muscle 21 d after injury (PAX7, green—MuSCs; laminin, red—basement membrane; DAPI, blue—cell nuclei). Scale bar: 200 μm (*n* = 6 mice). **K** Quantitative analysis of the number of MuSCs in the anterior tibial muscle of 8- and 24-month-old Nur77^−/−^ mice after AAV-Nur77-WT/K310R injection, 21 d after CTX injury. **L** WB detection of related protein expression in the anterior tibial muscle of 24-month-old Nur77^−/−^ mice after AAV-Nur77-WT/K310R injection and 21 d after CTX-mediated injury onset (*n* = 6 mice). *p < 0.05, **p < 0.01. Error bars represent SEM. See also Fig. S5.

To verify the physiological role of Nur77-K310 acetylation in muscle regeneration, we administered adeno-associated virus (AAV) vectors encoding Nur77-WT or K310R to 8- or 24-month-old Nur77^−/−^ mice for 8 weeks, followed by CTX-induced muscle injury. Tissue immunofluorescence staining with PAX7, LAMININ, and DAPI revealed that before injury (Day 0), compared with the WT group, the number of PAX7^+^ satellite cells in K310R mice showed a downward trend, but no statistical difference was observed; however, the cross-sectional area of muscle fibers in the K310R group was significantly reduced, while the number of muscle fibers remained unchanged (Fig. 7A, B). By day four post-injury, the number of PAX7^+^ satellite cells in the K310R group was lower than that in the WT group (Fig. 7C–E). These changes were more pronounced in the 24-month-old K310R group, where age-associated increases in Sirt2 synergistically exacerbated functional defects. Based on the observed reduction of PAX7^+^ satellite cells in the K310R group four days after CTX injury, we further conducted primary cultures of satellite cells, EdU staining, and Ki67 protein detection. No significant difference in the proliferation rate of satellite cells was observed between the WT and K310R groups (Fig. S5A–C), which confirmed that the reduction in PAX7^+^ cells after injury was due to a change in self-renewal ability rather than a proliferation defect. Hence, Nur77-K310 acetylation regulates the division pattern (self-renewal vs. differentiation) rather than the proliferation rate, determining the maintenance efficiency of the satellite cell pool after injury and providing a new perspective for the development of regenerative therapies targeting stem cell fate. Samples were collected from the anterior tibialis muscle of 8- and 24-month-old Nur77^−/−^ mice after AAV delivery of Nur77-WT or K310R mutant (on Day 0), with CTX injury induced on day 35 and tissue analysis performed on day 56. Immunofluorescence staining showed no difference in the number of muscle fibers between the 8-month-old K310R group and the WT group. However, the proportion of MHCIIA (slow-twitch muscle type) fibers in the K310R group was significantly increased, and that of the MHCIIB (fast-type muscle type) fibers decreased. The cross-sectional area of muscle fibers was also reduced (Fig. 7F–I). Additionally, the number of PAX7^+^ satellite cells was significantly reduced in the K310R group compared to that in the WT group (Fig. 7J, K). These results indicate that the acetylation of Nur77-K310 affects muscle contractile properties by regulating the muscle fiber type (promoting the maintenance of fast-twitch muscle fibers) and ensures regenerative ability by maintaining the satellite cell pool (possibly through regulating self-renewal). Functional defects in these mechanisms jointly lead to abnormal muscle structure and function. In the muscle tissues of 24-month-old Nur77^−/−^ mice supplemented with the K310R mutant, WB analysis revealed that fibrosis-related proteins, including fibronectin, collagen 1, vimentin, and α-SMA, were significantly increased in the K310R group, suggesting enhanced extracellular matrix deposition and exacerbated interstitial fibrosis of muscle fibers. Furthermore, the expression levels of MMP-2 and MMP-9, as well as TIMP1 and TIMP2, remained unchanged between groups, indicating that fibrosis may result from increased matrix synthesis rather than reduced degradation. The expression of the myosin subunit MYH2 (slow-twitch muscle) increased, whereas that of MYH4 (fast-twitch muscle) decreased (Fig. 7L), consistent with the immunofluorescence results from 8-month-old mice (an increase in MHCIIA and a decrease in MHCIIB), confirming that the deacetylation of K310 promotes the transformation of muscle fibers to the slow muscle phenotype. In summary, the acetylation of Nur77-K310 maintains muscle function through dual mechanisms: inhibiting fibrosis at the muscle fiber level and ensuring the expression of the myosin subunit MYH4 in fast-twitch muscle, while also preserving the PAX7^+^ satellite cell pool at the stem cell level. These findings elucidate the integrated mechanism of aging-induced muscle degeneration.

### Sirt2-P300 regulates the division pattern of MuSCs via Nur77 acetylation at K310

**Fig. 8:**
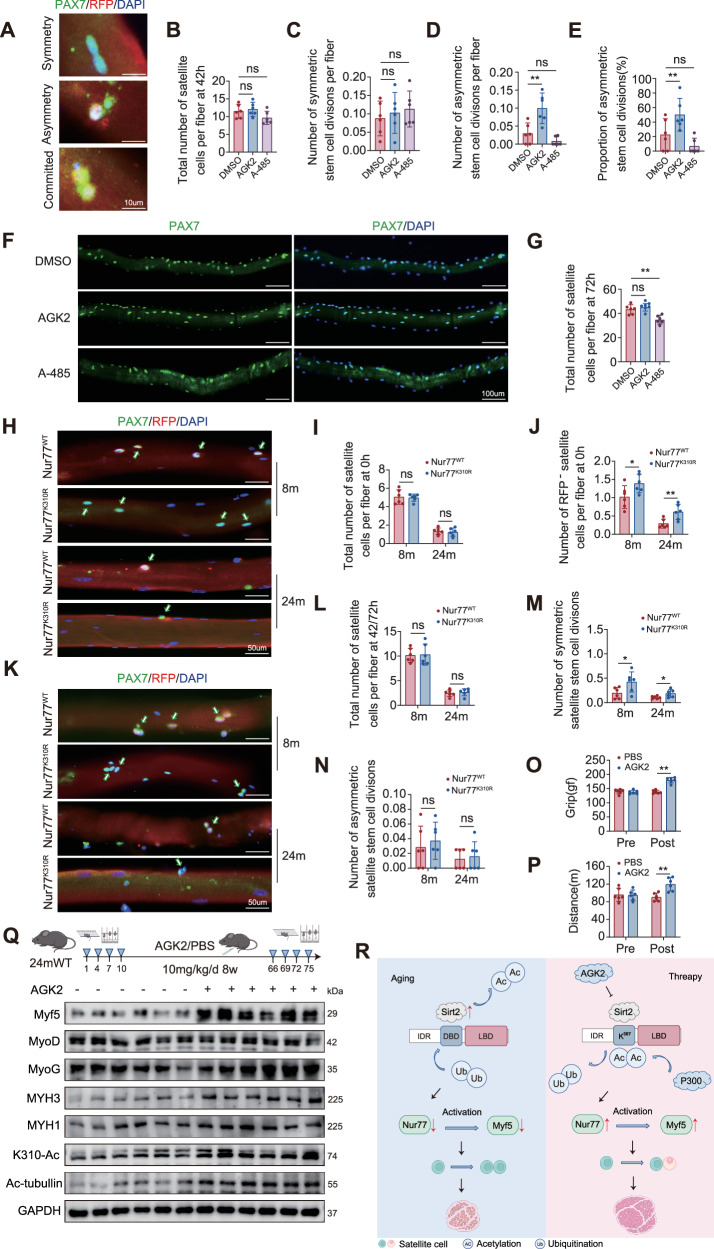
Sirt2-P300 regulates the MuSC division patterns through Nur77 acetylation at K310. **A** Representative immunofluorescence staining images of symmetric (RFP^−^/RFP^−^) and asymmetric (RFP^−^/RFP^+^) dividing satellite stem cells and committed MuSCs that undergo division (RFP^+^/RFP^+^) on single myofibers after 42 h of culture (PAX7, green; RFP, red; DAPI, blue). Scale bar: 10 μm. (*n* = 6 mice, at least 40 myofibers for each condition). **B**–**E** Quantitative analysis of total MuSC number, the number of satellite stem cells undergoing asymmetric and symmetric division, and the percentage of satellite stem cells undergoing asymmetric division on single myofibers after 42 h of culture. **F** Representative immunofluorescence staining image of MuSCs on single myofibers after 72 h of culture (PAX7, green; DAPI, blue). Scale bar: 100 μm. (*n* = 6 mice, at least 40 myofibers for each condition). **G** Quantitative analysis of total MuSC number on single myofibers after 72 h of culture. **H** Representative immunofluorescence staining image of MuSCs on freshly isolated single myofibers (PAX7, green; RFP, red; DAPI, blue). Scale bar: 50 μm. (*n* = 6 mice, at least 40 myofibers for each condition). **I**, **J** Quantitative analysis of MuSC and RFP^−^ satellite stem cell number on freshly isolated MuSCs. **K** Representative immunofluorescence staining image of MuSCs on single myofibers after 42/72 h of culture (PAX7, green; RFP, red; DAPI, blue). Scale bar: 50 μm (cells from 8-month-old mice cultured for 42 h, and those from 24-month-old mice cultured for 72 h; *n* = 6 mice; at least 40 myofibers for each condition). **L**–**N** Quantitative analysis of the total MuSC number and the number of satellite stem cells undergoing asymmetric and symmetric division on single myofibers after 42 h of culture. **O**, **P** forelimb grip strength and running distance after intraperitoneal AGK2 injection (10 mg/kg/d for 8 weeks) in 24-month-old WT mice (*n* = 6 mice). **Q** WB analysis of related protein expression in 24-month-old WT mice after AGK2 administration (10 mg/kg, intraperitoneal injection, 8 weeks). **R** The model shows that AGK2 can rescue the Sirt2 increase caused by aging. Deacetylation at the Nur77-K307/310 site triggers a cascade reaction of “Nur77 protein stability decline – Myf5 transcription reduction – symmetric division of MuSCs – sarcopenia in old age.” *p < 0.05, **p < 0.01. Error bars represent SEM.

The aforementioned results indicate that K310 acetylation is crucial for muscle homeostasis and regeneration. To understand the cellular mechanisms involved, we investigated the regulation of satellite cell fate. Single muscle fibers were isolated from 3-month-old Myf5-Cre/tdTomato mice and cultured in vitro for 42 h to examine the fate of satellite cells during the first division. RFP^−^ satellite stem cells that never expressed Myf5 underwent either symmetric division to expand the stem cell pool or asymmetric division to produce a self-renewing satellite stem cell (RFP^−^) and a committed satellite cell cluster (RFP^+^). Treatment with the Sirt2 inhibitor AGK2 significantly increased the proportion of asymmetric divisions, which originated from an increase in the absolute number of asymmetric divisions rather than a change in the number of asymmetric divisions (Fig. 8A–E). These results are consistent with previous findings that show that Sirt2 deacetylation of Nur77 reduces transcriptional activity, Myf5 expression, and the asymmetric division of satellite cells. Conversely, the P300 inhibitor A-485 reduced the proportion of asymmetric divisions. Hence, Sirt2 and P300 precisely control the division pattern selection of MuSCs by antagonistically regulating the acetylation status of Nur77 (Sirt2 promotes deacetylation, whereas P300 maintains acetylation), providing new insights into the epigenetic regulation of stem cell fate during muscle regeneration. To clarify the long-term effects of Sirt2 and P300 on the population dynamics of MuSCs, we extended the observation period to 72 h. AGK2 treatment did not affect the total number of satellite cells at 42 or 72 h; in contrast, P300 inhibition showed a decreasing trend at 42 hours and led to a significant reduction by 72 h (Fig. 8F, G). This temporal difference indicated that Sirt2 inhibition primarily influenced cell fate by promoting asymmetric division, whereas P300 inhibition gradually impaired the self-renewal capacity of stem cells, ultimately reducing the satellite cell pool. This finding revealed the dynamic role of acetylation in balancing stem cell maintenance and differentiation. To investigate the physiological function of Nur77-K310 acetylation in regulating satellite cell fate, we injected AAV vectors carrying Flag-Nur77-WT or K310R mutants into the anterior muscles of young and old Myf5-Cre/tdTomato mice. Immunofluorescence staining of single muscle fibers at 0 h post-isolation showed that, although the total number of satellite cells (PAX7^+^) in the WT and K310R groups was not significantly different within the same age group, the proportion of RFP^−^ satellite cells in the K310R group was significantly increased. This effect was more pronounced in the older mice (Fig. 8H–J), suggesting that under steady-state conditions without muscle damage, an age-related increase in Sirt2 may enhance K310 deacetylation, altering satellite stem cell fate decisions during division. To investigate the age-dependent effect of Nur77-K310 acetylation on the division pattern of satellite cells, we analyzed the fate of the first division in young (42 h) and old (72 h) Myf5-Cre/tdTomato mouse models after AAV delivery. No significant difference was observed in the total number of satellite cells (PAX7^+^) and the number of asymmetric divisions between the WT and K310R mutants within the same age group; however, the number of symmetrically dividing satellite stem cells in the K310R group was significantly increased (Fig. 8K–N). Nur77 acetylation did not affect cell proliferation. The absence of Nur77 acetylation led to a beneficial alternative self-renewal cell fate decision at the expense of commitment, likely owing to changes in the regulation of Nur77 target genes in the body. In conclusion, the Sirt2 inhibitor AGK2 promotes asymmetric division by enhancing Nur77 acetylation, whereas P300 inhibition impairs stem cell maintenance. In vivo experiments confirmed that K310 deacetylation (K310R mutant) specifically increases symmetric division, elucidating the age-dependent mechanism by which the dynamic balance of acetylation regulates the division pattern and affects the efficiency of muscle regeneration. We validated AGK2 (10 mg/kg, intraperitoneal injection for 8 weeks) treatment in 24-month-old WT mice based on previously described methods [31–33]. In terms of behavior, the grip strength of the AGK2 group was higher than that of the control group, and the running distance increased (Fig. 8O, P). At the molecular level, the expression of K310-acetylated Nur77, Myf5, and MYH3 proteins significantly increased, whereas MyoD or MyoG levels did not change (Fig. 8R). These results directly translate previous mechanistic findings, confirming that targeting the Sirt2-Nur77-K310 acetylation axis can specifically activate the Myf5 pathway and improve the function of aging muscles, providing experimental evidence for the treatment of sarcopenia (Fig. 8R).

## Discussion

We systematically revealed the core regulatory role of the Sirt2-P300/Nur77/K310 acetylation axis in muscle homeostasis and regeneration, providing a novel molecular perspective on aging-related diseases. Nur77 expression declines in multiple tissue organs, such as the liver, brain, heart, ovaries, and kidneys [7, 34–38]. The anterior tibialis is a fast-twitch muscle composed mainly of large glycolytic type IIB fibers and smaller oxidative glycolytic type IIA fibers [39]. In this study, we confirm that a similar reduction occurs in the skeletal muscle system, further emphasizing the crucial role of Nur77 in the aging process. We identified K310 acetylation of Nur77 as a key post-translational modification node, and its dynamic balance directly influences the protein stability of Nur77. Sirt2 and P300 antagonistically regulate this acetylation site, thereby modulating the transcriptional activity of Nur77 in a site-specific and evolutionarily conserved manner. Notably, Sirt2, as a deacetylase, exhibits distinct tissue-specific functions; in the muscle system, its overactivation impairs muscle regeneration by promoting Nur77 deacetylation, whereas in the nervous system, Sirt2 inhibition has demonstrated protective effects against neurodegenerative diseases, such as Parkinson’s and Alzheimer’s [40–42]. This tissue specificity may stem from differences in Sirt2 substrates: in the muscle, Sirt2 mainly regulates Nur77 and affects the function of MuSCs; in neurons, it acts on α-synuclein, Tau, and other proteins, regulating their aggregation state. These findings provide an important theoretical foundation for developing tissue-targeted Sirt2 regulators. Therefore, we are prompted to contemplate a deeper question: Does the core mechanism revealed in this study—namely, “Sirt2 driving functional loss by deacetylating key proteins”—constitute a conserved regulatory paradigm across multi-organ aging? Although the specific substrates of Sirt2 vary across different tissues, its catalytic essence and downstream consequences—altering substrate stability, activity, or aggregation state via deacetylation—are fundamentally shared. Therefore, we propose an extended hypothesis: Sirt2 may serve as a common senescence-associated pathway that collaboratively promotes multi-organ degeneration by deacetylating respective key target proteins in different tissues. Based on this hypothesis, future research is urgently needed to systematically map Sirt2’s substrate profiles and their dynamic modification patterns across diverse tissue environments, and to validate the universality of the Sirt2-Nur77 axis in other aging organs such as the liver and heart.

Static satellite cell populations show significant metabolic heterogeneity, with the satellite stem cell subpopulation exhibiting a more dormant state and lower metabolic activity [1, 43]. Single-cell-level studies have further reported the molecular and functional diversity within the quiescent satellite cell population, and this heterogeneity is closely associated with functional decline during aging. Stressors such as muscle damage can lead to a reduction in the diversity of satellite cell clones, thereby activating stem cells that are considered to be at the top of the hierarchical structure [44, 45]. Moreover, the self-renewal potential of satellite cells decreases with age [5], partly due to changes in the rates of symmetric and asymmetric stem cell divisions. We revealed that acetylation regulation mediated by Sirt2-P300 is a key factor in determining metabolic heterogeneity and self-renewal ability. From the perspective of metabolic regulation, the Sirt2-P300/Nur77 acetylation axis may be an important link connecting the cell metabolic state and the maintenance of stemness. This provides a new paradigm for understanding how the “metabolism-epigenetics axis” precisely regulates stem cell fate, a frontier field that emphasizes how metabolic states dynamically influence key protein functions via modifying enzymes to determine cellular destiny [46–49].

By regulating the activity of its dependent deacetylase Sirt2, NAD + , as a core metabolite within this axis, serves as a key nexus linking cellular metabolic status to epigenetic modifications. Modulating Sirt2 levels can significantly affect the metabolic patterns of satellite cells, which is consistent with previous studies demonstrating that Sirt2 promotes glycolysis in other stem cell types [50–52]. An elevated glycolysis level is a typical metabolic feature of satellite cell activation and differentiation [53]. In the anterior tibialis muscle, which is mainly composed of type IIB and IIA fast-twitch muscle fibers, this metabolic–epigenetic regulatory network shows unique sensitivity, which may be one of the reasons that fast-twitch muscle fibers are more susceptible to atrophy during aging. Notably, we found that disrupting the acetylation balance of Nur77 (via the K310R mutation) is sufficient to drive fiber type remodeling in the tibialis anterior muscle, manifested as an increased proportion of type IIA fibers and a decrease in type IIB fibers. Classic age-related muscle atrophy is characterized by the selective loss of type II (especially IIB) fast-twitch fibers and a relative increase in type I slow-twitch fibers [49, 54]. Although we did not directly examine changes in type I fibers (MYH7 expression), our findings propose a new mechanistic framework: the Sirt2-P300/Nur77-K310 axis serves as a critical node regulating the balance of muscle fiber types, particularly in initiating the internal remodeling of type II fibers (the conversion from IIB to IIA). This suggests that such early subtype conversion, driven by epigenetic dysregulation and confined to the fast-twitch fiber pool, may constitute a precursor step or one of the molecular foundations for the broader global shift from “fast-twitch to slow-twitch” fiber types during aging. Future research should include systematic evaluations of whether this regulatory axis also directly influences the proportion of type I fibers, and whether such effects are achieved by determining the differentiation fate of satellite cells or driving metabolic reprogramming of existing muscle fibers. In addition to its long-term effects on muscle fiber types, the Sirt2-Nur77 axis also directly regulates the immediate regenerative capacity of satellite cells. From the perspective of self-renewal, we demonstrated that inhibiting Sirt2 can enhance muscle regeneration through Nur77-K310 acetylation. AGK2 treatment significantly increased the K310 acetylation levels of Nur77, promoted Myf5 expression and the recovery of muscle function, and improved the self-renewal capability of satellite cells. Additionally, the Nur77 agonist, CSNB, was shown to alleviate symptoms of sarcopenia in older adults, offering a new strategy for clinical treatment. Neurological studies have also indicated that Sirt2 inhibition provides a protective effect against neurodegenerative diseases [55, 56]. This dual effect suggests that targeting the Nur77 pathway may become a potential strategy for intervening in multiple systems of aging.

During aging, the dynamic balance of Sirt2 activity is disrupted, leading to multisystem dysfunction [8, 57]. In our research, we further elucidate its specific action cascade in skeletal muscle aging. Experiments demonstrate that in skeletal muscle, the dynamic acetylation of the Nur77-K310 site mediated by Sirt2/P300 directly regulates the core metabolic state of satellite cells. This metabolic reprogramming subsequently determines the fate decisions of stem cells, ultimately manifesting in the regenerative capacity and homeostatic maintenance of skeletal muscle. The elucidation of this “acetylation-metabolism-fate-function” axis provides an integrative mechanistic framework for understanding muscle aging and reveals multiple potential intervention points.

This study provides important insights for targeted interventions from the perspective of translational medicine. First, the acetylation status of Nur77-K310 may serve as a biomarker for evaluating the degree of muscle aging. Second, any intervention strategy targeting the Sirt2-Nur77 regulatory axis needs to consider tissue specificity. Ideally, allosteric regulators should be developed to differentiate between muscle and nerve effects. Finally, the universal decline of Nur77 in multiple organs and the therapeutic effect of its agonist CSNB suggest the need for a more comprehensive aging research framework that incorporates degenerative changes across different organ systems.

However, our data indicate that after CTX injury, the expression of PAX7 protein in Nur77 knockout mice decreased, whereas Nur77 did not affect the mRNA level of PAX7. We hypothesize that Nur77 may regulate the protein level of PAX7 through non-transcriptional mechanisms under stress conditions. This hypothesis should be verified through subsequent work.

## Materials and methods

### Animal model

In this study, we used C57BL/6 background Nur77 knockout mice (Nur77 − /−) and their wild-type littermates, as well as muscle-specific Nur77 knockout mice (Myf5-Cre:Nur77^flox/flox^, CKO and Myf5-Cre:tdTomato reporter mice obtained through hybridization (mouse strain information is provided in Table S3). All mice were housed under specific pathogen-free (SPF) conditions at our institution, maintained at 23 ± 2 °C, on a 12-h light/dark cycle, with free access to food and water.

Experimental animals were allocated to groups using a randomization procedure. Specifically, after genotype identification, a researcher not involved in subsequent behavioral tests or data analysis used a computer-generated random number table to randomly assign wild-type and knockout mice from the same litter and of the same sex into different experimental groups.

Blinding: Due to the inherently identifiable nature of the experimental interventions, the researchers performing the procedures could not be blinded to group allocation. To minimize assessment bias, blinding was implemented during critical phases of outcome evaluation: histological analysis, image quantification, and related assessments were independently performed by researchers unaware of the experimental groupings. Whenever feasible, the execution of behavioral tests was also conducted by personnel not involved in animal allocation.

The experiments were conducted by local injection of AAV9-CMV-Flag-Nur77-WT/K310R (1 × 10¹² vg/μl) into the anterior tibialis muscle for 8 weeks for gene supplementation and by intraperitoneal injections of AGK2 (10 mg/kg/d; 8 weeks) or CSNB (10 mg/kg/d; 4 weeks). The muscle injury model was established by injecting 50 μl of 10 μM CTX into multiple sites of the anterior tibialis muscle, and samples were collected for assessment of the regeneration process on days 0, 4, 7, 15, and 21 post-injury. Behavioral tests included three weekly running wheel experiments, with the average activity value recorded, and grip strength tests performed five times per mouse, with the maximum value recorded. The running wheel experiment used a mouse rotation fatigue apparatus set at a 15° incline, 20 rpm speed, and a total duration of 50 min. Exhaustion parameters included a 0.3 mA current and a 5-s shock tolerance time. The experiment was automatically terminated when the mice reached all three fatigue levels. All animal procedures were approved by the Animal Ethics Committee of Shengjing Hospital, affiliated with China Medical University (Approval Number: 2023PS1235K).

### Mouse genotype identification

Genomic DNA extracted from the tails of the mice was used in this study. The specific operation steps were as follows: 1–2 mm of mouse tail tissue was harvested, and DNA was extracted. The PCR reaction program was as follows: 94 °C for 2 min of pre-denaturation, 10 cycles of drop PCR (94 °C for 20 s, 65 °C for 15 s with a 0.5 °C decrement per cycle, and 68 °C for 10 s), 28 standard cycles (94 °C for 15 s, 60 °C for 15 s, 72 °C for 10 s), and finally storage at 10 °C. The PCR products were analyzed by agarose gel electrophoresis to confirm the genotypes.

### Morphological analysis of the anterior tibialis muscle tissue

We used standard paraffin sectioning technology to assess the morphology of the anterior tibialis muscle tissue. The specific methods were as follows:

Tissue fixation and sectioning: The anterior tibialis muscle samples were fixed in 4% paraformaldehyde for 24 h, dehydrated with a gradient of ethanol, and embedded in paraffin. Sections were cut continuously (thickness: 4 μm) using a sectioning machine and adhered to anti-detachment glass slides.

H&E staining: Routine H&E staining was performed to evaluate the muscle fiber structure and regeneration status. Multiple immunofluorescence staining was performed using a combination of antibodies against PAX7 (stem cell marker), laminin (basement membrane marker), and DAPI (nuclear staining) to mark the distribution of satellite cells and the boundaries of muscle fibers.

Image acquisition and analysis: Images were obtained under an optical microscope (Nikon Eclipse Ni-E, Nikon, Tokyo, Japan), and quantitative analysis was performed using ImageJ software (NIH, Bethesda, MD, USA). Parameters measured included muscle fiber cross-sectional area, laminin + muscle fiber outlines, and the proportion of centrally nucleated fibers, a regeneration marker. Satellite cell density was quantified as the number of PAX7^+^ cells per muscle fiber.

### Cell line

HEK-293T cells were used for Co-IP, chromatin immunoprecipitation (ChIP), and dual luciferase reporter assays. C2C12 myoblasts were used for immunofluorescence co-localization experiments. Both cell lines were cultured in high-glucose Dulbecco’s Modified Eagle Medium, containing 10% fetal bovine serum (FBS) + 1% penicillin or streptomycin. Cells were maintained at 37 °C in a 5% CO₂ incubator. Mycoplasma contamination was routinely monitored and confirmed to be negative (cellular information is shown in Table S3.

### Isolating and culturing single muscle fibers

Single muscle fibers were isolated from the anterior tibial muscle by digesting with 0.2% collagenase I at 37 °C for 60–90 min, followed by gentle disruption to release the muscle fibers. Subsequently, the isolated fibers were cultured in high-glucose DMEM supplemented with 10% FBS, 10% horse serum, 1% chicken embryo extract, and 4 mM glutamine. The treatment groups received 10 μM AGK2 (Sirt2 inhibitor) or 2 μM A-485 (P300 inhibitor), whereas the control group was given an equal volume of DMSO. At least 40 muscle fibers were analyzed under each treatment condition to evaluate satellite cell activation (PAX7^+^ or Myf5^+^ expression), division patterns (symmetric or asymmetric), and other indicators.

### Extracting and culturing primary satellite cells

Primary satellite cells were extracted from the anterior tibial muscle using magnetic bead sorting, after which the tissue was minced and digested with a mixture of collagenase and dispersion enzymes, filtered, centrifuged to remove red blood cells, and resuspended in MACS buffer (0.5% BSA + 2 mM EDTA in PBS). Successive negative screening was performed using CD11b/SCA-1/CD45/CD31 antibody-conjugated magnetic beads to remove hematopoietic and endothelial cells, followed by positive screening using α7-integrin magnetic beads (Miltenyi Biotec, Gladbach, Germany) to obtain the final cells. The purified cells were seeded onto collagen-coated culture dishes and into Ham’s F-10 medium supplemented with 20% FBS, 1% chicken embryo extract, and 2.5 ng/ml bFGF. Differentiation was induced using 50% Ham’s F-10 + 50% DMEM containing 1% double anticoagulant, 5% horse serum, and 10 μg/ml insulin.

### Co-IP assay

HEK-293T cells were transfected with specific plasmids or lentiviruses using Lipofectamine 2000 (Invitrogen, Thermo Fisher Scientific, Waltham, MA, USA). After 48 h, the cells were collected and lysed in Bio-Rad’s weak RIPA lysis buffer containing protease inhibitors (Bio-Rad Laboratories, Hercules, CA, USA) for 30 min. Lysates were centrifuged, and the protein concentration of the supernatants was determined using the BCA method. Protein concentrations were normalized across samples. Lysates were incubated at 4 °C overnight with specific primary antibodies, followed by a 4-h incubation with Protein A/G magnetic beads. The bead-bound complexes were washed thrice with lysis buffer and eluted by boiling in 2×SDS loading buffer. Subsequently, the proteins were subjected to western blot analyses.

### In vitro GST pull-down assay

A glutathione S-transferase-tagged fusion protein expression plasmid was constructed and transformed into competent BL21 (DE3) cells (Takara Biosciences, Shiga, Japan). The target protein was expressed by induction with 1 mM IPTG at 30 °C for 6 h. The bacterial pellet was resuspended in lysis buffer (50 mM Tris-HCl, pH 8.0, 20% glucose, 10% glycerol, 2 mM MgCl₂), sonicated, and purified using glutathione agarose 4B beads (Cytiva, Marlborough, MA, USA). The purified protein was incubated with a Flag-tagged target protein synthesized via an in vitro transcription or translation system in the binding buffer (20 mM Tris-HCl, pH 7.5, 50 mM NaCl, 10% glycerol, 1% NP-40) at 4 °C for 4 h. The complexes were washed three times with binding buffer, eluted with 2×SDS loading buffer, and subjected to western blot analysis using an anti-Flag antibody (1:2000).

### ChIP assay

The standard ChIP experimental protocol was as follows: cells were cross-linked with 1% formaldehyde in serum-free DMEM for 10 min, treated with 0.125 M glycine for 5 min, washed with PBS, and lysed in ChIP lysis buffer (50 mM Tris-HCl pH 8.0, 10 mM EDTA, and 1% SDS). The chromatin was sonicated to fragments of 200 − 500 bp (30 s on or 30 s off, 15 cycles), diluted 10 times with ChIP dilution buffer (16.7 mM Tris-HCl pH 8.0, 167 mM NaCl, 1.2 mM EDTA, 0.01% SDS, 1.1% Triton X-100), then incubated with pre-coated Protein A/G agarose beads and a specific antibody for 2 h. The immune complexes were washed, de-crosslinked, and analyzed by qPCR to determine the degree of target sequence enrichment. Table S4 presents the primer sequences. IgG isotype and input controls were set, and the data were expressed as a percentage of the input (calculated using the 2^−ΔΔCt^ method).

### Double luciferase reporter gene assay

HEK-293T cells stably expressing Flag-Nur77-WT or K307R, were co-transfected with the pGL3-Myf5 promoter-luciferase reporter vector or the pRL-TK-Renilla luciferase reference vector at a mass ratio of 20:1 using Lipofectamine 2000. Luciferase activity was measured using a Promega Dual-Luciferase Reporter Gene Detection System 48 h after transfection. Firefly luciferase activity (reflecting Myf5 promoter activity) was first measured by adding Luciferase Assay Reagent II, followed by the addition of Stop & Glo® Reagent to quench and detect the Renilla luciferase activity. The data were expressed as the ratio of firefly to Renilla luciferase activity (normalized to the reference). Each group included three technical replicates, and the experiment was independently repeated three times.

### Acetylation site mass spectrometry analysis

Purified human Nur77 protein was subjected to reduction with 10 mM DTT at 56 °C for 30 min and alkylation with 55 mM IAA at room temperature in the dark for 30 min. The reaction product was desalted and freeze-dried and then incubated in an acetylation buffer at 37 °C for 20 h. The acetylated product was diluted and re-solubilized in 0.1% formic acid solution and analyzed using ultra-high-performance liquid chromatography (Easy-nLC 1000, Thermo Fisher Scientific) coupled with high-resolution mass spectrometry (Q Exactive HF-X, Thermo Fisher Scientific). Chromatographic separation was performed on a 75 μm × 25 cm reversed-phase column with a 120 min gradient (5 − 35% acetonitrile in 0.1% formic acid). Mass spectrometry was conducted in positive ion mode with resolutions of 70000 (MS1) and 17500 (MS2), using a higher-energy collisional dissociation fragmentation energy of 28%. Raw data were retrieved from the UniProt human database (containing common contaminants) using MaxQuant (v1.6.14), setting acetylation (K + 42.0106 Da) as a variable modification, carbamidomethylation as a fixed modification, and a false discovery rate of <1%.

### Real-time quantitative PCR (qPCR) analysis

Total RNA was extracted using the RNAiso Plus reagent, and genomic DNA was removed with the gDNA Eraser. Subsequently, 1 μg of RNA was reverse transcribed into cDNA using a PrimeScript RT kit. The qPCR was performed in a 20 μl reaction system containing SYBR Premix Ex Taq II, 10 μM forward and reverse primers, and a 1:10 diluted cDNA template. The reaction procedure was as follows: 95 °C for 30 s (pre-denaturation), followed by 40 cycles of 95 °C for 10 s and 60 °C for 30 s, and a final melting curve analysis (95 °C for 15 s, 60 °C for 1 min, 95 °C for 15 s). All reactions were conducted on a QuantStudio 6 Flex system, with three technical replicates and a no-template control (NTC). Relative mRNA expression levels were calculated using the 2^−ΔΔCt^ method, with *GAPDH* as the internal reference. Primer efficiency (90–110%) was confirmed via a standard curve using 5-fold serial dilutions. Key quality control measures included a single peak in the melting curve to confirm amplification specificity, and only samples with Ct values < 35 were included in the analysis. Table S4 lists the primer sequences.

### Statistical analysis

We did not use a priori statistical methods to determine the sample size. All experiments were conducted with at least three independent biological replicates. Figure legends specify the replication times. Data are presented as mean ± standard error (SEM). Co-IP and western blot experiments were independently repeated at least three times, yielding consistent results. The experimental design included user blinding where feasible. Statistical analysis was based on the following data types: 1) two-tailed Student’s t-test (a paired t-test was applied to paired samples and a non-paired t-test was applied to non-paired samples) and 2) one-way analysis of variance for comparisons among multiple groups. SPSS software (version 26.0) was employed for analysis. Statistical significance was set at *p* < 0.05, *p < 0.01. For quantitative analyses of western blot and immunofluorescence data, parametric tests were applied after confirming a normal distribution (Shapiro−Wilk test) and homogeneity of variance (Levene’s test).

### Ethics

All animal procedures were approved by the Animal Ethics Committee of Shengjing Hospital, affiliated with China Medical University (Approval Number: 2023PS1235K). The animal experiments were strictly conducted in accordance with the guidelines and policies of the Animal Center of Shengjing Hospital, China Medical University, and followed the ARRIVE guidelines.

## Supplementary information

Supplemental information

Figure S1

Figure S2

Figure S3

Figure S4

Figure S5

western blots

aj-checklist

## Data Availability

All data are available in the main text or the Supplementary Materials. All unique/stable reagents generated in this study are available from the corresponding authors without restriction. This paper does not report original code.
