# Supplementary material for: The Sirt2–Nur77 axis regulates muscle stem cell quiescence and senescence via epigenetic–metabolic synergy
Source: Cell Death Dis. 2026 Mar 28;17(1):429. doi: 10.1038/s41419-026-08645-w (PMC13153389; doi:10.1038/s41419-026-08645-w)
Supplement: Supplementary file 1 — Supplemental information [file 41419_2026_8645_MOESM1_ESM.docx]

**SUPPLEMENTAry MATERIAL**

**for**

**The Sirt2–Nur77 axis regulates muscle stem cell quiescence and senescence via epigenetic–metabolic synergy**

Yanteng Wang, Yichen Yang, Wan Yu, Yue Liu, Wenwei Guan, Yingxi Wang, Na Li^✉^, Liu Cao^✉^, Difei Wang^✉^

**Figure S1. Sirt2 binds to the Nur77 DBD domain and induces deacetylation of Nur77 in the nucleus. A.** WB analysis of relevant proteins in the anterior tibialis muscle of 8- and 24-month-old WT female mice (n = 6 mice). **B–E.** Co-IP demonstrated the interaction between endogenous Nur77 and Sirt2 in primary MuSCs and anterior tibialis muscle. IgG, immunoglobulin. **F.** Co-IP of Flag- Nur77 fragment and Myc- Sirt2 in HEK-293T cells. **G–J.** RT-qPCR was used to detect the expression of *Nur77* mRNA after overexpression or knockdown of Sirt2 in HEK-293T and primary MuSCs (n = 3 replicate experiments). **K.** Co-IP shows the interaction between Nur77 and P300/CBP/PCAF/GCN5 in HEK-293T cells. **L, M.** In HEK-293T cells, Co-IP showed the acetylation of external and internal Nur77 by Flag-Sirt2-WT/MUT. n = 3 independent experiments. **N, O.** In HEK-293T cells, Co-IP showed the acetylation levels of the full-length Nur77 and various recombinant fragments. **P.** Nuclear**–**cytoplasmic separation and Co-IP in HEK-293T cells detected the acetylation level of exogenous Nur77 by AGK2 (10 μM, 24 h). *p < 0.05, **p < 0.01. Error bars represent SEM.

**Figure S2. Sirt2 regulates the stability of Nur77 protein through a K307-site acetylation–ubiquitination cascade. A.** WB was used to detect the expression levels of Nur77 protein in primary MuSCs from HEK-293T and 8-month-old mice in the gradient AGK2 (10 μM for 24 h) and the presence or absence of H_2_O_2_ (500 μM for 1 h) (n = 3 replicate experiments). **B–G.** WB was used to detect and quantify the exogenous Nur77 protein levels in primary MuSCs from HEK-293T and 8-month-old WT mice under Myc-Sirt2 (or 10 μM AGK2 for 24 h) and 100 μg/mL actinomycin D CHX (n = 3 independent experiments). **H, I.** Co-IP showed the ubiquitination level of exogenous Nur77 in HEK-293T cells under Myc-Sirt2 (or 10 μM AGK2 for 24 h). n = 3 independent experiments. **J.** Co-IP showed the ubiquitination levels of Flag-Nur77-WT, K307R, K536R, and K307/536R. n = 3 independent experiments. **K–N.** Quantitative analysis of the protein levels of Flag-Nur77-WT, K307(310)R, and K307(310)Q in primary MuSCs from HEK-293T and 8-month-old WT mice in response to 100 μg/mL actinomycin D CHX. *p < 0.05, **p < 0.01. Error bars represent SEM.

**Figure S3. Nur77 shows an age-related decline in the anterior tibial muscle of mice, and the knockout of Nur77 leads to a sarcopenia phenotype. A.** Genotype identification diagram of WT and Nur77^−/−^ mice; **B.** Representative HE staining images of the anterior tibial muscle cross-sections of 8- and 24-month-old WT and Nur77^−/−^ mice. Scale: 200 μm (n = 6 mice). **C, D.** Representative HE staining images of the anterior tibial muscle cross-sections of 6-month-old Nur77^flox/flox^ and Nur77 CKO (Myf5-Cre^+^ Nur77^flox/flox^). Scale: 200 μm (n = 3 mice). **E, F.** Statistical analysis of the total number of muscle fibers and cross-sectional areas (μm²) in the anterior tibialis cross-sections of 6-month-old Nur77^flox/flox^ and Nur77 CKO mice. (n = 3 mice). *p < 0.05, **p < 0.01. Error bars represent SEM.

**Figure S4. Nur77 regulates Myf5-mediated activation of MuSCs and improves the dysfunction of aging-related muscle regeneration. A.** After 1 h of inducing senescence stress with H₂O₂ on HEK-293T cells and MuSCs, WB was used to detect the protein levels of Nur77, Sirt2, p53, and p21. n = 3 independent experiments. **B.** WB detection of Ki67 protein level in primary MuSCs isolated from anterior tibialis muscles of 8- and 24-month-old WT and Nur77^−/−^ mice. **C.** WB analysis was performed to examine the protein expression levels of relevant genes in MuSCs during various stages of differentiation. **D.** Nuclear**–**cytoplasmic separation and WB were used to detect the expression levels of Nur77 protein in the primary MuSCs of the anterior tibial muscles of 8-month-old WT mice during proliferation and differentiation for 3 d. **E.** WB analysis validating the Nur77-dependency of CSNB's effect on Myf5 protein levels in H₂O₂-stimulated primary MuSCs. n = 3 independent experiments. *p < 0.05, **p < 0.01. Error bars represent SEM.

**Figure S5. Acetylation of Nur77-K310 regulates muscle fiber type conversion and fibrosis to maintain muscle homeostasis and regenerative functions. A.** Representative immunofluorescence staining images of primary MuSCs isolated from the TA muscle of 8- and 24-month-old Nur77-WT/K310R mice. EdU, red; PAX7, green; DAPI, blue. The red arrow indicates cells that are positive for both EdU and PAX7. Scale bar: 100 μm. n = 3 independent experiments. **B.** Quantitative analysis of the percentage of EDU^+^/PAX7^+^ in primary MuSCs 4 d after CTX injury in the tibialis anterior muscle of 8- and 24-month-old Nur77^−/−^ mice after injection of AAV-Nur77-WT/K310R. **C.** WB detection of Ki67 protein expression level in primary MuSCs 4 days after CTX injury in the tibialis anterior muscle of 8- and 24-month-old Nur77^−/−^ mice after injection of AAV-Nur77-WT/K310R. *p < 0.05, **p < 0.01. Error bars represent SEM.

Table S1. Amino acid sequence alignment of Nur77 K307 and K536-containing regions

| Species | Sequence | Sequence |
| --- | --- | --- |
| Hs(598aa) | YICLANKDCPVDKRRRNRCQ(K307) | PRRVEELQNRIASCLKEHVA(K536) |
| Mus.m(601aa) | YICLANKDCPVDKRRRNRCQ(K310) | PRRVEELQNRIASCLKEHMA(K539) |
| Rn(597aa) | YICLANKDCPVDKRRRNRCQ(K306) | PRRVEELQNRIASCLKEHMA(K535) |
| Cf(598aa) | YICLANKDCPVDKRRRNRCQ(K307) | PRRVEELQNRIASCLKEHVS(K536) |
| Oa(598aa) | YICLANKDCPVDKRRRNRCQ(K307) | PRRVEELQNRIASCLKEHVS(K536) |
| Fc(598aa) | YICLANKDCPVDKRRRNRCQ(K307) | PRRVEELQNRIASCLKEHVS(K536) |
| Bt(598aa) | YICLANKDCPVDKRRRNRCQ(K307) | PRRVEELQNRIASCLKEHVS(K536) |
| Pt(598aa) | YICLANKDCPVDKRRRNRCQ(K307) | PRRVEELQNRIASCLKEHVA(K536) |
| Mac.m(598aa) | YICLANKDCPVDKRRRNRCQ(K307) | PRRVEELQNRIASCLKEHVA(K536) |

Abbreviations: Hs, *Homo sapiens*; Mus.m, *Mus musculus*; Rn, *Rattus norvegicus*; Cf, *Canis lupus* *familiaris*; Oa, *Ovis aries*; Fc, *Felis catus*; Bt, *Bos taurus*; Pt, *Pan troglodytes*; Mac.m, *Macaca mulatta*.

Table S2. The predicted sequence of Nur77 that binds to Myf5

| Name | Score | Relative score | Start | End | Strand | Predicted sequence |
| --- | --- | --- | --- | --- | --- | --- |
| Nur77 | 9.32 | 0.89 | 690 | 699 | - | TTAAGGTCTA |
| Nur77 | 8.81 | 0.88 | 1493 | 1500 | + | AAATGCCA |
| Nur77 | 8.15 | 0.87 | 392 | 401 | + | TCAAGGTCTT |
| Nur77 | 8.82 | 0.87 | 1491 | 1502 | + | GAAAATGCCACT |
| Nur77 | 7.70 | 0.86 | 811 | 818 | + | AAATGACA |
| Nur77 | 7.70 | 0.861 | 1054 | 1061 | + | AAATGACA |
| Nur77 | 7.70 | 0.86 | 1406 | 1413 | + | AAATGACA |
| Nur77 | 8.13 | 0.85 | 809 | 820 | + | TTAAATGACAAC |
| Nur77 | 7.64 | 0.85 | 1404 | 1415 | + | ATAAATGACAAT |
| Nur77 | 7.27 | 0.84 | 1052 | 1063 | + | AGAAATGACAGG |
| Nur77 | 5.94 | 0.83 | 1561 | 1568 | + | GAAGTTCA |
| Nur77 | 6.19 | 0.82 | 1559 | 1570 | + | TTGAAGTTCATG |
| Nur77 | 5.00 | 0.82 | 1560 | 1569 | + | TGAAGTTCAT |
| Nur77 | 4.84 | 0.81 | 290 | 299 | - | AAAAGATCTC |
| Nur77 | 5.19 | 0.81 | 426 | 433 | + | AAAGGTTG |
| Nur77 | 4.61 | 0.81 | 425 | 434 | + | AAAAGGTTGC |
| Nur77 | 4.94 | 0.81 | 1844 | 1851 | + | AAGGGGCA |
| Nur77 | 4.93 | 0.81 | 504 | 511 | + | AAAGTACA |
| Nur77 | 4.79 | 0.81 | 691 | 698 | - | TAAGGTCT |
| Nur77 | 5.45 | 0.81 | 502 | 513 | + | AAAAAGTACAAA |
| Nur77 | 4.62 | 0.80 | 1025 | 1032 | + | AAAGCCCA |
| Nur77 | 5.21 | 0.80 | 424 | 435 | + | GAAAAGGTTGC |

Table S3. Reagent

| REAGENT | SOURCE | IDENTIFIER |
| --- | --- | --- |
| Antibodies | | |
| Rabbit Monoclonal anti-Nur77 | Novus (USA) | NBP2-66980 |
| Rabbit anti-Nur77 | Novus (USA) | NB10056745 |
| Mouse Monoclonal anti-Nur77 | SantaCruz (USA) | sc-365113 |
| anti-Nur77 K307/310Ac | This manuscript | N/A |
| anti-Nur77 K536Ac | This manuscript | N/A |
| anti-Nur77 K539Ac | This manuscript | N/A |
| Rabbit Monoclonal anti-Sirt2 | Abcam (UK) | ab211033 |
| Mouse Monoclonal anti-Sirt2 | Abmart (China) | MA8263S |
| Rabbit anti-DYKDDDDK Tag | Cell Signaling Technology (USA) | 14793S |
| Mouse Monoclonal anti-DYKDDDDK Tag | Proteintech | 66008-4-Ig |
| Rabbit anti-MYC Tag | Proteintech | 16286-1-AP |
| Mouse Monoclonal anti-MYC Tag | Proteintech | 60003-2-Ig |
| Rabbit anti-acetylated lysine | Cell Signaling Technology (USA) | 9441S |
| Mouse Monoclonal anti-Ubiquitin | Cell Signaling Technology (USA) | 3936S |
| Rabbit Monoclonal anti-Acetyl-α-Tubulin | Cell Signaling Technology (USA) | 5335S |
| Mouse Monoclonal anti-MuRF1 | SantaCruz (USA) | sc-398608 |
| Mouse Monoclonal anti-MAFbx | SantaCruz (USA) | sc-166806 |
| Rabbit anti-P53 | Proteintech | 10442-1-AP |
| Rabbit anti-P21 | Proteintech | 10355-1-AP |
| Rabbit Monoclonal anti-P16 | Abcam (UK) | ab211542 |
| Rabbit anti-PAX7 | Abcam (UK) | ab187339 |
| Rabbit anti-MYF5 | Thermo Fisher Scientiﬁc (USA) | PA5115608 |
| Mouse Monoclonal anti-MYOD | SantaCruz (USA) | sc-32758 |
| Mouse Monoclonal anti-myogenin | SantaCruz (USA) | sc-52903 |
| Rabbit anti-MYH1 | Abmart (China) | PS04877S |
| Rabbit anti-MYH2 | Affbiotech | DF8414 |
| Mouse Monoclonal anti-MYH3 | SantaCruz (USA) | sc-53091 |
| Mouse Monoclonal anti-Myosin 4 | Thermo Fisher Scientiﬁc (USA) | 14-6503-82 |
| Mouse Monoclonal anti-Ki67 | Abcam (UK) | ab279653 |
| Rabbit Monoclonal anti-TIMP1 | Cell Signaling Technology (USA) | 8946T |
| Rabbit Monoclonal anti-TIMP2 | Cell Signaling Technology (USA) | 5738T |
| Rabbit Monoclonal anti-MMP-2 | Cell Signaling Technology (USA) | 87809T |
| Rabbit Monoclonal anti-MMP-9 | Cell Signaling Technology (USA) | 13667T |
| Rabbit anti-Fibronectin | Proteintech | 15613-1-AP |
| Rabbit anti-α-SMA | Proteintech | 14395-1-AP |
| Rabbit anti-collagen 1 | Proteintech | 14695-1-AP |
| Rabbit Monoclonal anti-Vimentin | Cell Signaling Technology (USA) | 5741S |
| Mouse Monoclonal anti-GAPDH | Proteintech | 60004-1-Ig |
| Mouse Monoclonal anti-HSP90 | SantaCruz(USA) | sc-13119 |
| Rabbit Monoclonal anti-YY1 | Cell Signaling Technology (USA) | 46395S |
| Anti-mouse IgG for IP (HRP) | Abcam (UK) | ab131368 |
| Anti-rabbit IgG | Cell Signaling Technology (USA) | 7074S |
| Anti-mouse IgG | Cell Signaling Technology (USA) | 7076S |
| Anti-Rabbit IgG Alexa Fluor^TM^ 568 | Thermo Fisher Scientiﬁc (USA) | A-11011 |
| Anti-Mouse IgG Alexa Fluor^TM^ 488 | Thermo Fisher Scientiﬁc (USA) | A-11008 |
| Bacterial and virus strains | | |
| Recombinant adenoviruses (carrying Nur77 wild-type, K310Rmutants) | GeneChem (Shanghai, China) | NA |
| Lentiviruses | This manuscript | N/A |
| Chemicals, peptides, and recombinant proteins | | |
| AGK2 | Medchemexpress (USA) | [HY-100578](https://www.medchemexpress.cn/AGK2.html) |
| A-485 | Medchemexpress (USA) | [HY-107455](https://www.medchemexpress.cn/A-485.html) |
| Chloroquine | Medchemexpress (USA) | HY-17589A |
| MG132 | Medchemexpress (USA) | [HY-13259](https://www.medchemexpress.cn/MG-132.html) |
| [Cycloheximide](https://www.medchemexpress.cn/Cycloheximide.html) | Medchemexpress (USA) | [HY-12320](https://www.medchemexpress.cn/Cycloheximide.html) |
| Cytosporone B | Medchemexpress (USA) | HY-N2148 |
| hFGF basic/FGF2 | Medchemexpress (USA) | HY-12320 |
| Critical commercial assays and reagents | | |
| PrimeScript RT reagent Kit with gDNA Eraser | TAKARA (Japan) | RRO47A |
| TNT® T7 Quick Coupled Transcription/Translation System | Promega (USA) | L1170 |
| GLUTATHIONE SEPHAROSE 4B | Cytiva (USA) | 17075601 |
| SimpleChIP®Enzymatic Chromatin IP Kit | Cell Signaling Technology (USA) | 9002S |
| QuantiNova SYBR Green PCR Kit | Qiagen (Germany) | 208054 |
| Dual-Luciferase® Reporter Assay System | Promega (USA) | E1910 |
| Skeletal Muscle Dissociation Kit, m & r | Miltenyi (Germany) | 130-098-305 |
| MACS BSA Stock Solution | Miltenyi (Germany) | 130-091-376 |
| MACS Tissue Storage Solution | Miltenyi (Germany) | 130-100-008 |
| Satellite Cell Isolation Kit, mouse | Miltenyi (Germany) | 130-104-268 |
| Corning® Matrigel® Basement Membrane Matrix | Corning (USA) | 354234 |
| Experimental models: Cell lines | | |
| HEK-293T(RRID:CVCL_0063) | HaiXing (Shanghai, China) | TCH-C101 |
| C2C12(RRID: CVCL_0188) | HaiXing (Shanghai, China) | TCM-C720 |
| Satellite cell | This manuscript | N/A |
| Experimental models: Organisms/strains | | |
| Mouse:Nur77^−/−^ | GemPharmatech (Jiangsu,China) | N/A |
| Mouse:Nur77^flox/flox^ | Cyagen (Jiangsu, China) | N/A |
| Mouse:Myf5-Cre | Cyagen (Jiangsu, China) | C001451 |
| Mouse:tdTomato | GemPharmatech (Jiangsu, China) | T002249 |
| Recombinant DNA | | |
| h-Flag-Nur77(FL) | Miaoling (Wuhai, China) | N/A |
| h-Flag-Nur77(1-268) | This manuscript | N/A |
| h-Flag-Nur77(268-598) | This manuscript | N/A |
| h-Flag-Nur77(del268-339) | This manuscript | N/A |
| h-Flag-Nur77(360-598) | This manuscript | N/A |
| h-Flag-Nur77(K307R) | Miaoling (Wuhai, China) | N/A |
| h-Flag-Nur77(K536R) | Miaoling (Wuhai, China) | N/A |
| h-Flag-Nur77(K307/536R) | Miaoling (Wuhai, China) | N/A |
| m-Flag-Nur77(K310R) | Miaoling (Wuhai, China) | N/A |
| m-Flag-Nur77(K539R) | Miaoling (Wuhai, China) | N/A |
| m-Flag-Nur77(K310/539R) | Miaoling (Wuhai, China) | N/A |
| h-Flag-Nur77(K307Q) | Miaoling (Wuhai, China) | N/A |
| m-Flag-Nur77(K310Q) | Miaoling (Wuhai, China) | N/A |
| m-Flag-Nur77(FL) | This manuscript | N/A |
| h-Myc-Nur77 | This manuscript | N/A |
| h-GST-Nur77 | Miaoling (Wuhai, China) | N/A |
| h-GST-Nur77(1-268) | This manuscript | N/A |
| h-GST-Nur77(268-598) | This manuscript | N/A |
| h-GST-Nur77(del268-339) | This manuscript | N/A |
| h-GST-Nur77(360-598) | This manuscript | N/A |
| h-Flag-Sirt2-WT | Laboratory of Liu Cao | N/A |
| h-Flag-Sirt2-MUT(H187Y) | Laboratory of Liu Cao | N/A |
| h-Myc-Sirt2 | Laboratory of Liu Cao | N/A |
| m-Flag-Sirt2 | Miaoling (Wuhai, China) | N/A |
| h-Flag-P300 | Laboratory of Liu Cao | N/A |
| h-Flag-CBP | Laboratory of Liu Cao | N/A |
| h-Flag-GCN5 | Laboratory of Liu Cao | N/A |
| h-Flag-PCAF | Laboratory of Liu Cao | N/A |
|  |  |  |

Table S4. Primers used

|  | Gene | Primer |
| --- | --- | --- |
| RT-qPCR | h-Nur77-Fw | TGACTACTATGGCAGCCCCT |
| RT-qPCR | h-Nur77-Rv | GGCTGCTTGGGTTTTGAAGG |
| RT-qPCR | m-Nur77-Fw | GTGTCAGCACTATGGGGTCC |
| RT-qPCR | m-Nur77-Rv | TCTTGTCCACAGGGCAATCC |
| RT-qPCR | m-Sirt2-Fw | TGGCCTCTATGCAAACCTGG |
| RT-qPCR | m-Sirt2-Rv | TCCGTCTGGCCTGTCTTTTC |
| RT-qPCR | h-Sirt2-Fw | CATCCACCGGCCTCTATGAC |
| RT-qPCR | h-Sirt2-Rv | CACCCAGCCAGGCCAC |
| RT-qPCR | m-Pax7 Fw | GTGCCCTCAGTGAGTTCGAT |
| RT-qPCR | m-Pax7 Rv | CTGATGCATGGTTGATGGCG |
| RT-qPCR | m-Myf5 Fw | TCTGGTCCCGAAAGAACAGC |
| RT-qPCR | m-Myf5 Rv | GCTCGGATGGCTCTGTAGAC |
| RT-qPCR | h-Myf5 Fw | GCTGCCAGTTCTCACCTTCT |
| RT-qPCR | h-Myf5 Rv | CCTGCAGGCTCTCGATGTAG |
| RT-qPCR | m-P300-Fw | ACAGCTGATCCAGAGAAGCG |
| RT-qPCR | m-P300-Rv | TGTCCAGATGGCTGGCTTTG |
| RT-qPCR | h-GAPDH-Fw | CAAATTCCATGGCACCGTCA |
| RT-qPCR | h-GAPDH-Rv | GATGGCATGGACTGTGGTCA |
| RT-qPCR | m-GAPDH-Fw | AACAGCAACTCCCACTCTTC |
| RT-qPCR | m-GAPDH-Rv | CCTGTTGCTGTAGCCGTATT |
| CHIP | Myf5-S1-1-Fw | TGTCACTCTCCCACCTCTGG |
| CHIP | Myf5-S1-1-Rv | CCTTTTCAGGGTGCGATCTCT |
| CHIP | Myf5-S1-2-Fw | CTCTCCCACCTCTGGTTTTCAA |
| CHIP | Myf5-S1-2-Rv | CAGGGGCCAGAGAAGTAGCAA |
| CHIP | Myf5-S1-3-Fw | GAGACAGCAATGGTGTGGCTA |
| CHIP | Myf5-S1-3-Rv | CAACCTTTTCAGGGTGCGAT |
| CHIP | Myf5-S2-1-Fw | TTTGGAAAGGCAAACCCTGT |
| CHIP | Myf5-S2-1-Rv | TCCAGCAATTGGATTGAATAACGG |
| CHIP | Myf5-S2-2-Fw | GATTTGGAAAGGCAAACCCTGT |
| CHIP | Myf5-S2-2-Rv | CCAGCAATTGGATTGAATAACGGT |
| CHIP | Myf5-S2-3-Fw | ATTTGGAAAGGCAAACCCTGT |
| CHIP | Myf5-S2-3-Rv | CCAGCAATTGGATTGAATAACGG |

Abbreviations: Fw, Forward; Rv, Reverse.
