# Supplementary figures and images for: The Sirt2–Nur77 axis regulates muscle stem cell quiescence and senescence via epigenetic–metabolic synergy
Source: Cell Death Dis. 2026 Mar 28;17(1):429. doi: 10.1038/s41419-026-08645-w (PMC13153389; doi:10.1038/s41419-026-08645-w)

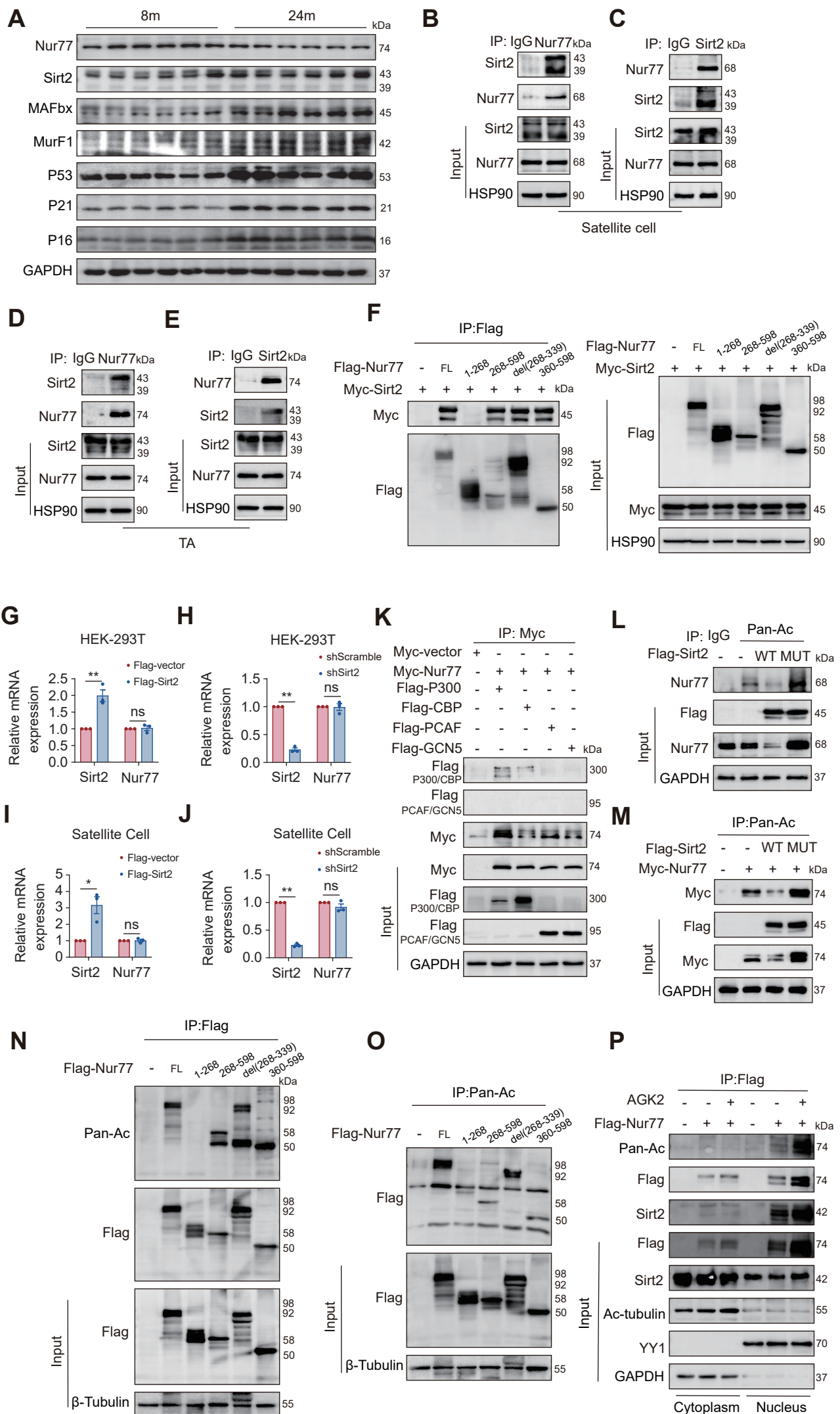

Supplement: Supplementary file 2 — Figure S1 [file 41419_2026_8645_MOESM2_ESM.pdf]

**A**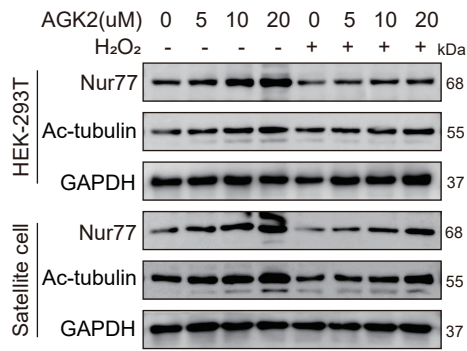**B**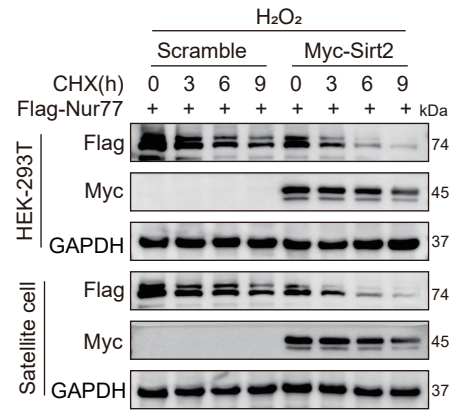**C**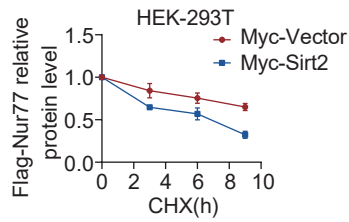**D**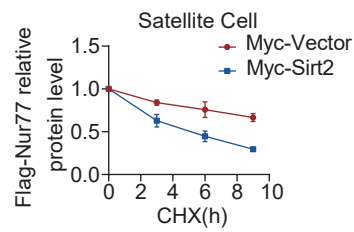**E**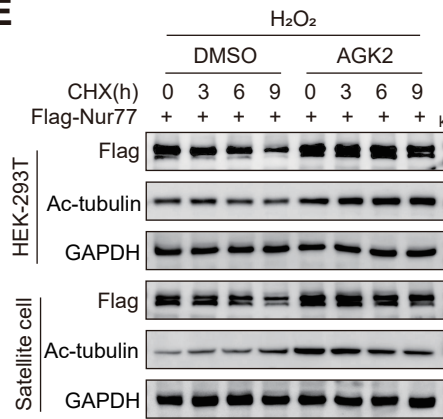**F**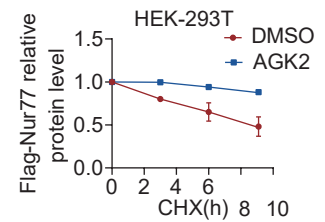**G**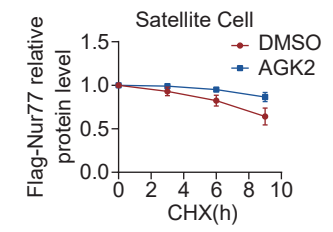**H**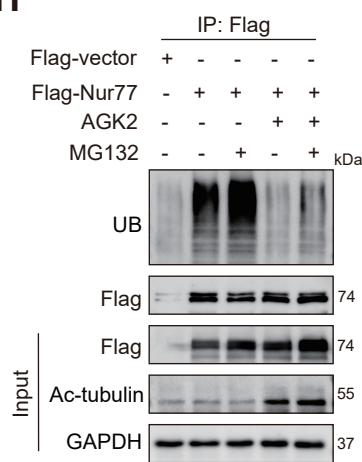**I**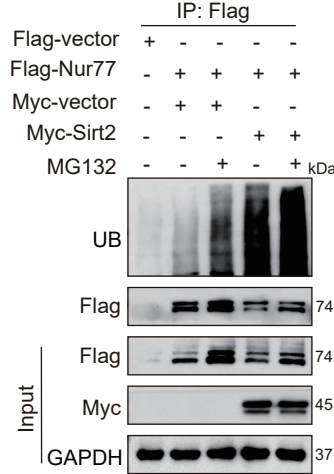**J**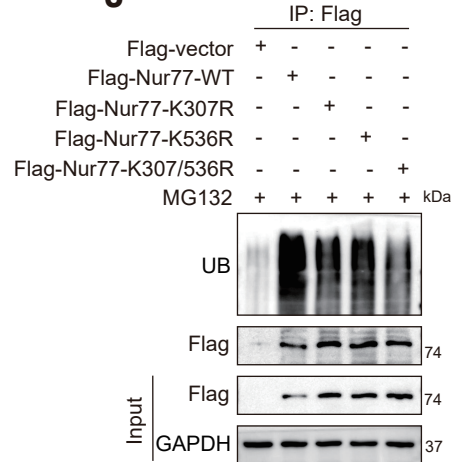**K**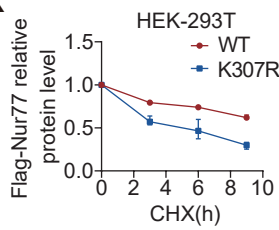**L**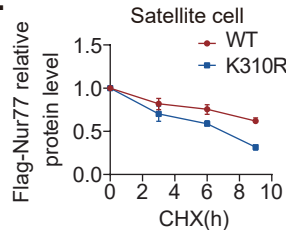**M**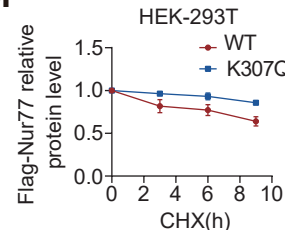**N**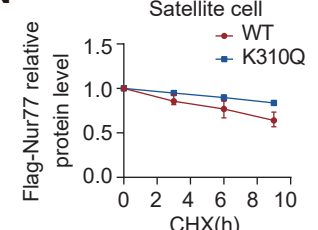

Supplement: Supplementary file 3 — Figure S2 [file 41419_2026_8645_MOESM3_ESM.pdf]

**A**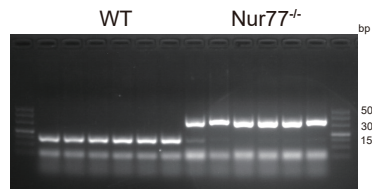**B**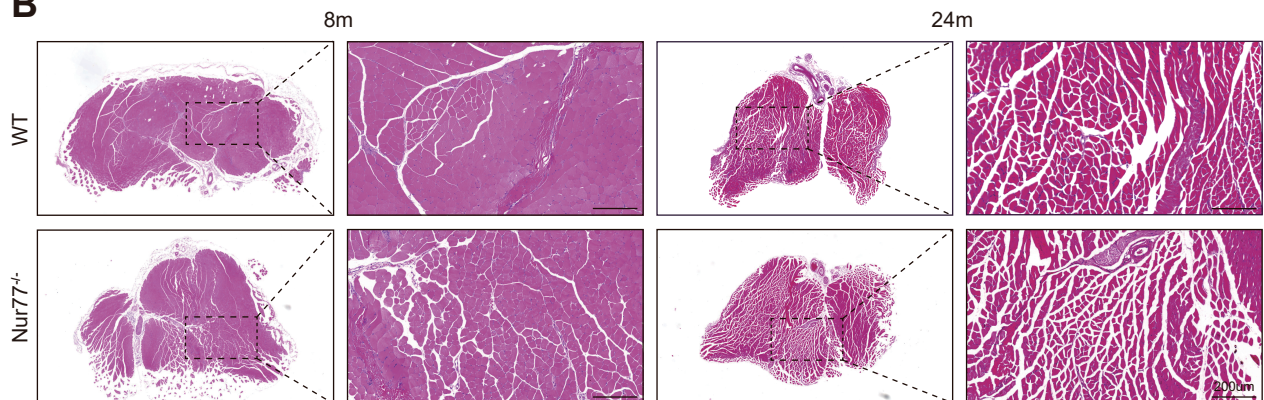**C**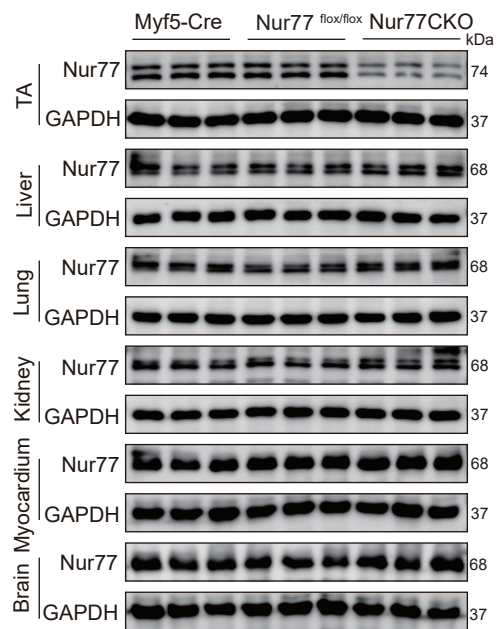**D**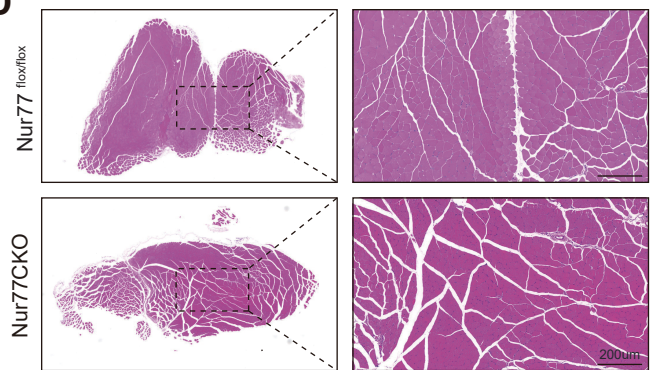**E**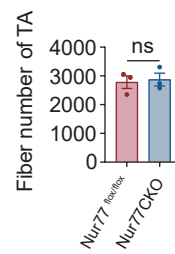**F**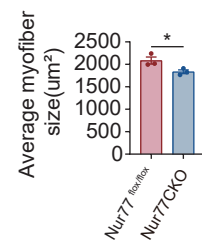

Supplement: Supplementary file 4 — Figure S3 [file 41419_2026_8645_MOESM4_ESM.pdf]

**A**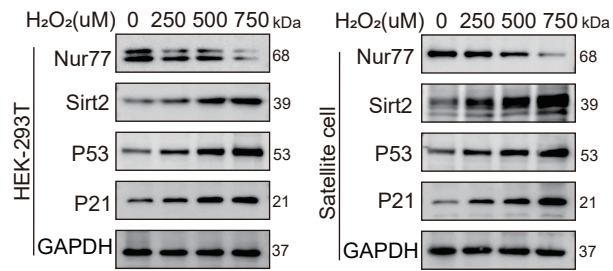**B**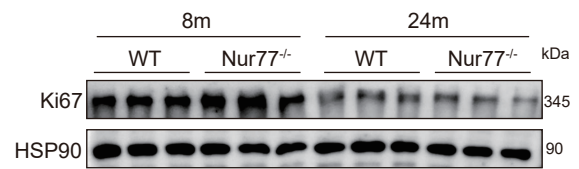**C**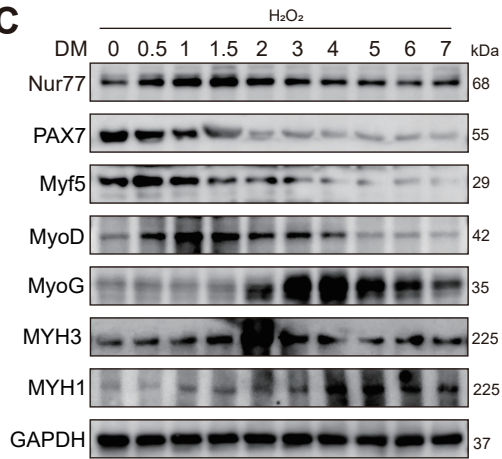**D**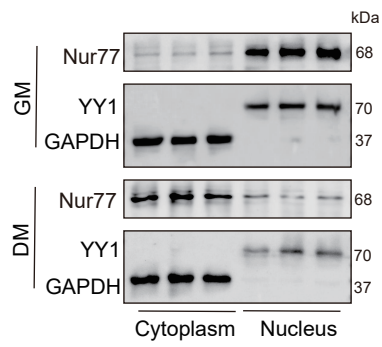**E**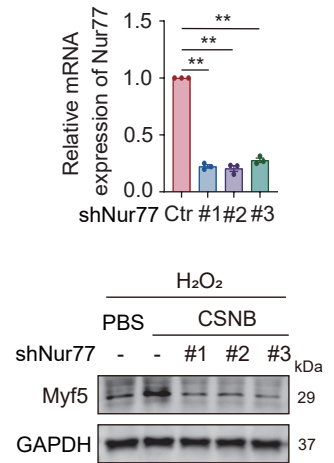

Supplement: Supplementary file 5 — Figure S4 [file 41419_2026_8645_MOESM5_ESM.pdf]

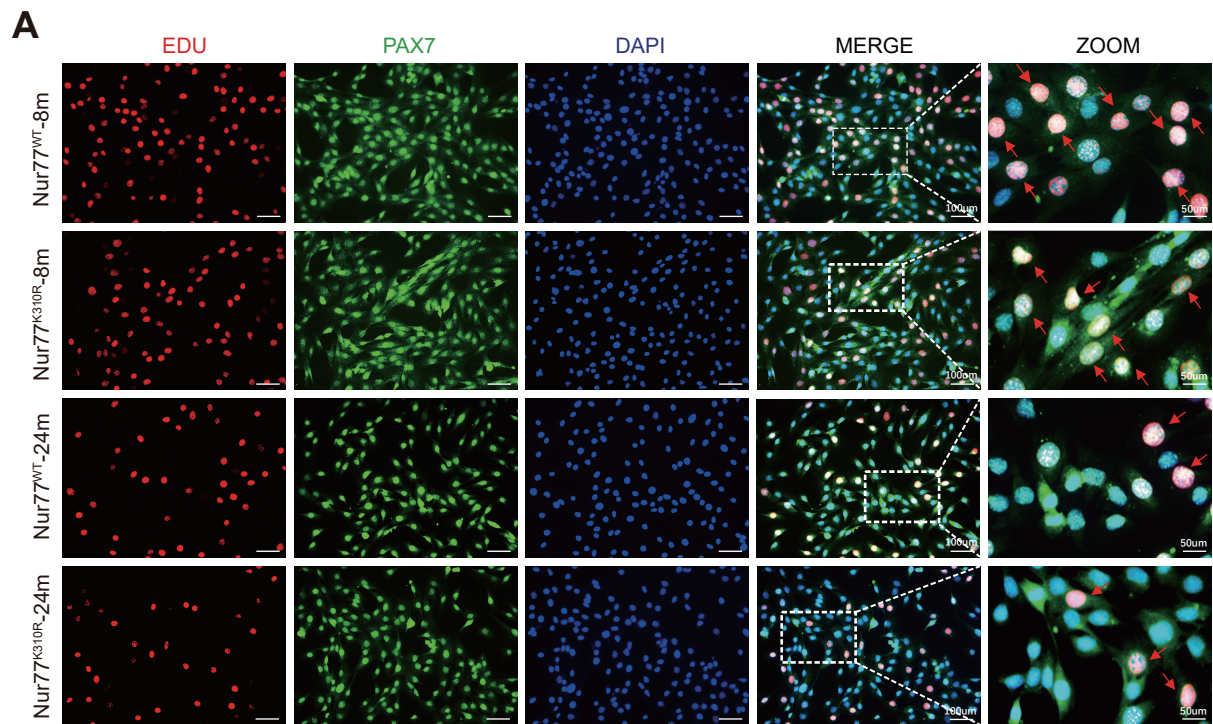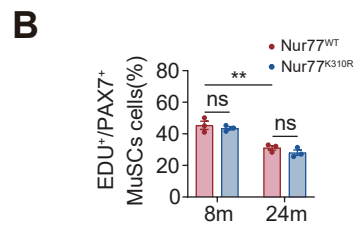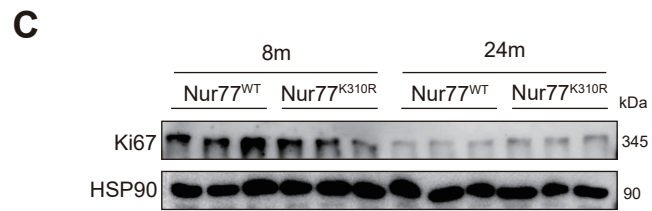

Supplement: Supplementary file 6 — Figure S5 [file 41419_2026_8645_MOESM6_ESM.pdf]
