# Supplementary material for: The Sirt2–Nur77 axis regulates muscle stem cell quiescence and senescence via epigenetic–metabolic synergy
Source: Cell Death Dis. 2026 Mar 28;17(1):429. doi: 10.1038/s41419-026-08645-w (PMC13153389; doi:10.1038/s41419-026-08645-w)
Supplement: Supplementary file 7 — western blots [file 41419_2026_8645_MOESM7_ESM.pdf]

Figure 1A

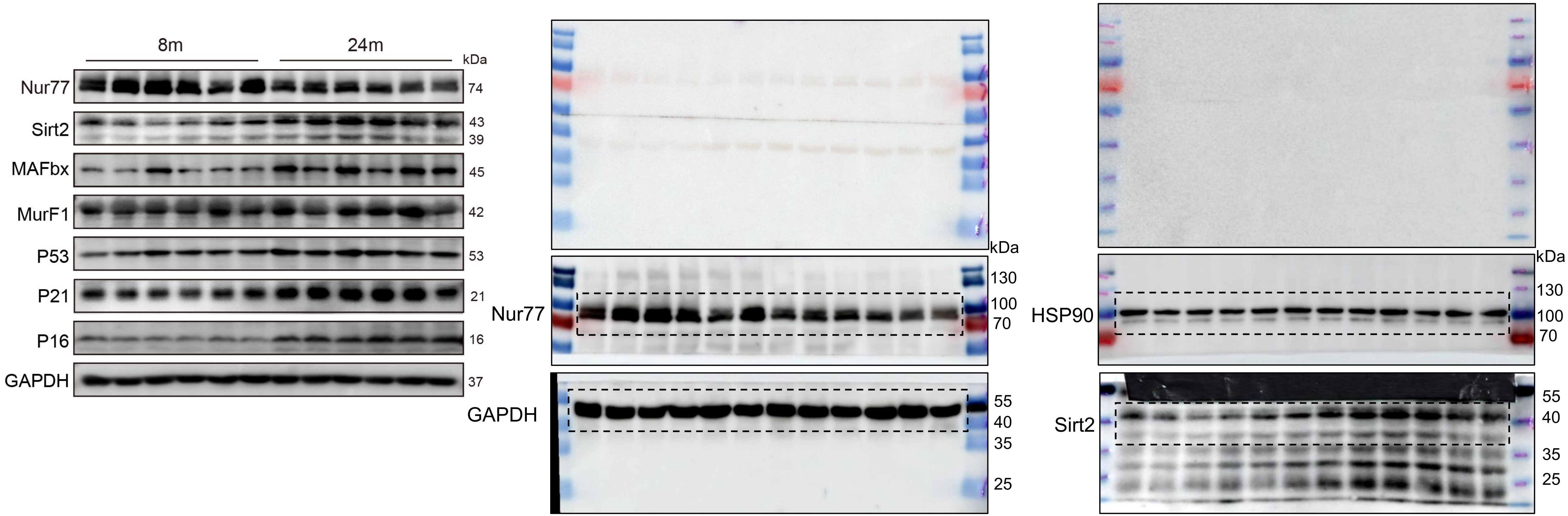
8m
24m
kDa
Nur77
74
Sirt2
43
39
MAFbx
45
MurF1
42
P53
53
P21
21
P16
16
GAPDH
37
kDa
130
100
70
Nur77
GAPDH
55
40
35
25
kDa
130
100
70
HSP90
Sirt2
55
40
35
25

Figure 1A

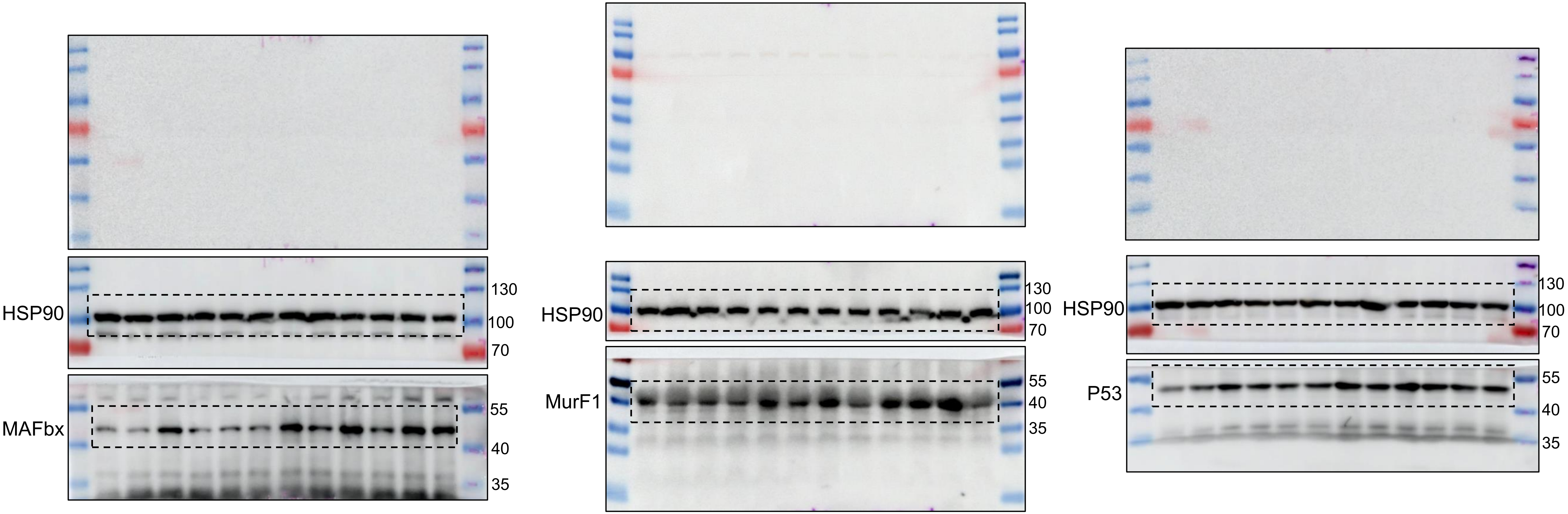
HSP90
130
100
70
MAFbx
55
40
35
HSP90
130
100
70
MurF1
55
40
35
HSP90
130
100
70
P53
55
40
35

Figure 1A

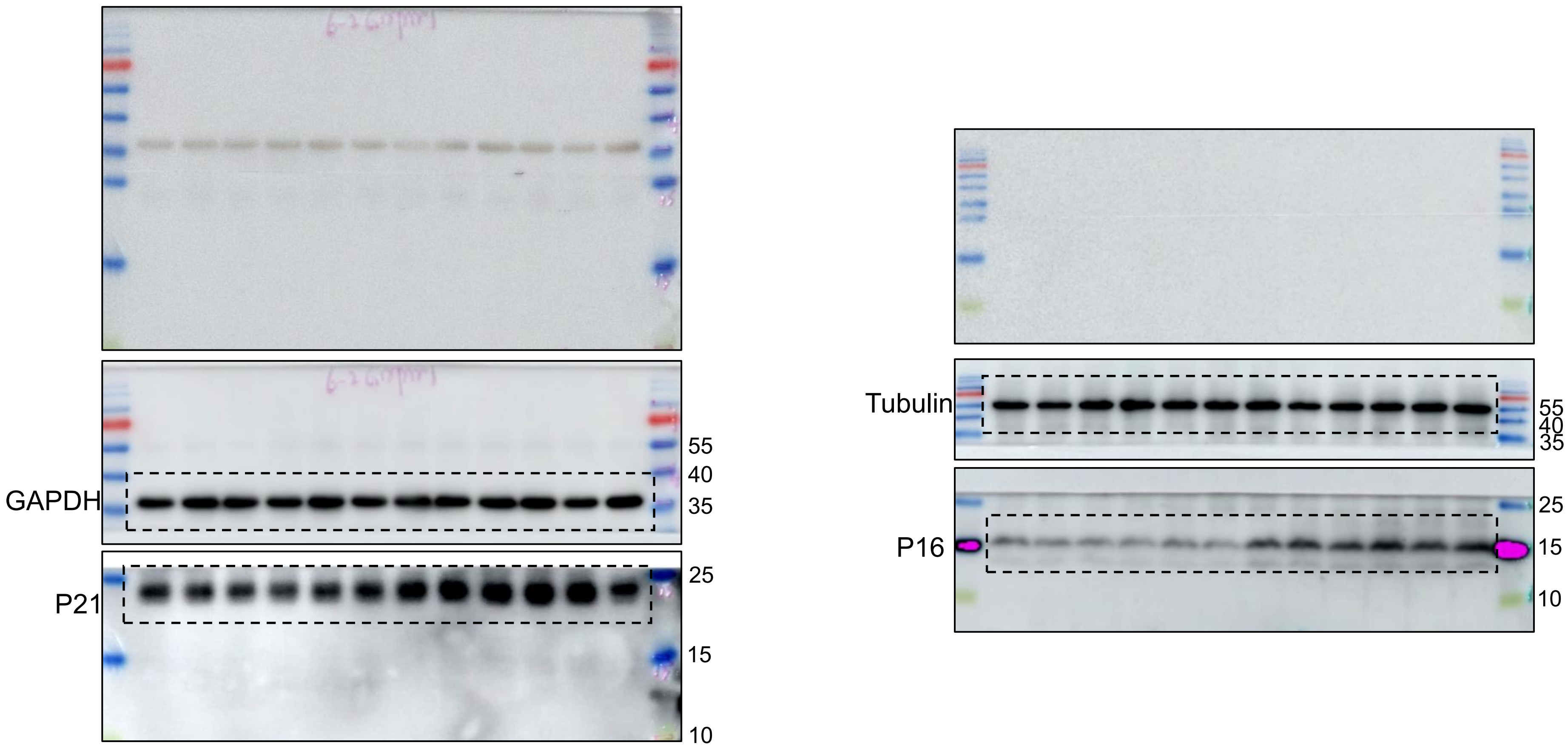

Figure 1B

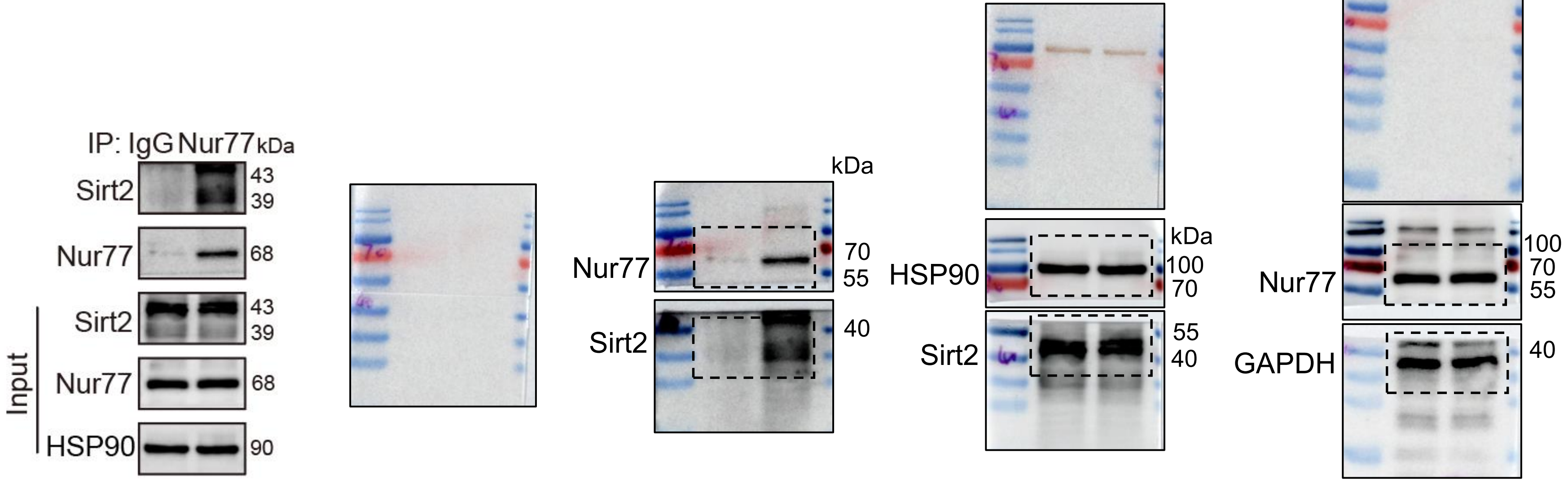

Figure 1D

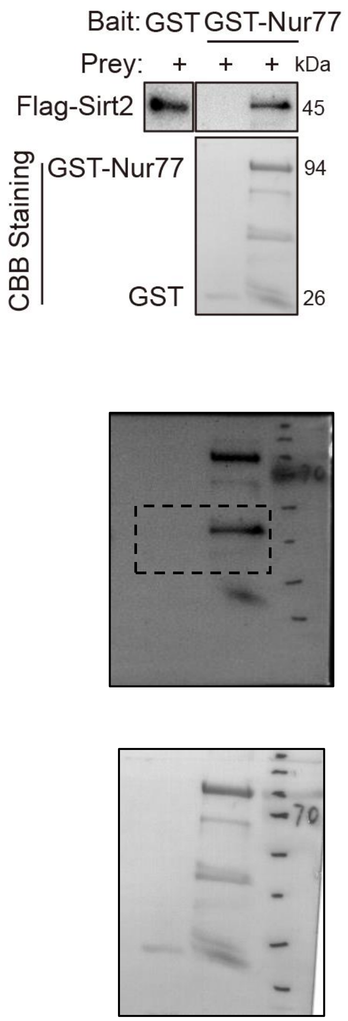

Figure 1C

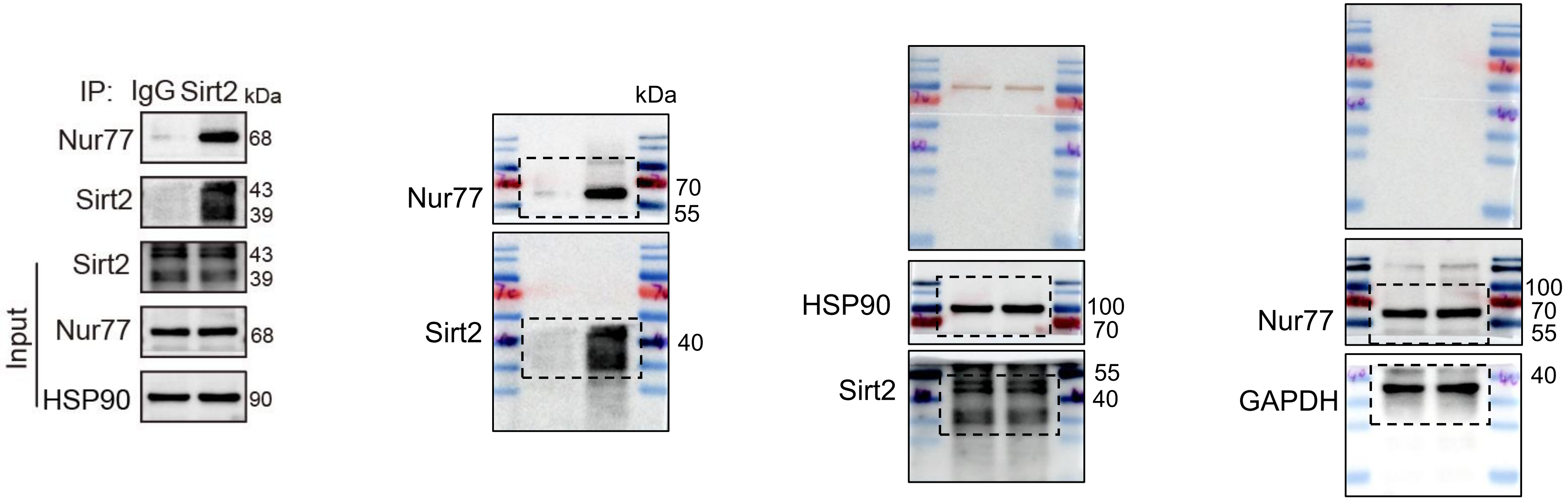

Figure 1E

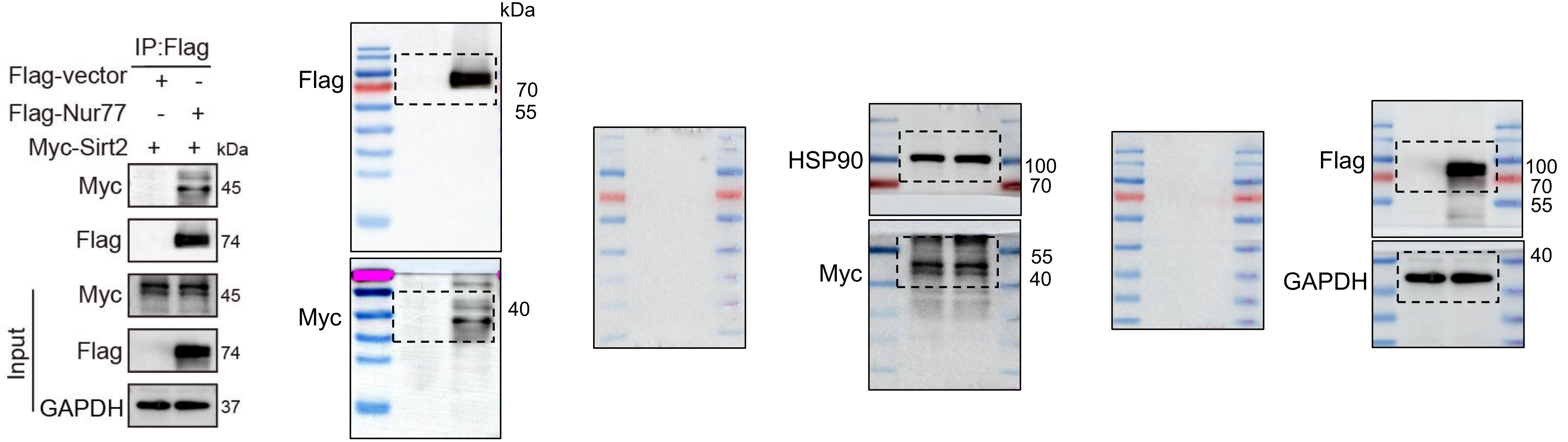

Figure 1F

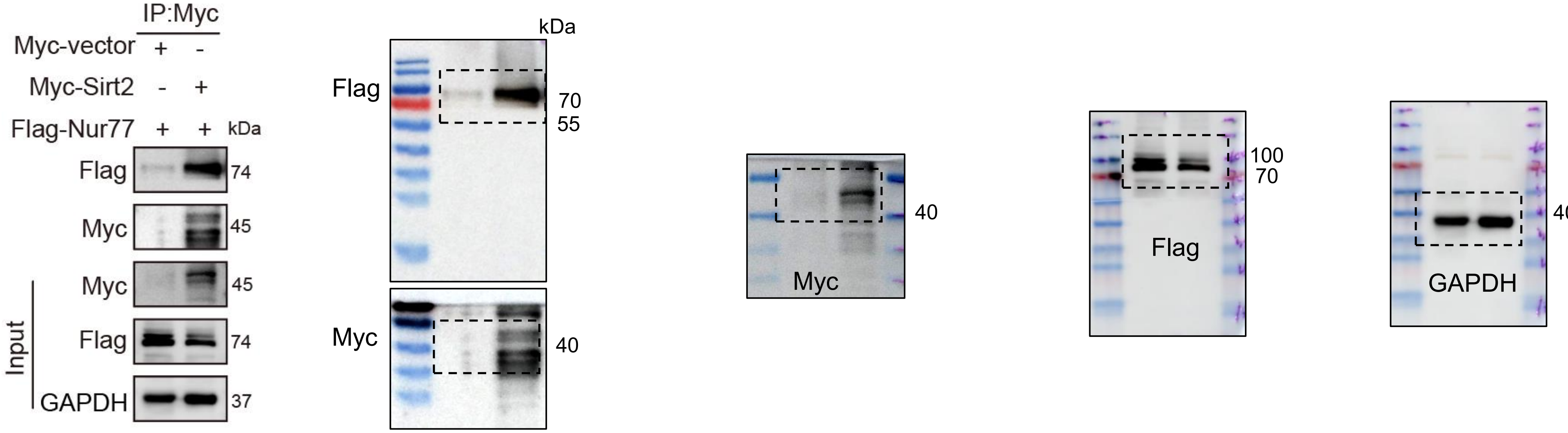

Figure 1K

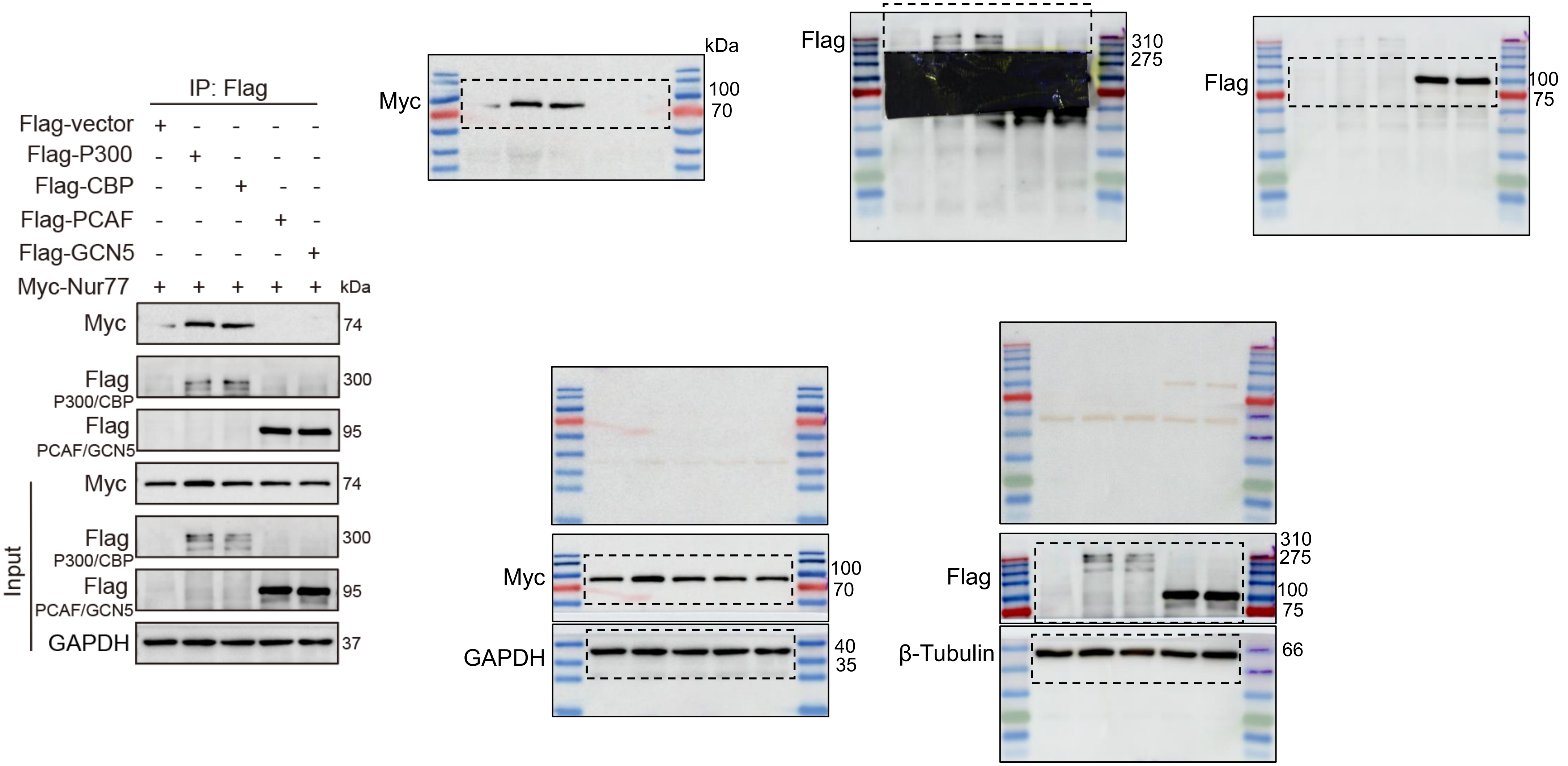
IP: Flag
Flag-vector + - - - -
Flag-P300 - + - - -
Flag-CBP - - + - -
Flag-PCAF - - - + -
Flag-GCN5 - - - - +
Myc-Nur77 + + + + +
kDa
Myc 74
Flag P300/CBP 300
Flag PCAF/GCN5 95
Input
Myc 74
Flag P300/CBP 300
Flag PCAF/GCN5 95
GAPDH 37
Myc
kDa
100
70
Flag
310
275
Flag
100
75
Myc
100
70
GAPDH
40
35
Flag
310
275
100
75
β-Tubulin
66

Figure 1L

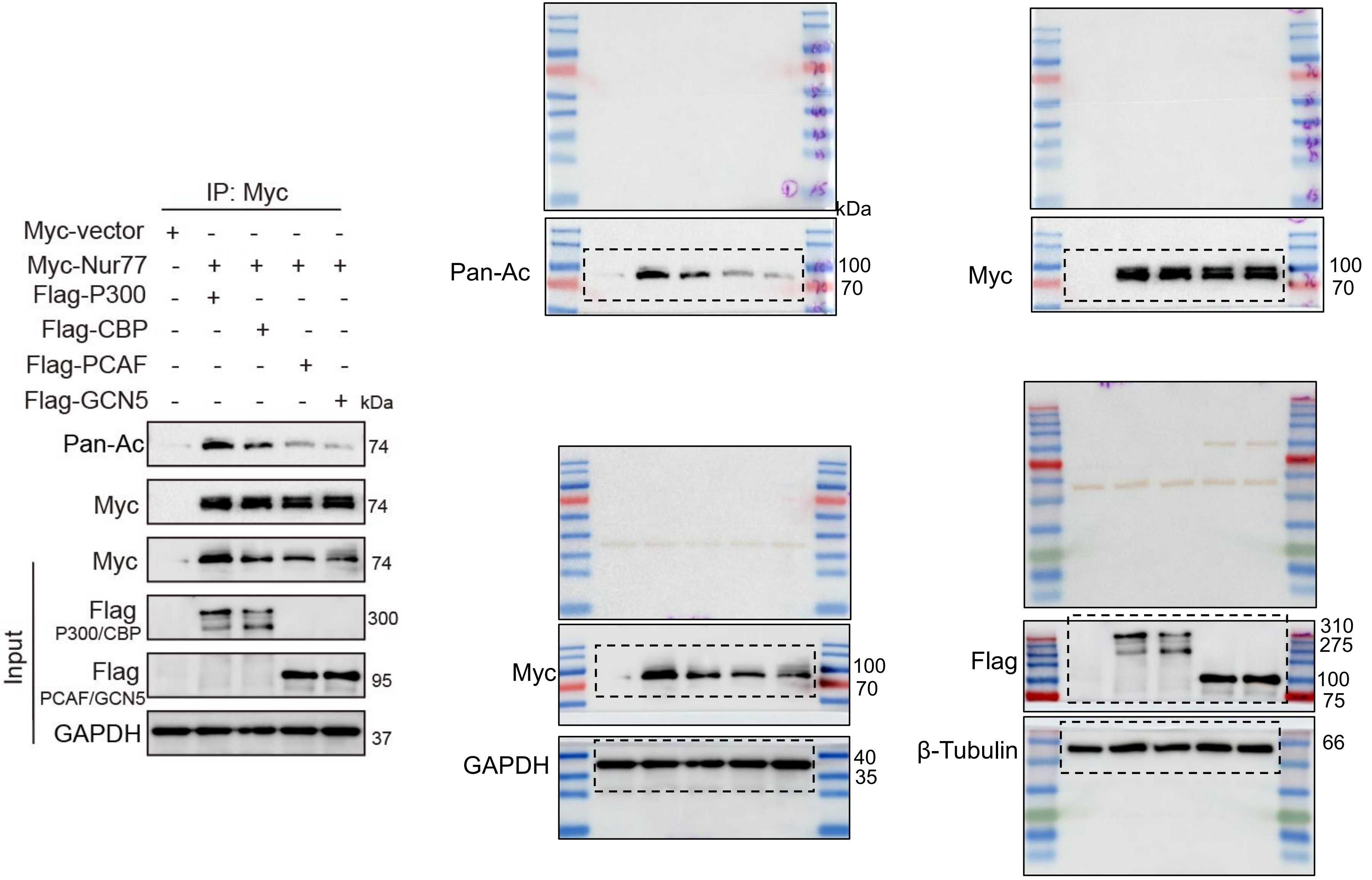

IP: Myc
Myc-vector + - - - -
Myc-Nur77 - + + + +
Flag-P300 - + - - -
Flag-CBP - - + - -
Flag-PCAF - - - + -
Flag-GCN5 - - - - +
kDa
Pan-Ac 74
Myc 74
Myc 74
Flag P300/CBP 300
Flag PCAF/GCN5 95
GAPDH 37
Input
kDa
Pan-Ac
100
70
Myc
100
70
Myc
100
70
GAPDH
40
35
Flag
310
275
100
75
β-Tubulin
66

Figure 1M

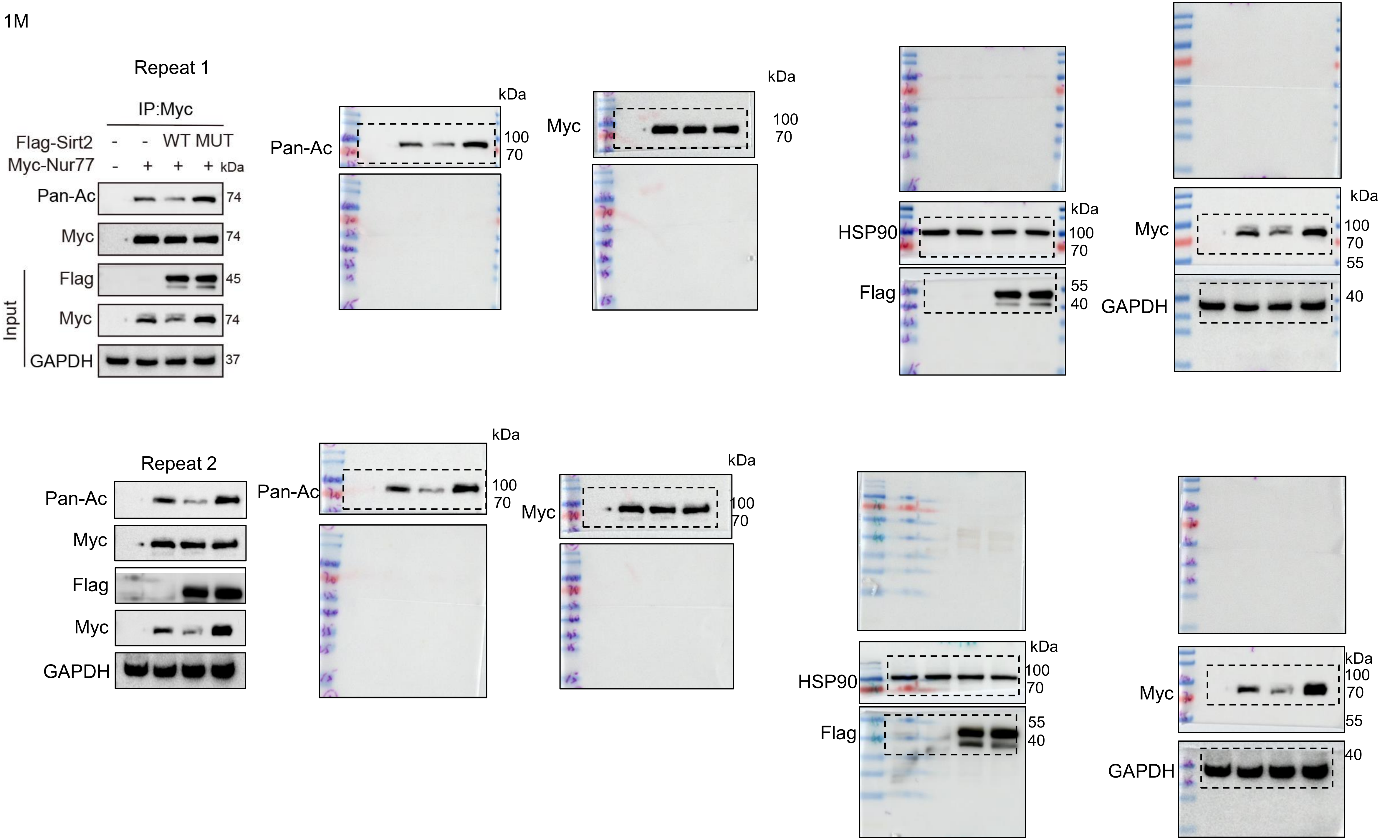
Repeat 1
IP:Myc
Flag-Sirt2 - - WT MUT
Myc-Nur77 - + + + kDa
Pan-Ac 74
Myc 74
Flag 45
Myc 74
GAPDH 37
Input
kDa
Pan-Ac
100
70
Myc
kDa
100
70
kDa
HSP90
100
70
Flag
55
40
kDa
Myc
100
70
55
GAPDH
40
Repeat 2
Pan-Ac
Myc
Flag
Myc
GAPDH
kDa
Pan-Ac
100
70
kDa
Myc
100
70
kDa
HSP90
100
70
Flag
55
40
kDa
Myc
100
70
55
GAPDH
40

Figure 1M

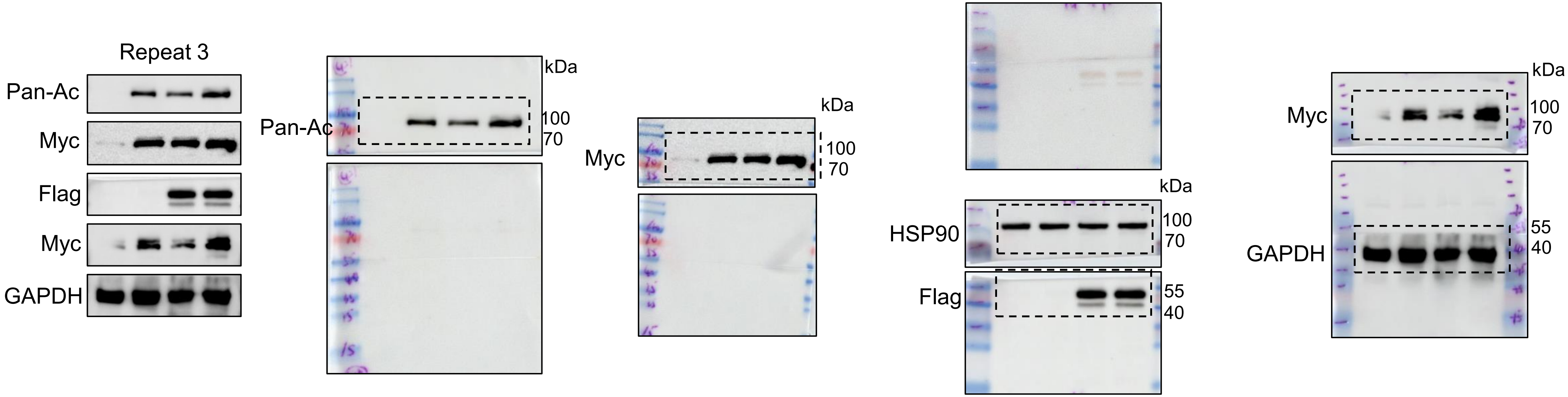

Repeat 3
Pan-Ac
Myc
Flag
Myc
GAPDH
kDa
Pan-Ac
100
70
kDa
Myc
100
70
kDa
HSP90
100
70
Flag
55
40
kDa
Myc
100
70
55
GAPDH
40

Figure 1N

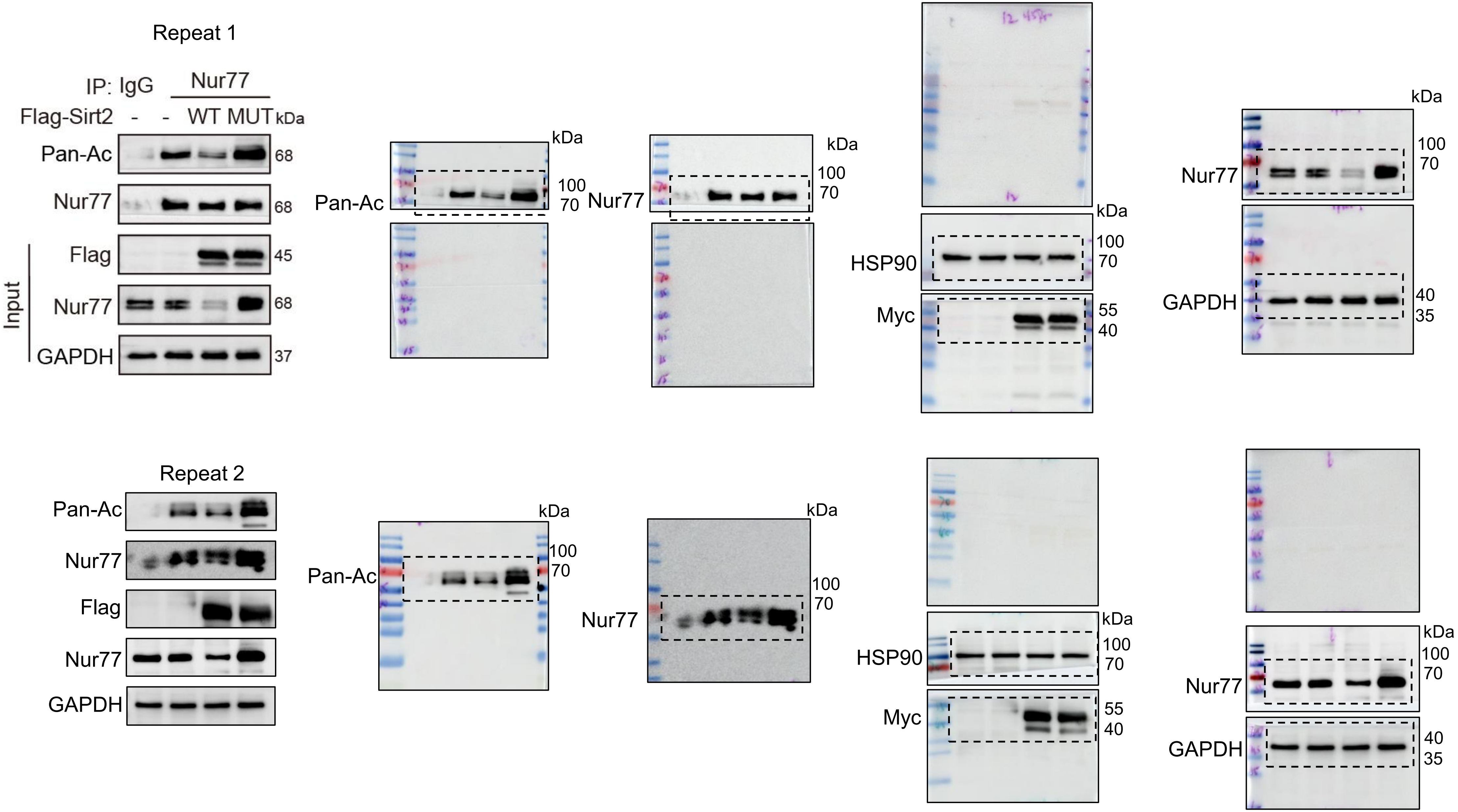
Repeat 1
IP: IgG
Nur77
Flag-Sirt2 - - WT MUT
kDa
Pan-Ac 68
Nur77 68
Input
Flag 45
Nur77 68
GAPDH 37
Pan-Ac
kDa
100
70
Nur77
kDa
100
70
HSP90
kDa
100
70
Myc
55
40
Nur77
kDa
100
70
GAPDH
40
35
Repeat 2
Pan-Ac
Nur77
Flag
Nur77
GAPDH
Pan-Ac
kDa
100
70
Nur77
kDa
100
70
HSP90
kDa
100
70
Myc
55
40
Nur77
kDa
100
70
GAPDH
40
35

Figure 1N

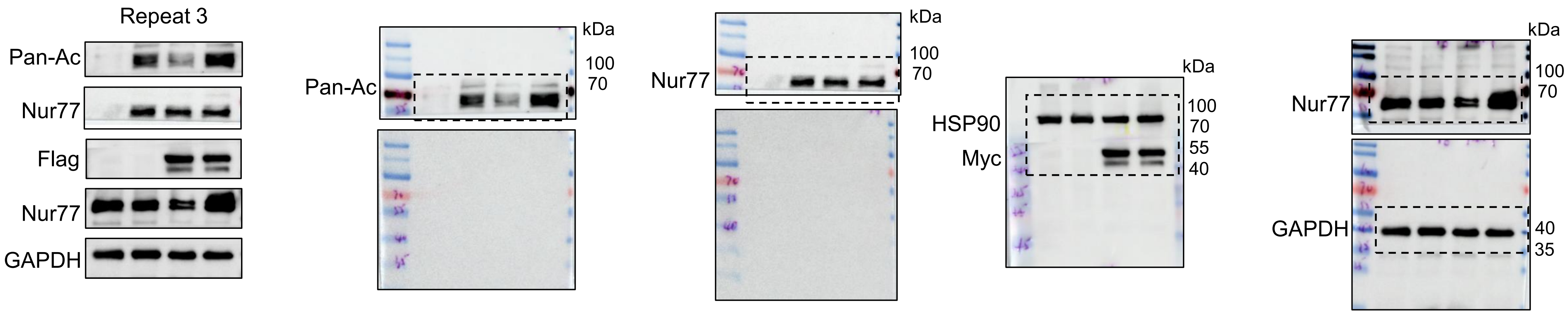

Repeat 3
Pan-Ac
Nur77
Flag
Nur77
GAPDH
Pan-Ac
kDa
100
70
Nur77
kDa
100
70
HSP90
Myc
kDa
100
70
55
40
Nur77
kDa
100
70
GAPDH
40
35

Figure 1Q

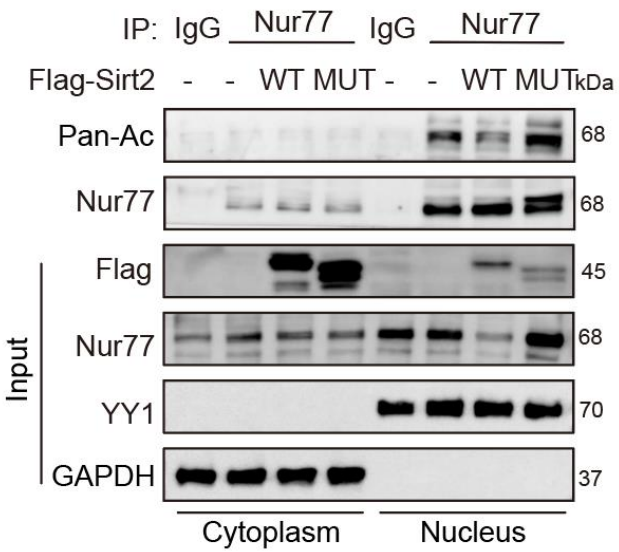

IP: IgG Nur77 IgG Nur77
Flag-Sirt2 - - WT MUT - - WT MUT kDa
Pan-Ac 68
Nur77 68
Flag 45
Nur77 68
YY1 70
GAPDH 37
Input
Cytoplasm
Nucleus

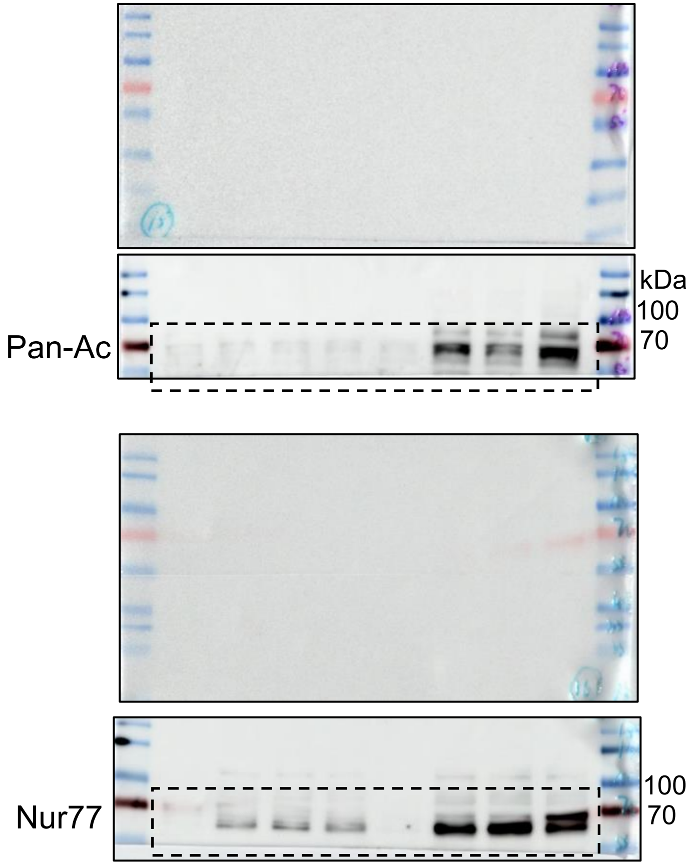

Pan-Ac
kDa
100
70
Nur77
100
70

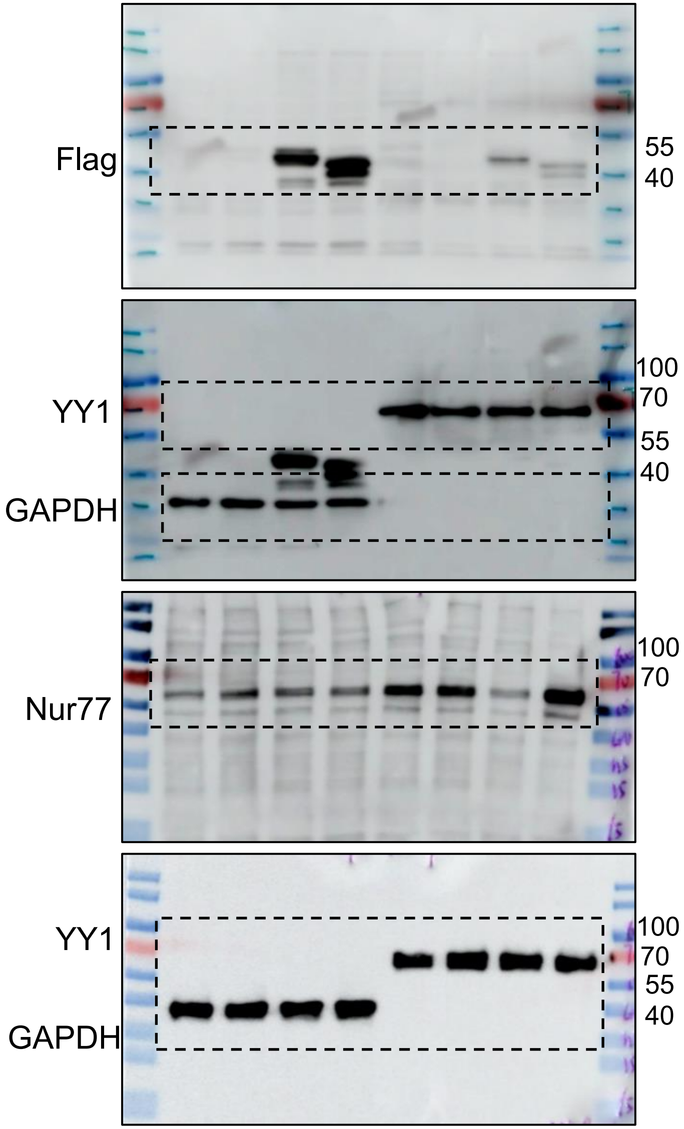

Flag
55
40
YY1
GAPDH
100
70
55
40
Nur77
100
70
YY1
GAPDH
100
70
55
40

Figure 2A

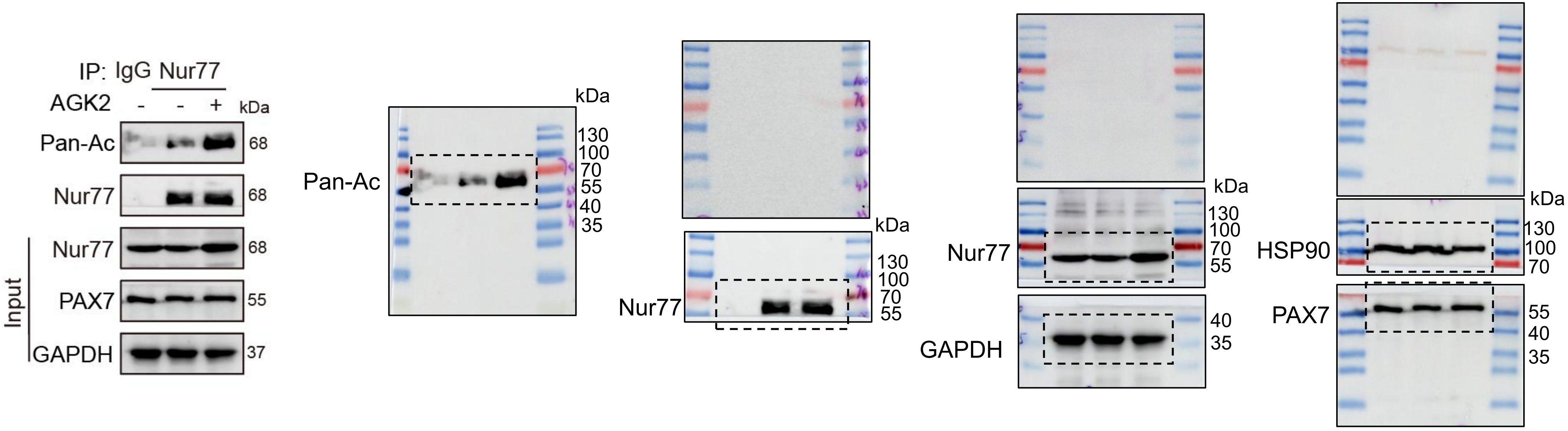

Figure 2B

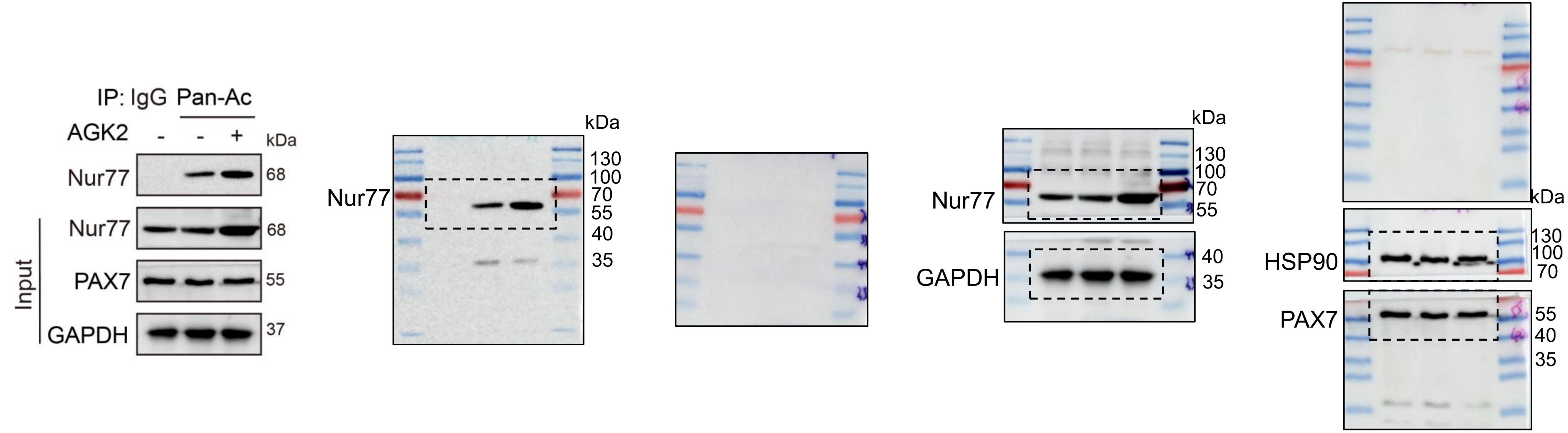

Figure 2E

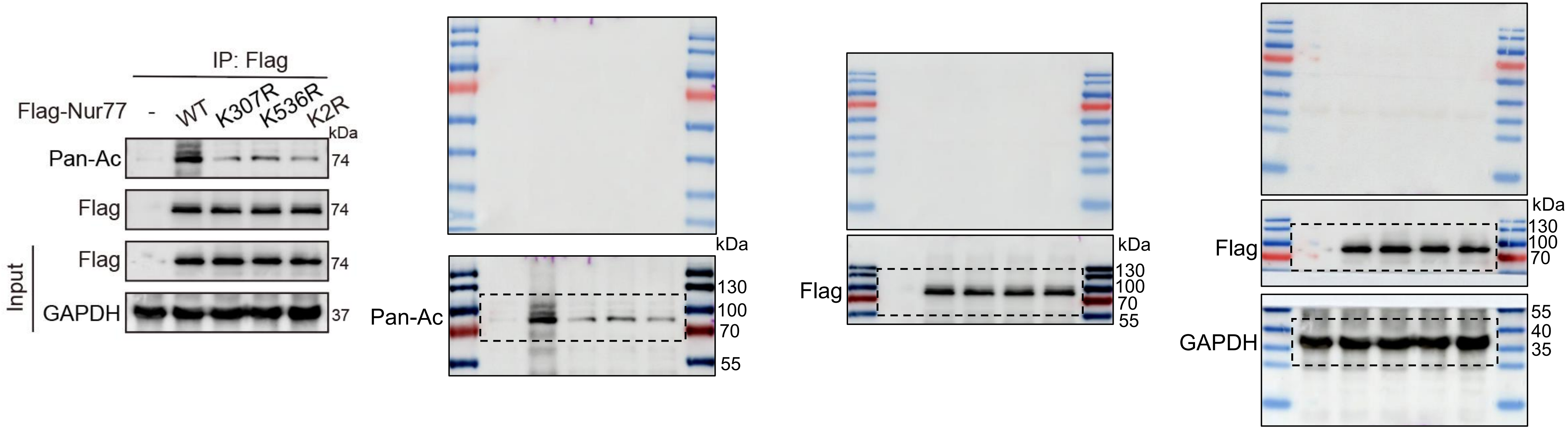

Figure 2F

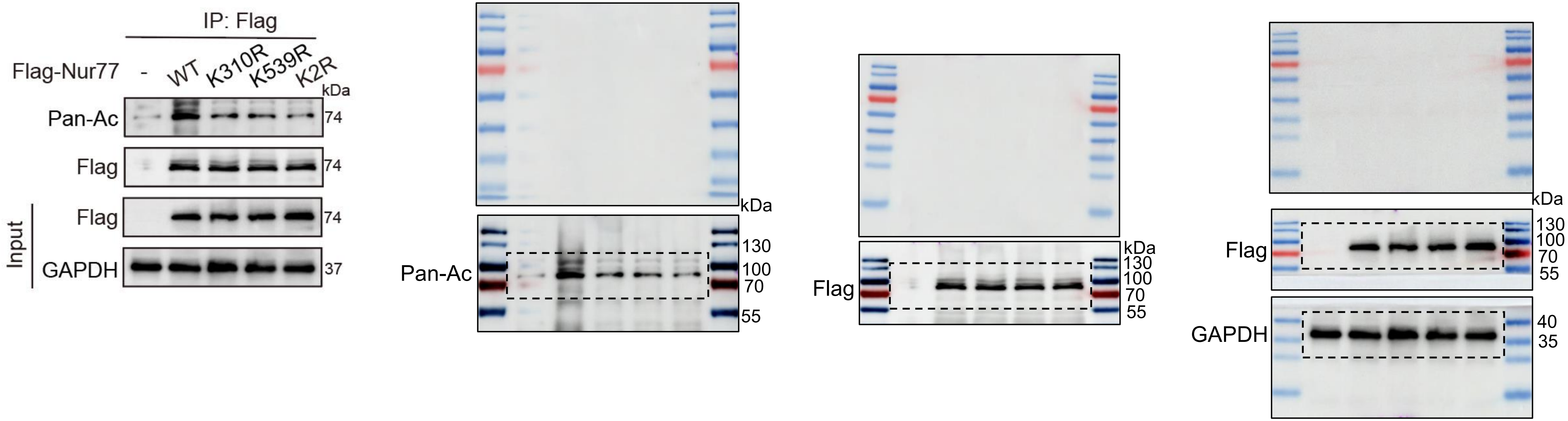

Figure 2I

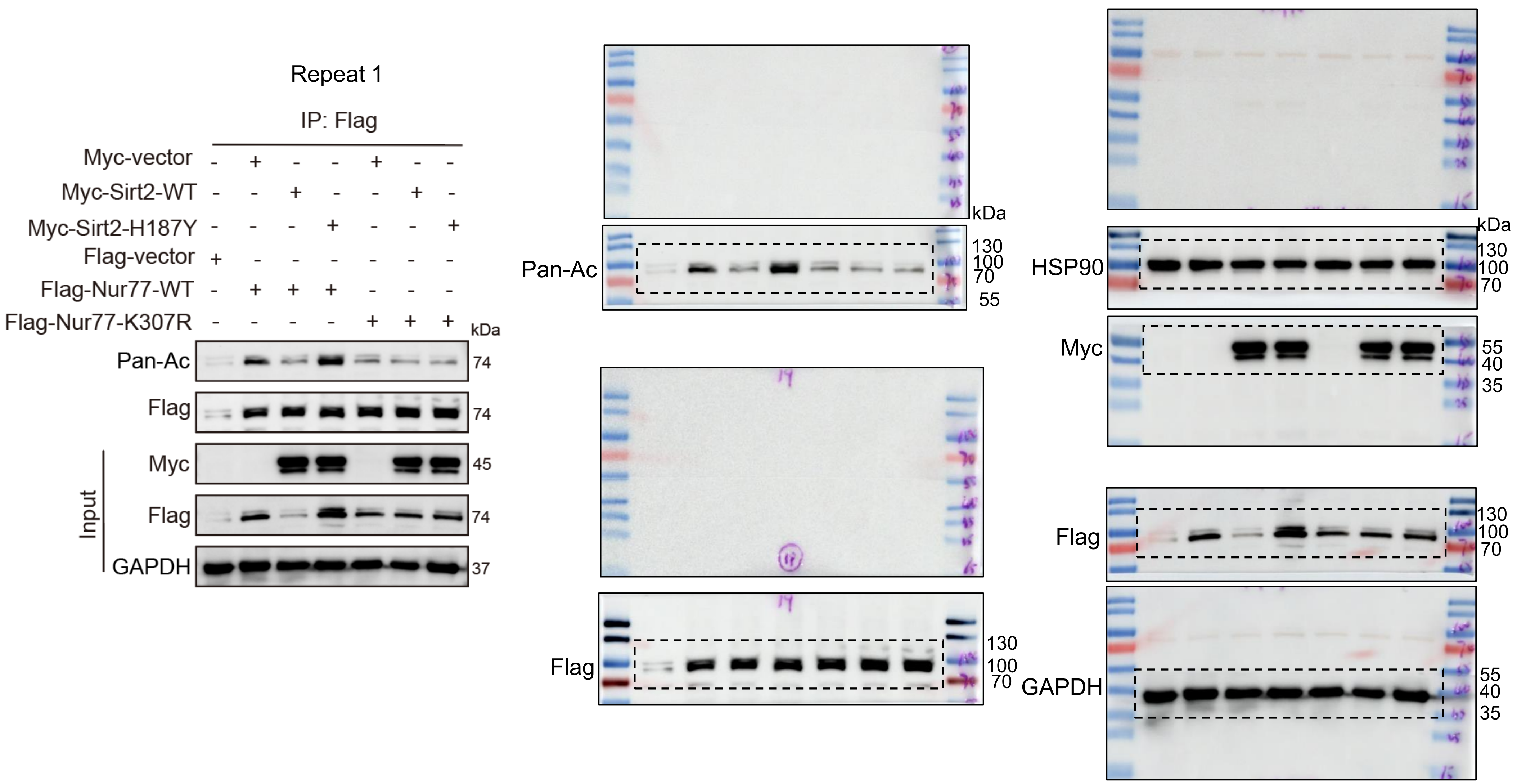
Repeat 1
IP: Flag
Myc-vector
Myc-Sirt2-WT
Myc-Sirt2-H187Y
Flag-vector
Flag-Nur77-WT
Flag-Nur77-K307R
kDa
Pan-Ac
74
Flag
74
Input
Myc
45
Flag
74
GAPDH
37
Pan-Ac
kDa
130
100
70
55
Flag
130
100
70
HSP90
kDa
130
100
70
Myc
55
40
35
Flag
130
100
70
GAPDH
55
40
35

Figure 2I

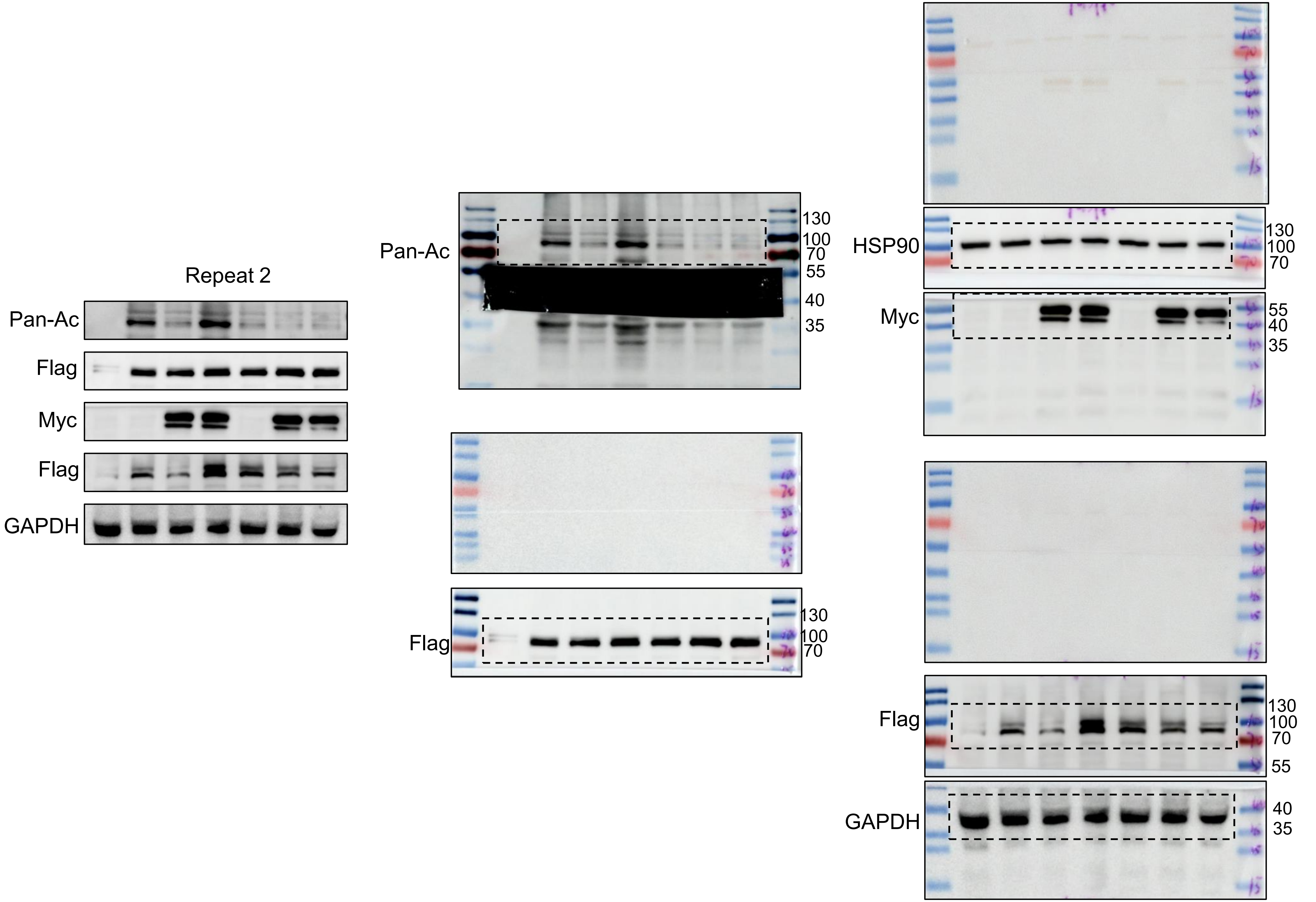
Repeat 2
Pan-Ac
Flag
Myc
Flag
GAPDH
Pan-Ac
130
100
70
55
40
35
Flag
130
100
70
HSP90
130
100
70
Myc
55
40
35
Flag
130
100
70
55
GAPDH
40
35

Figure 2I

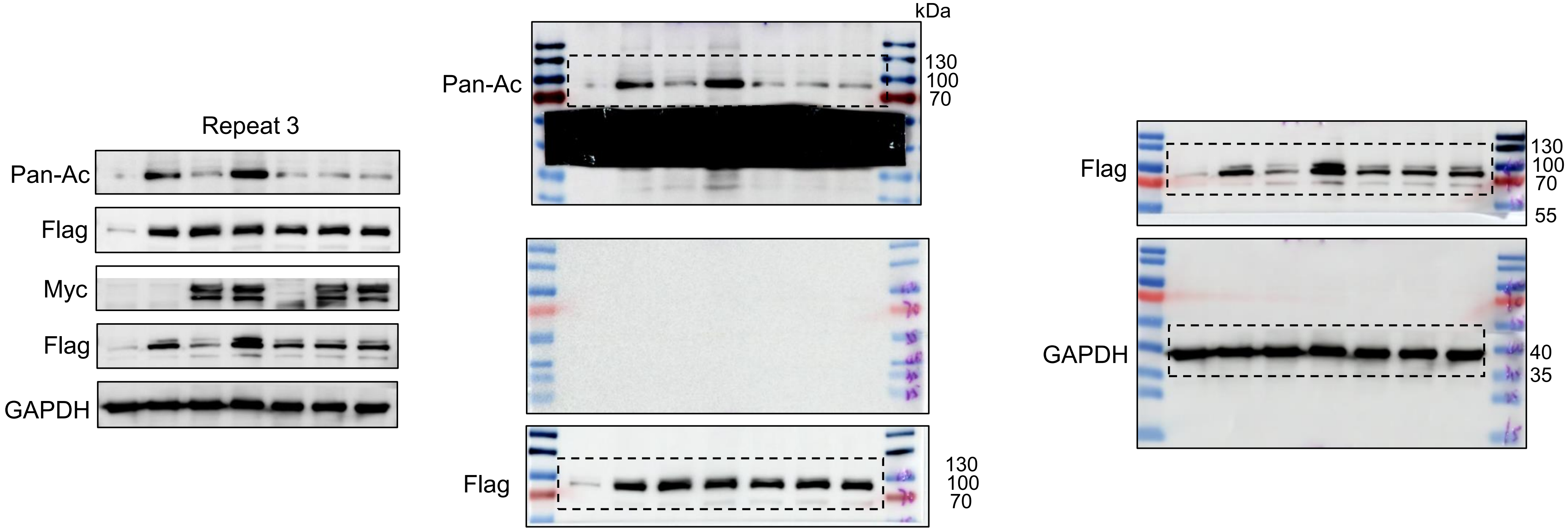
Repeat 3
Pan-Ac
Flag
Myc
Flag
GAPDH
kDa
Pan-Ac
130
100
70
Flag
130
100
70
Flag
130
100
70
55
GAPDH
40
35

Figure 2J

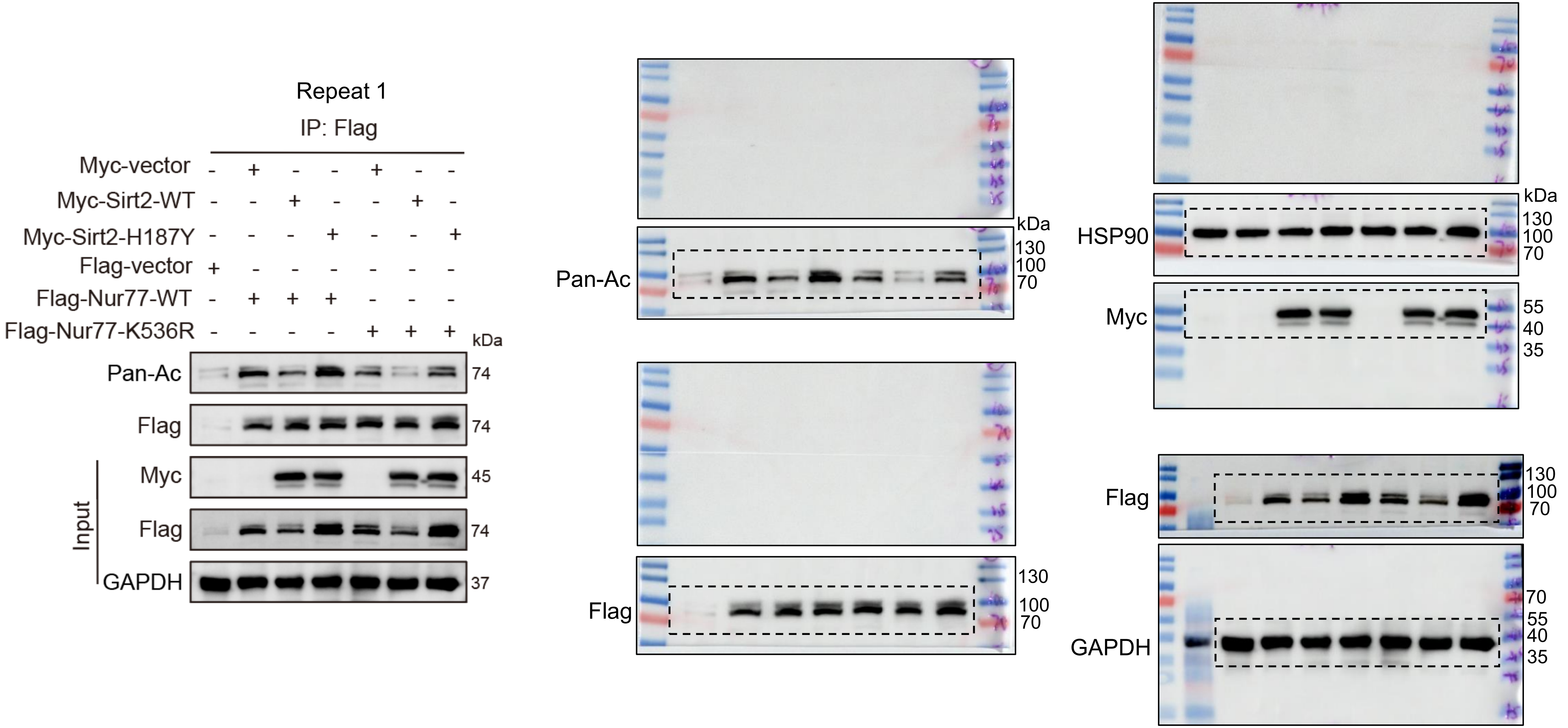
Repeat 1
IP: Flag
Myc-vector - + - - + - -
Myc-Sirt2-WT - - + - - + -
Myc-Sirt2-H187Y - - - + - - +
Flag-vector + - - - - - -
Flag-Nur77-WT - + + + - - -
Flag-Nur77-K536R - - - - + + +
kDa
Pan-Ac 74
Flag 74
Input
Myc 45
Flag 74
GAPDH 37
Pan-Ac
kDa
130
100
70
Flag
130
100
70
HSP90
kDa
130
100
70
Myc
55
40
35
Flag
130
100
70
GAPDH
70
55
40
35

Figure 2J

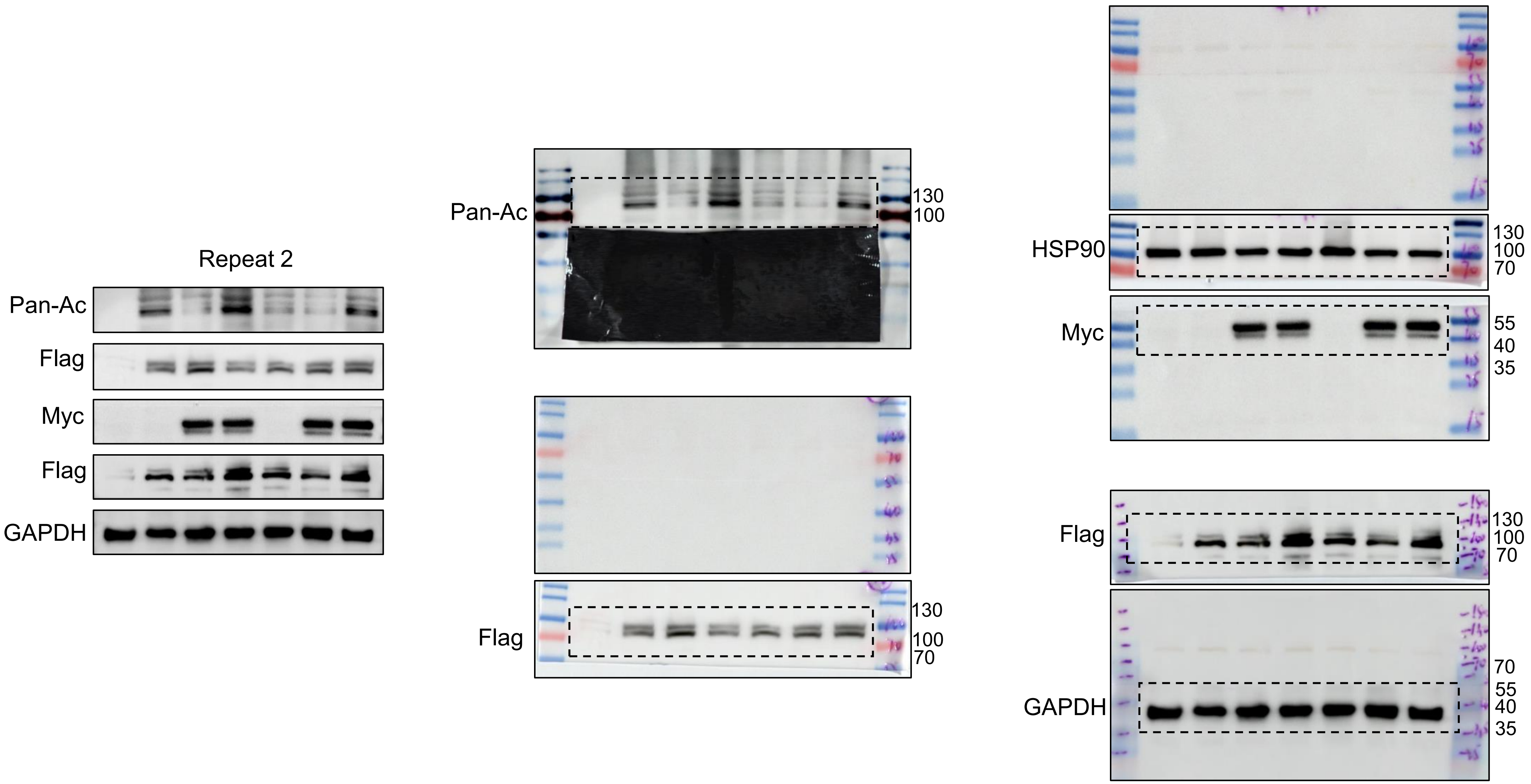
Repeat 2
Pan-Ac
Flag
Myc
Flag
GAPDH
Pan-Ac
130
100
Flag
130
100
70
HSP90
130
100
70
Myc
55
40
35
Flag
130
100
70
GAPDH
70
55
40
35

Figure 2J

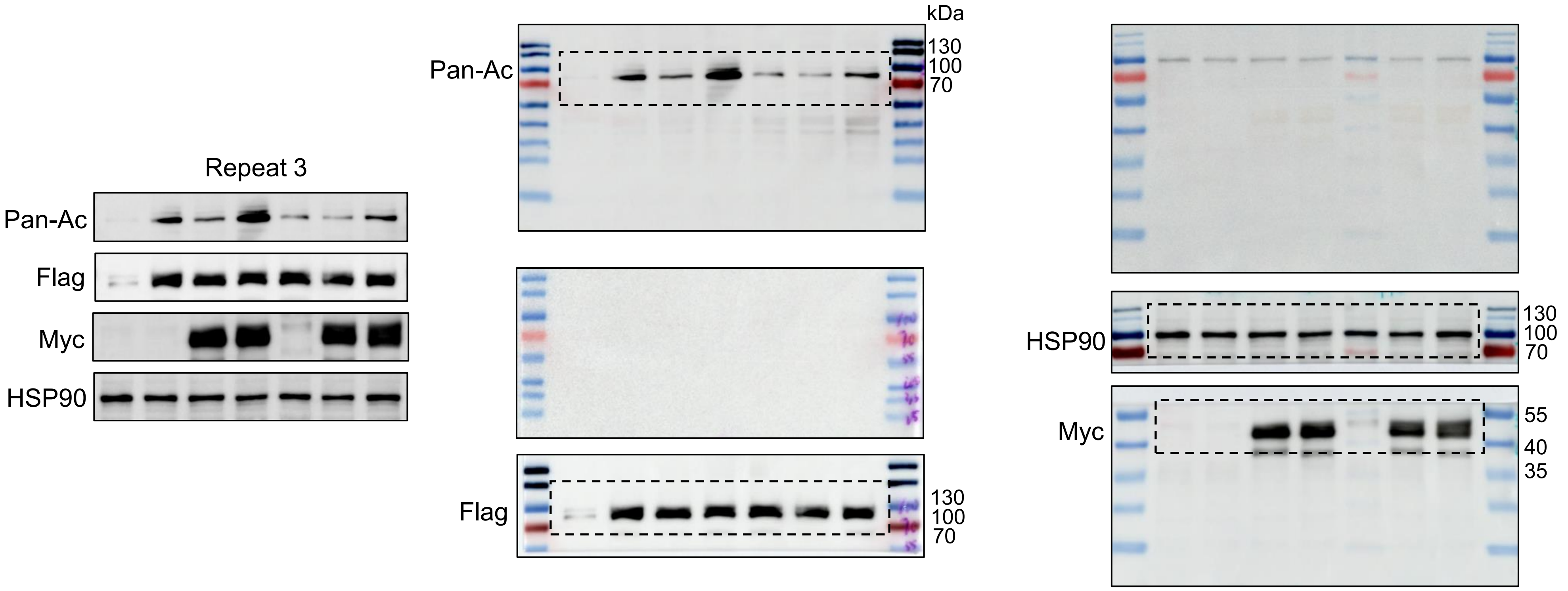

Repeat 3
Pan-Ac
Flag
Myc
HSP90
kDa
Pan-Ac
130
100
70
Flag
130
100
70
HSP90
130
100
70
Myc
55
40
35

Figure 2K

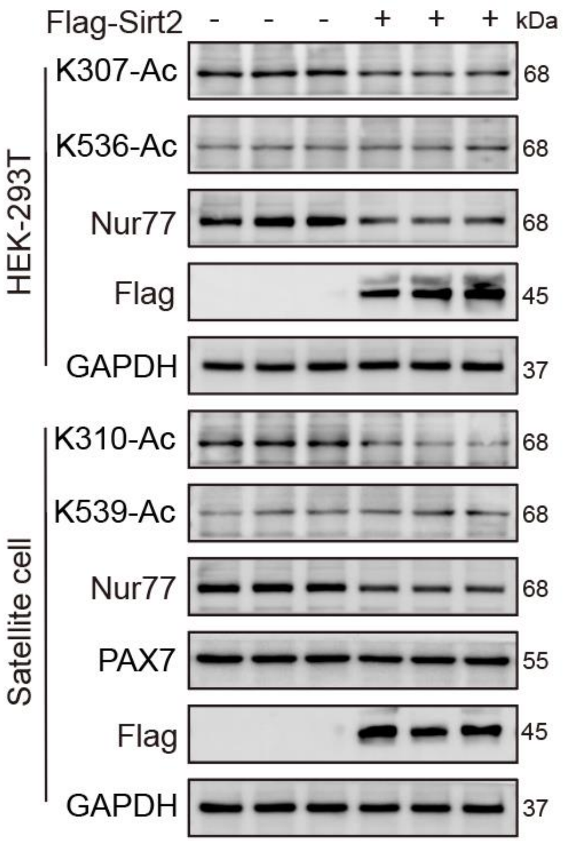

Flag-Sirt2 - - - + + + kDa
HEK-293T
K307-Ac 68
K536-Ac 68
Nur77 68
Flag 45
GAPDH 37
Satellite cell
K310-Ac 68
K539-Ac 68
Nur77 68
PAX7 55
Flag 45
GAPDH 37

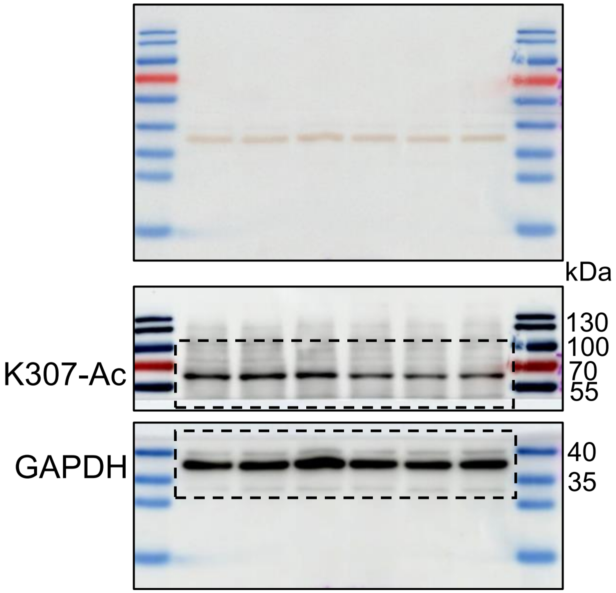

kDa
K307-Ac
130
100
70
55
GAPDH
40
35

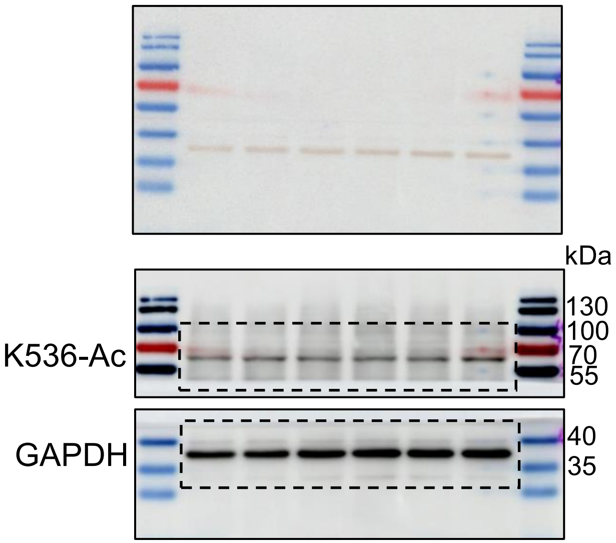

kDa
K536-Ac
130
100
70
55
GAPDH
40
35

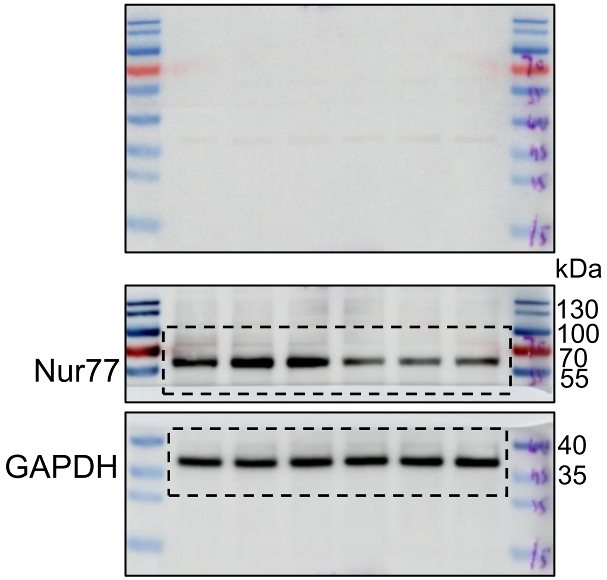

kDa
Nur77
130
100
70
55
GAPDH
40
35

Figure 2K

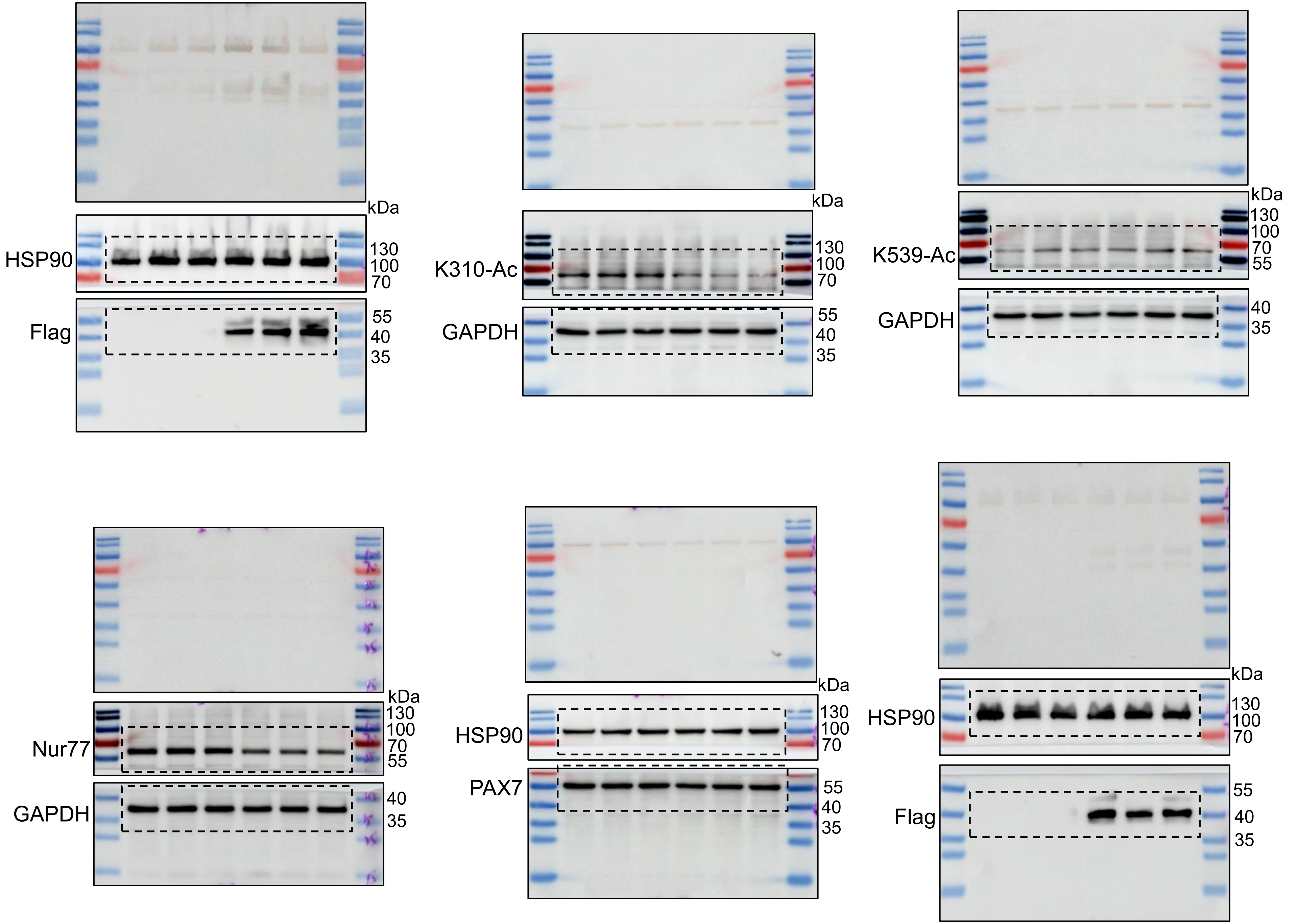
kDa
HSP90
130
100
70
Flag
55
40
35
kDa
K310-Ac
130
100
70
GAPDH
55
40
35
kDa
K539-Ac
130
100
70
55
GAPDH
40
35
kDa
Nur77
130
100
70
55
GAPDH
40
35
kDa
HSP90
130
100
70
PAX7
55
40
35
kDa
HSP90
130
100
70
Flag
55
40
35

Figure 2L

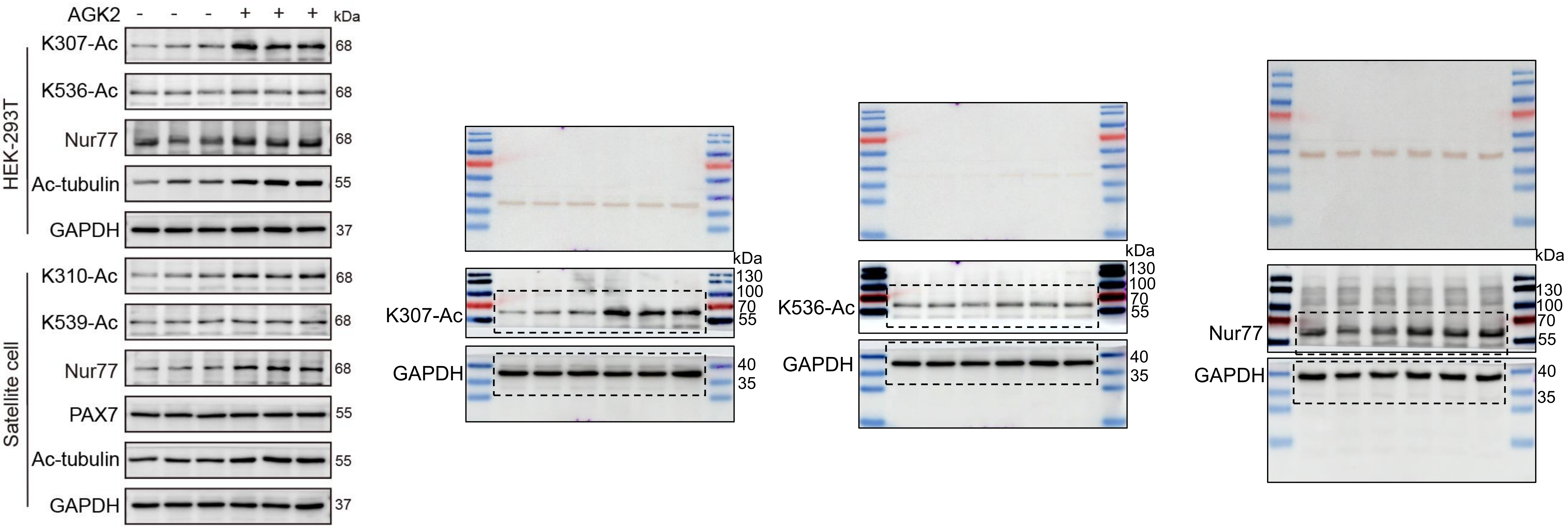
AGK2 - - - + + + kDa
HEK-293T
K307-Ac 68
K536-Ac 68
Nur77 68
Ac-tubulin 55
GAPDH 37
Satellite cell
K310-Ac 68
K539-Ac 68
Nur77 68
PAX7 55
Ac-tubulin 55
GAPDH 37
K307-Ac
GAPDH
K536-Ac
GAPDH
Nur77
GAPDH
kDa
130
100
70
55
40
35

Figure 2L

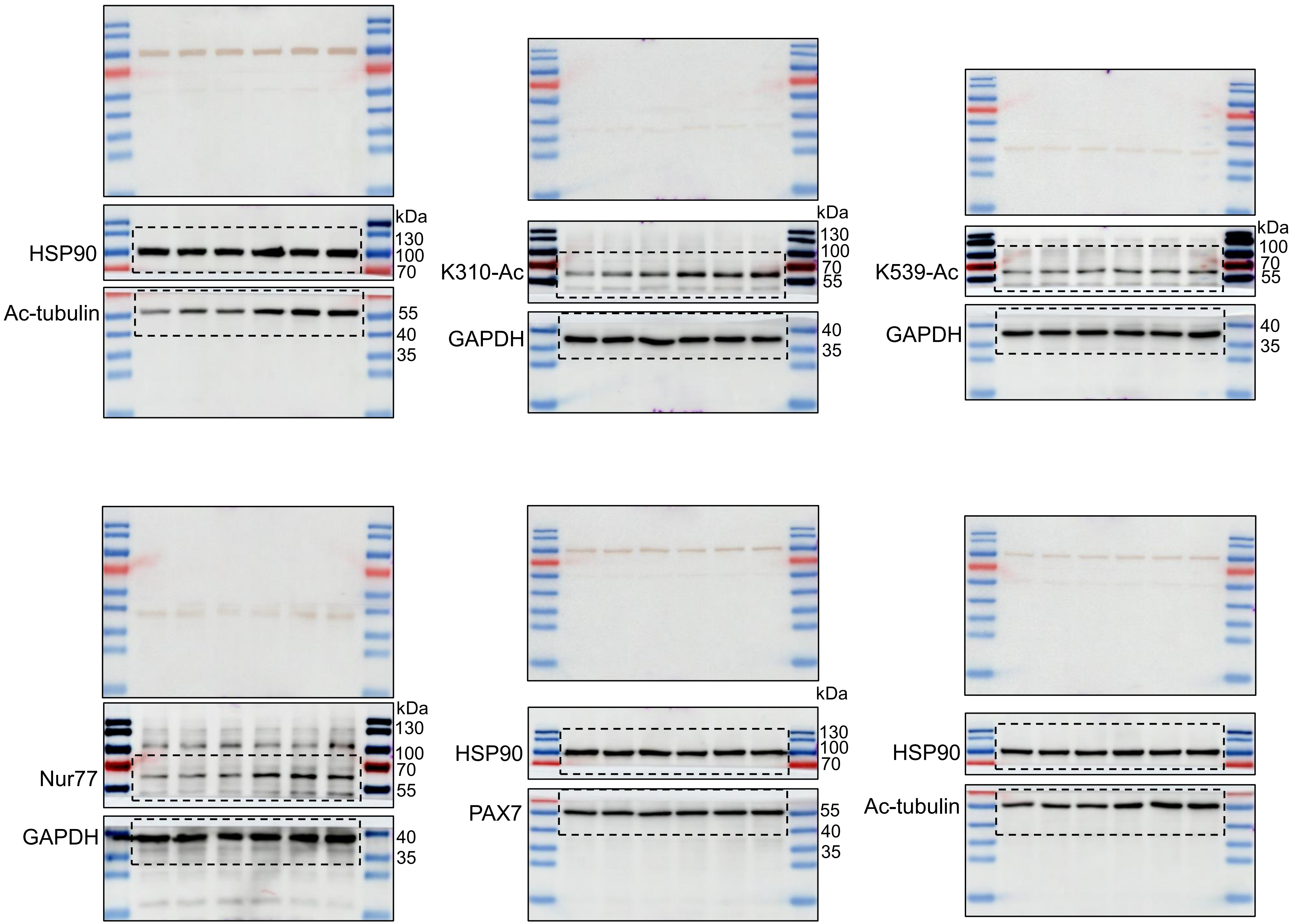
kDa
HSP90
130
100
70
Ac-tubulin
55
40
35
kDa
K310-Ac
130
100
70
55
GAPDH
40
35
kDa
K539-Ac
100
70
55
GAPDH
40
35
kDa
Nur77
130
100
70
55
GAPDH
40
35
kDa
HSP90
130
100
70
PAX7
55
40
35
HSP90
Ac-tubulin

Figure 3A

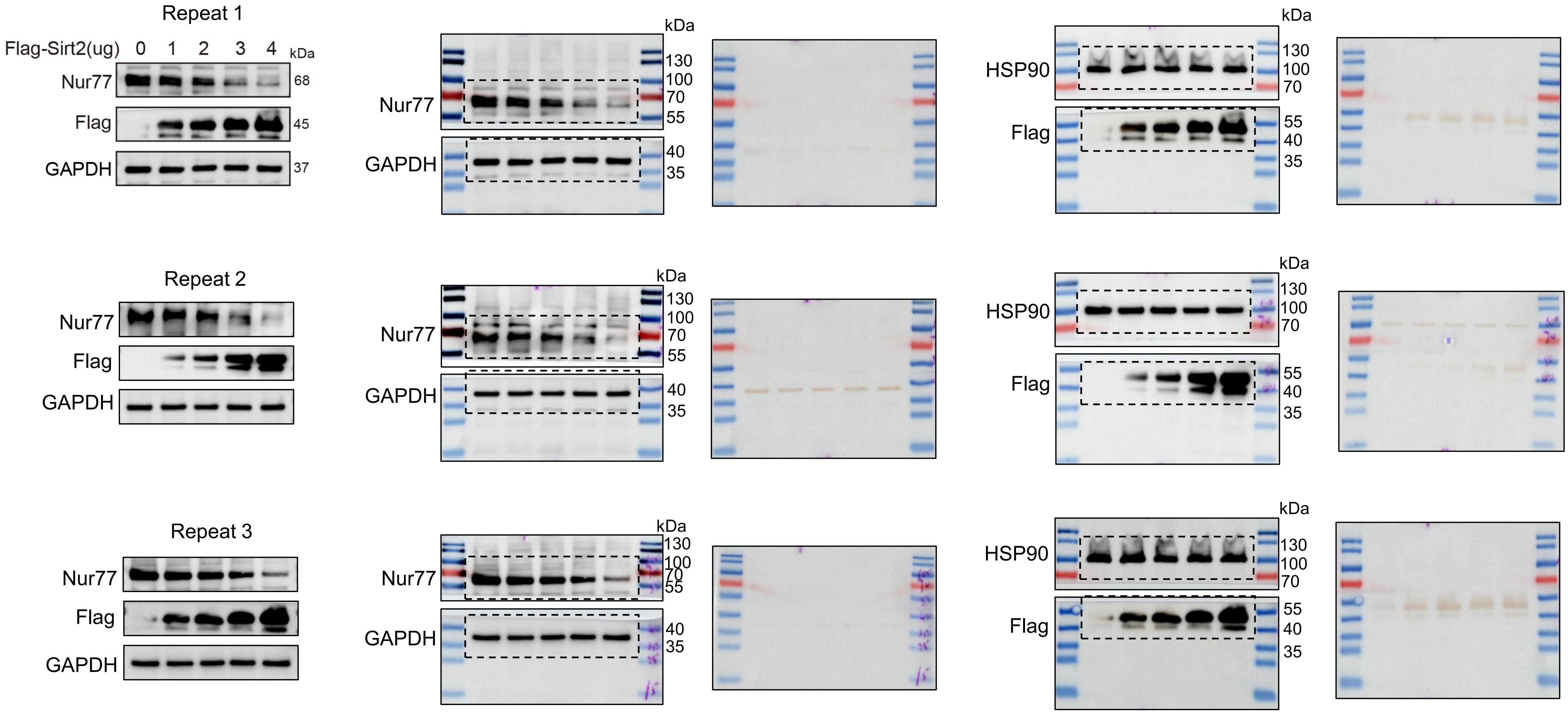
Repeat 1
Flag-Sirt2(ug) 0 1 2 3 4
kDa
Nur77
68
Flag
45
GAPDH
37
Nur77
GAPDH
kDa
130
100
70
55
40
35
HSP90
Flag
kDa
130
100
70
55
40
35
Repeat 2
Nur77
Flag
GAPDH
Nur77
GAPDH
kDa
130
100
70
55
40
35
HSP90
Flag
kDa
130
100
70
55
40
35
Repeat 3
Nur77
Flag
GAPDH
Nur77
GAPDH
kDa
130
100
70
55
40
35
HSP90
Flag
kDa
130
100
70
55
40
35

Figure 3B

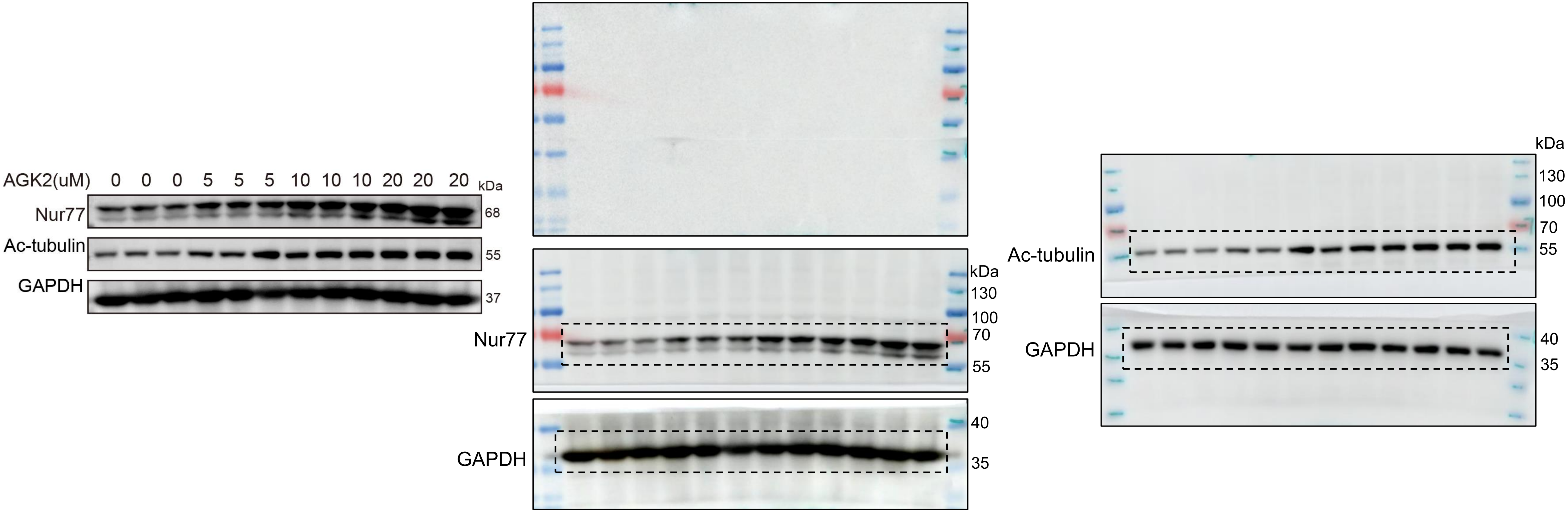
AGK2(uM) 0 0 0 5 5 5 10 10 10 20 20 20
kDa
Nur77
68
Ac-tubulin
55
GAPDH
37
kDa
130
100
70
55
Nur77
40
35
GAPDH
kDa
130
100
70
55
Ac-tubulin
40
35
GAPDH

Figure 3C

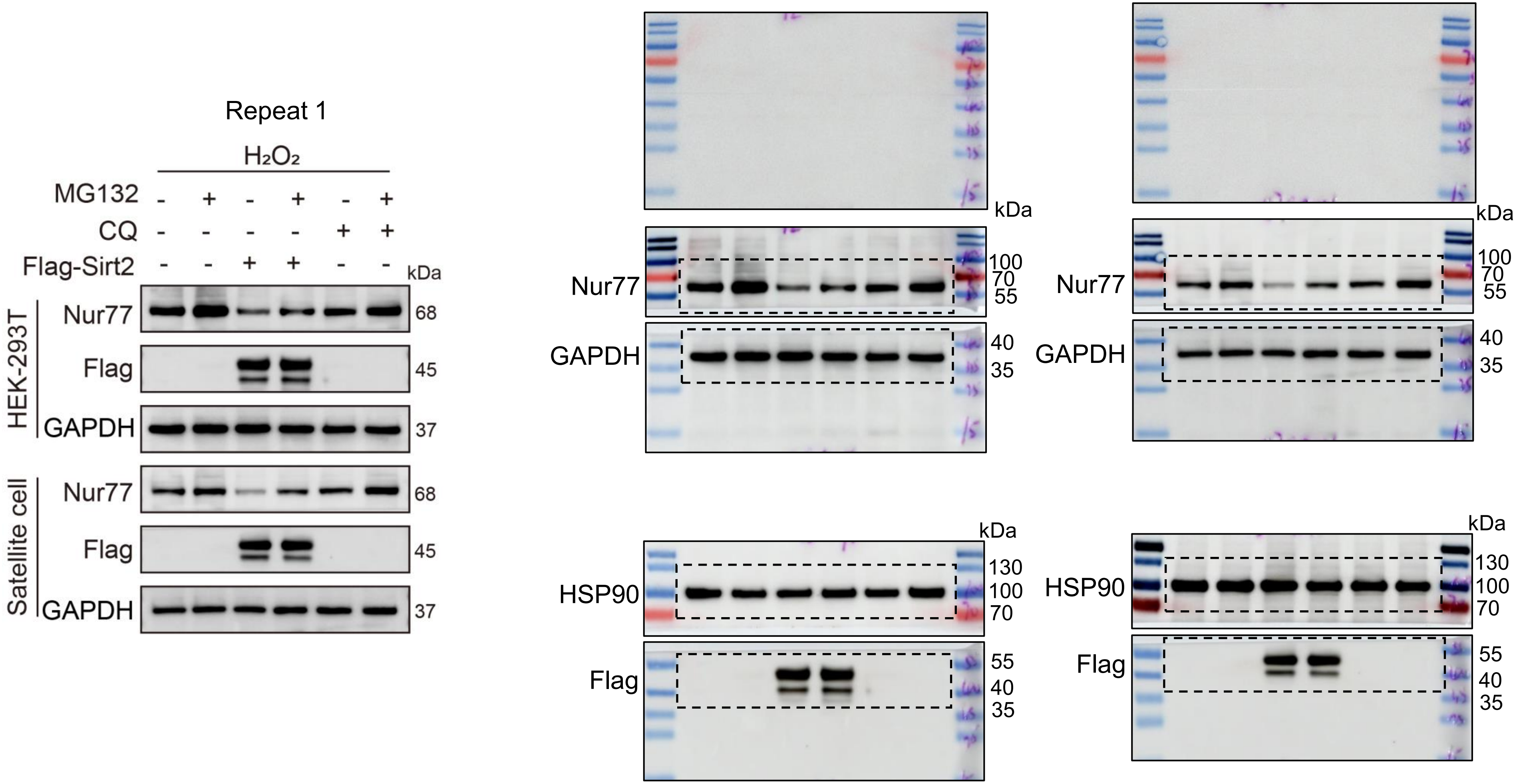

Repeat 1
H2O2
MG132 - + - + - +
CQ - - - - + +
Flag-Sirt2 - - + + - -
kDa
HEK-293T
Nur77
68
Flag
45
GAPDH
37
Satellite cell
Nur77
68
Flag
45
GAPDH
37
kDa
Nur77
100
70
55
GAPDH
40
35
kDa
Nur77
100
70
55
GAPDH
40
35
kDa
HSP90
130
100
70
Flag
55
40
35
kDa
HSP90
130
100
70
Flag
55
40
35

Figure 3C

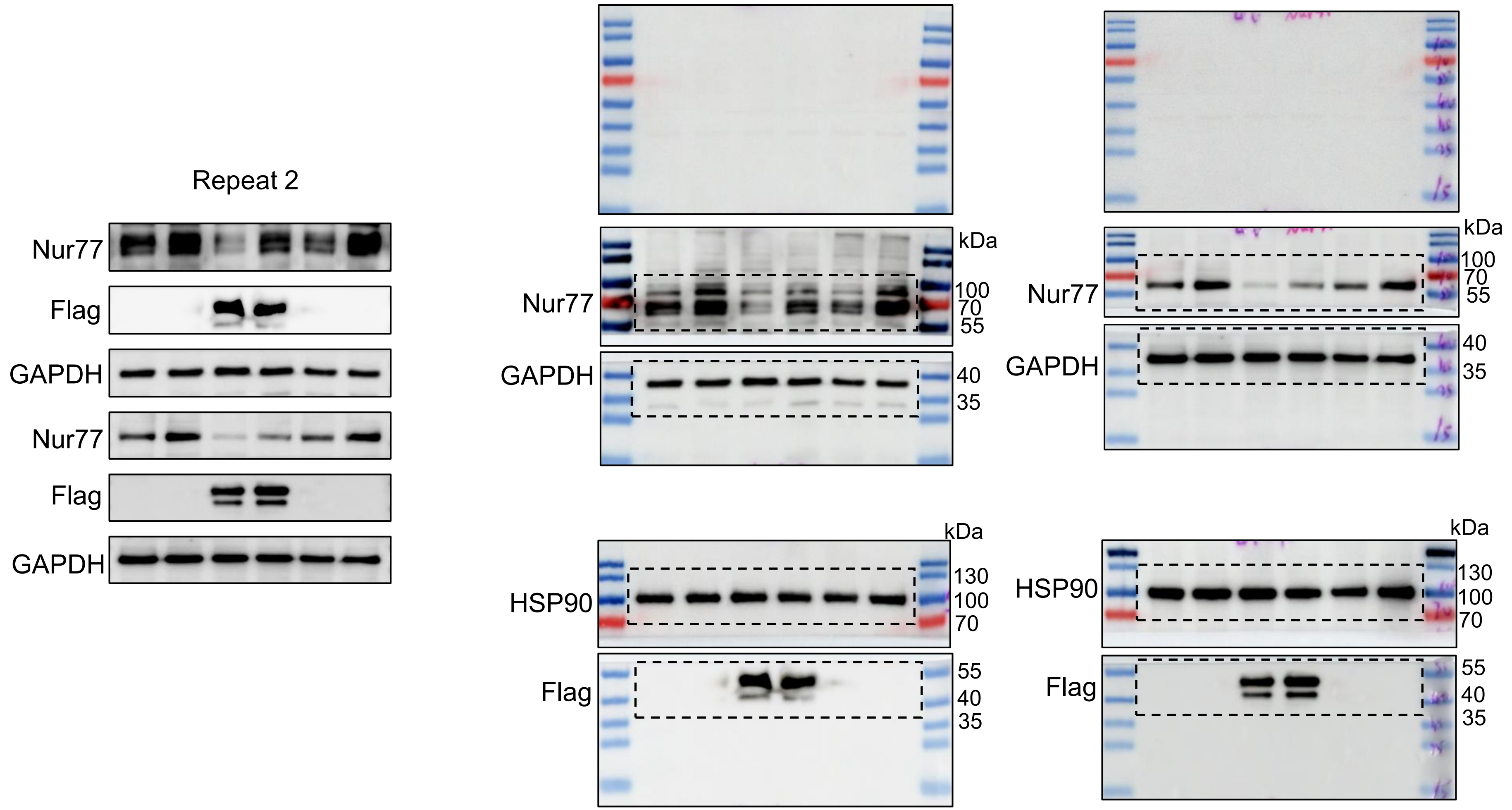

Repeat 2
Nur77
Flag
GAPDH
Nur77
Flag
GAPDH
kDa
Nur77
100
70
55
GAPDH
40
35
kDa
Nur77
100
70
55
GAPDH
40
35
kDa
HSP90
130
100
70
Flag
55
40
35
kDa
HSP90
130
100
70
Flag
55
40
35

Figure 3C

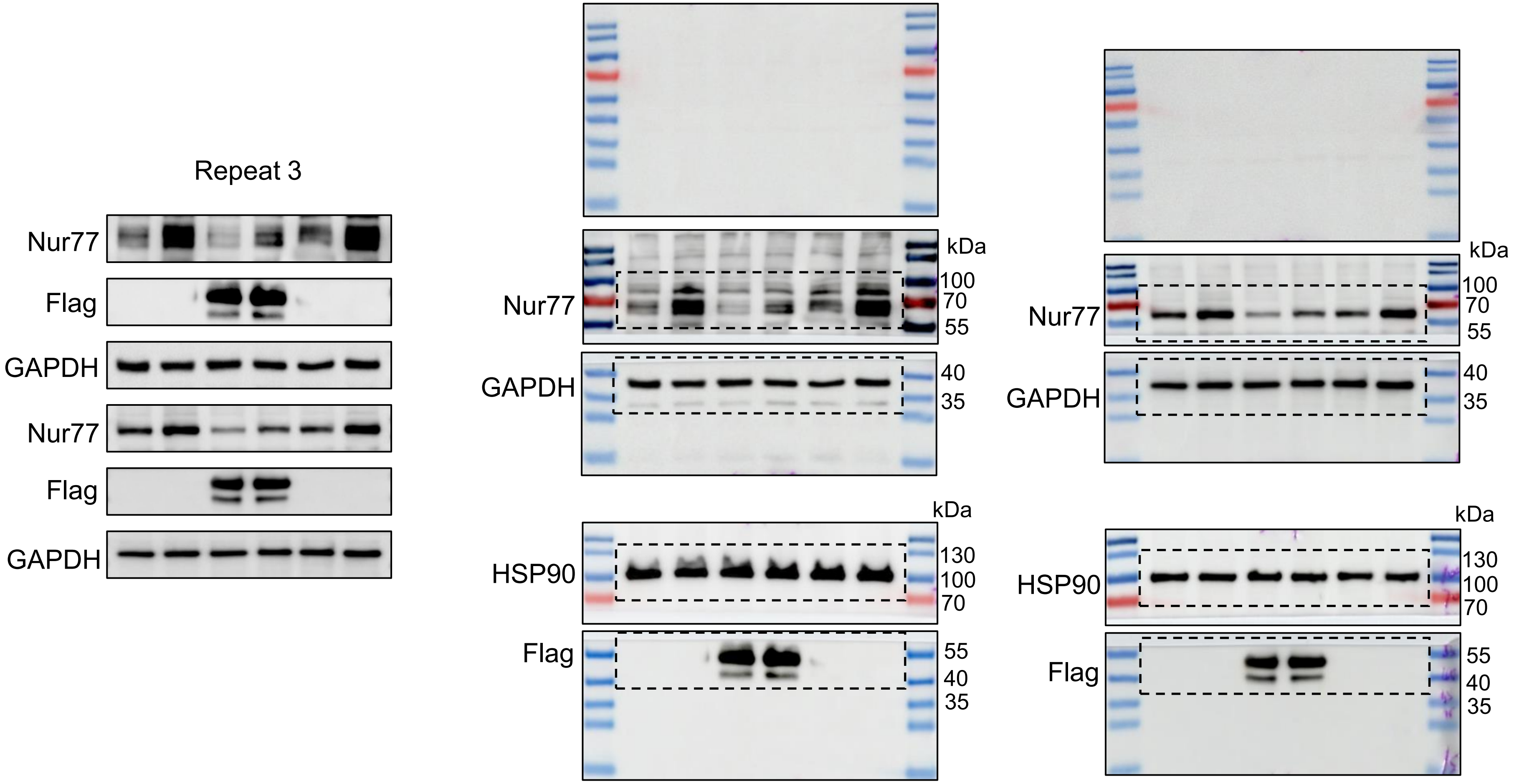
Repeat 3
Nur77
Flag
GAPDH
Nur77
Flag
GAPDH
Nur77
GAPDH
HSP90
Flag
kDa
100
70
55
40
35
130
100
70
55
40
35
Nur77
GAPDH
HSP90
Flag
kDa
100
70
55
40
35
130
100
70
55
40
35

Figure 3D

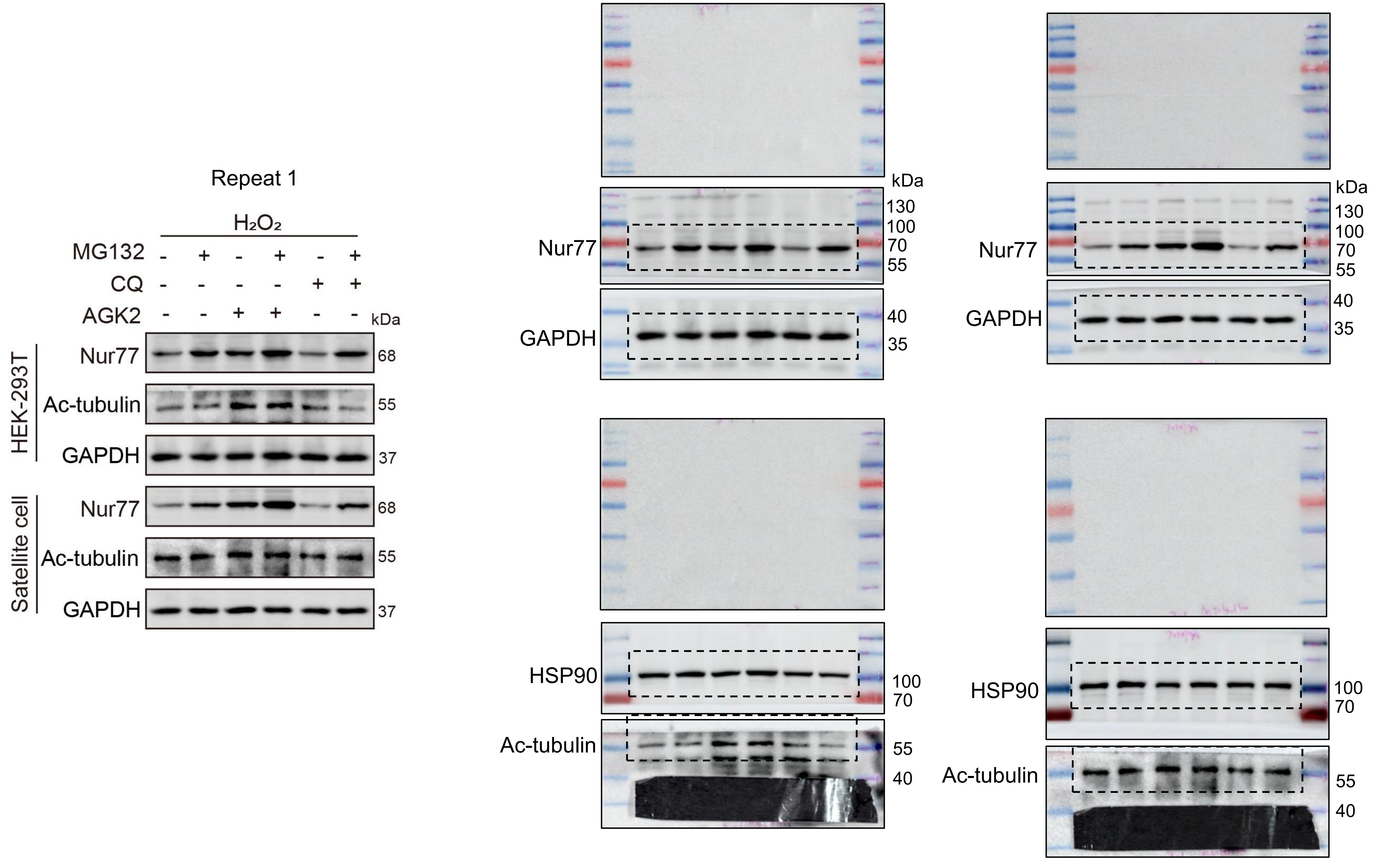

Repeat 1
H_2O_2
MG132 - + - + - +
CQ - - - - + +
AGK2 - - + + - -
kDa
HEK-293T
Nur77 68
Ac-tubulin 55
GAPDH 37
Satellite cell
Nur77 68
Ac-tubulin 55
GAPDH 37
kDa
Nur77
130
100
70
55
GAPDH
40
35
HSP90
100
70
Ac-tubulin
55
40

Figure 3D

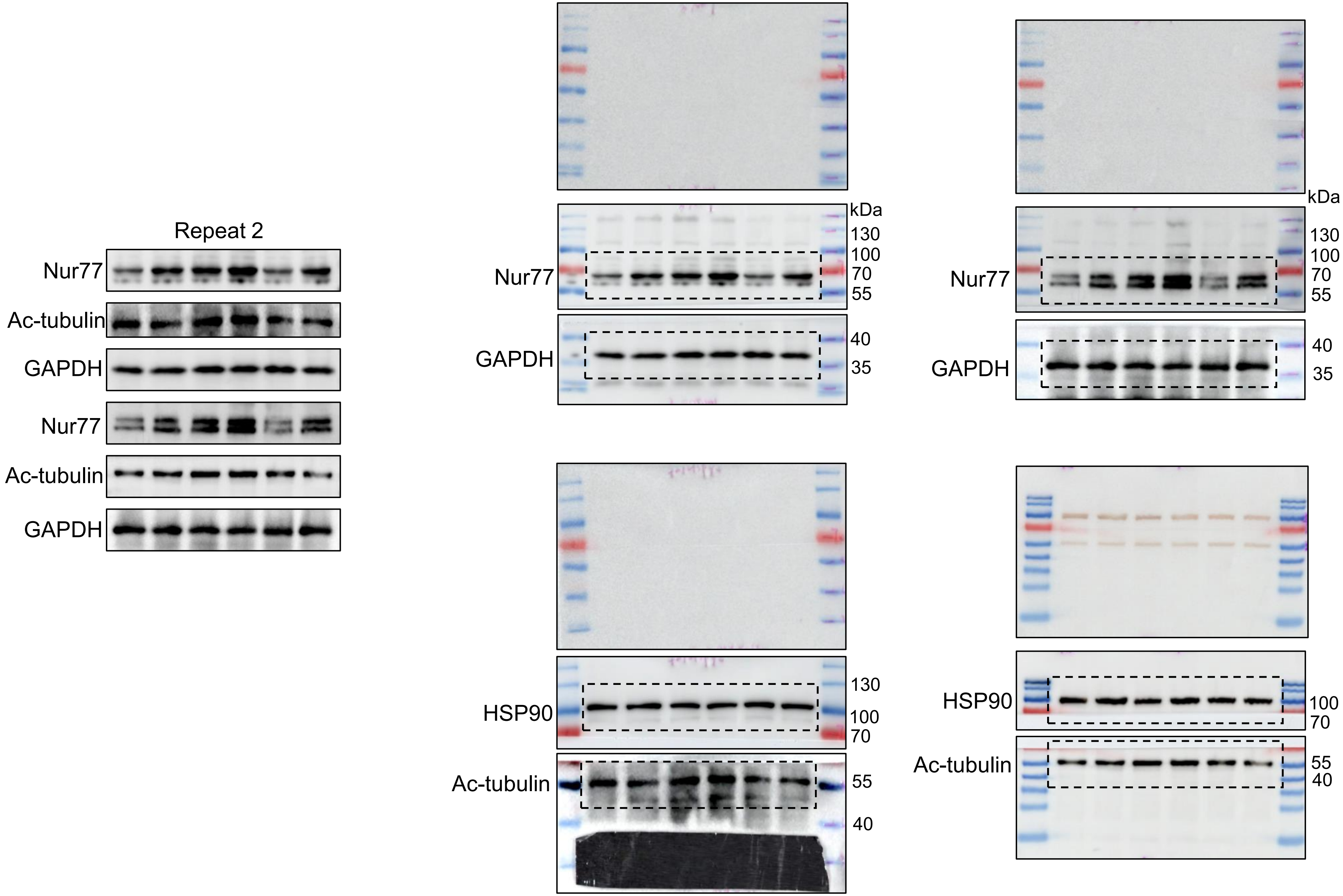

Repeat 2
Nur77
Ac-tubulin
GAPDH
Nur77
Ac-tubulin
GAPDH
kDa
130
100
70
55
40
35
Nur77
GAPDH
kDa
130
100
70
55
40
35
Nur77
GAPDH
130
100
70
55
40
HSP90
Ac-tubulin
100
70
55
40
HSP90
Ac-tubulin

Figure 3D

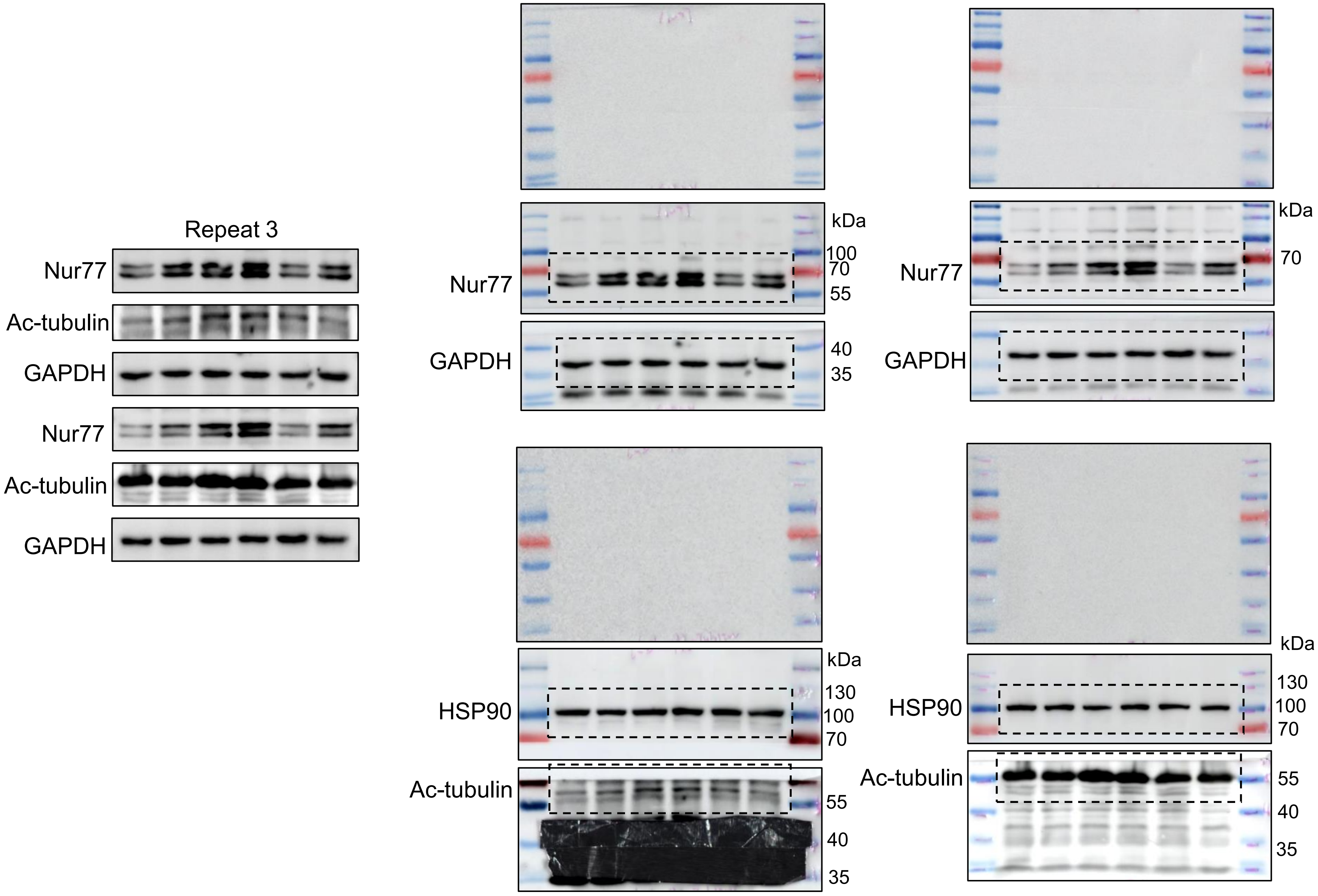
Repeat 3
Nur77
Ac-tubulin
GAPDH
Nur77
Ac-tubulin
GAPDH
Nur77
kDa
100
70
55
GAPDH
40
35
Nur77
kDa
70
GAPDH
HSP90
kDa
130
100
70
Ac-tubulin
55
40
35
HSP90
kDa
130
100
70
Ac-tubulin
55
40
35

Figure 3E

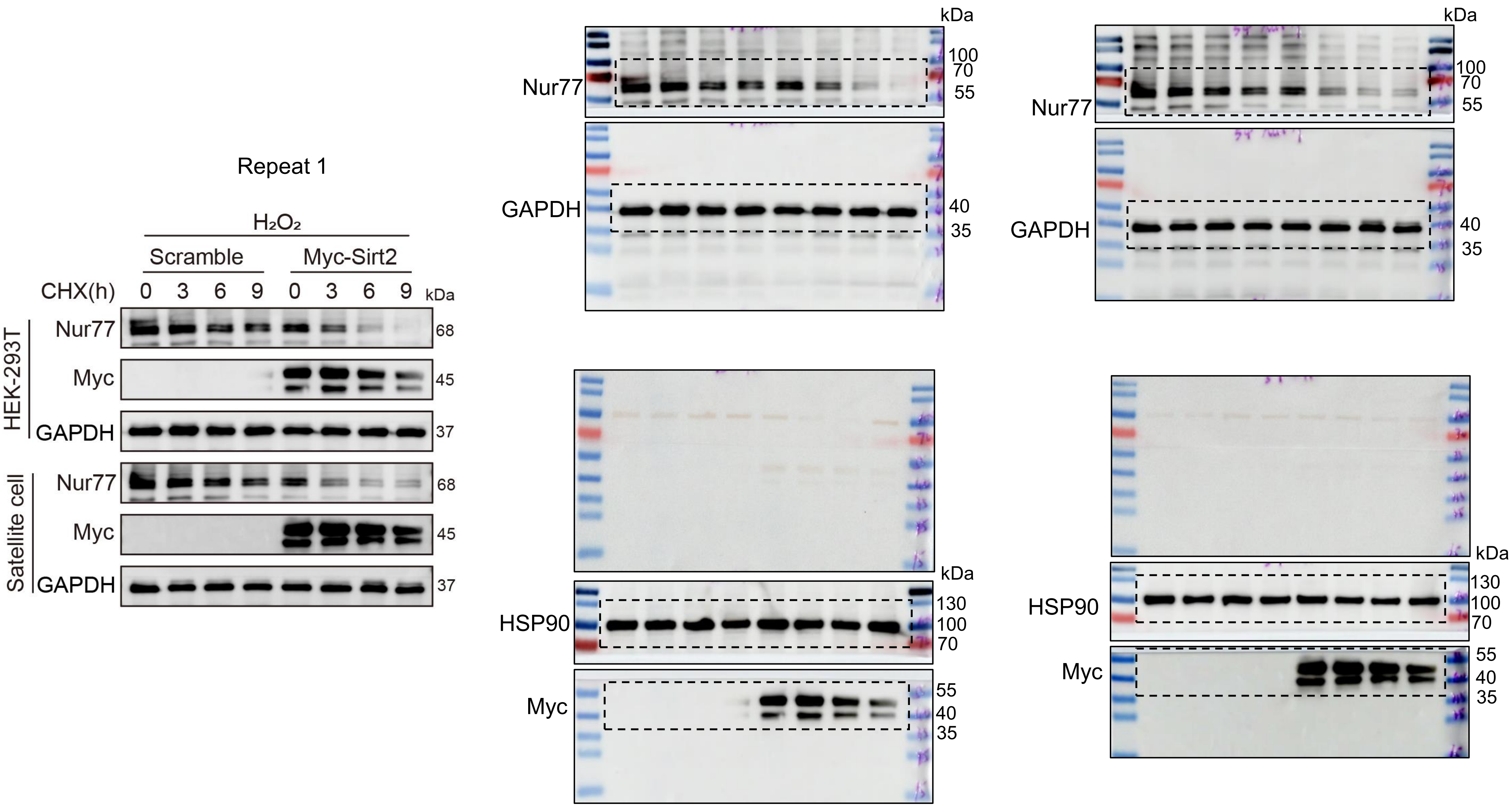

Repeat 1
H₂O₂
Scramble
Myc-Sirt2
CHX(h) 0 3 6 9 0 3 6 9
kDa
HEK-293T
Nur77 68
Myc 45
GAPDH 37
Satellite cell
Nur77 68
Myc 45
GAPDH 37
kDa
Nur77
100
70
55
GAPDH
40
35
HSP90
130
100
70
Myc
55
40
35

Figure 3E

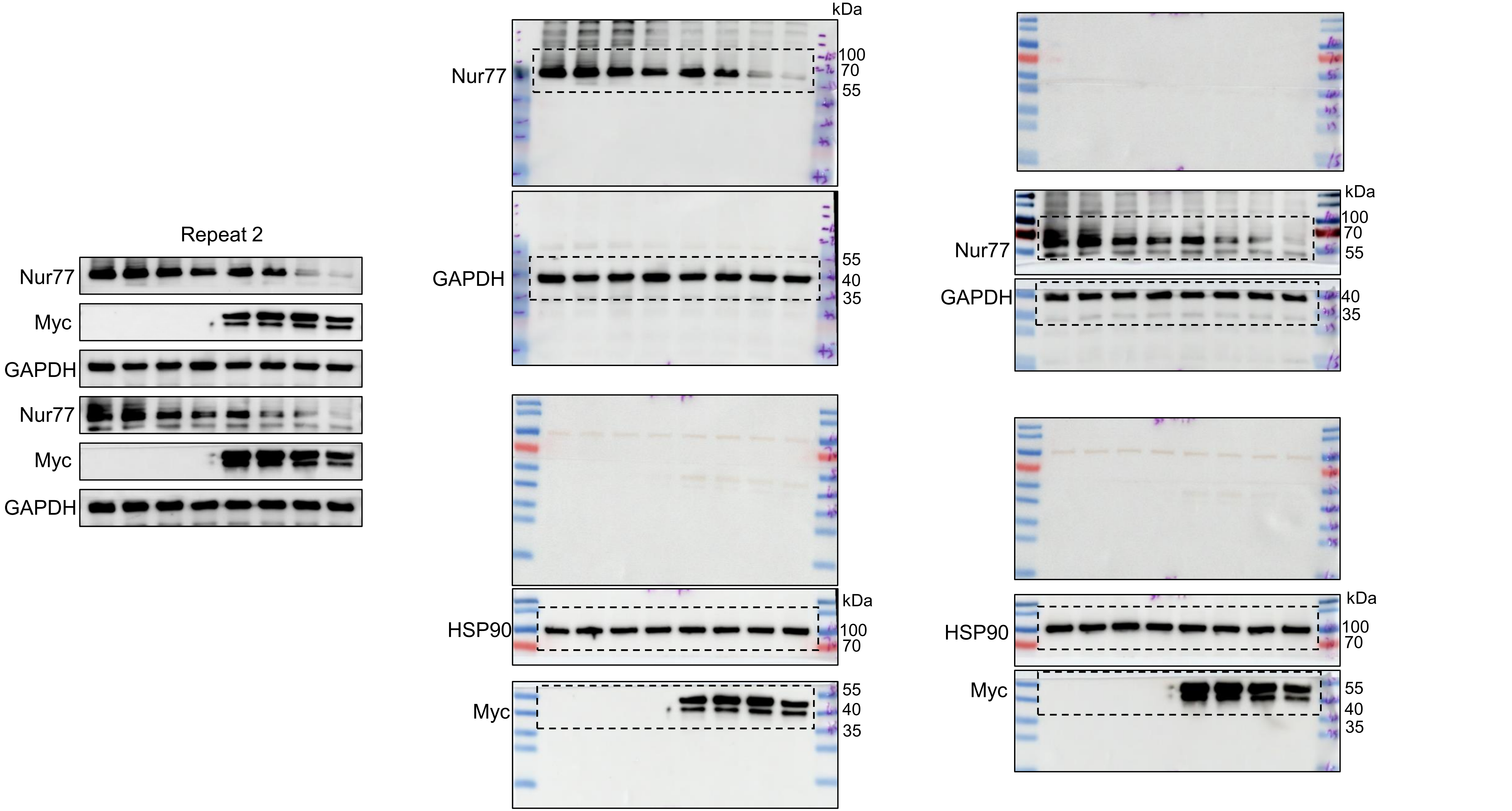
Repeat 2
Nur77
Myc
GAPDH
Nur77
Myc
GAPDH
kDa
Nur77
100
70
55
GAPDH
55
40
35
kDa
HSP90
100
70
Myc
55
40
35
kDa
Nur77
100
70
55
GAPDH
40
35
kDa
HSP90
100
70
Myc
55
40
35

Figure 3E

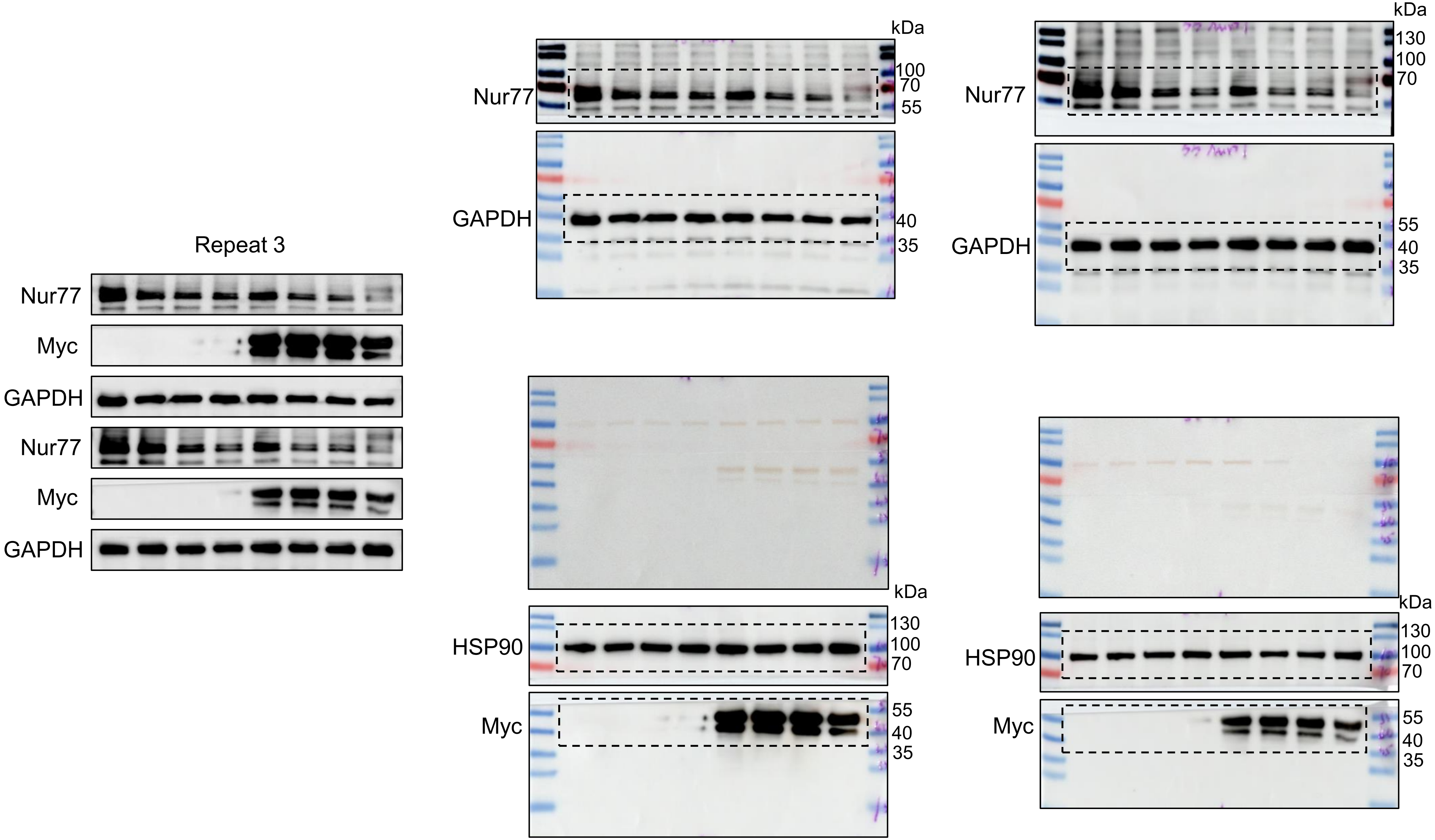
Repeat 3
Nur77
Myc
GAPDH
Nur77
Myc
GAPDH
kDa
Nur77
100
70
55
GAPDH
40
35
kDa
130
100
70
Nur77
GAPDH
55
40
35
kDa
HSP90
130
100
70
Myc
55
40
35
kDa
HSP90
130
100
70
Myc
55
40
35

Figure 3H

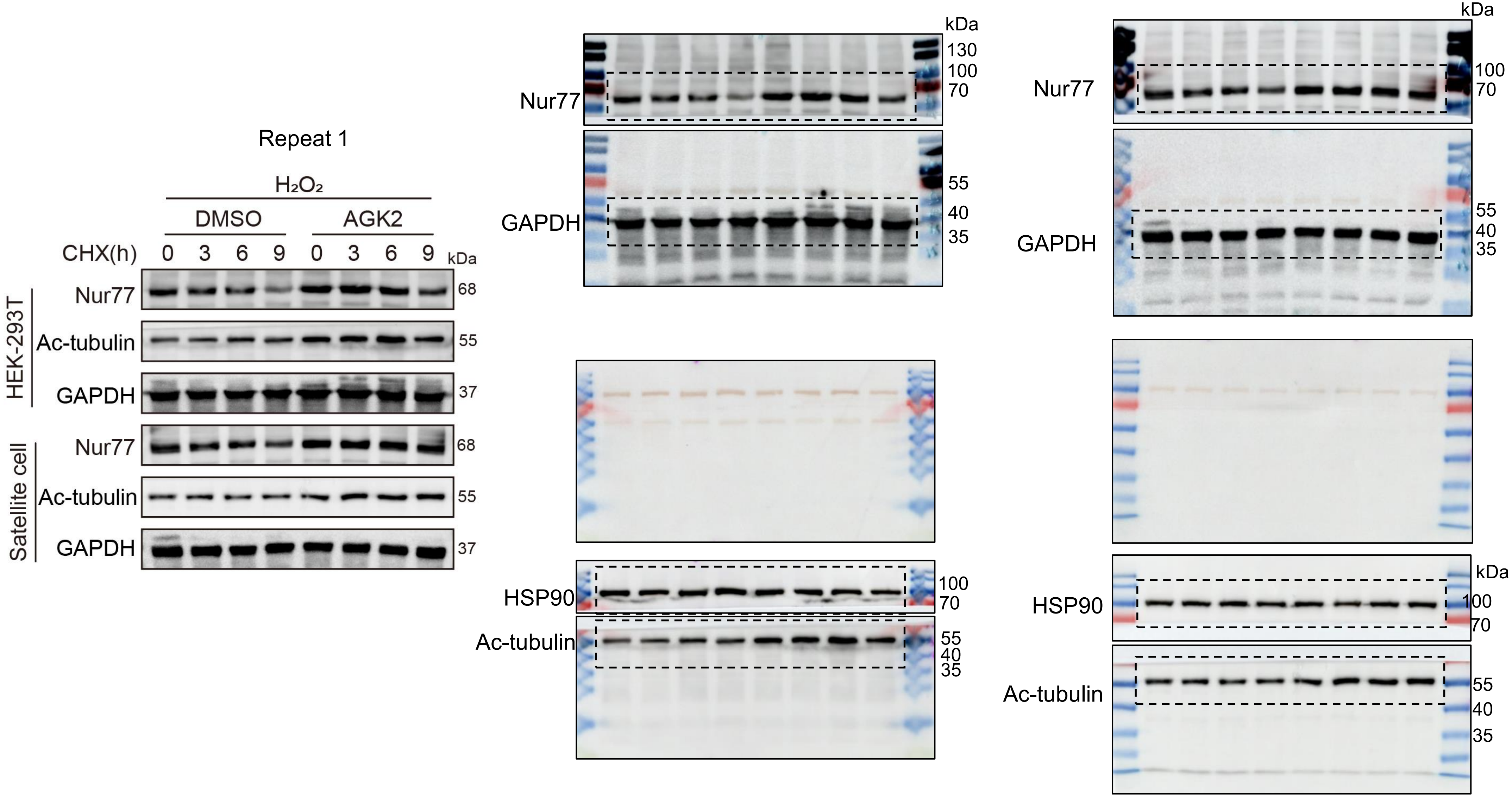
Repeat 1
H₂O₂
DMSO
AGK2
CHX(h)
0 3 6 9 0 3 6 9
kDa
HEK-293T
Satellite cell
Nur77
Ac-tubulin
GAPDH
HSP90
68
55
37
130
100
70
40
35

Figure 3H

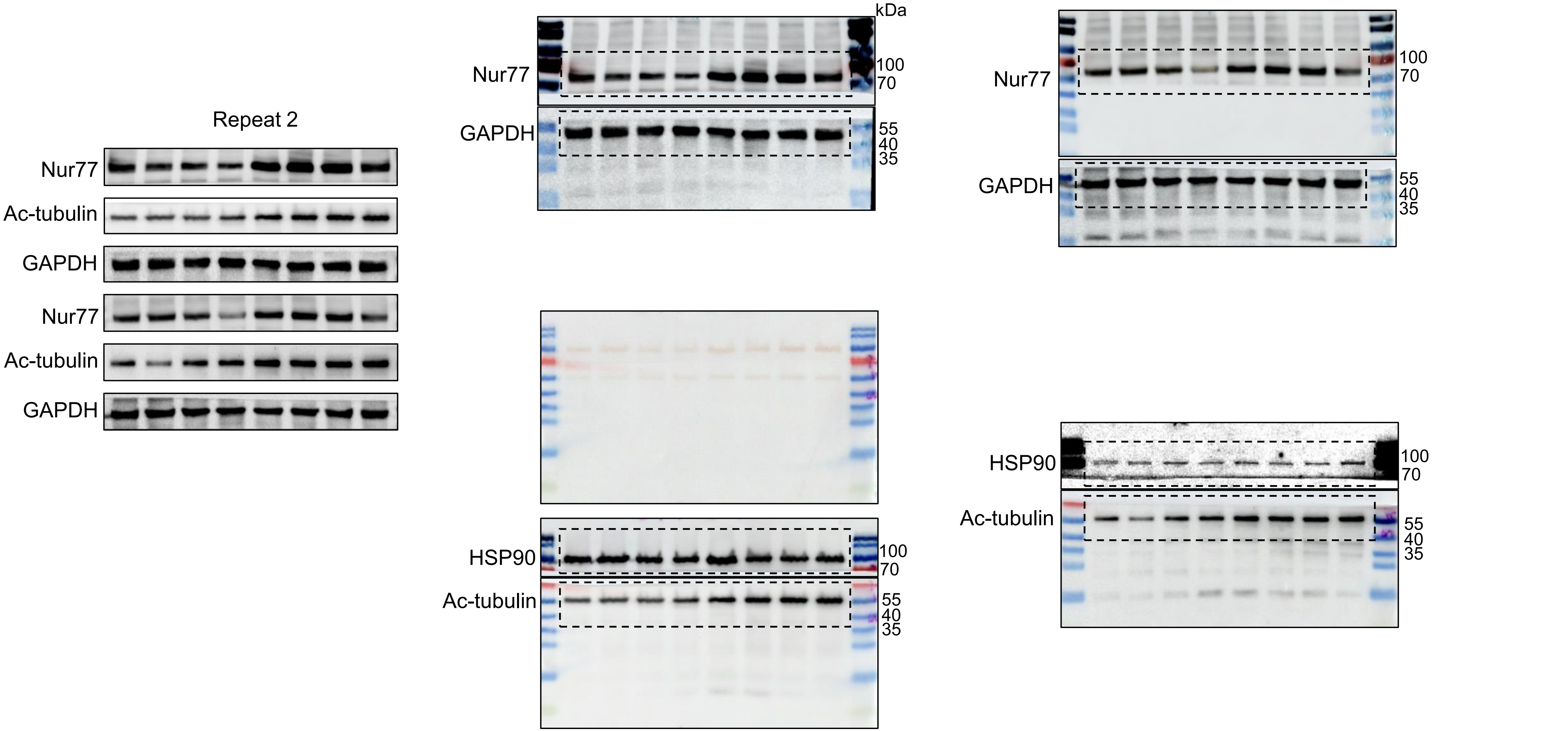
Repeat 2
Nur77
Ac-tubulin
GAPDH
Nur77
Ac-tubulin
GAPDH
kDa
Nur77
100
70
GAPDH
55
40
35
HSP90
100
70
Ac-tubulin
55
40
35
Nur77
100
70
GAPDH
55
40
35
HSP90
100
70
Ac-tubulin
55
40
35

Figure 3H

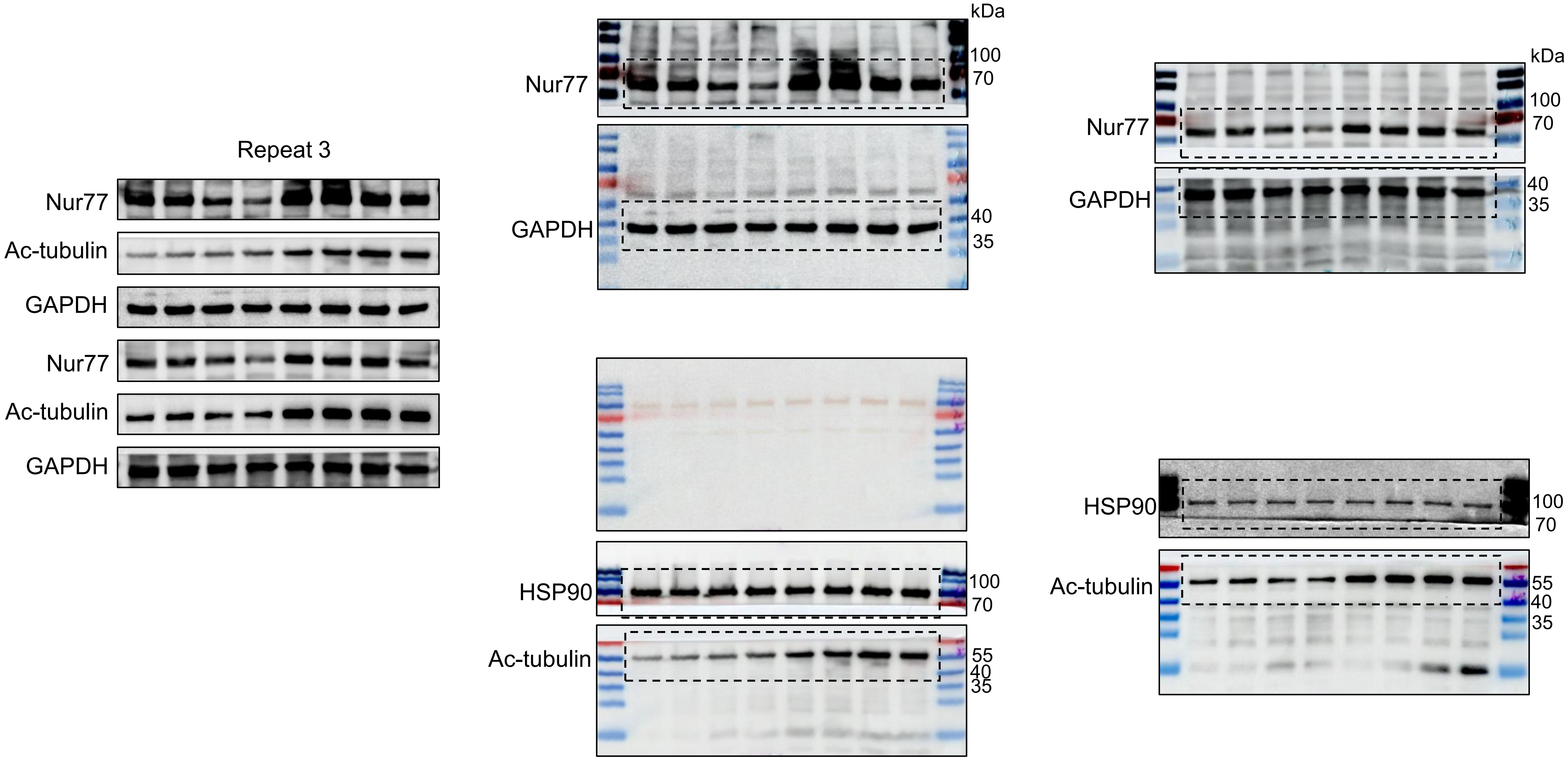

Repeat 3
Nur77
Ac-tubulin
GAPDH
Nur77
Ac-tubulin
GAPDH
kDa
100
70
Nur77
GAPDH
40
35
HSP90
100
70
Ac-tubulin
55
40
35
kDa
100
70
Nur77
GAPDH
40
35
HSP90
100
70
Ac-tubulin
55
40
35

Figure 3K

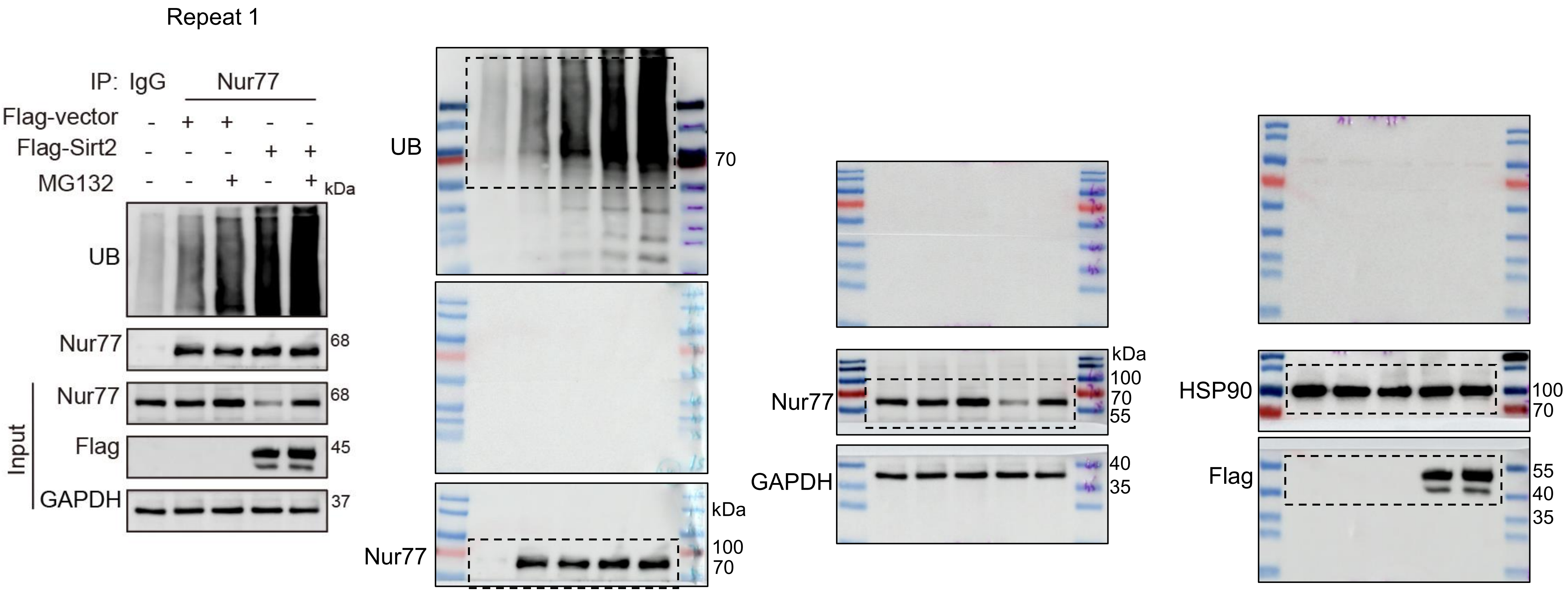

Repeat 1
IP: IgG Nur77
Flag-vector - + + - -
Flag-Sirt2 - - - + +
MG132 - - + - +
kDa
UB
Nur77
68
Input
Nur77
68
Flag
45
GAPDH
37
UB
70
Nur77
kDa
100
70
Nur77
kDa
100
70
55
GAPDH
40
35
HSP90
100
70
Flag
55
40
35

Figure 3K

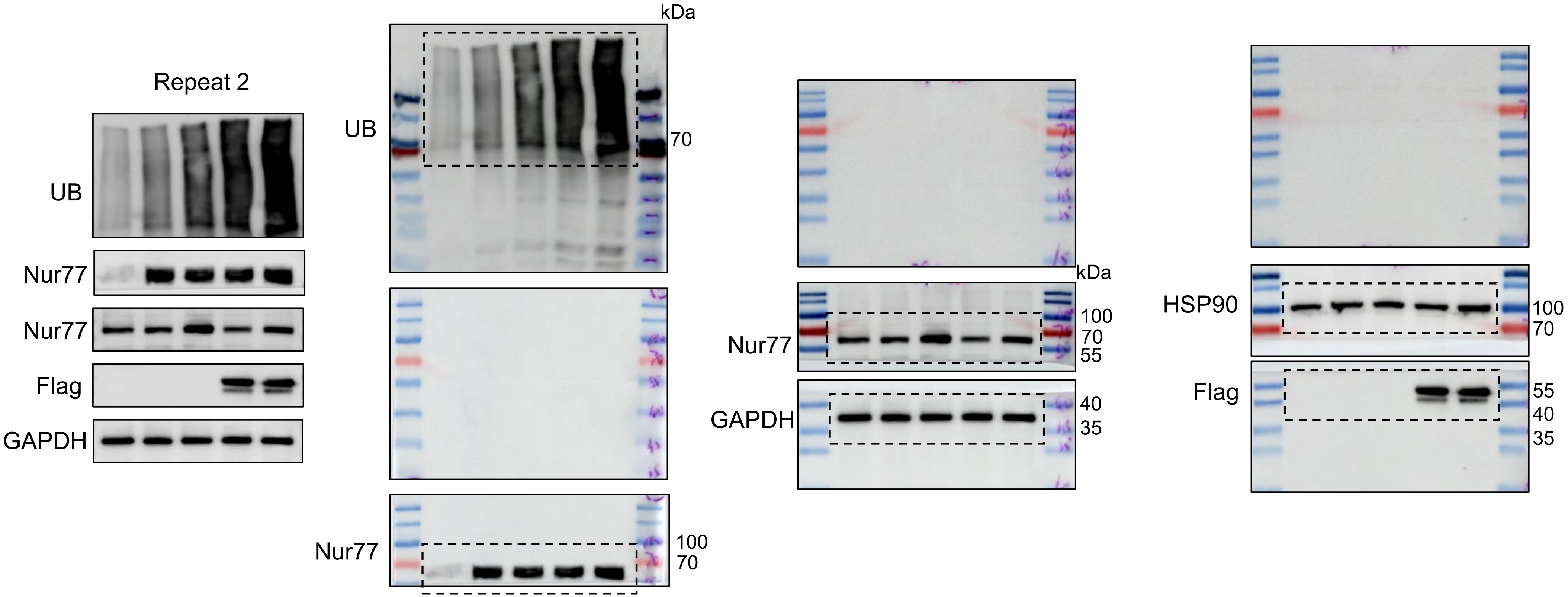

Repeat 2
UB
Nur77
Nur77
Flag
GAPDH
kDa
UB
70
Nur77
100
70
kDa
Nur77
100
70
55
GAPDH
40
35
HSP90
100
70
Flag
55
40
35

Figure 3K

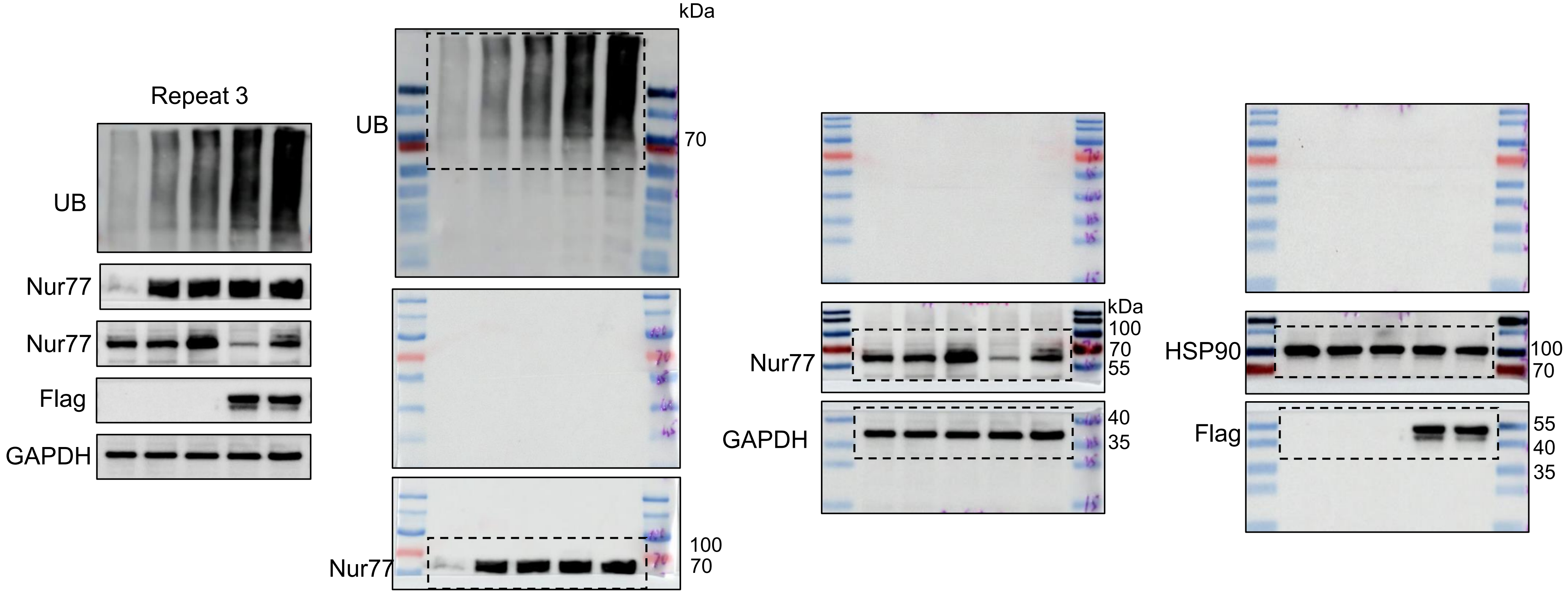
Repeat 3
UB
Nur77
Nur77
Flag
GAPDH
kDa
UB
70
Nur77
100
70
kDa
Nur77
100
70
55
GAPDH
40
35
HSP90
100
70
Flag
55
40
35

Figure 3L

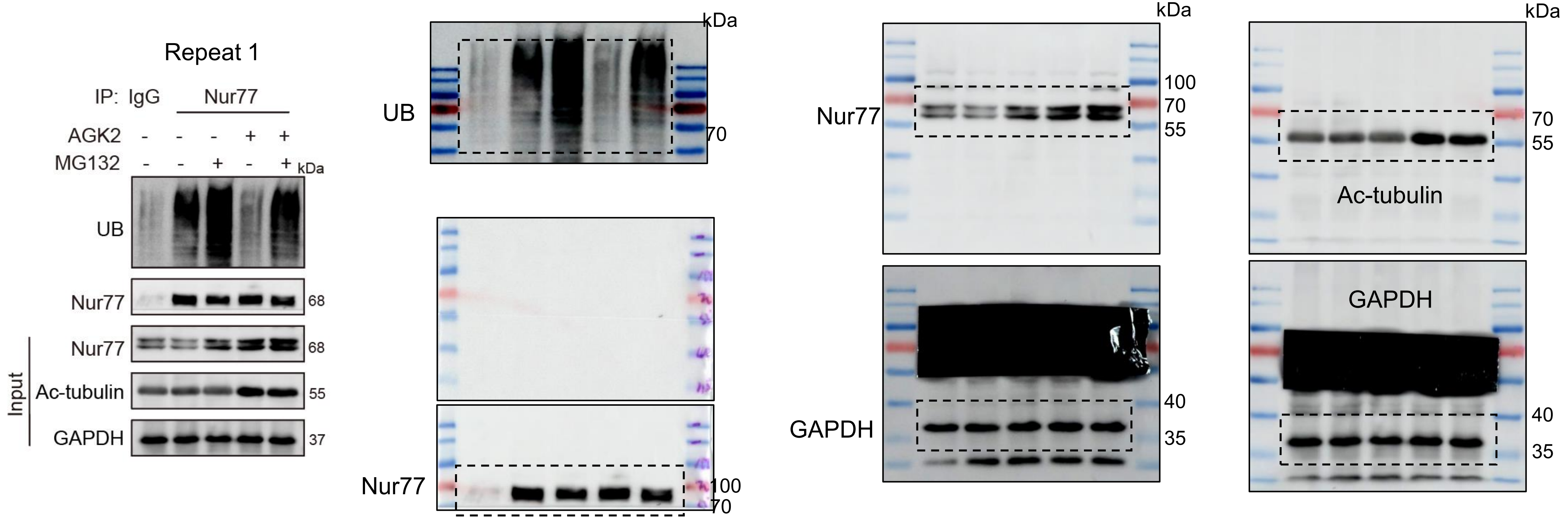
Repeat 1
IP: IgG Nur77
AGK2 - - - + +
MG132 - - + - +
kDa
UB
Nur77 68
Nur77 68
Ac-tubulin 55
GAPDH 37
Input
UB
kDa
70
Nur77
100
70
Nur77
kDa
100
70
55
GAPDH
40
35
kDa
70
55
Ac-tubulin
GAPDH
40
35

Figure 3L

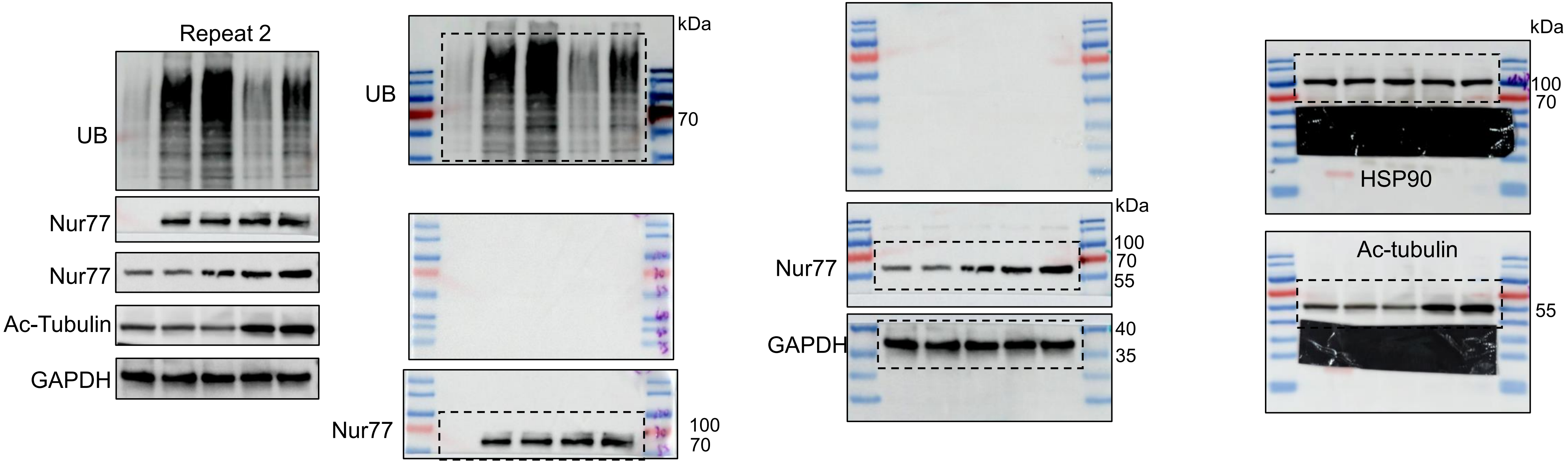
Repeat 2
UB
Nur77
Nur77
Ac-Tubulin
GAPDH
UB
kDa
70
Nur77
100
70
Nur77
kDa
100
70
55
GAPDH
40
35
kDa
100
70
HSP90
Ac-tubulin
55

Figure 3L

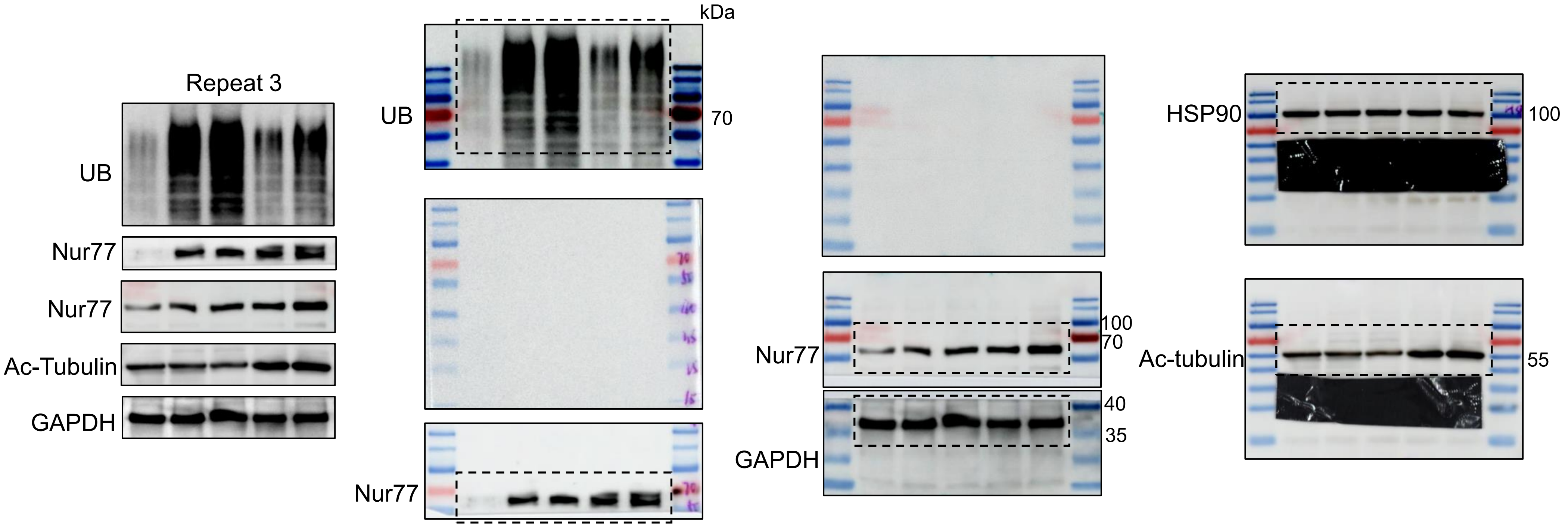
Repeat 3
UB
Nur77
Nur77
Ac-Tubulin
GAPDH
kDa
UB
70
Nur77
Nur77
100
70
GAPDH
40
35
HSP90
100
Ac-tubulin
55

Figure 3M

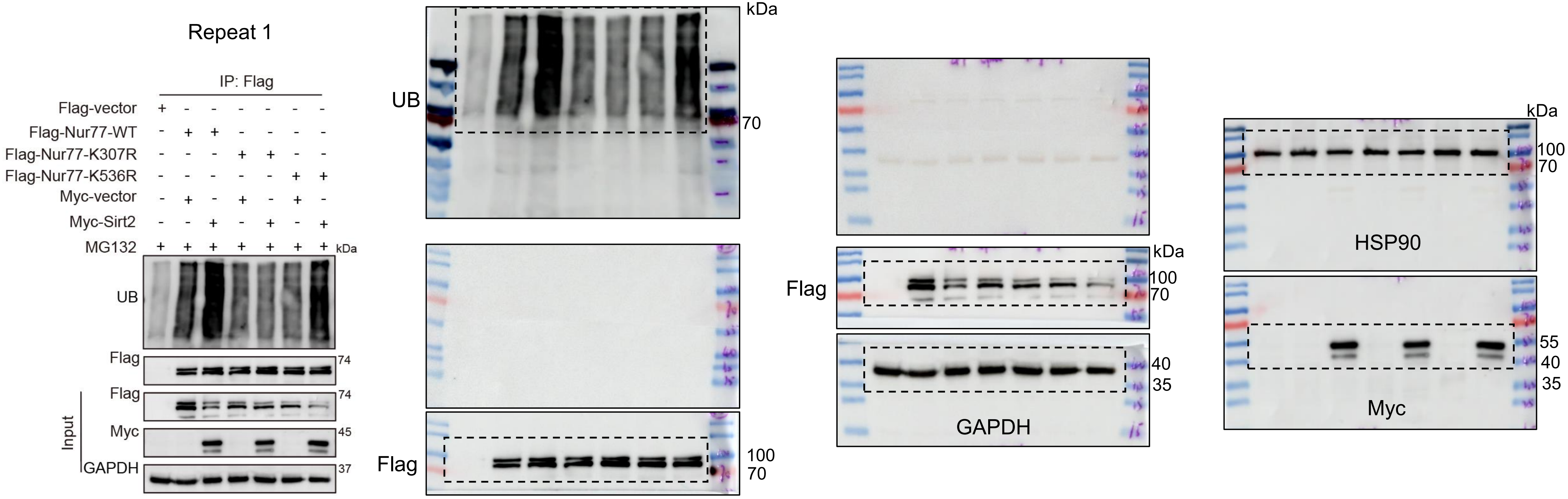

Repeat 1
IP: Flag
Flag-vector
Flag-Nur77-WT
Flag-Nur77-K307R
Flag-Nur77-K536R
Myc-vector
Myc-Sirt2
MG132
kDa
UB
Flag
74
Input
Flag
74
Myc
45
GAPDH
37
UB
kDa
70
Flag
100
70
Flag
kDa
100
70
40
35
GAPDH
kDa
100
70
HSP90
55
40
35
Myc

Figure 3M

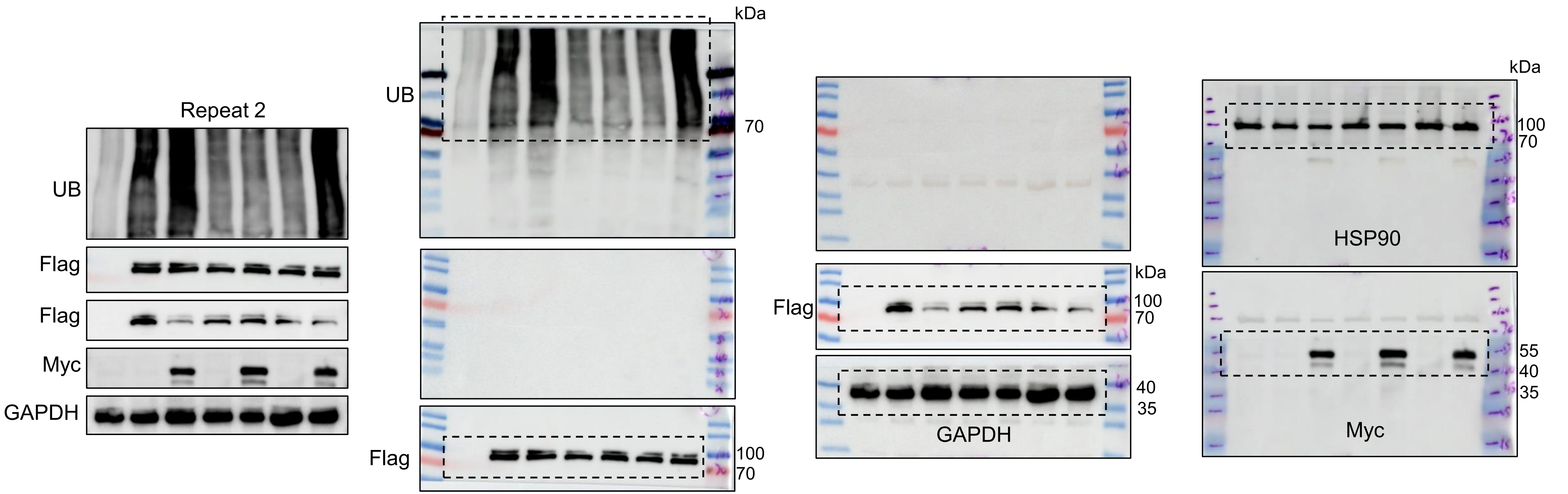
Repeat 2
UB
Flag
Flag
Myc
GAPDH
kDa
UB
70
Flag
100
70
Flag
kDa
100
70
40
35
GAPDH
kDa
100
70
HSP90
55
40
35
Myc

Figure 3M

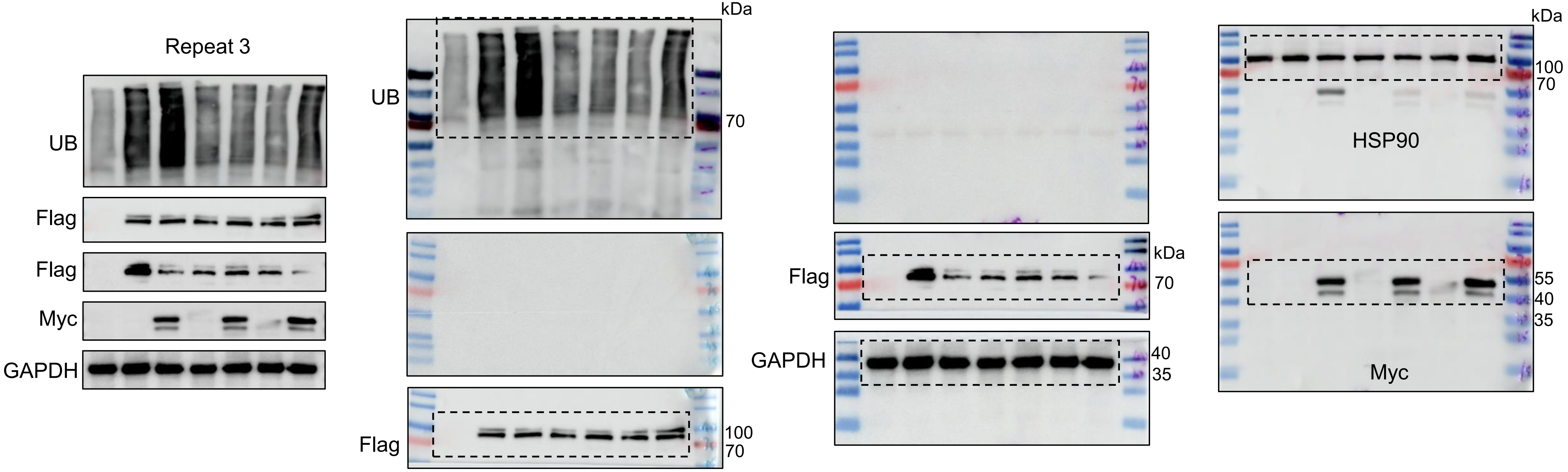

Repeat 3
UB
Flag
Flag
Myc
GAPDH
kDa
UB
70
Flag
100
70
Flag
kDa
70
GAPDH
40
35
kDa
100
70
HSP90
55
40
35
Myc

Figure 3N

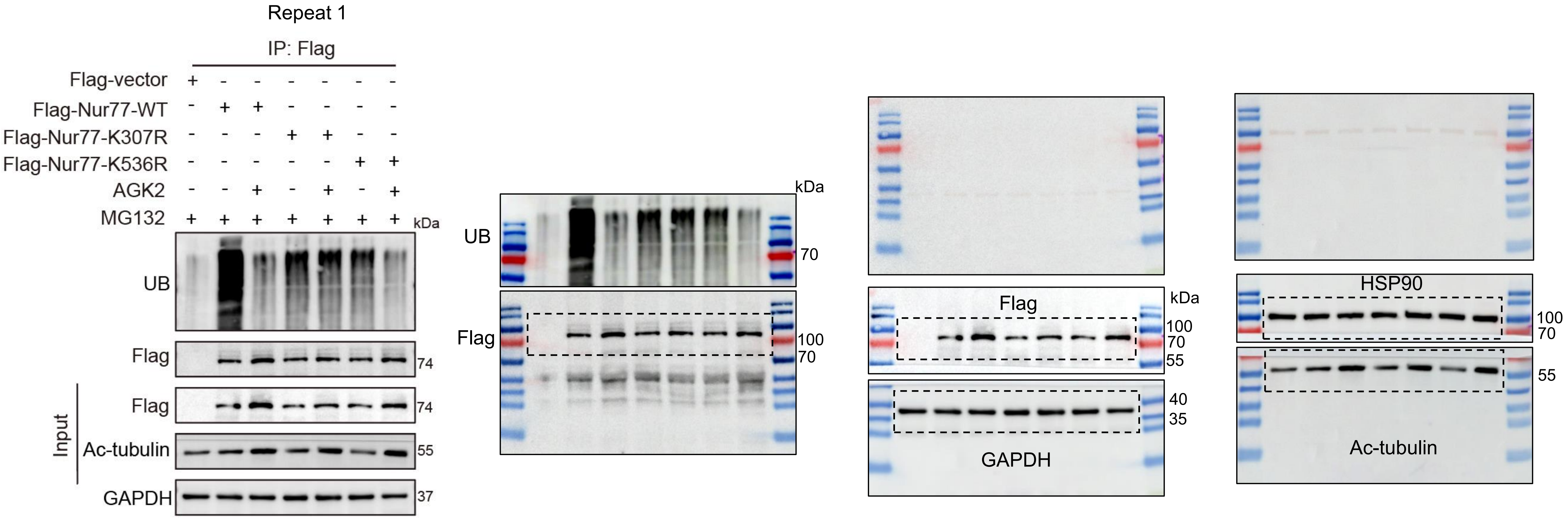

Repeat 1
IP: Flag
Flag-vector + - - - - - -
Flag-Nur77-WT - + + - - - -
Flag-Nur77-K307R - - - + + - -
Flag-Nur77-K536R - - - - - + +
AGK2 - - + - + - +
MG132 + + + + + + +
kDa
UB
Flag 74
Input
Flag 74
Ac-tubulin 55
GAPDH 37
kDa
UB 70
Flag 100 70
Flag
kDa
100 70 55
40 35
GAPDH
HSP90
100 70
55
Ac-tubulin

Figure 3N

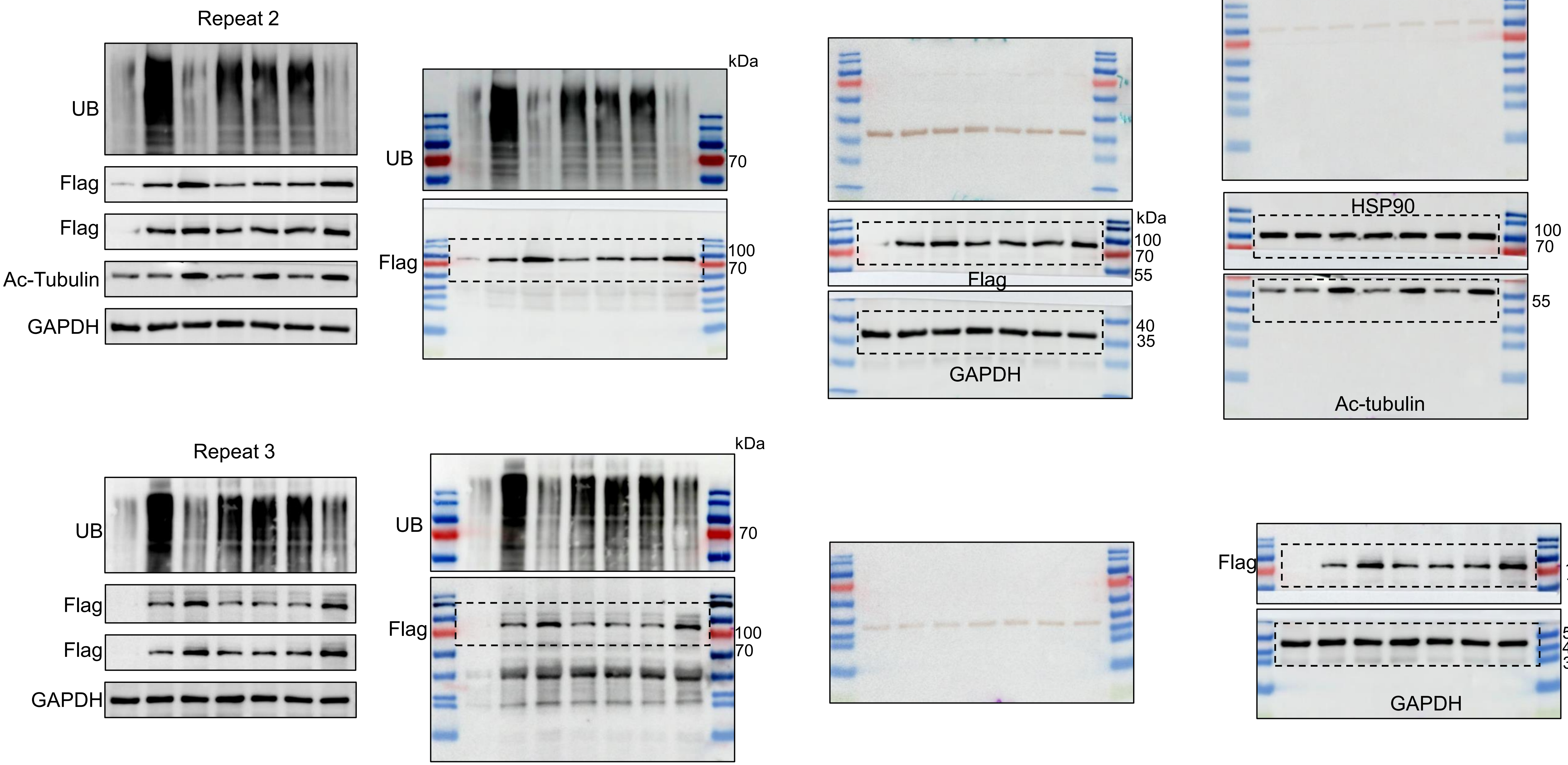
Repeat 2
UB
Flag
Flag
Ac-Tubulin
GAPDH
kDa
UB
70
Flag
100
70
kDa
100
70
55
Flag
40
35
GAPDH
HSP90
100
70
55
Ac-tubulin
Repeat 3
UB
Flag
Flag
GAPDH
kDa
UB
70
Flag
100
70
Flag
100
70
55
40
35
GAPDH

Figure 3O

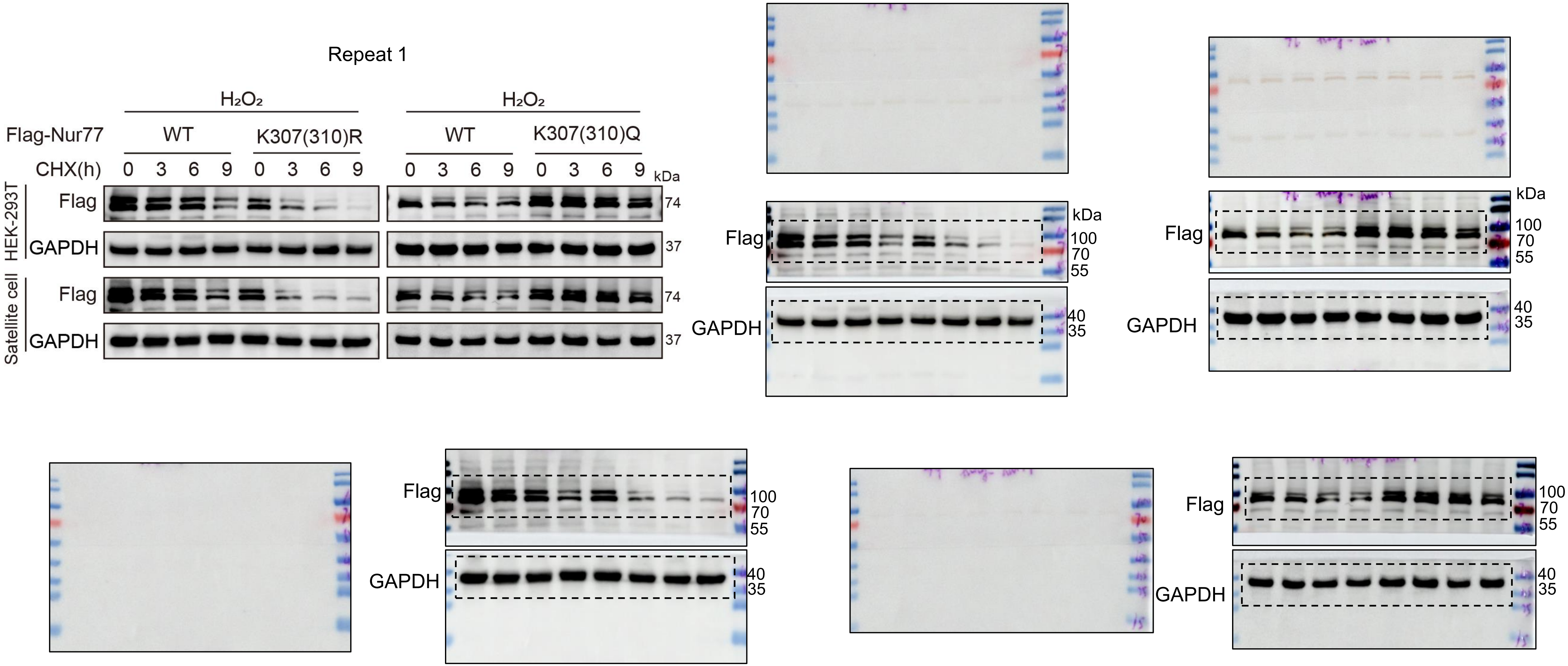
Repeat 1
H2O2
H2O2
Flag-Nur77
WT
K307(310)R
WT
K307(310)Q
CHX(h)
0 3 6 9 0 3 6 9
0 3 6 9 0 3 6 9
kDa
HEK-293T
Flag
74
GAPDH
37
Satellite cell
Flag
74
GAPDH
37
Flag
kDa
100
70
55
GAPDH
40
35
Flag
kDa
100
70
55
GAPDH
40
35
Flag
100
70
55
GAPDH
40
35
Flag
100
70
55
GAPDH
40
35

Figure 3O

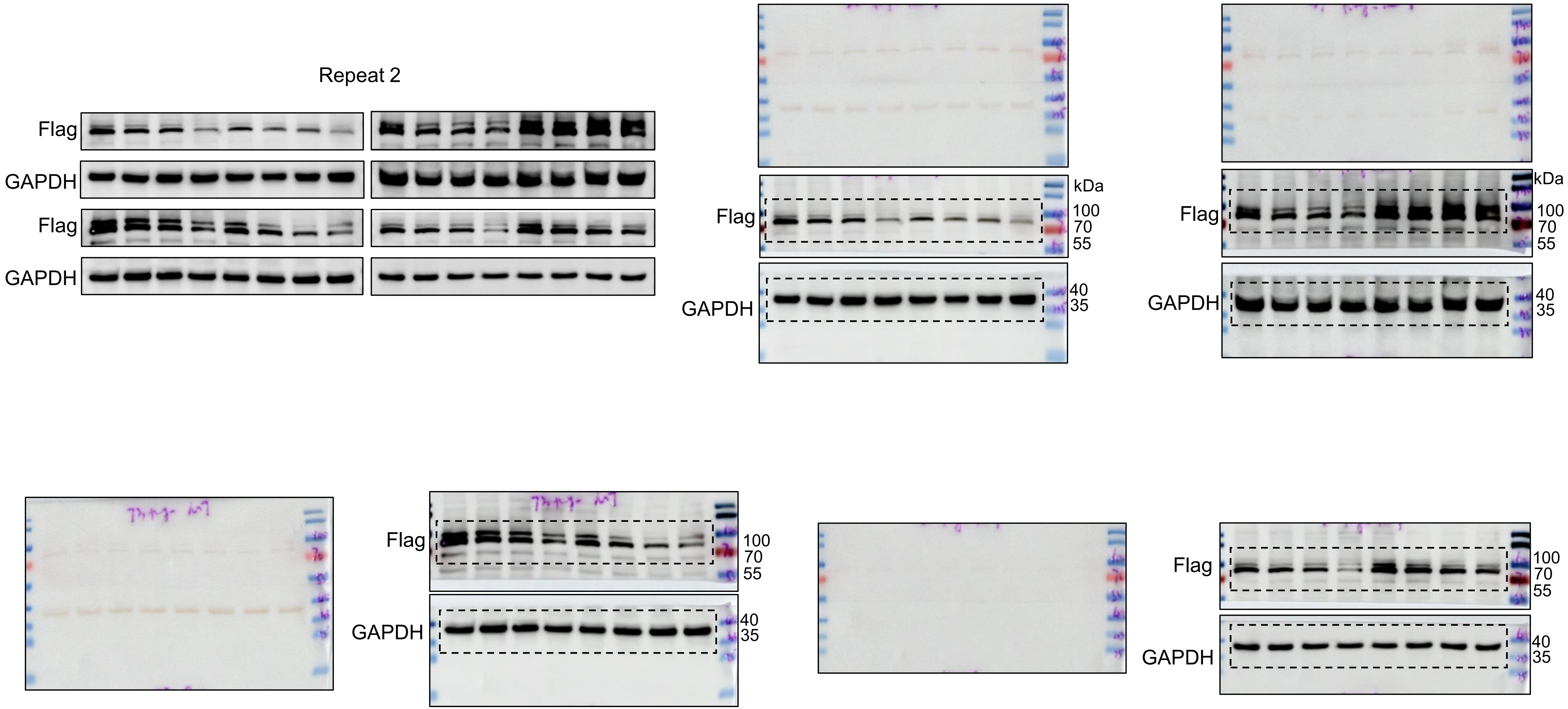
Repeat 2
Flag
GAPDH
Flag
GAPDH
kDa
Flag
100
70
55
GAPDH
40
35
kDa
Flag
100
70
55
GAPDH
40
35
Flag
100
70
55
GAPDH
40
35
Flag
100
70
55
GAPDH
40
35

Figure 3O

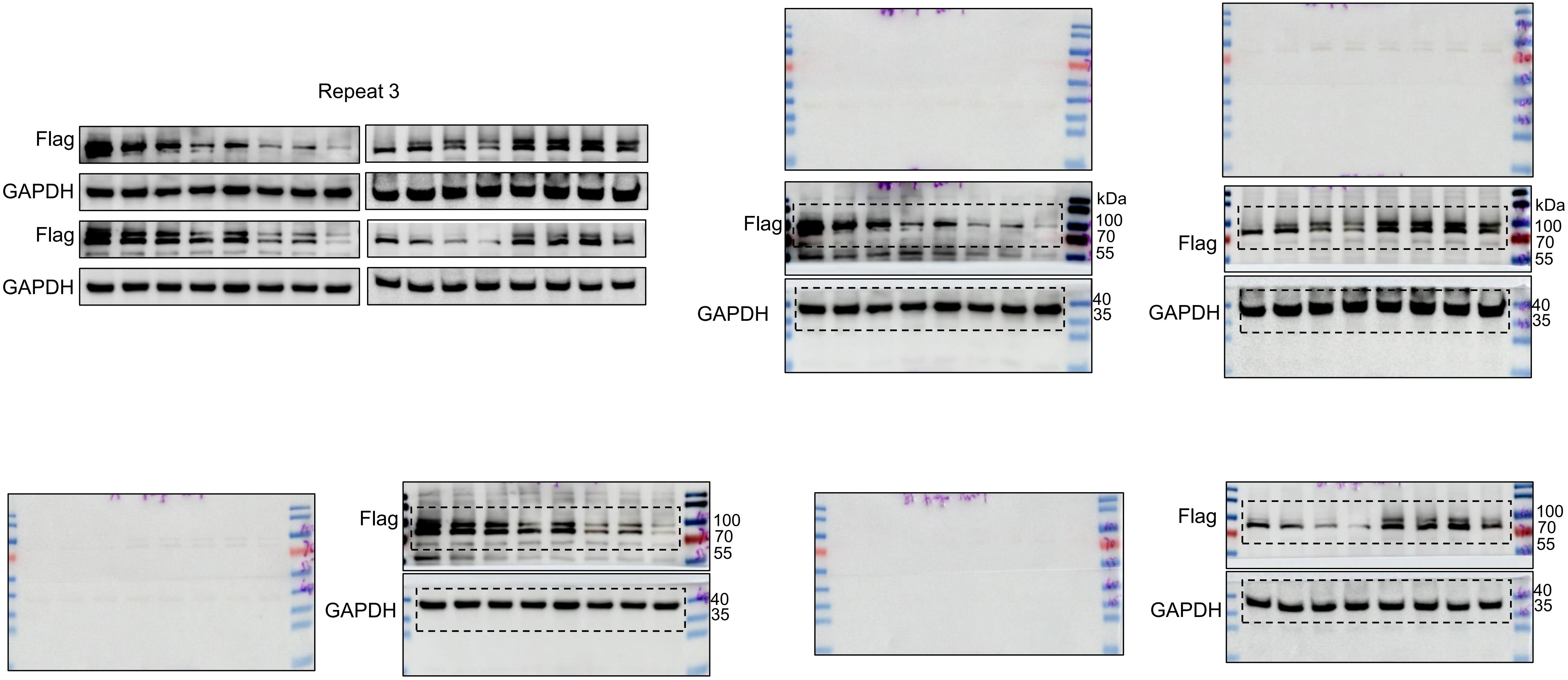

Figure 4G

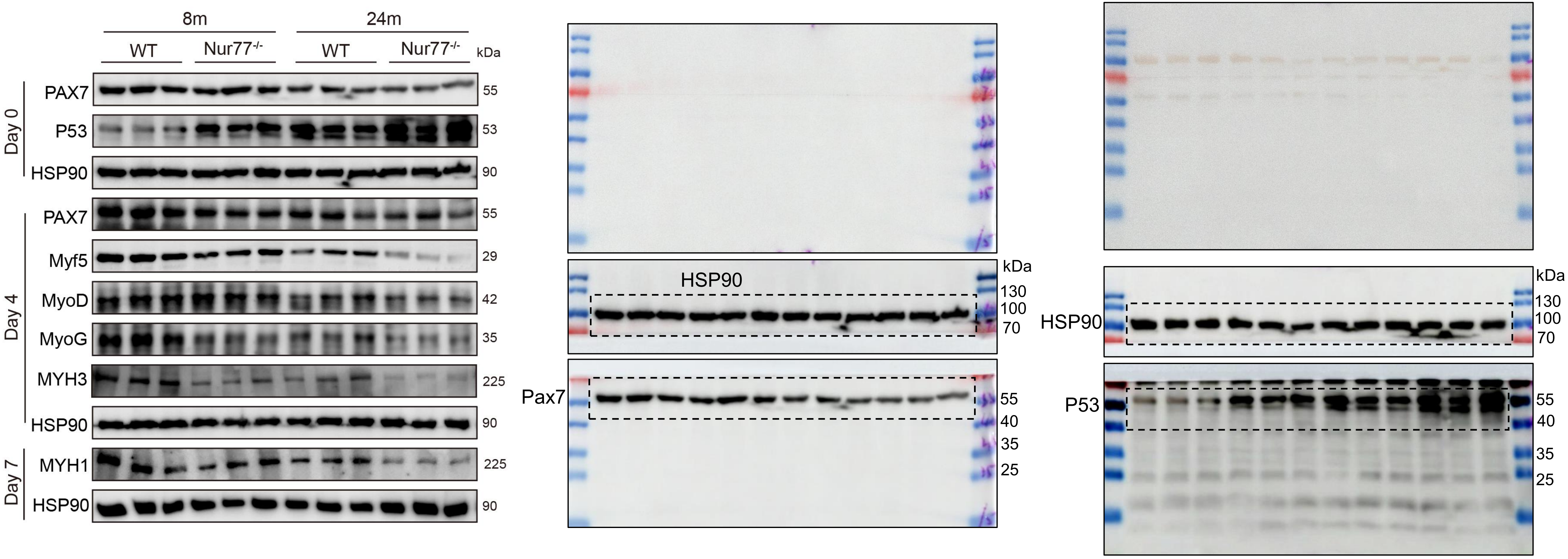

8m
24m
WT
Nur77-/-
WT
Nur77-/-
kDa
Day 0
PAX7
55
P53
53
HSP90
90
Day 4
PAX7
55
Myf5
29
MyoD
42
MyoG
35
MYH3
225
HSP90
90
Day 7
MYH1
225
HSP90
90
kDa
HSP90
130
100
70
Pax7
55
40
35
25
kDa
HSP90
130
100
70
P53
55
40
35
25

Figure 4G

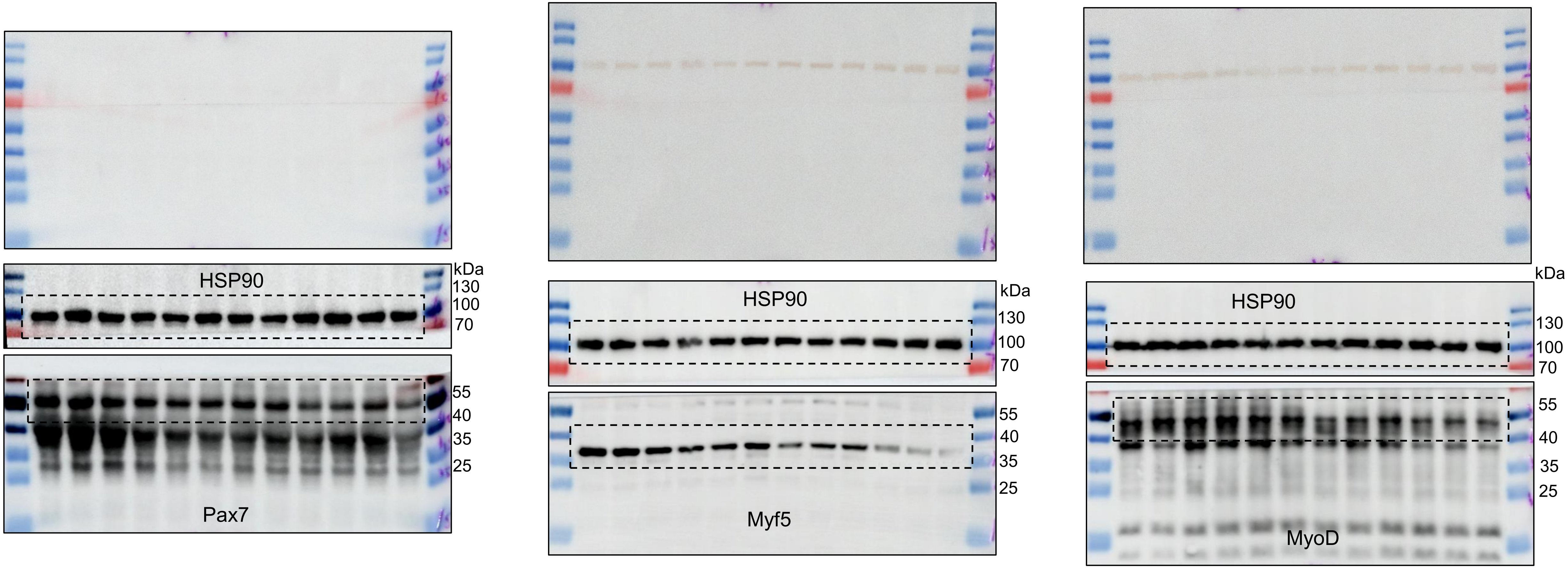
HSP90
kDa
130
100
70
55
40
35
25
Pax7
HSP90
kDa
130
100
70
55
40
35
25
Myf5
kDa
HSP90
130
100
70
55
40
35
25
MyoD

Figure 4G

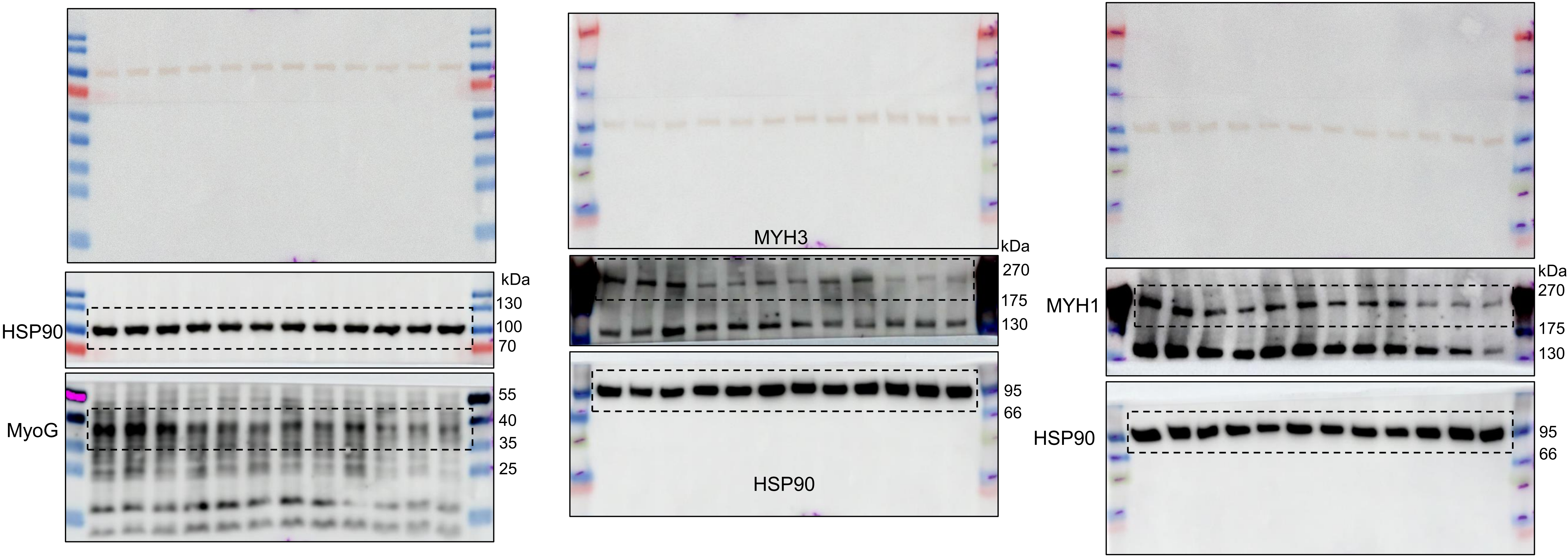

kDa
130
HSP90
100
70
55
MyoG
40
35
25
MYH3
kDa
270
175
130
95
66
HSP90
kDa
270
MYH1
175
130
HSP90
95
66

Figure 5C

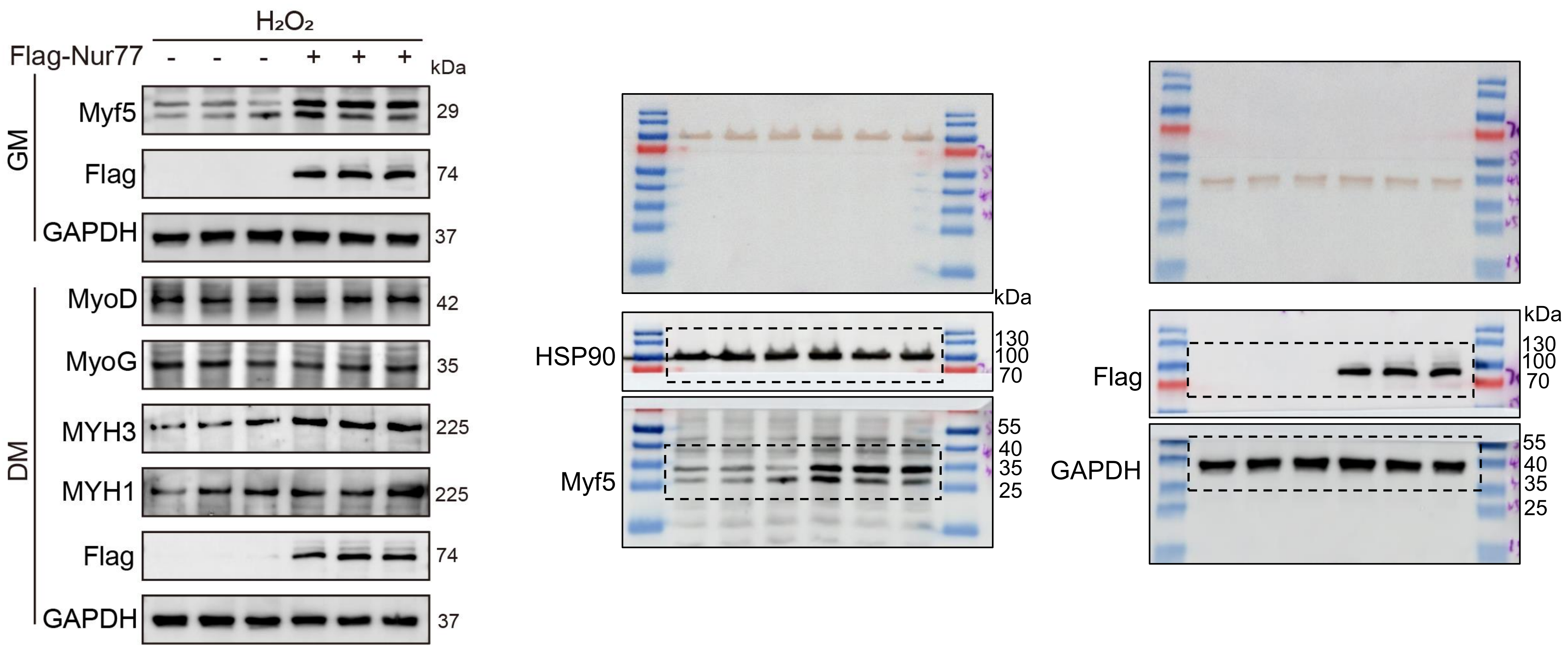
H2O2
Flag-Nur77 - - - + + +
kDa
GM
Myf5 29
Flag 74
GAPDH 37
DM
MyoD 42
MyoG 35
MYH3 225
MYH1 225
Flag 74
GAPDH 37
kDa
HSP90
130
100
70
Myf5
55
40
35
25
kDa
Flag
130
100
70
GAPDH
55
40
35
25

Figure 5C

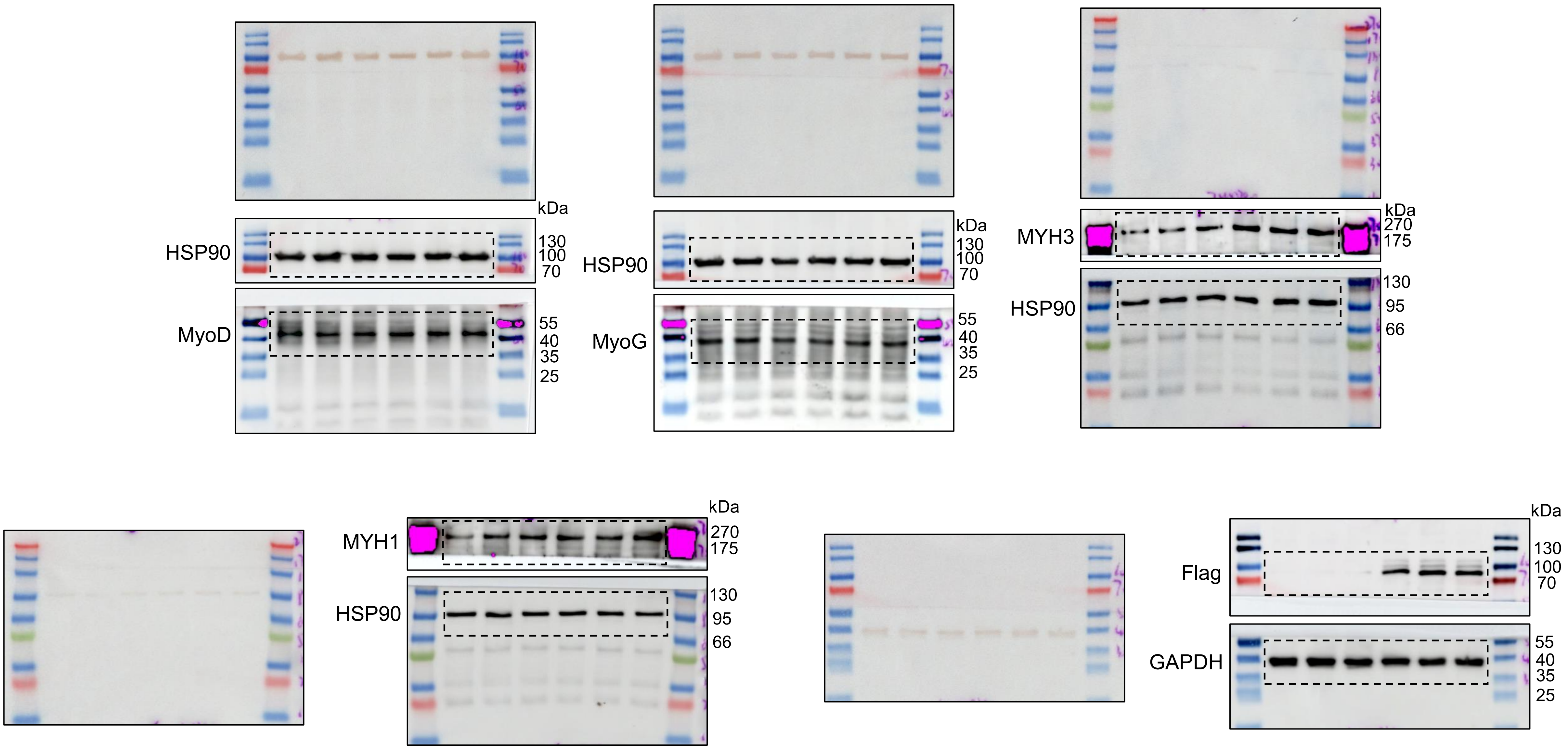
kDa
HSP90
130
100
70
MyoD
55
40
35
25
kDa
HSP90
130
100
70
MyoG
55
40
35
25
MYH3
kDa
270
175
HSP90
130
95
66
kDa
MYH1
270
175
HSP90
130
95
66
kDa
Flag
130
100
70
GAPDH
55
40
35
25

Figure 5D

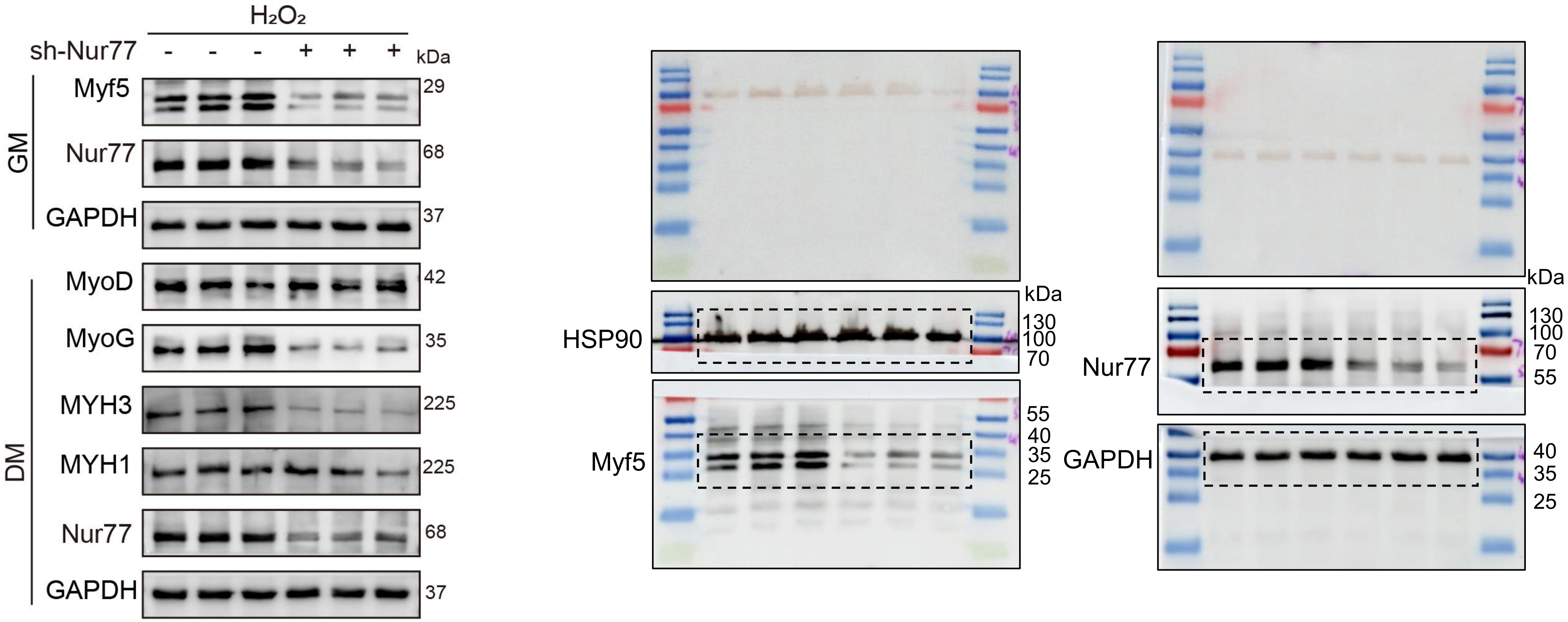
H2O2
sh-Nur77 - - - + + +
kDa
GM
Myf5 29
Nur77 68
GAPDH 37
DM
MyoD 42
MyoG 35
MYH3 225
MYH1 225
Nur77 68
GAPDH 37
HSP90
130
100
70
Myf5
55
40
35
25
Nur77
130
100
70
55
GAPDH
40
35
25

Figure 5D

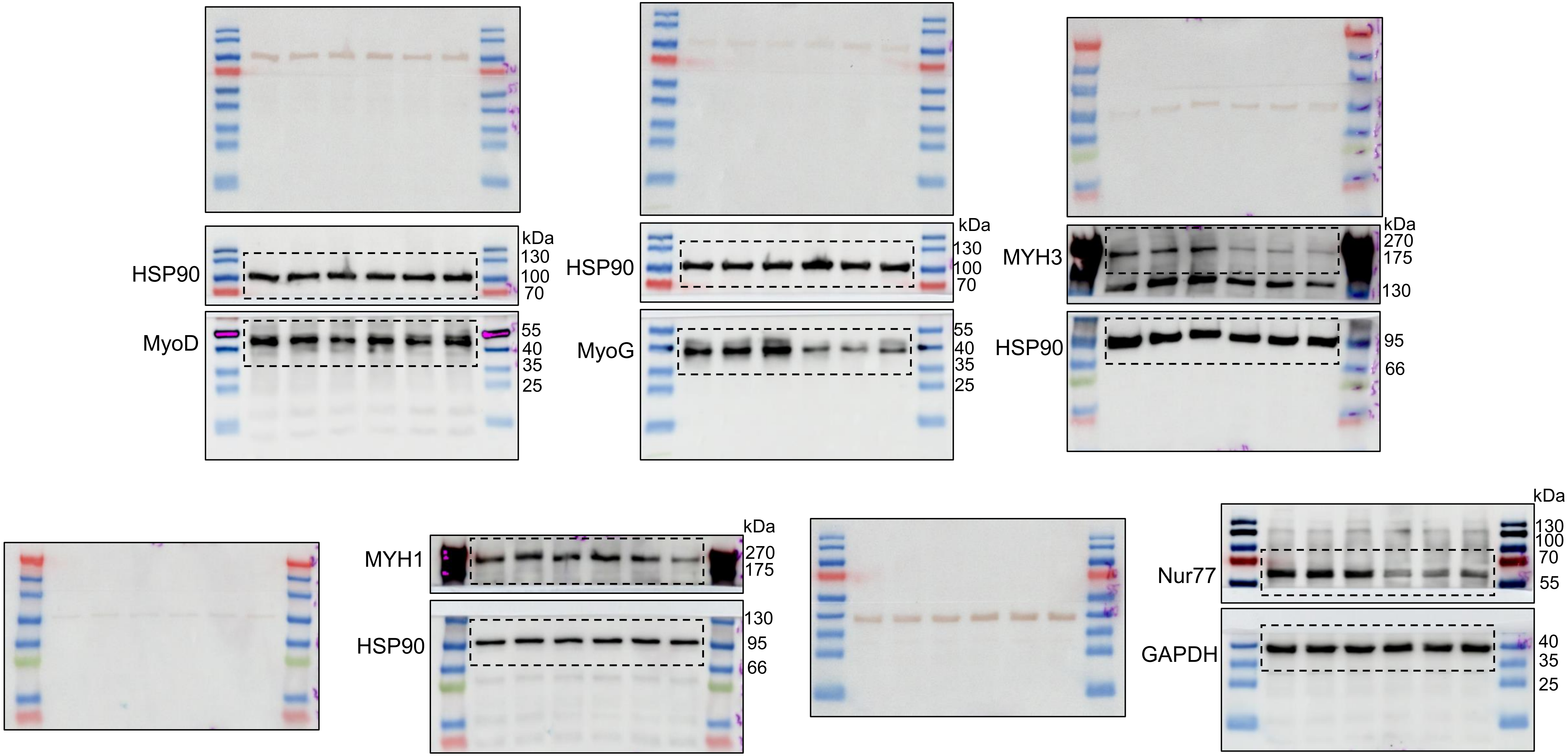

HSP90
kDa
130
100
70
MyoD
55
40
35
25
HSP90
kDa
130
100
70
MyoG
55
40
35
25
MYH3
kDa
270
175
130
HSP90
95
66
MYH1
kDa
270
175
HSP90
130
95
66
Nur77
kDa
130
100
70
55
GAPDH
40
35
25

Figure 5M

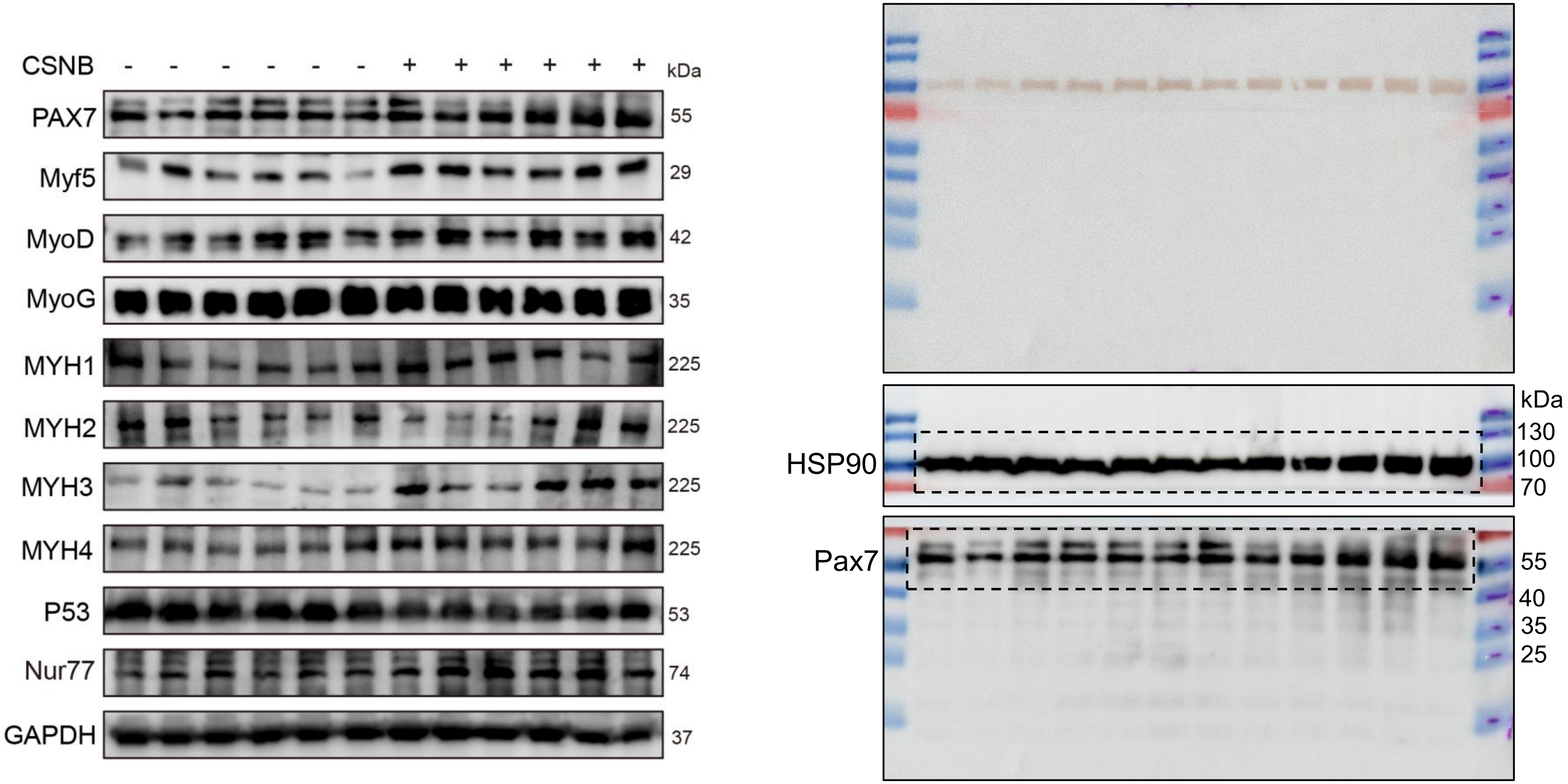

CSNB - - - - - - + + + + + +
kDa
PAX7 55
Myf5 29
MyoD 42
MyoG 35
MYH1 225
MYH2 225
MYH3 225
MYH4 225
P53 53
Nur77 74
GAPDH 37
kDa
HSP90
130
100
70
Pax7
55
40
35
25

Figure 5M

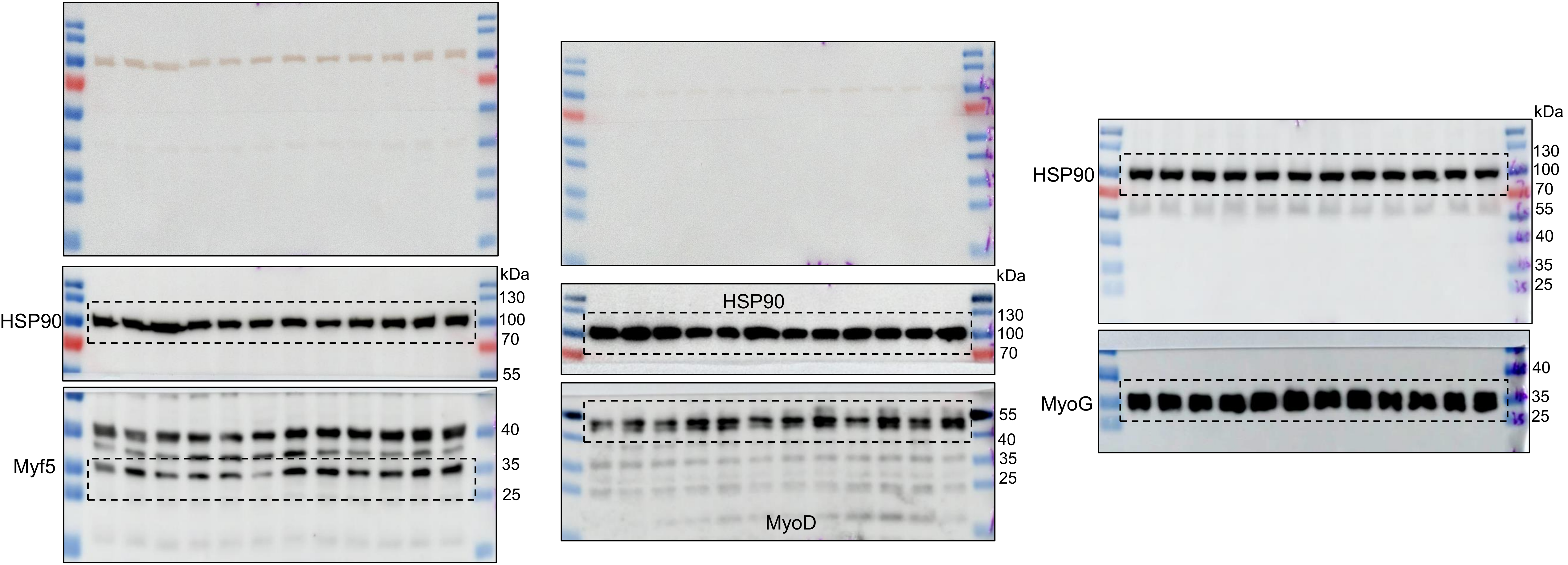
HSP90
kDa
130
100
70
55
Myf5
40
35
25
HSP90
kDa
130
100
70
MyoD
55
40
35
25
HSP90
kDa
130
100
70
55
40
35
25
MyoG
40
35
25

Figure 5M

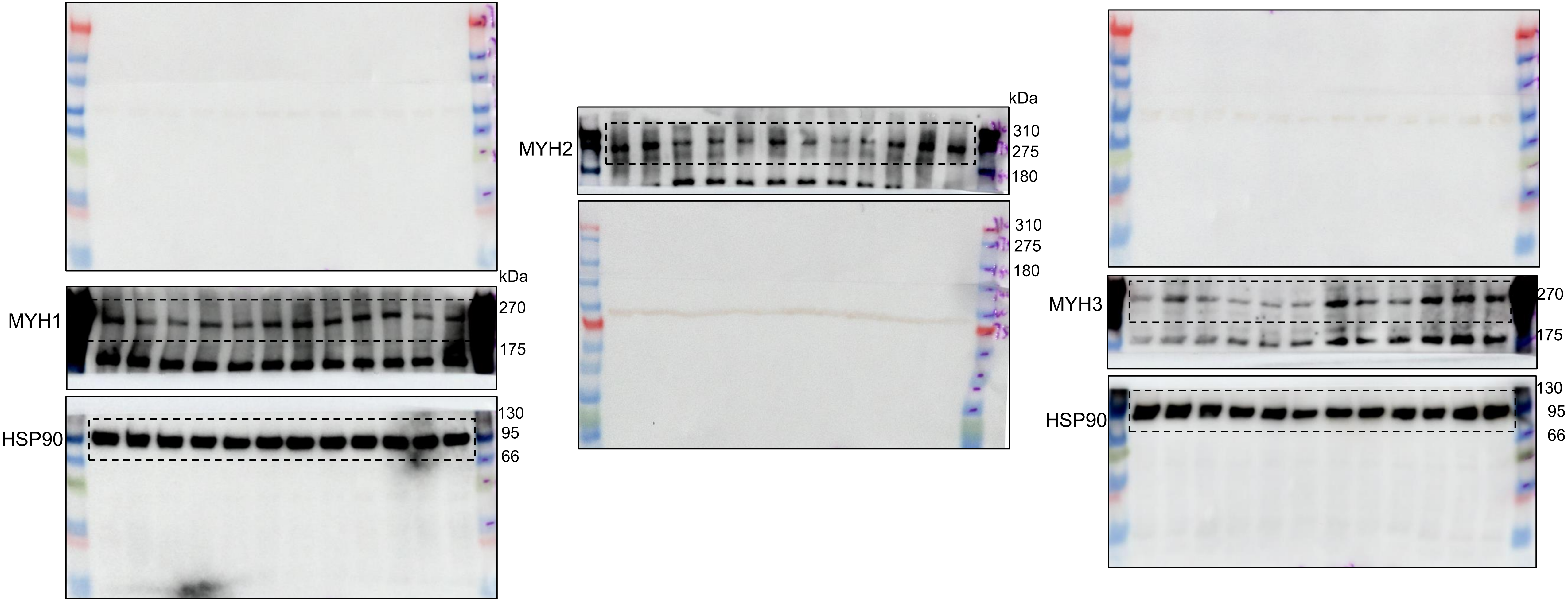

kDa
MYH2
310
275
180
310
275
180
kDa
MYH1
270
175
HSP90
130
95
66
MYH3
270
175
HSP90
130
95
66

Figure 5M

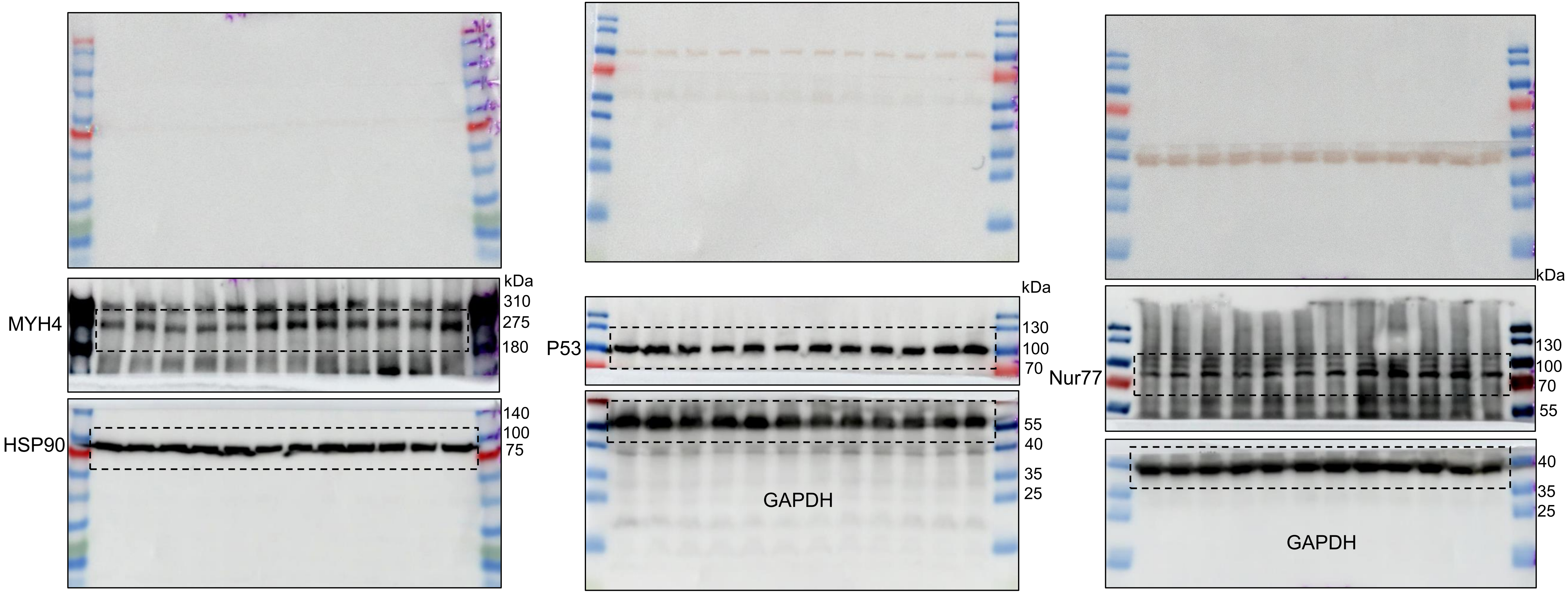

MYH4
kDa
310
275
180
HSP90
140
100
75
P53
kDa
130
100
70
55
40
35
25
GAPDH
Nur77
kDa
130
100
70
55
40
35
25
GAPDH

Figure 6G

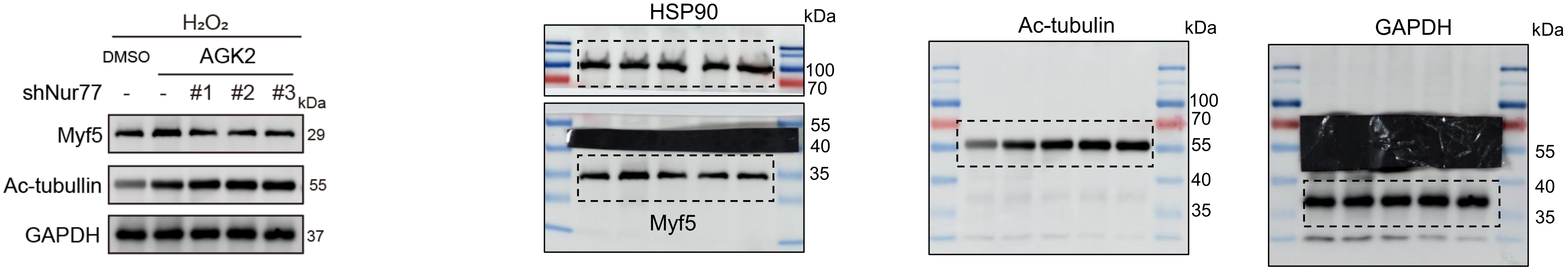

Figure 6L

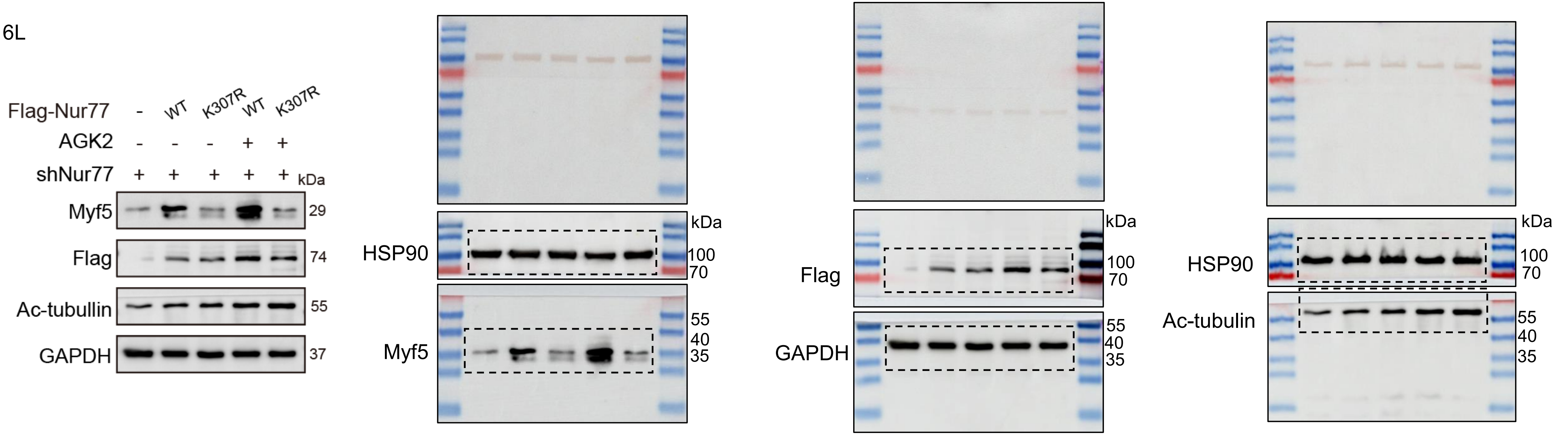

Figure 6M

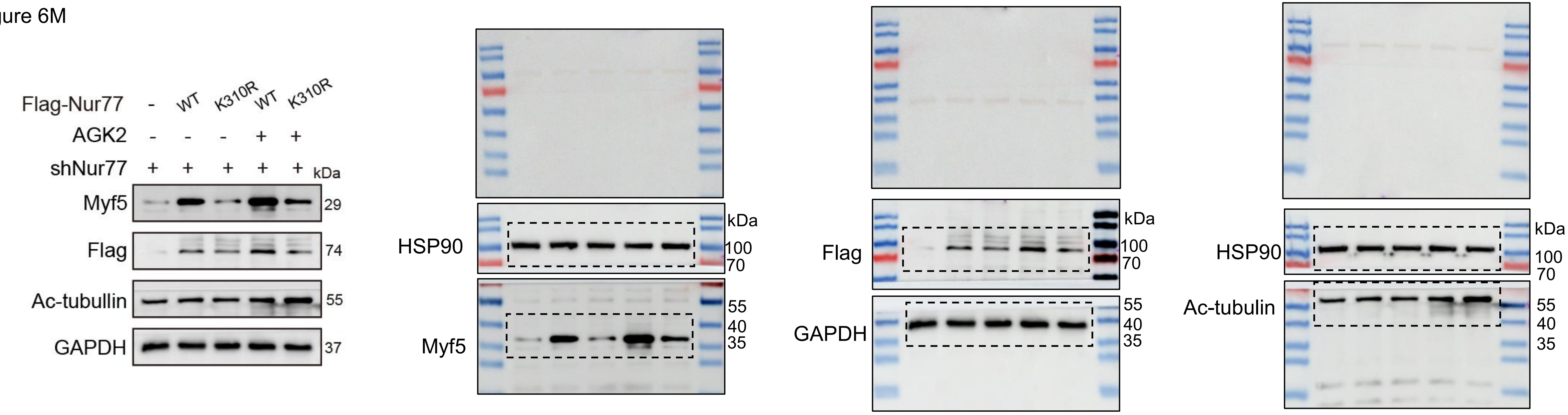

Figure 6P

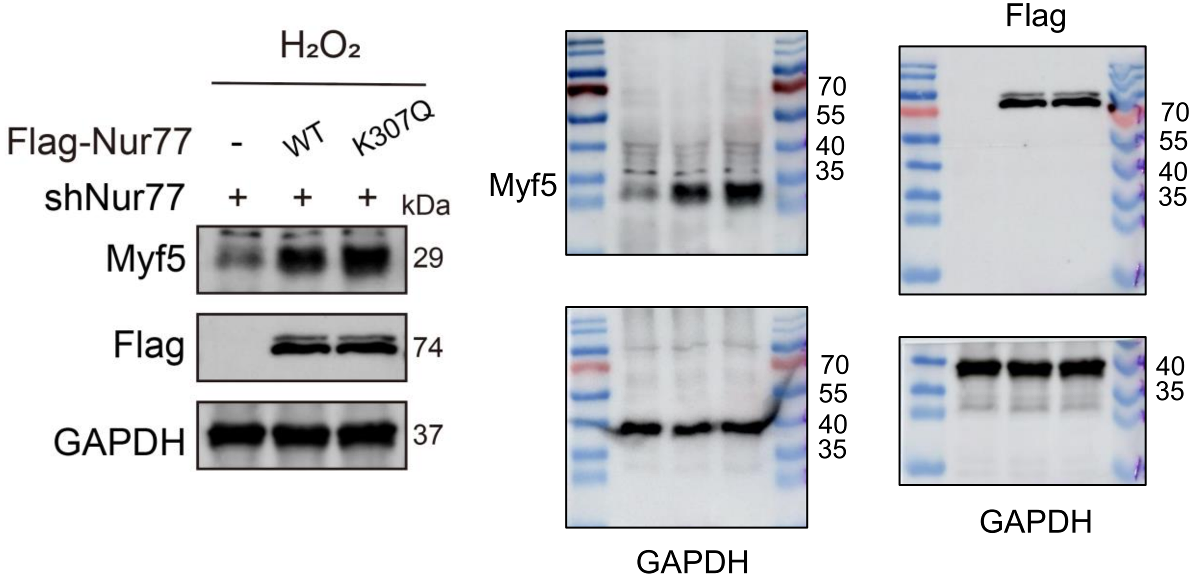

Figure 6Q

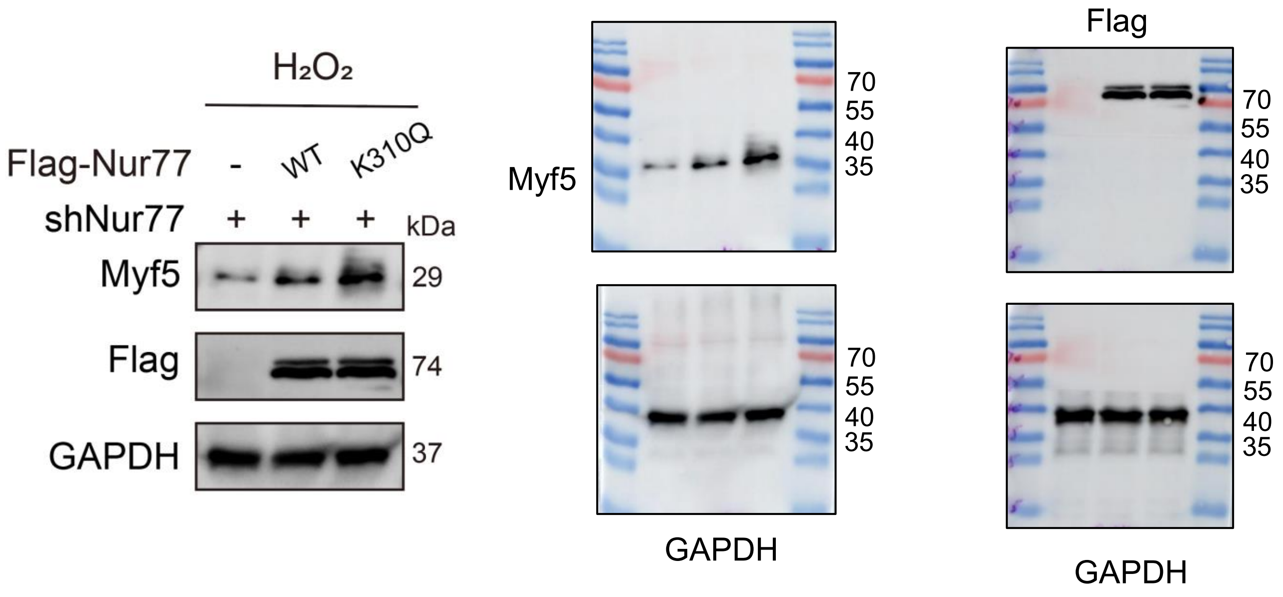

Figure 7N

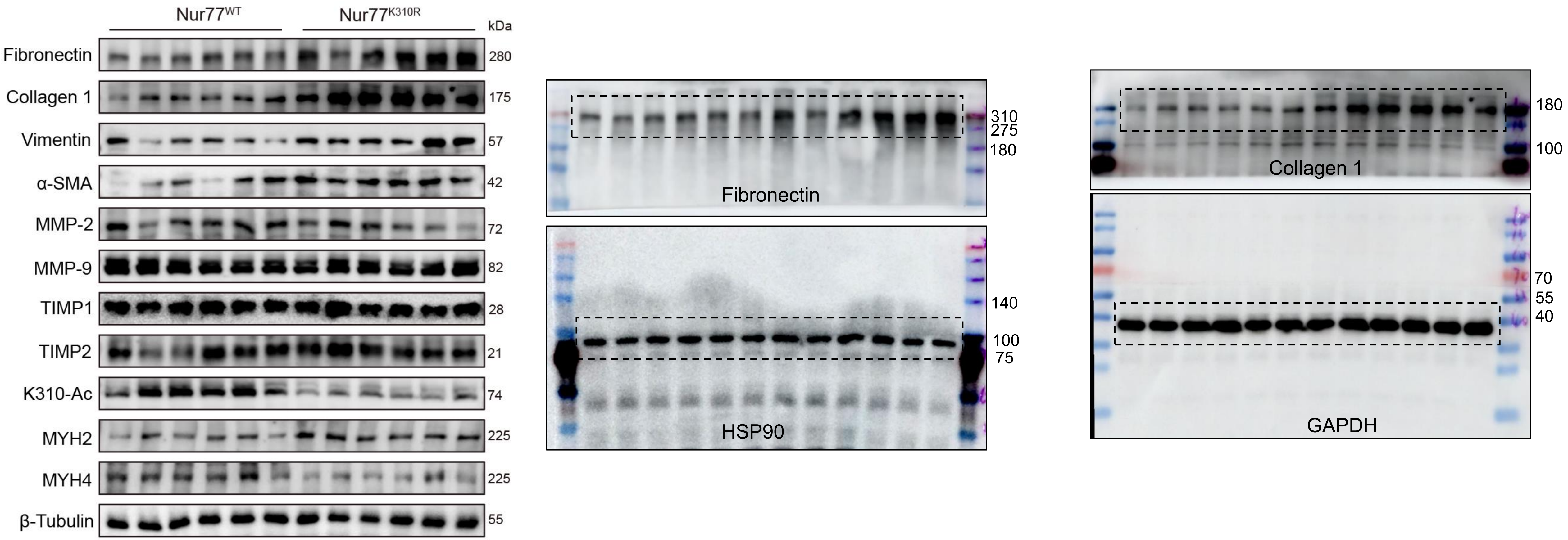

Nur77^{WT}
Nur77^{K310R}
kDa
Fibronectin 280
Collagen 1 175
Vimentin 57
α-SMA 42
MMP-2 72
MMP-9 82
TIMP1 28
TIMP2 21
K310-Ac 74
MYH2 225
MYH4 225
β-Tubulin 55
310
275
180
Fibronectin
140
100
75
HSP90
180
100
Collagen 1
70
55
40
GAPDH

Figure 7N

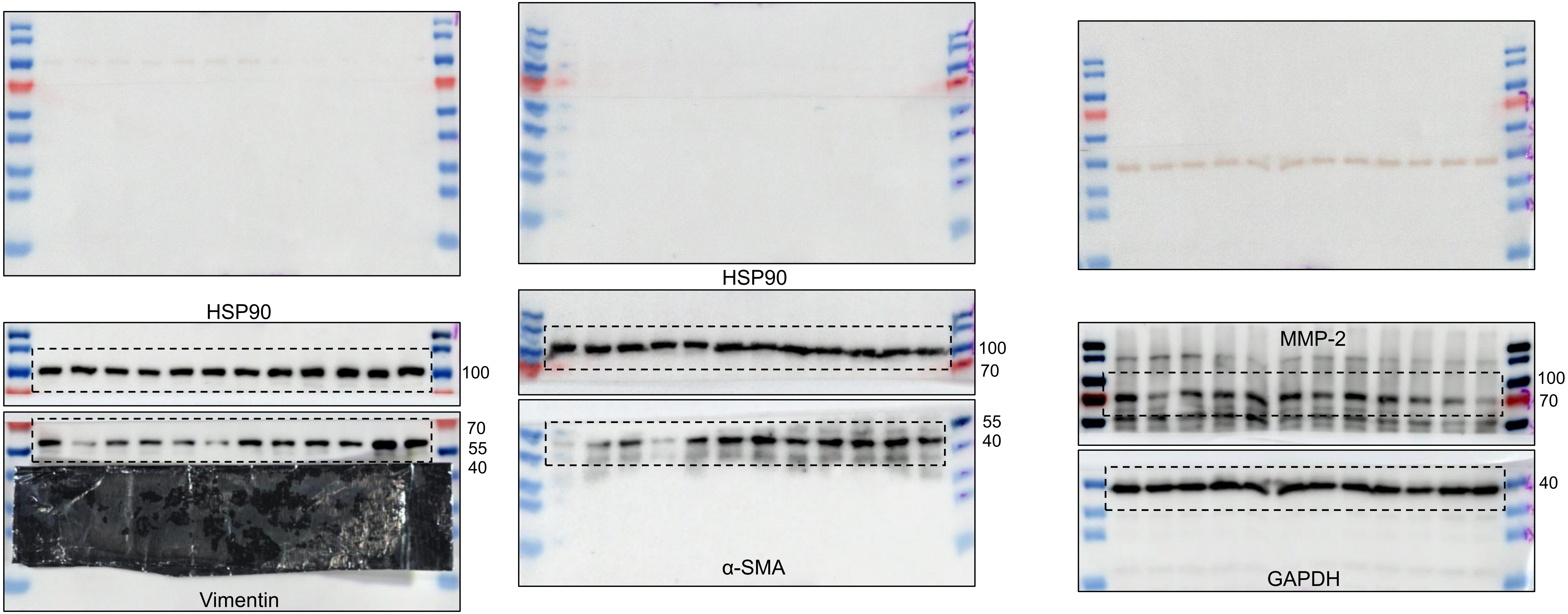
HSP90
100
70
55
40
Vimentin
HSP90
100
70
55
40
α-SMA
MMP-2
100
70
40
GAPDH

Figure 7N

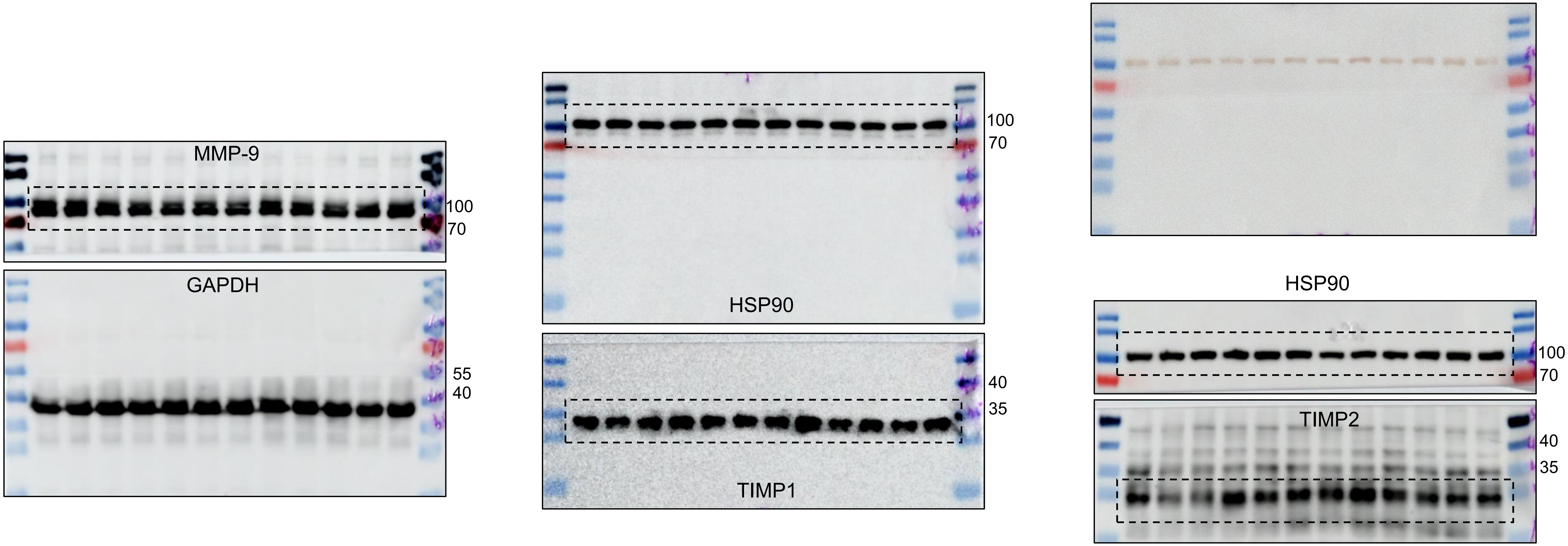
MMP-9
100
70
GAPDH
55
40
100
70
HSP90
40
35
TIMP1
HSP90
100
70
TIMP2
40
35

Figure 7N

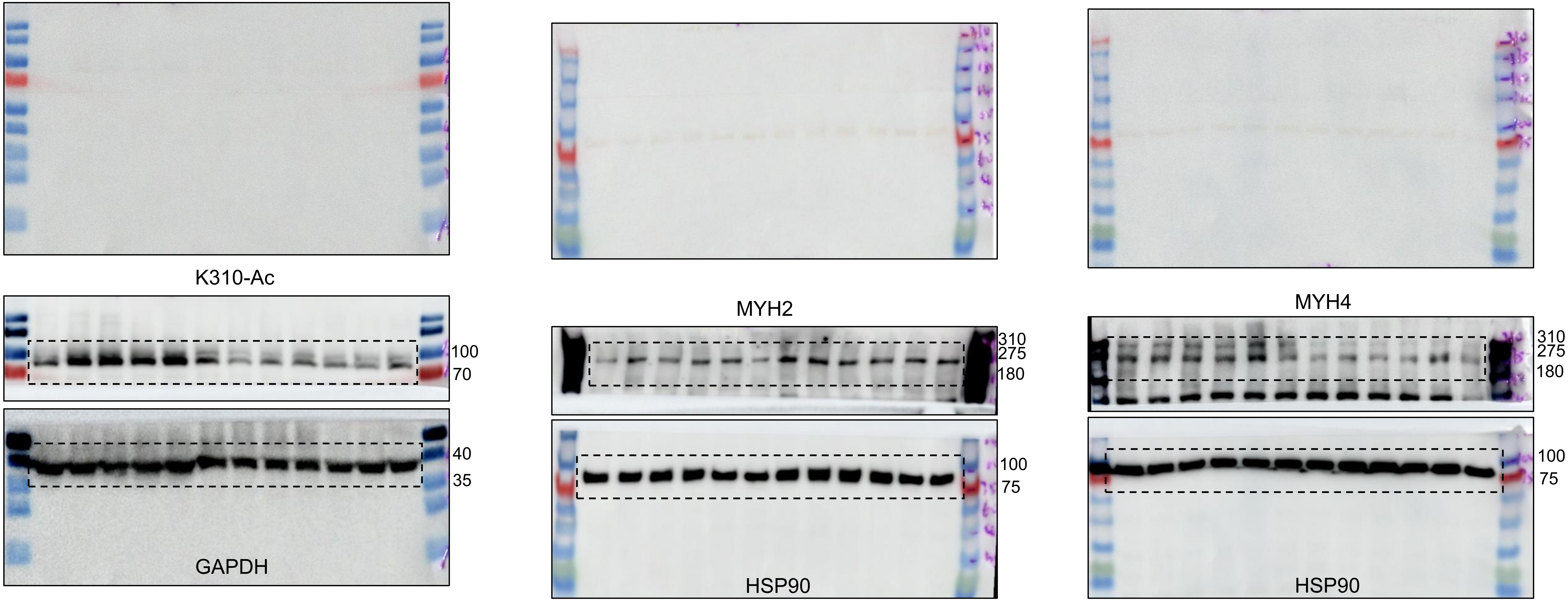

K310-Ac
100
70
40
35
GAPDH
MYH2
310
275
180
100
75
HSP90
MYH4
310
275
180
100
75
HSP90

Figure 8Q

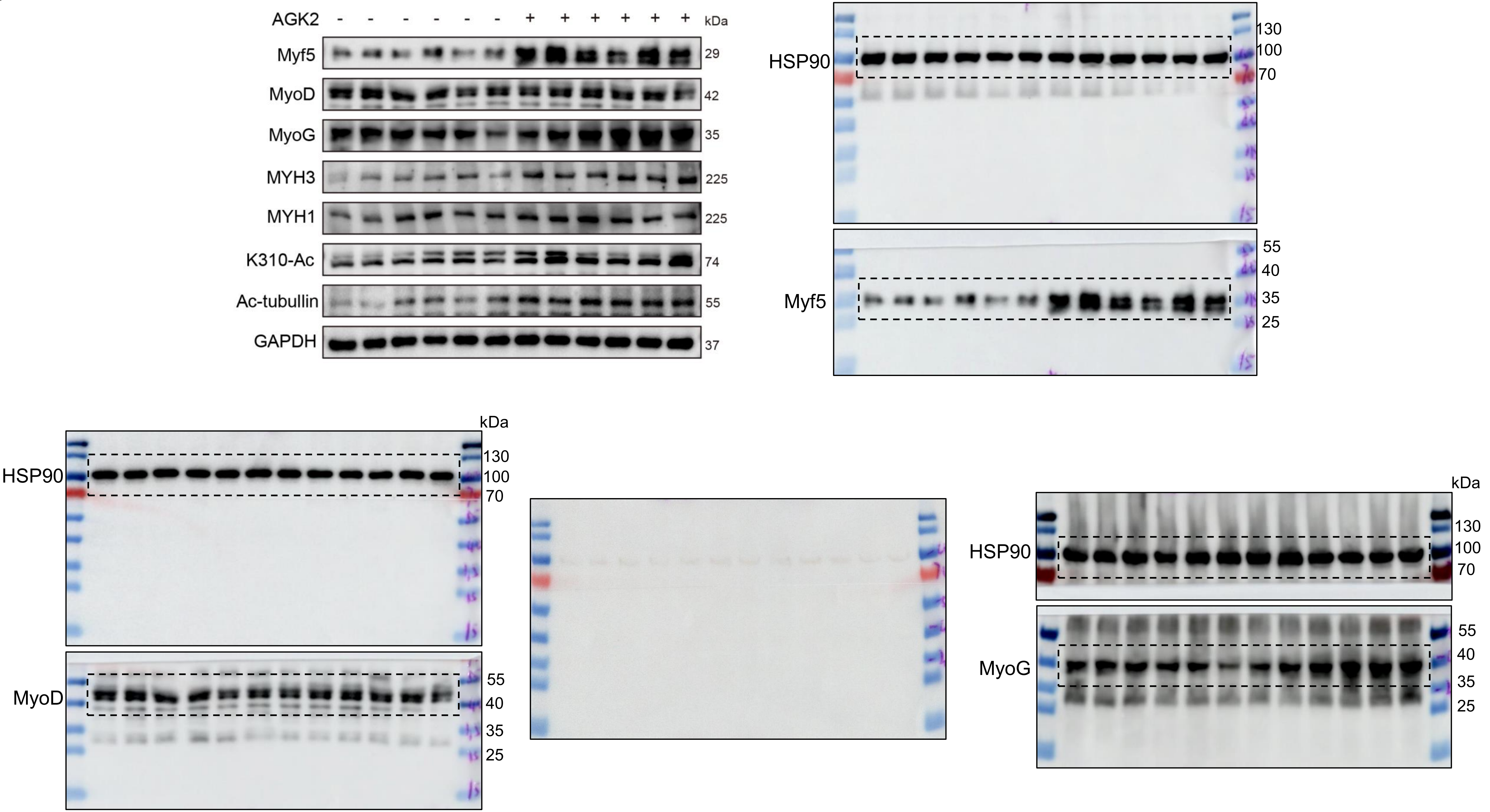
AGK2 - - - - - - + + + + + +
kDa
Myf5 29
MyoD 42
MyoG 35
MYH3 225
MYH1 225
K310-Ac 74
Ac-tubullin 55
GAPDH 37
kDa
HSP90
130
100
70
Myf5
55
40
35
25
kDa
HSP90
130
100
70
MyoD
55
40
35
25
kDa
HSP90
130
100
70
MyoG
55
40
35
25

Figure 8Q

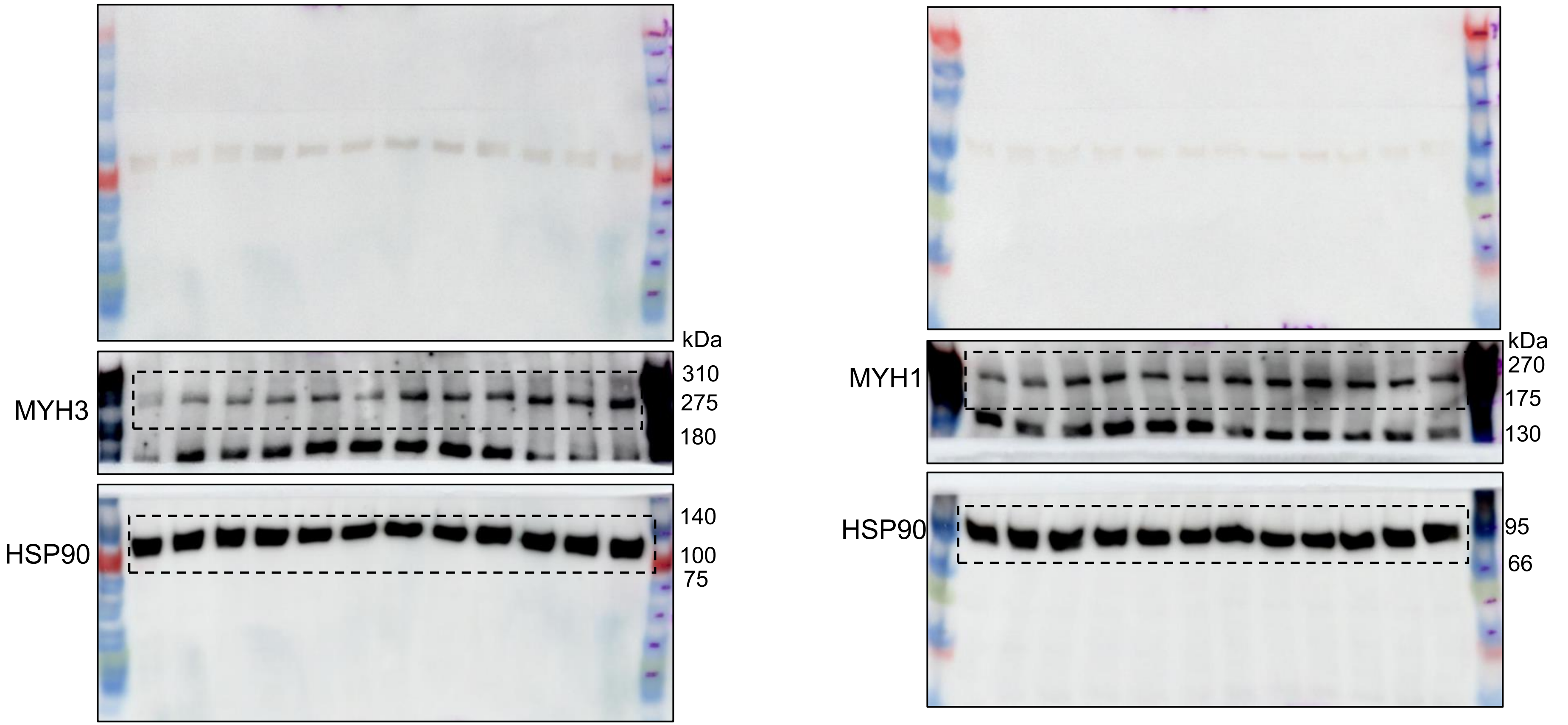
kDa
MYH3
310
275
180
HSP90
140
100
75
kDa
MYH1
270
175
130
HSP90
95
66

Figure 8Q

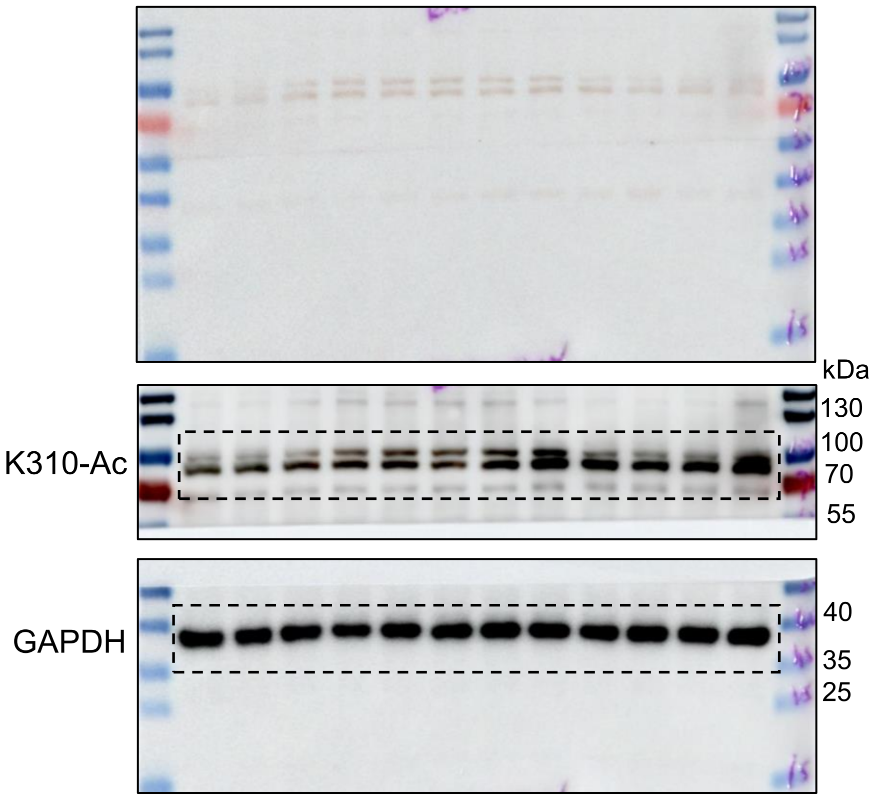
kDa
K310-Ac
130
100
70
55
GAPDH
40
35
25

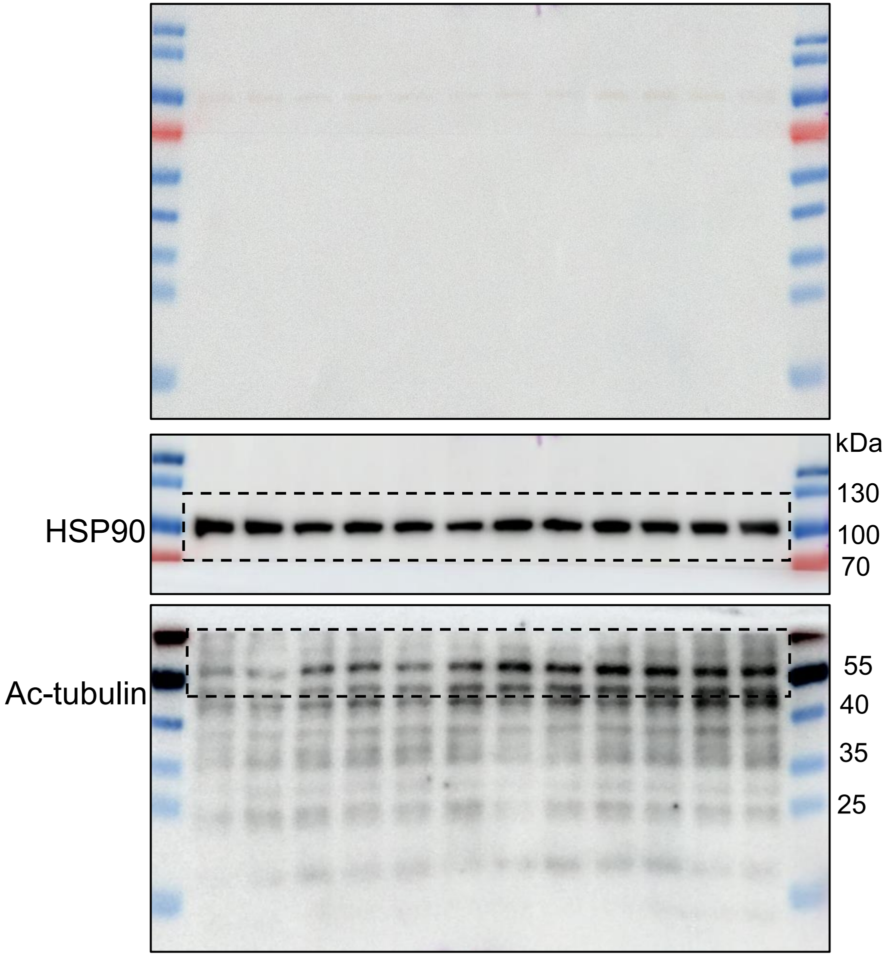
kDa
HSP90
130
100
70
Ac-tubulin
55
40
35
25

Figure S1A

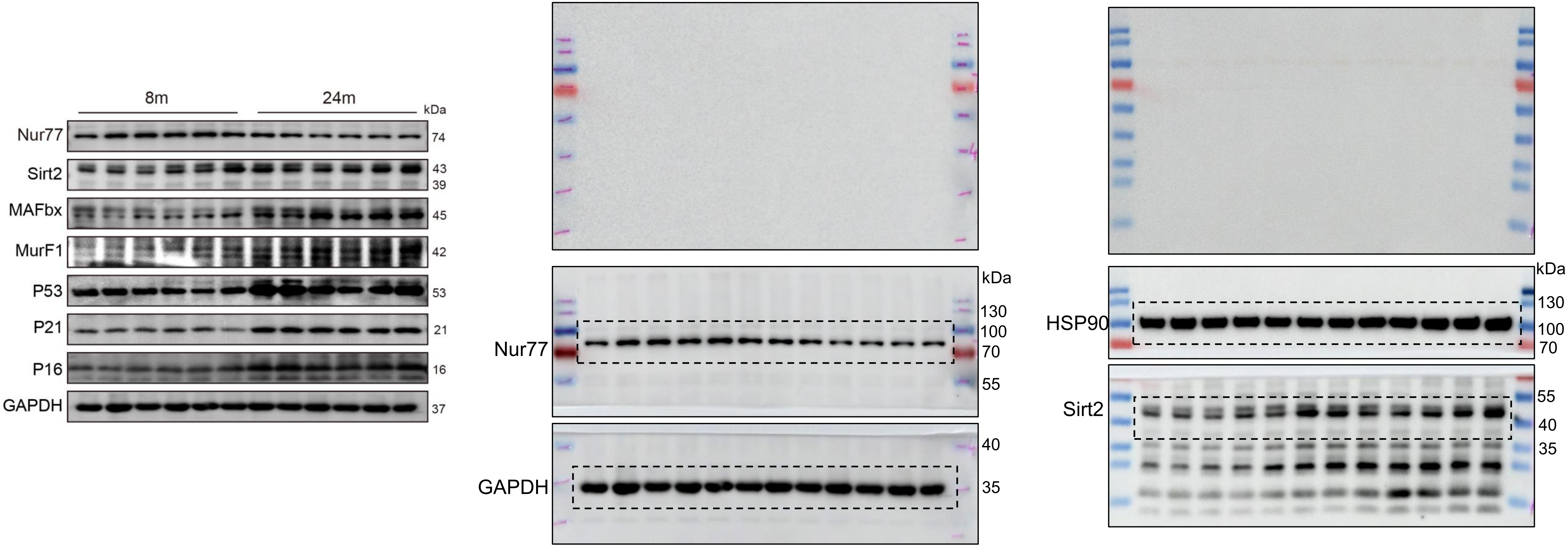

8m
24m
kDa
Nur77
74
Sirt2
43
39
MAFbx
45
MurF1
42
P53
53
P21
21
P16
16
GAPDH
37
kDa
130
100
Nur77
70
55
40
GAPDH
35
kDa
130
HSP90
100
70
55
Sirt2
40
35

Figure S1A

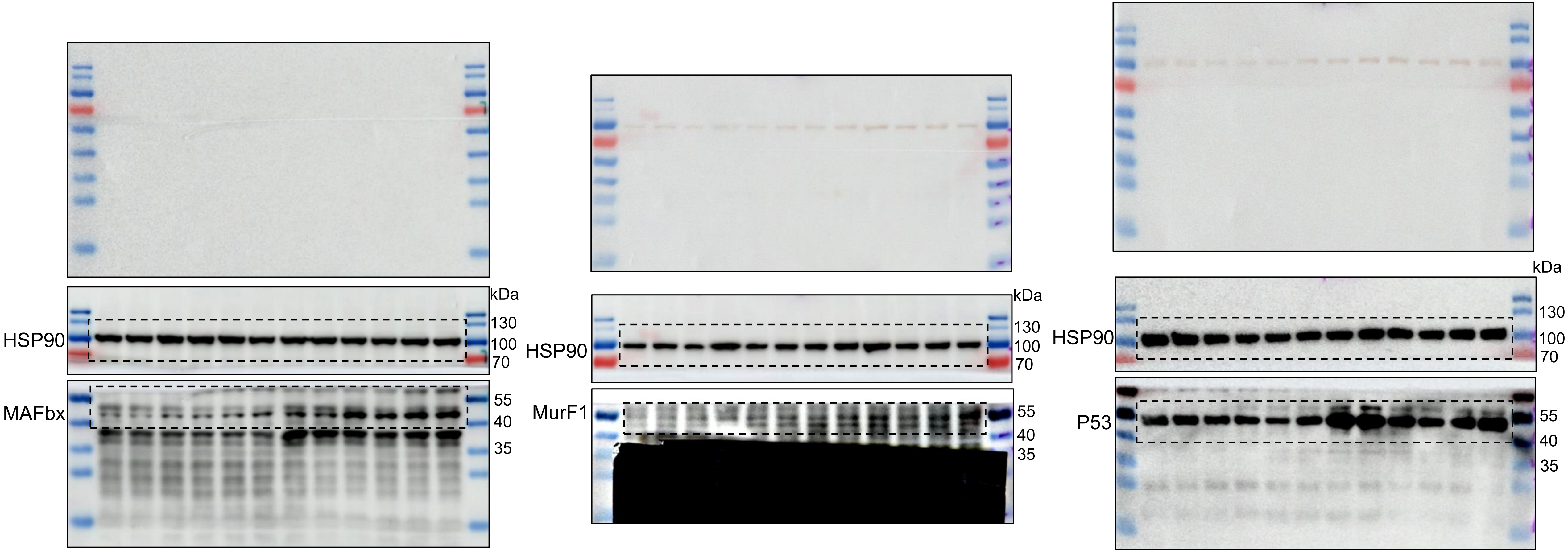

HSP90
MAFbx
kDa
130
100
70
55
40
35
HSP90
MurF1
kDa
130
100
70
55
40
35
HSP90
P53
kDa
130
100
70
55
40
35

Figure S1A

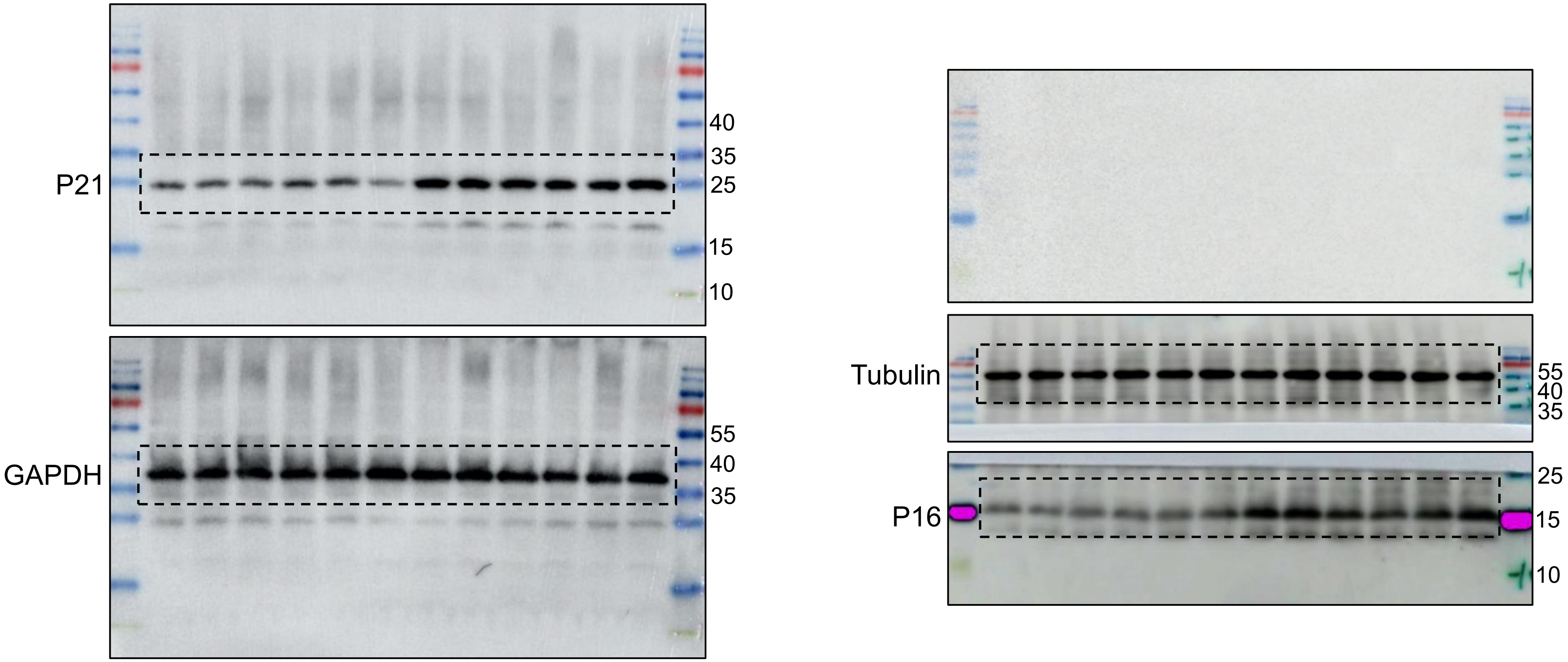

Figure S1B

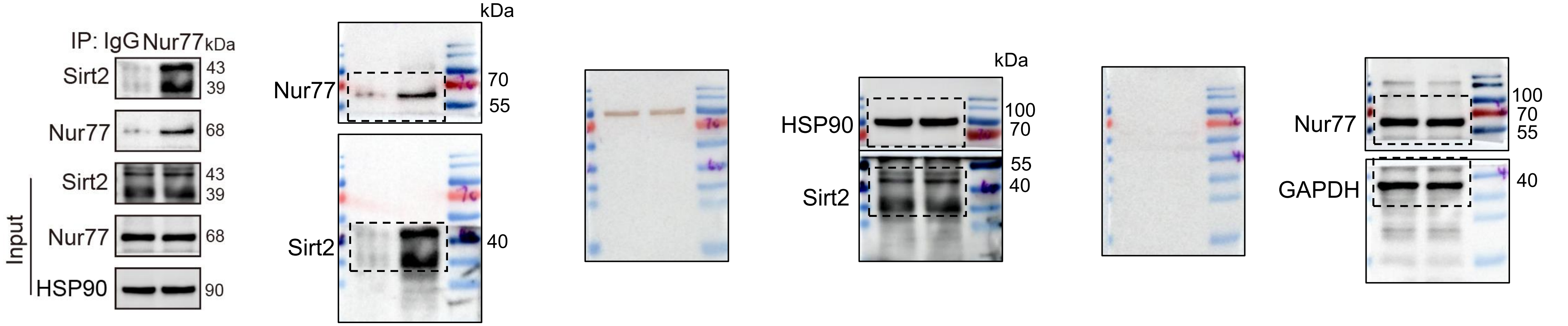

Figure S1C

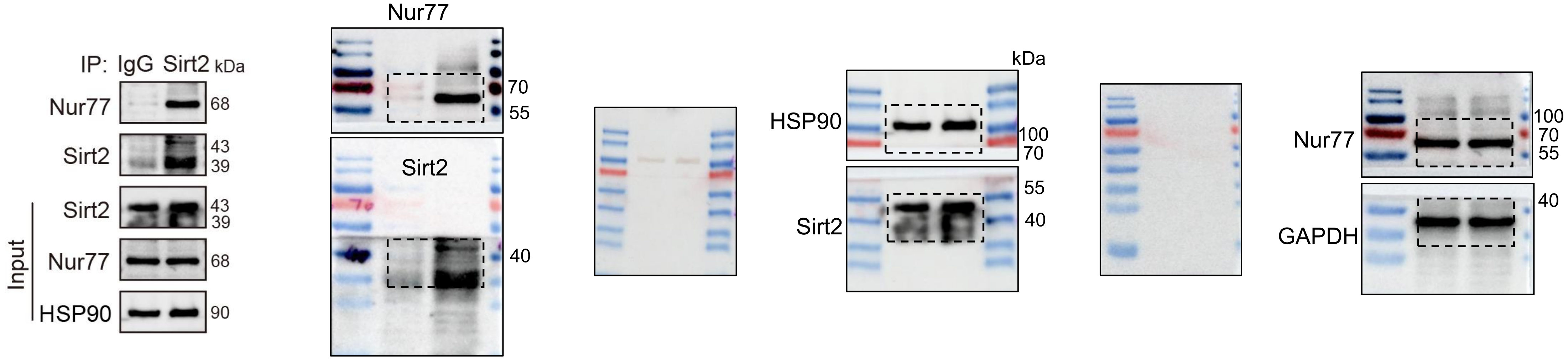

Figure S1D

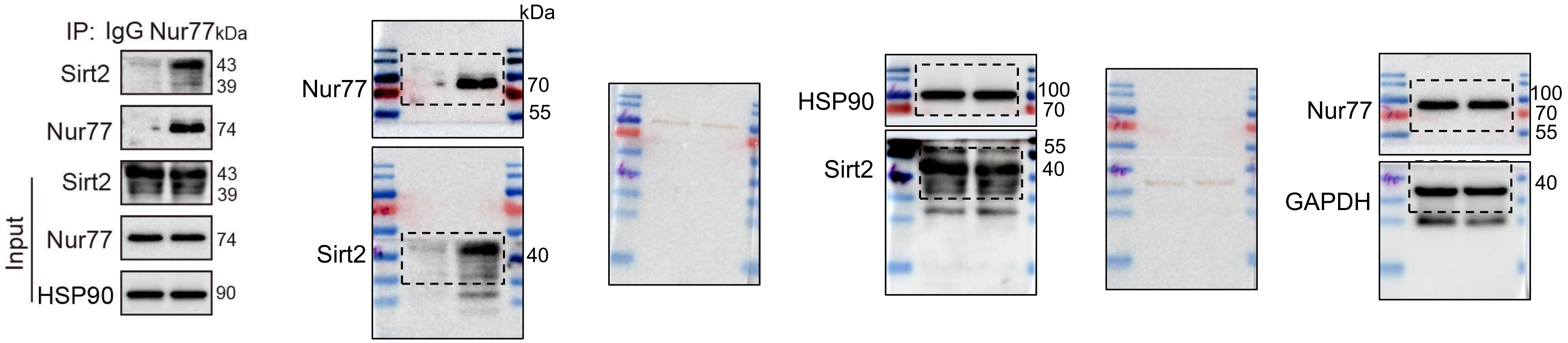

Figure S1E

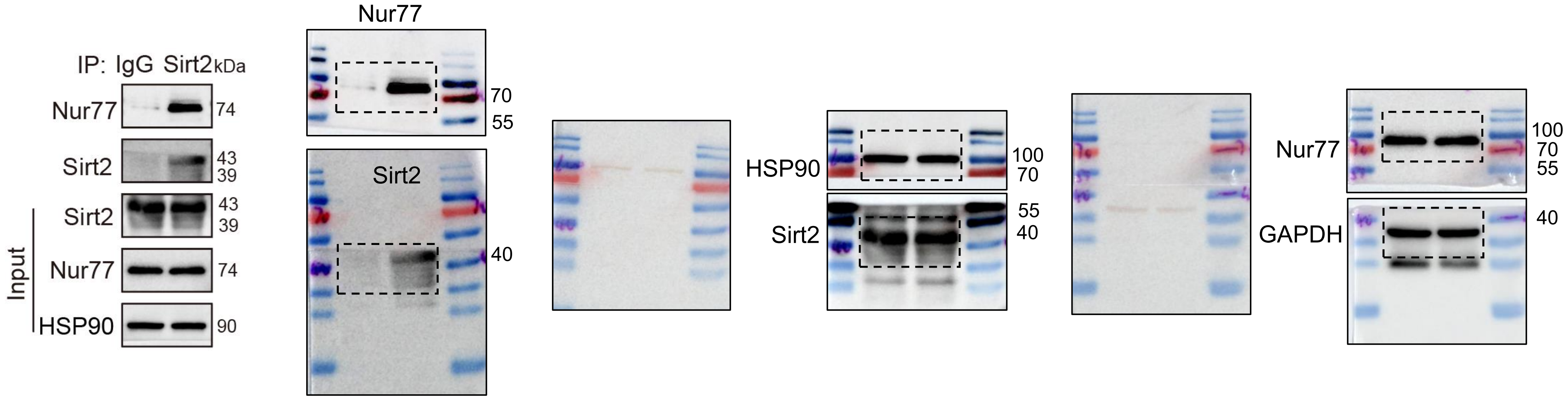

Figure S1K

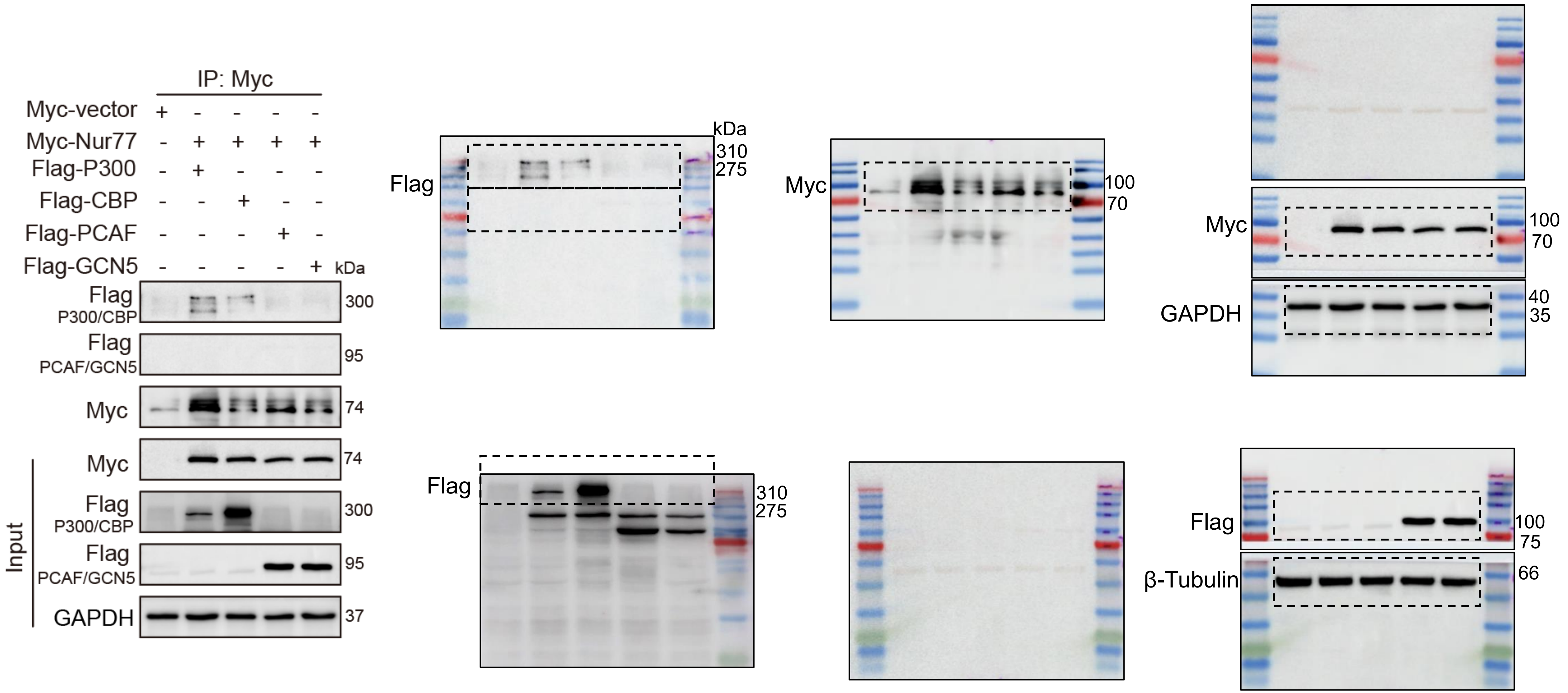
IP: Myc
Myc-vector + - - - -
Myc-Nur77 - + + + +
Flag-P300 - + - - -
Flag-CBP - - + - -
Flag-PCAF - - - + -
Flag-GCN5 - - - - +
kDa
Flag P300/CBP 300
Flag PCAF/GCN5 95
Myc 74
Input
Myc 74
Flag P300/CBP 300
Flag PCAF/GCN5 95
GAPDH 37
Flag
kDa
310
275
Myc
100
70
Myc
100
70
GAPDH
40
35
Flag
310
275
Flag
100
75
β-Tubulin
66

Figure S1L

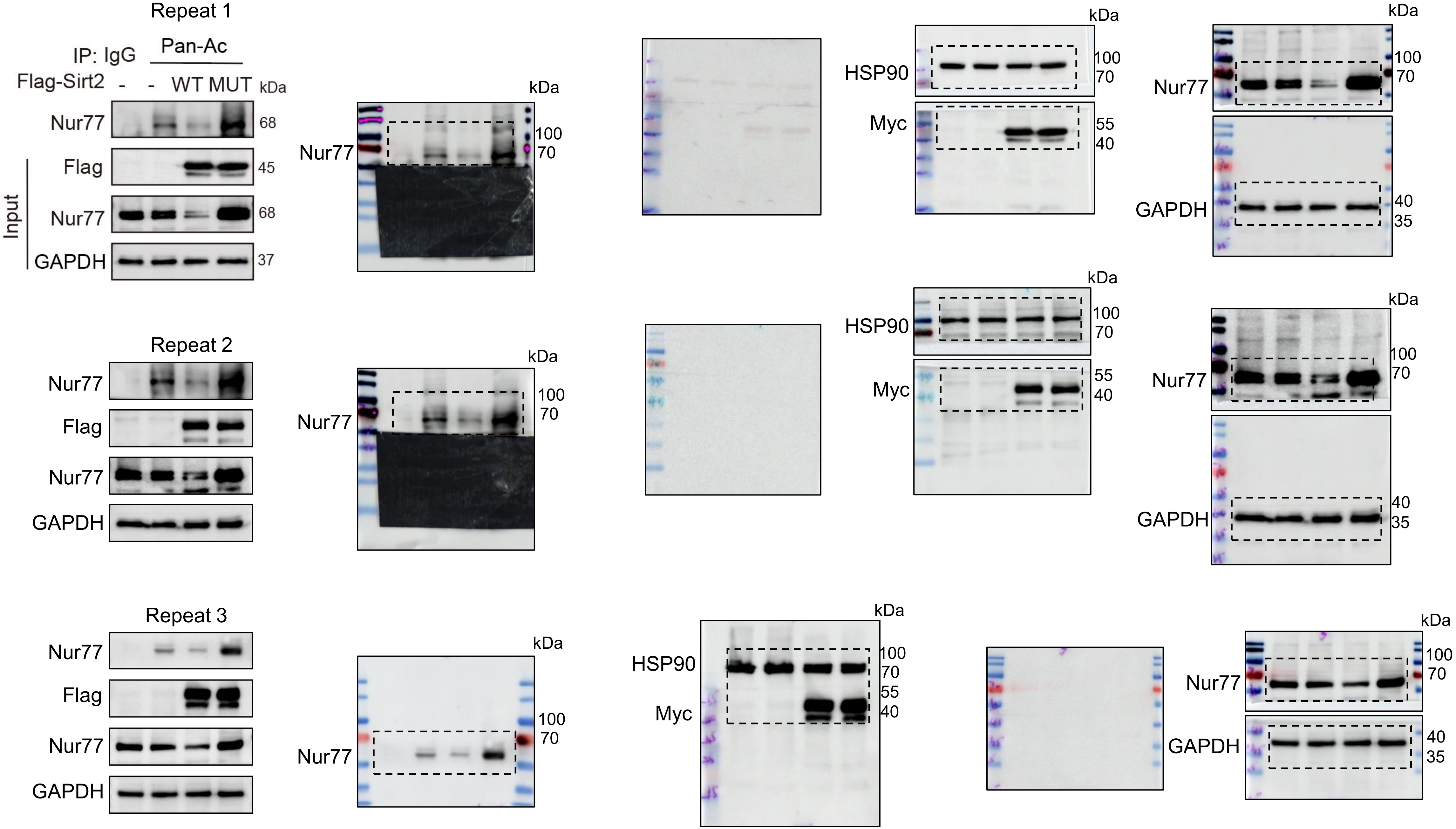
Repeat 1
IP: IgG
Pan-Ac
Flag-Sirt2
- - WT MUT
kDa
Nur77
68
Input
Flag
45
Nur77
68
GAPDH
37
Nur77
100
70
HSP90
Myc
55
40
35
Repeat 2
Nur77
Flag
Nur77
GAPDH
Repeat 3
Nur77
Flag
Nur77
GAPDH

Figure S1M

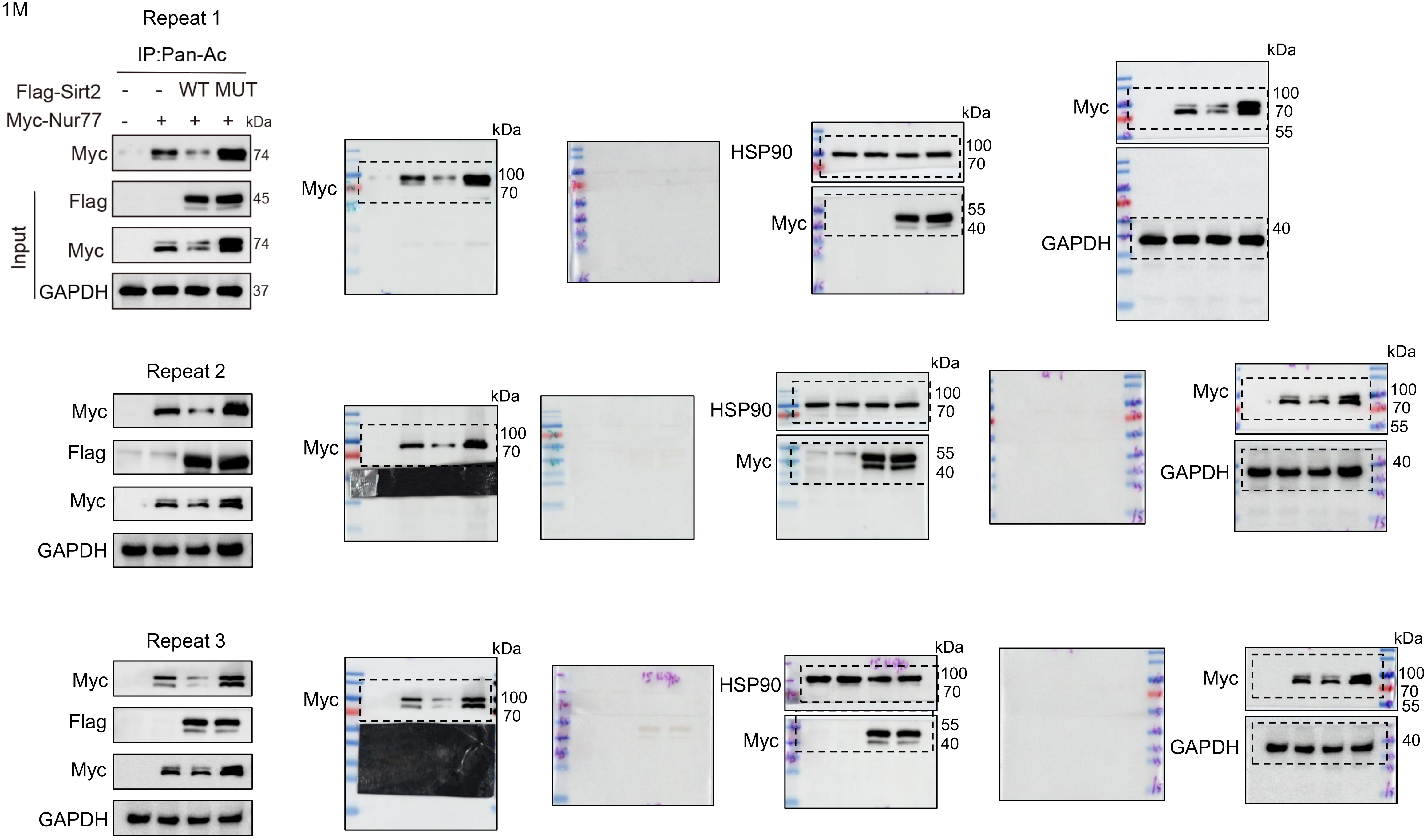
Repeat 1
IP:Pan-Ac
Flag-Sirt2 - - WT MUT
Myc-Nur77 - + + + kDa
Myc 74
Flag 45
Input
Myc 74
GAPDH 37
kDa
Myc 100 70
HSP90 100 70
kDa
Myc 55 40
kDa
Myc 100 70 55
GAPDH 40
Repeat 2
Myc
Flag
Myc
GAPDH
kDa
Myc 100 70
kDa
HSP90 100 70
Myc 55 40
kDa
Myc 100 70 55
GAPDH 40
Repeat 3
Myc
Flag
Myc
GAPDH
kDa
Myc 100 70
kDa
HSP90 100 70
Myc 55 40
kDa
Myc 100 70 55
GAPDH 40

Figure S1P

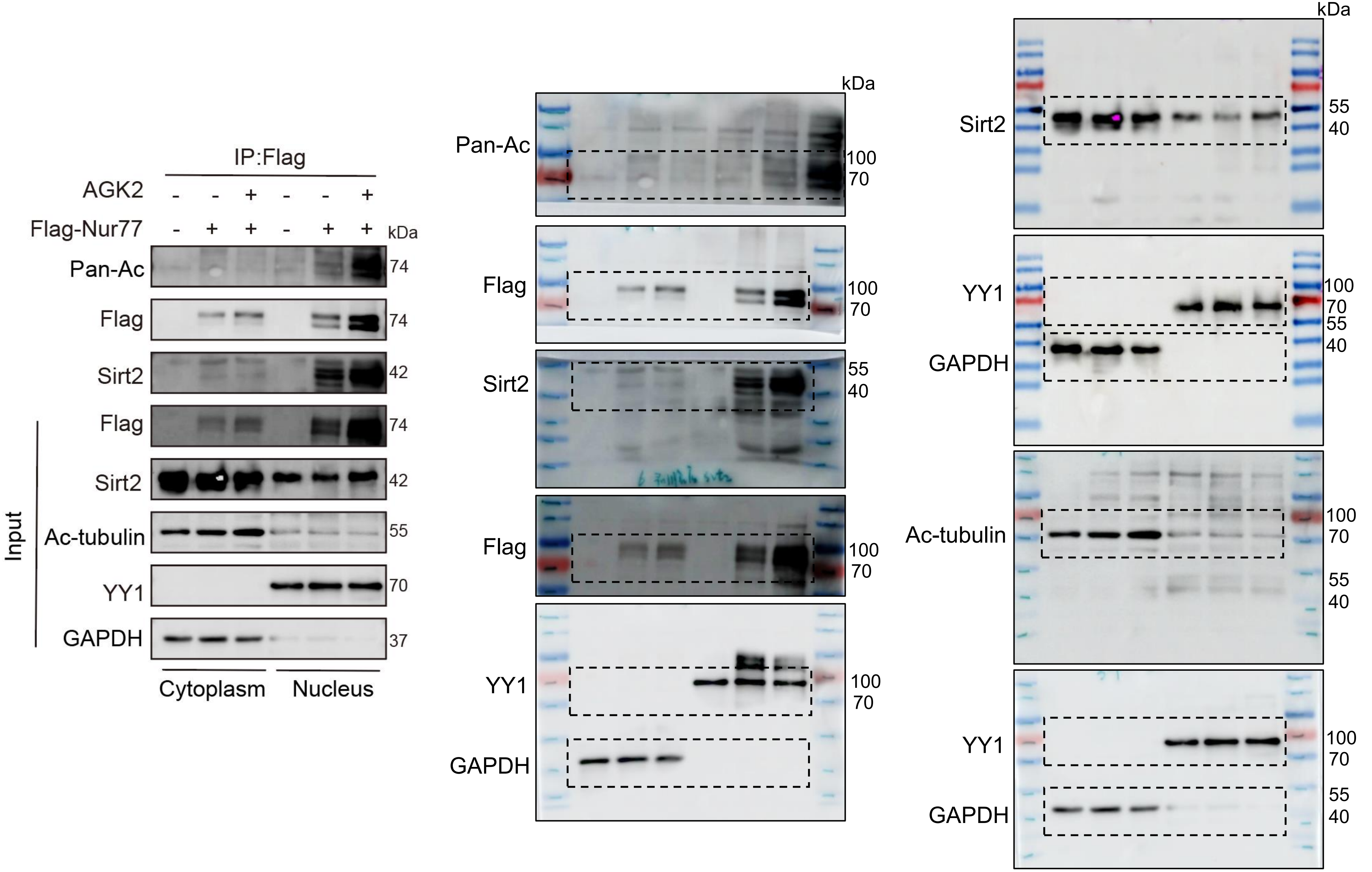
IP:Flag
AGK2
Flag-Nur77
kDa
Pan-Ac
Flag
Sirt2
Input
Ac-tubulin
YY1
GAPDH
Cytoplasm
Nucleus
100
70
55
40
74
42
37

Figure S2A

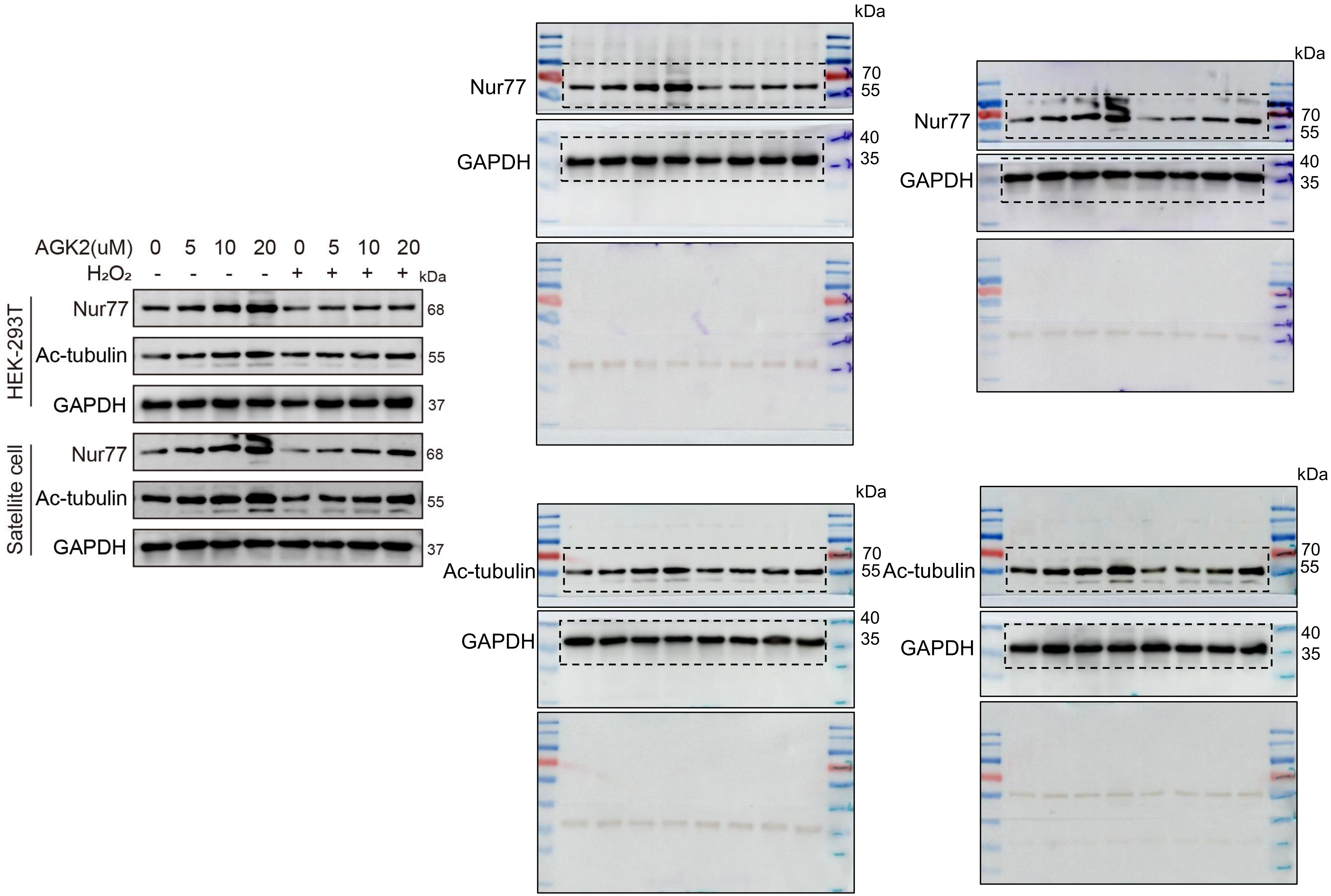
AGK2(uM) 0 5 10 20 0 5 10 20
H2O2 - - - - + + + +
kDa
HEK-293T
Nur77
68
Ac-tubulin
55
GAPDH
37
Satellite cell
Nur77
68
Ac-tubulin
55
GAPDH
37
Nur77
GAPDH
Ac-tubulin
70
55
40
35

Figure S2B

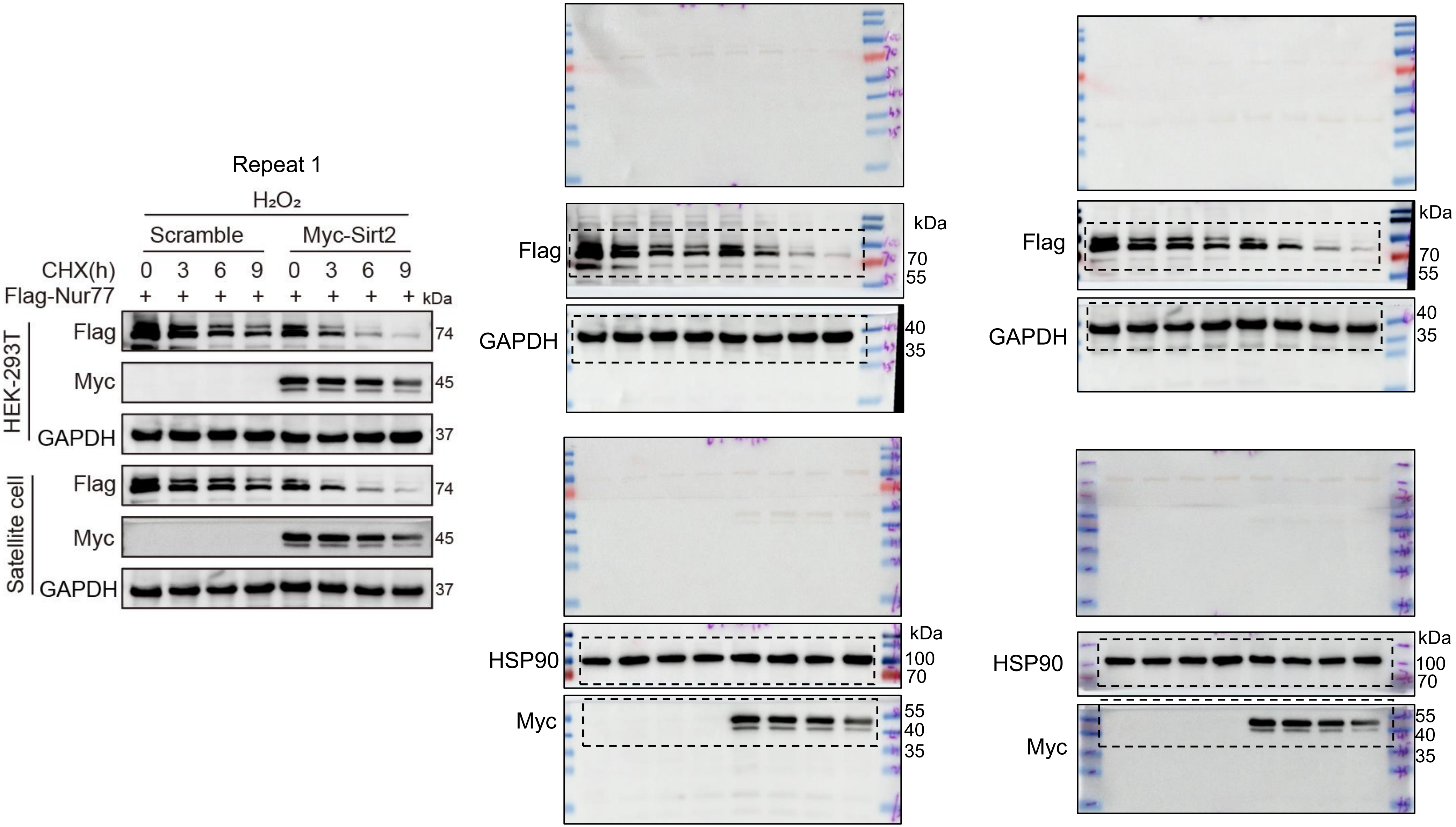
Repeat 1
H₂O₂
Scramble
Myc-Sirt2
CHX(h) 0 3 6 9 0 3 6 9
Flag-Nur77 + + + + + + + +
kDa
HEK-293T
Flag
74
Myc
45
GAPDH
37
Satellite cell
Flag
74
Myc
45
GAPDH
37
Flag
kDa
70
55
GAPDH
40
35
HSP90
100
70
Myc
55
40
35

Figure S2B

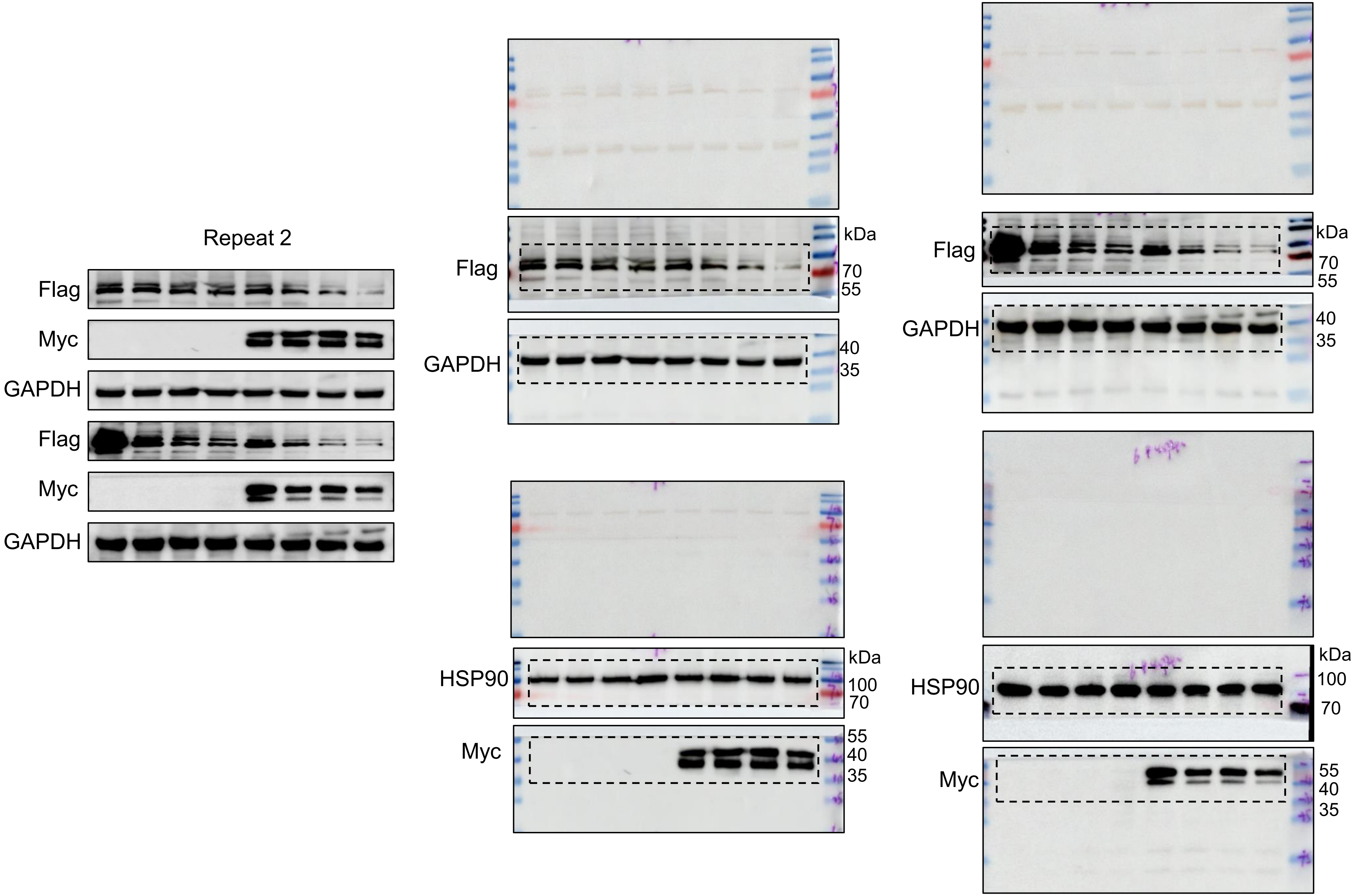
Repeat 2
Flag
Myc
GAPDH
Flag
Myc
GAPDH
Flag
kDa
70
55
GAPDH
40
35
HSP90
kDa
100
70
Myc
55
40
35
Flag
kDa
70
55
GAPDH
40
35
HSP90
kDa
100
70
Myc
55
40
35

Figure S2B

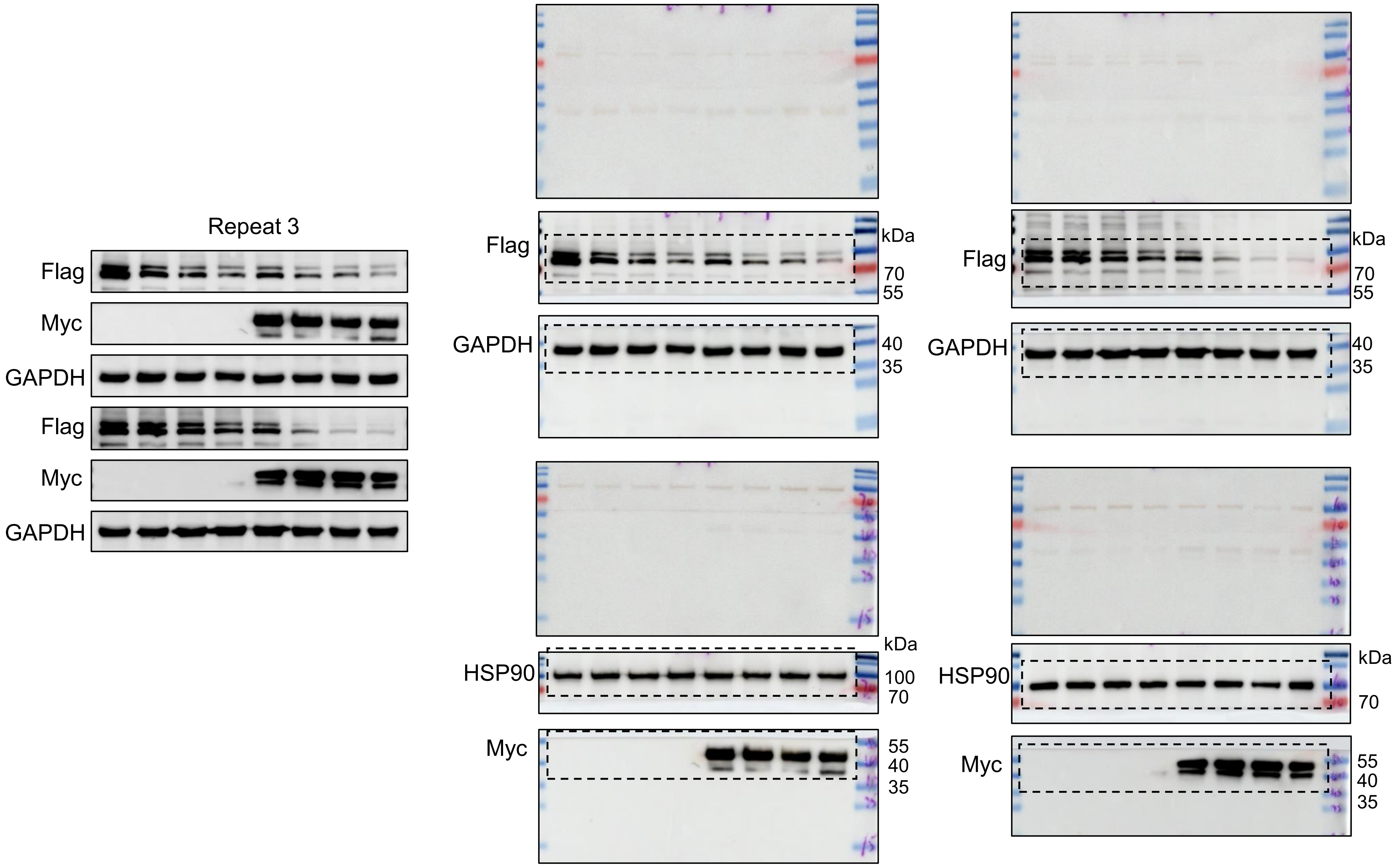

Repeat 3
Flag
Myc
GAPDH
Flag
Myc
GAPDH
Flag
kDa
70
55
GAPDH
40
35
HSP90
kDa
100
70
Myc
55
40
35
Flag
kDa
70
55
GAPDH
40
35
HSP90
kDa
70
Myc
55
40
35

Figure S2E

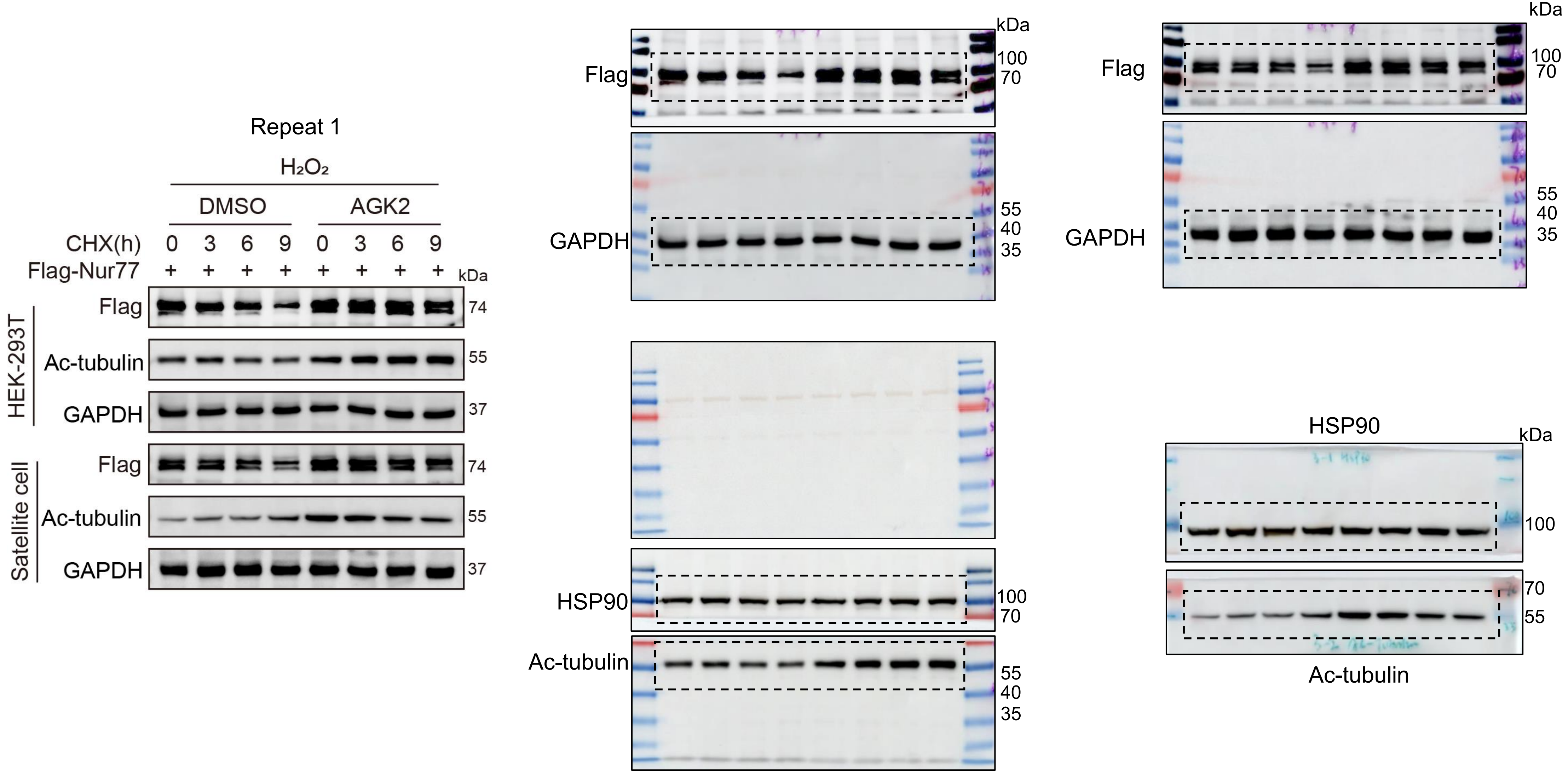

Repeat 1
H₂O₂
DMSO
AGK2
CHX(h) 0 3 6 9 0 3 6 9
Flag-Nur77 + + + + + + + +
kDa
HEK-293T
Flag 74
Ac-tubulin 55
GAPDH 37
Satellite cell
Flag 74
Ac-tubulin 55
GAPDH 37
Flag
GAPDH
HSP90
Ac-tubulin
100
70
55
40
35
HSP90
Ac-tubulin

Figure S2E

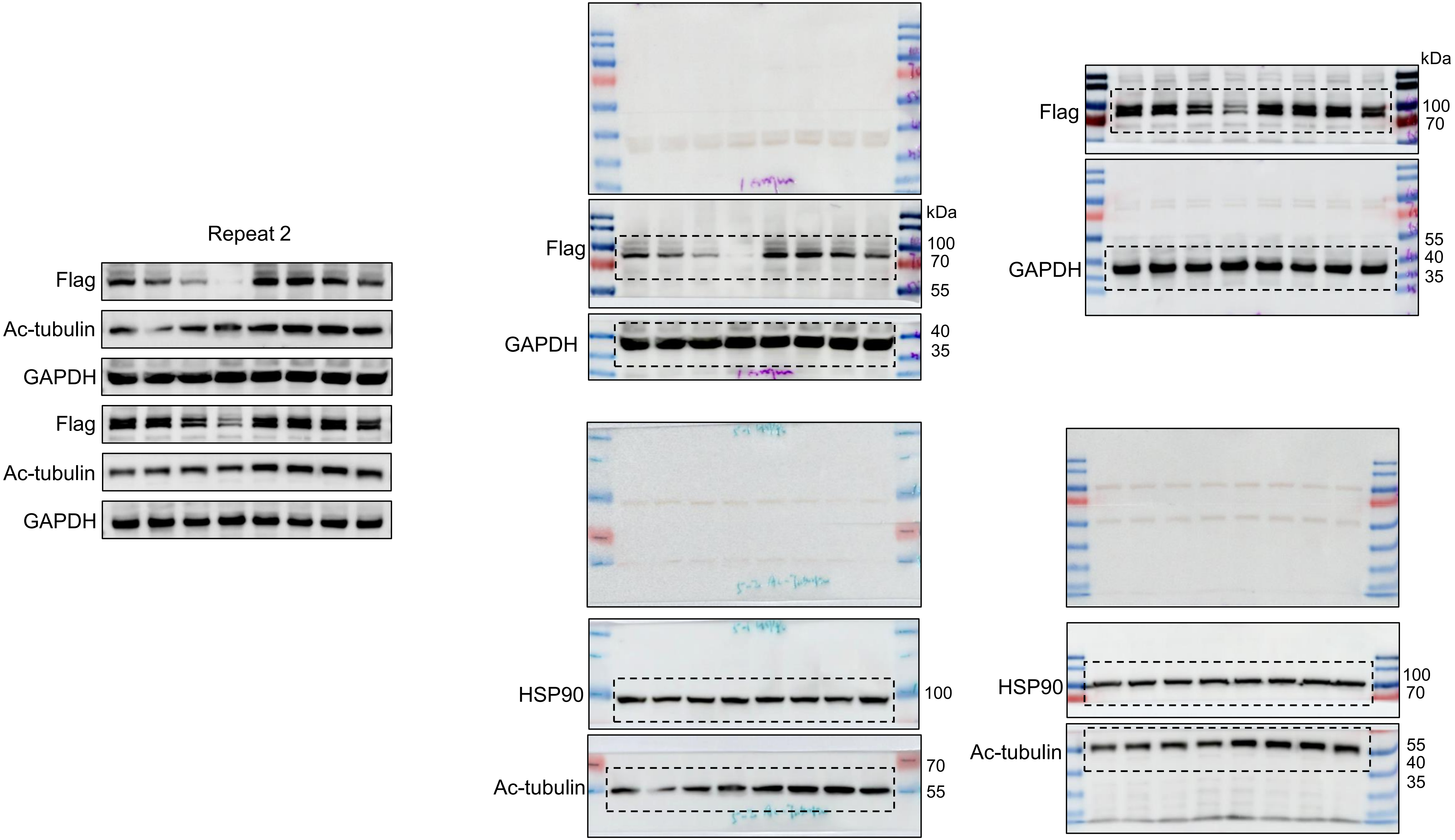

Repeat 2
Flag
Ac-tubulin
GAPDH
Flag
Ac-tubulin
GAPDH
kDa
Flag
100
70
55
GAPDH
40
35
HSP90
100
Ac-tubulin
70
55
kDa
Flag
100
70
GAPDH
55
40
35
HSP90
100
70
Ac-tubulin
55
40
35

Figure S2E

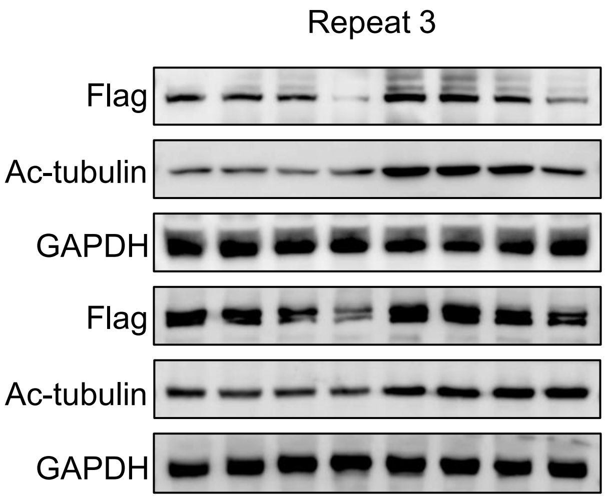

Repeat 3
Flag
Ac-tubulin
GAPDH
Flag
Ac-tubulin
GAPDH

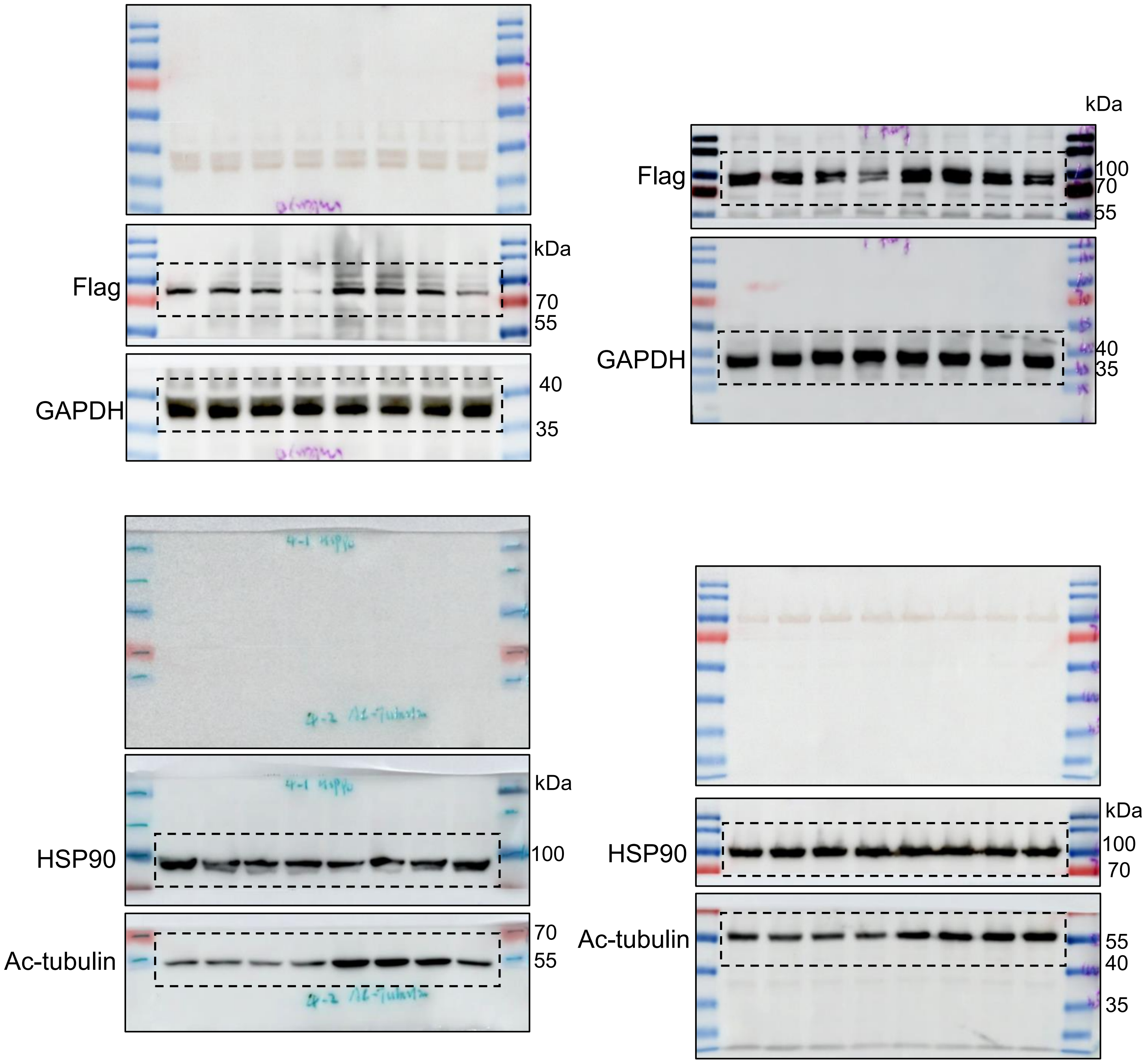

Flag
kDa
70
55
GAPDH
40
35
kDa
Flag
100
70
55
GAPDH
40
35
HSP90
kDa
100
Ac-tubulin
70
55
kDa
HSP90
100
70
Ac-tubulin
55
40
35

Figure S2H

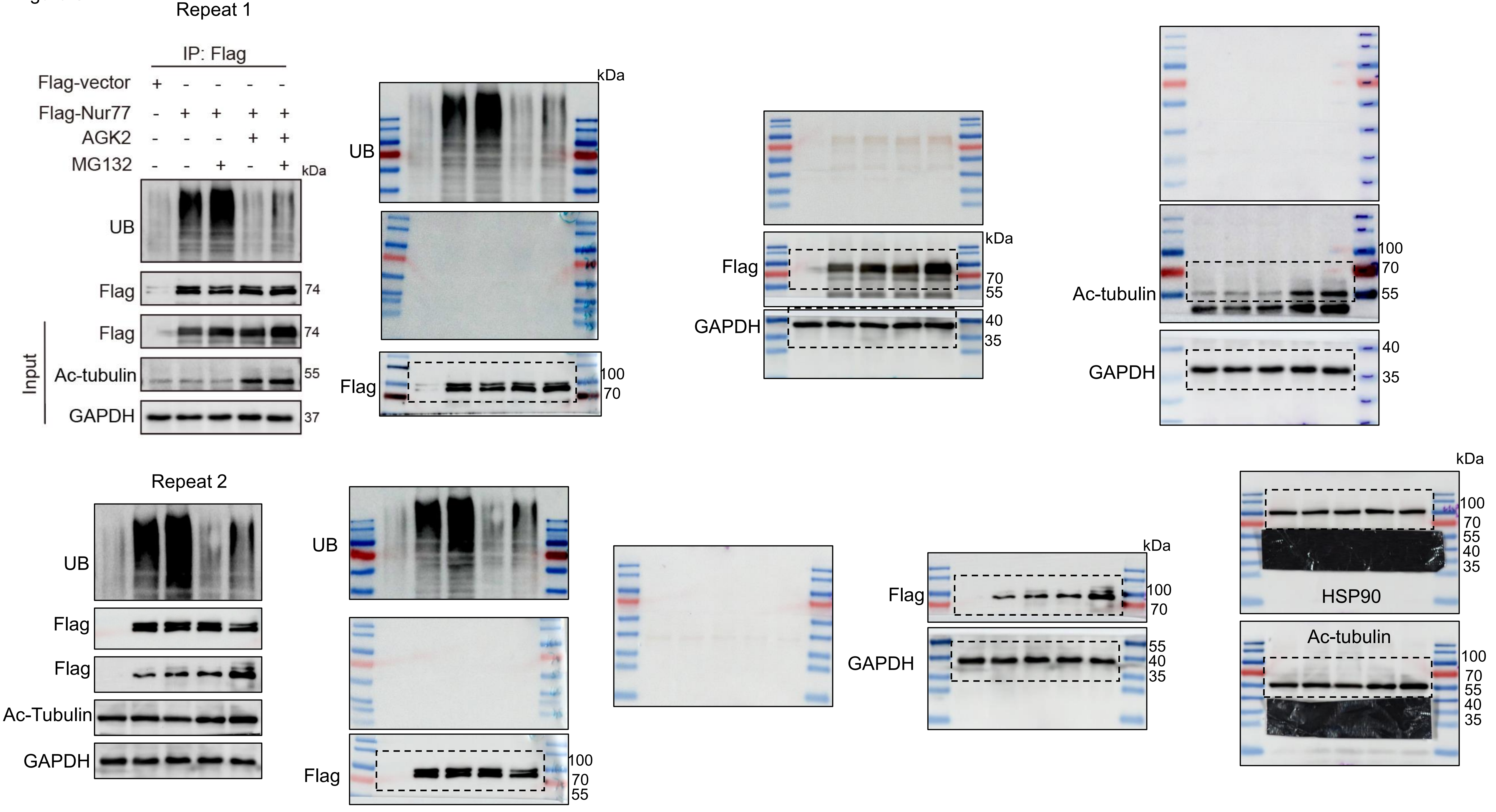

Repeat 1
IP: Flag
Flag-vector + - - - -
Flag-Nur77 - + + + +
AGK2 - - - + +
MG132 - - + - +
kDa
UB
Flag
74
Input
Flag
74
Ac-tubulin
55
GAPDH
37
UB
Flag
100
70
Flag
GAPDH
70
55
40
35
Ac-tubulin
100
70
55
GAPDH
40
35
Repeat 2
UB
Flag
Flag
Ac-Tubulin
GAPDH
UB
Flag
100
70
55
Flag
100
70
GAPDH
55
40
35
HSP90
100
70
55
40
35
Ac-tubulin
100
70
55
40
35

Figure S2H

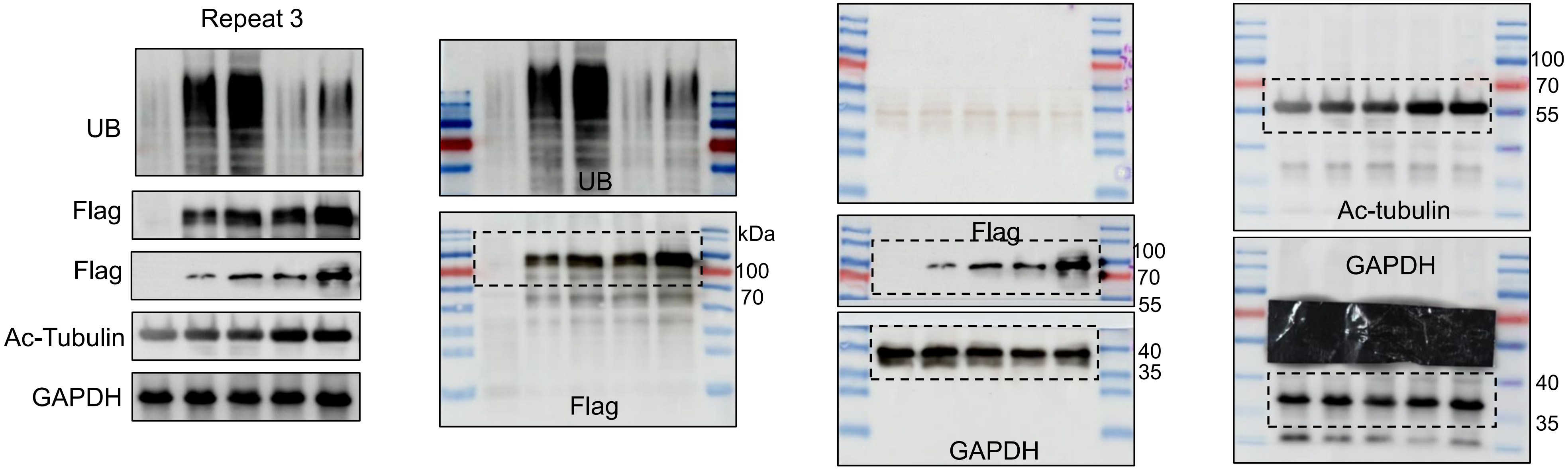
Repeat 3
UB
Flag
Flag
Ac-Tubulin
GAPDH
UB
kDa
100
70
Flag
Flag
100
70
55
40
35
GAPDH
100
70
55
Ac-tubulin
GAPDH
40
35

Figure S2I

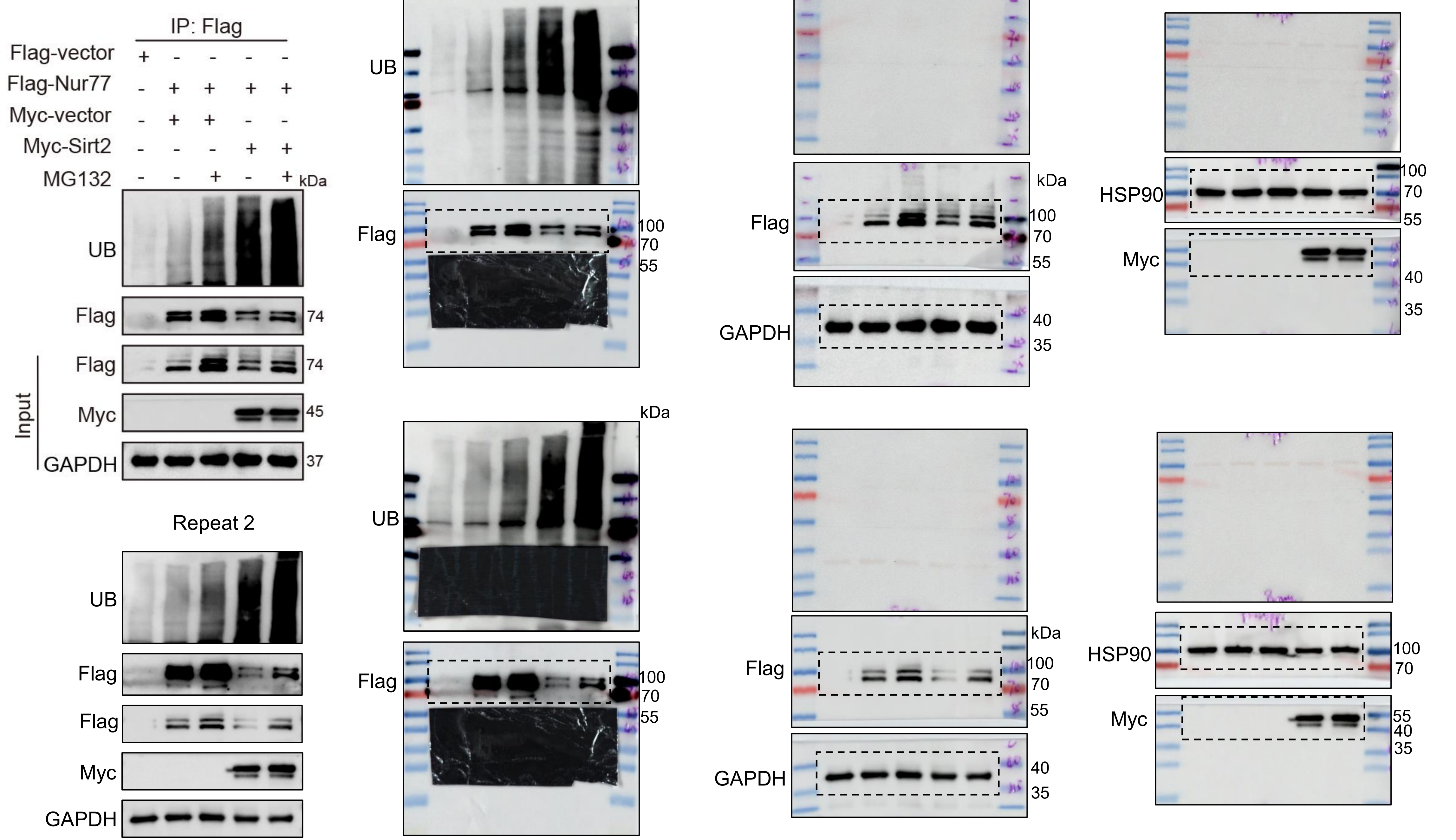
Repeat 1
IP: Flag
Flag-vector + - - - -
Flag-Nur77 - + + + +
Myc-vector - + + - -
Myc-Sirt2 - - - + +
MG132 - - + - +
kDa
UB
Flag 74
Input
Flag 74
Myc 45
GAPDH 37
Repeat 2
UB
Flag
Flag
Myc
GAPDH
kDa
UB
Flag
100
70
55
kDa
UB
Flag
100
70
55
kDa
Flag
100
70
55
GAPDH
40
35
HSP90
100
70
55
Myc
40
35
kDa
Flag
100
70
55
GAPDH
40
35
HSP90
100
70
Myc
55
40
35

Figure S2I

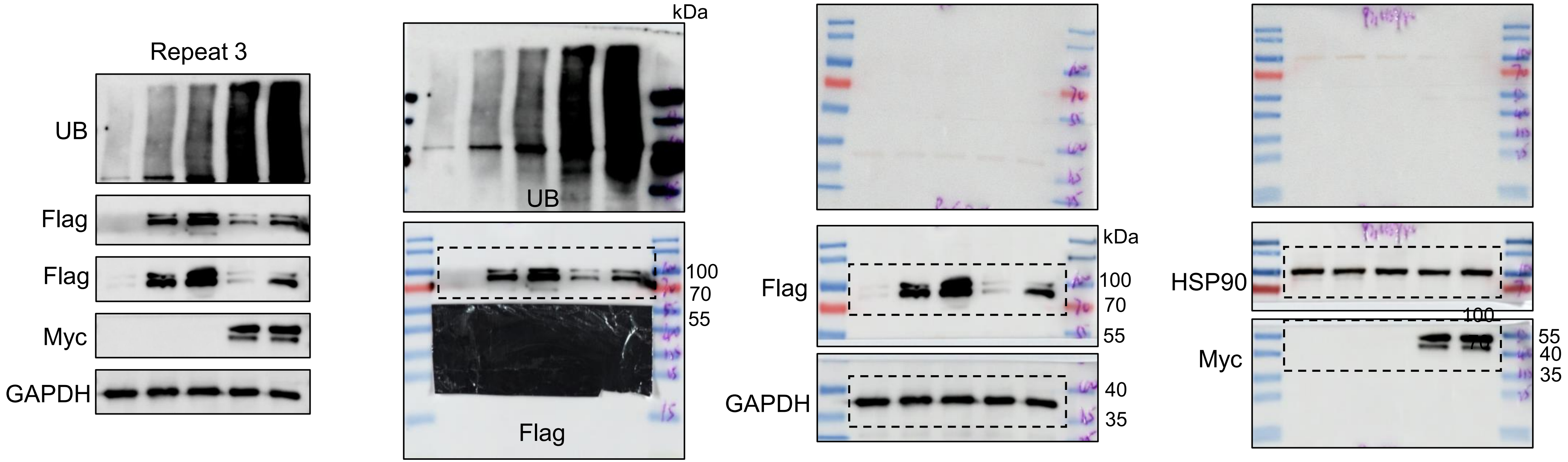

Repeat 3
UB
Flag
Flag
Myc
GAPDH
kDa
UB
100
70
55
Flag
Flag
kDa
100
70
55
GAPDH
40
35
HSP90
Myc
100
70
55
40
35

Figure S2J

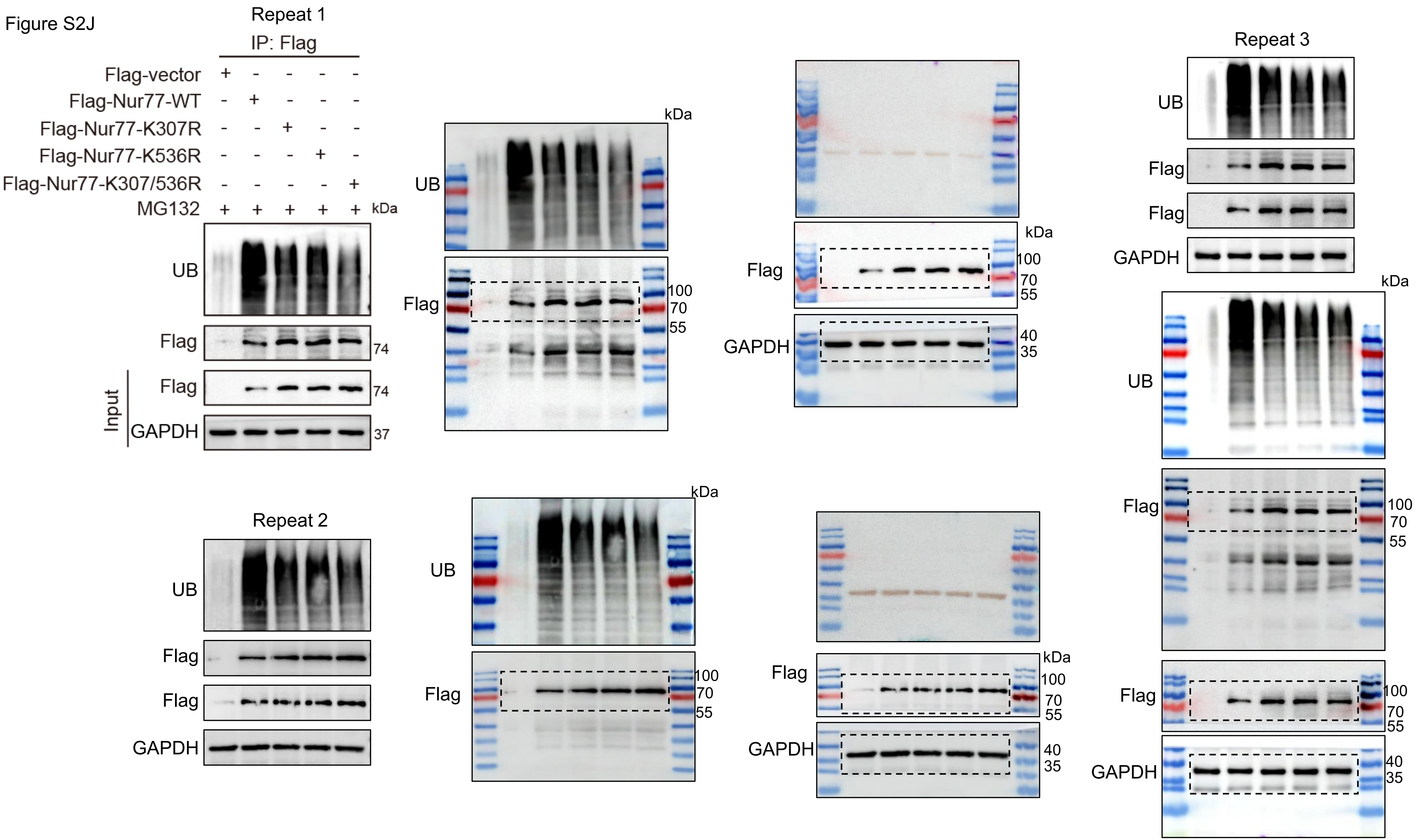
Repeat 1
IP: Flag
Flag-vector
Flag-Nur77-WT
Flag-Nur77-K307R
Flag-Nur77-K536R
Flag-Nur77-K307/536R
MG132
kDa
UB
Flag
74
Input
Flag
74
GAPDH
37
UB
Flag
100
70
55
Flag
100
70
55
GAPDH
40
35
Repeat 2
UB
Flag
Flag
GAPDH
UB
Flag
100
70
55
Flag
100
70
55
GAPDH
40
35
Repeat 3
UB
Flag
Flag
GAPDH
UB
Flag
100
70
55
Flag
100
70
55
GAPDH
40
35

Figure S3C

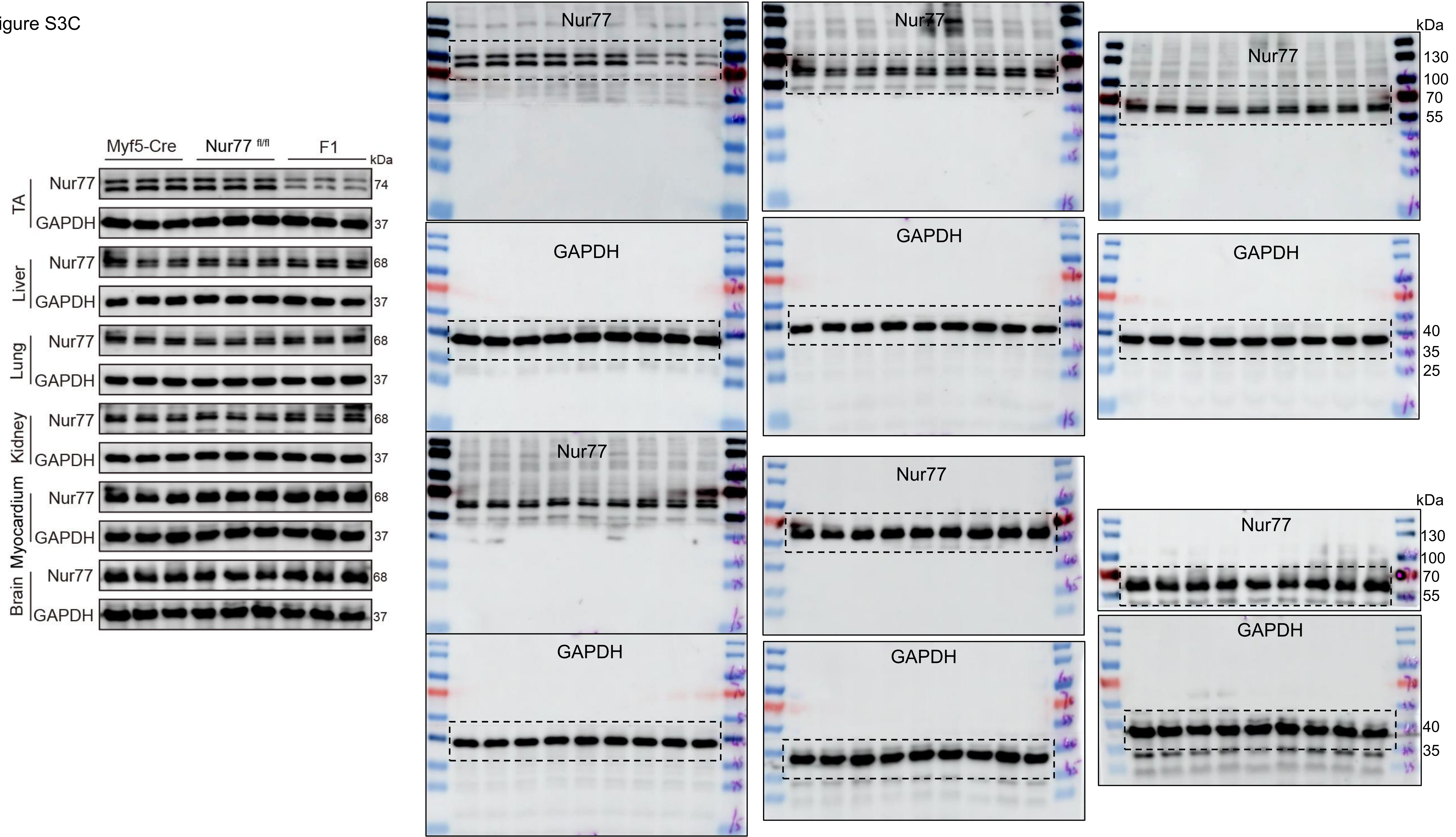
Myf5-Cre
Nur77 fl/fl
F1
kDa
TA
Liver
Lung
Kidney
Myocardium
Brain
Nur77
GAPDH
74
68
37
130
100
70
55
40
35
25

Figure S4A

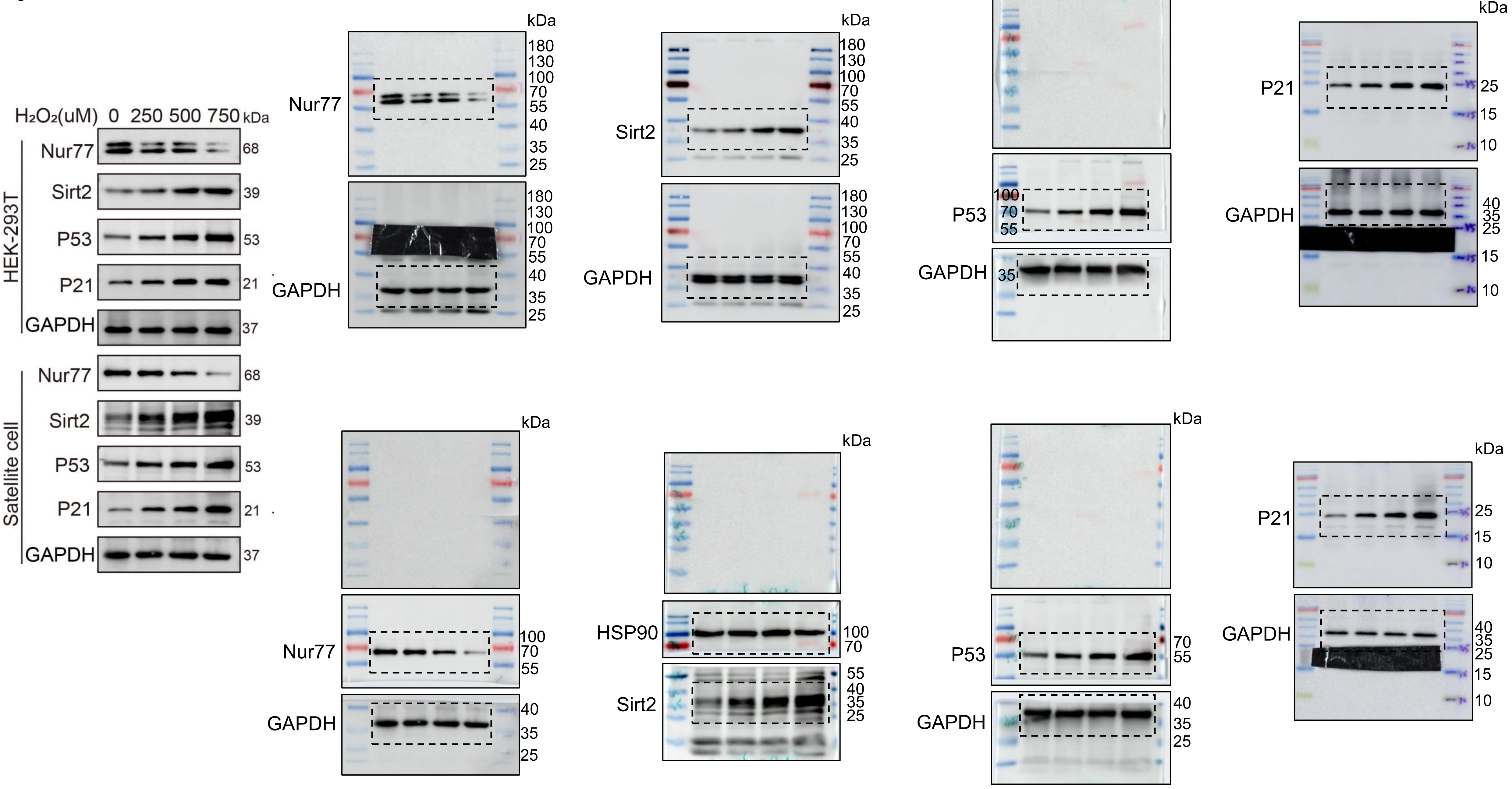

H2O2(uM) 0 250 500 750 kDa
HEK-293T
Nur77 68
Sirt2 39
P53 53
P21 21
GAPDH 37
Satellite cell
Nur77 68
Sirt2 39
P53 53
P21 21
GAPDH 37
kDa
Nur77
GAPDH
Sirt2
P53
P21
HSP90
180
130
100
70
55
40
35
25
15
10

Figure S4B

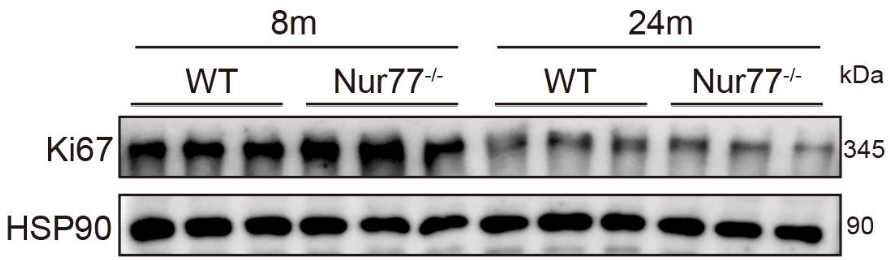

8m
24m
WT
Nur77-/-
WT
Nur77-/-
kDa
Ki67
345
HSP90
90

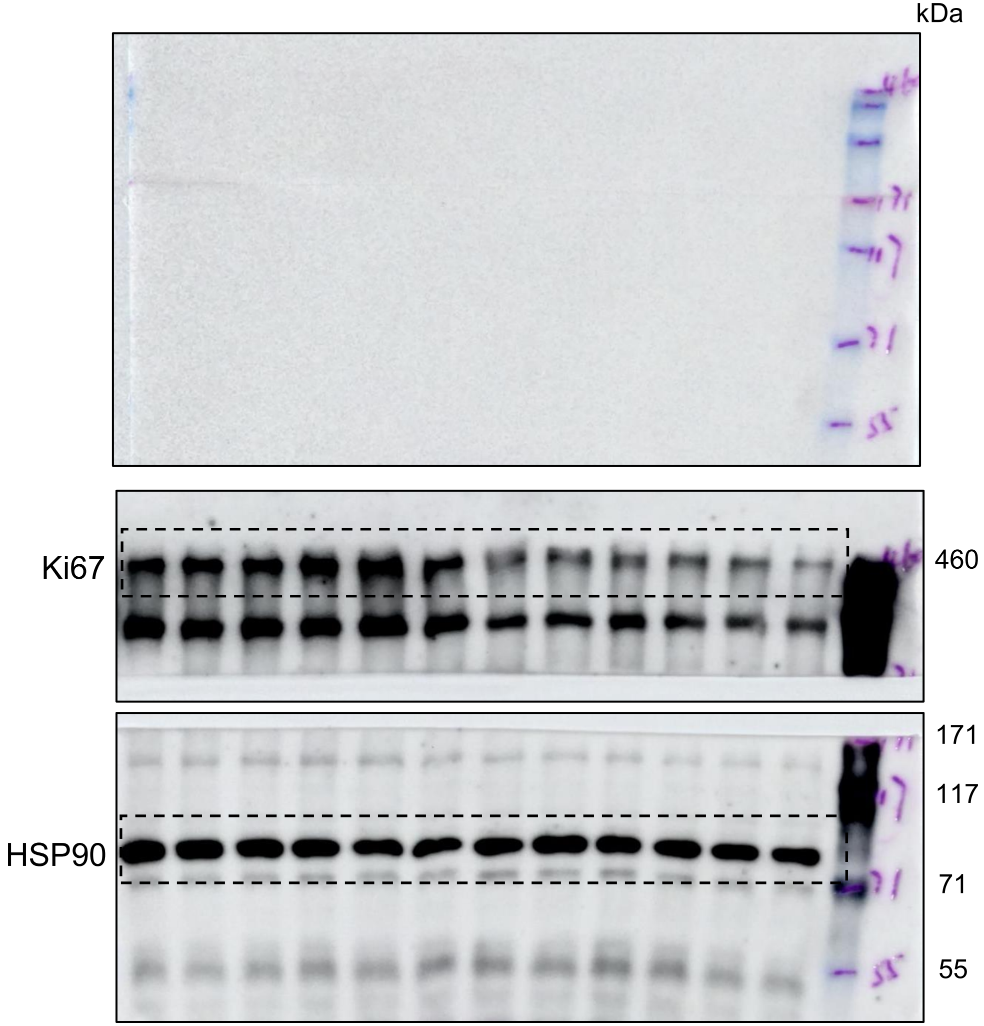

kDa
Ki67
460
171
117
HSP90
71
55

Figure S4D

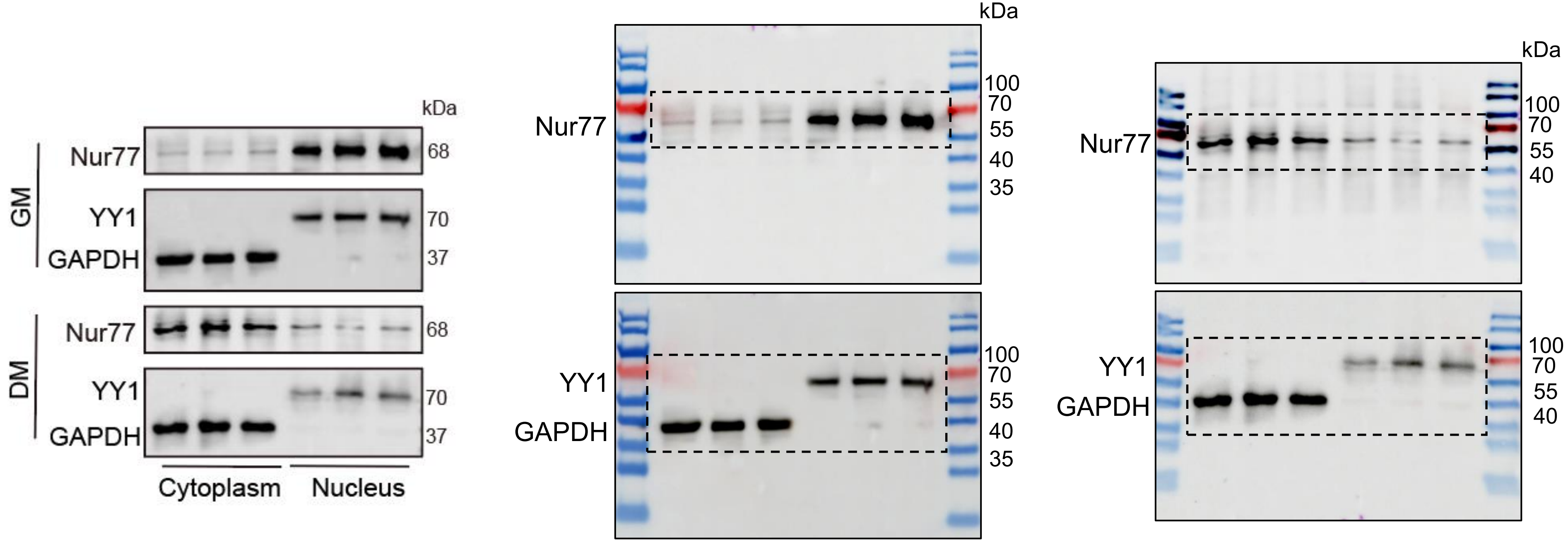

Figure S4E

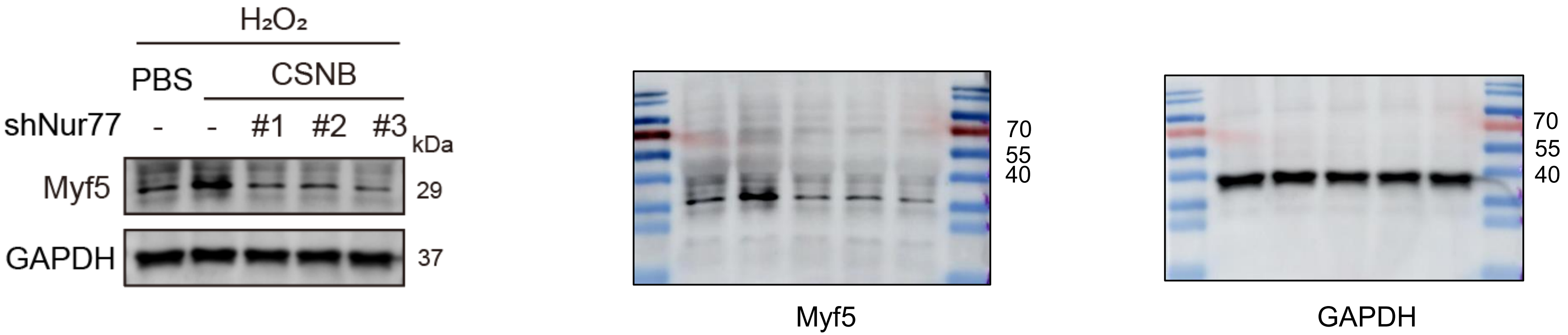

Figure S5C

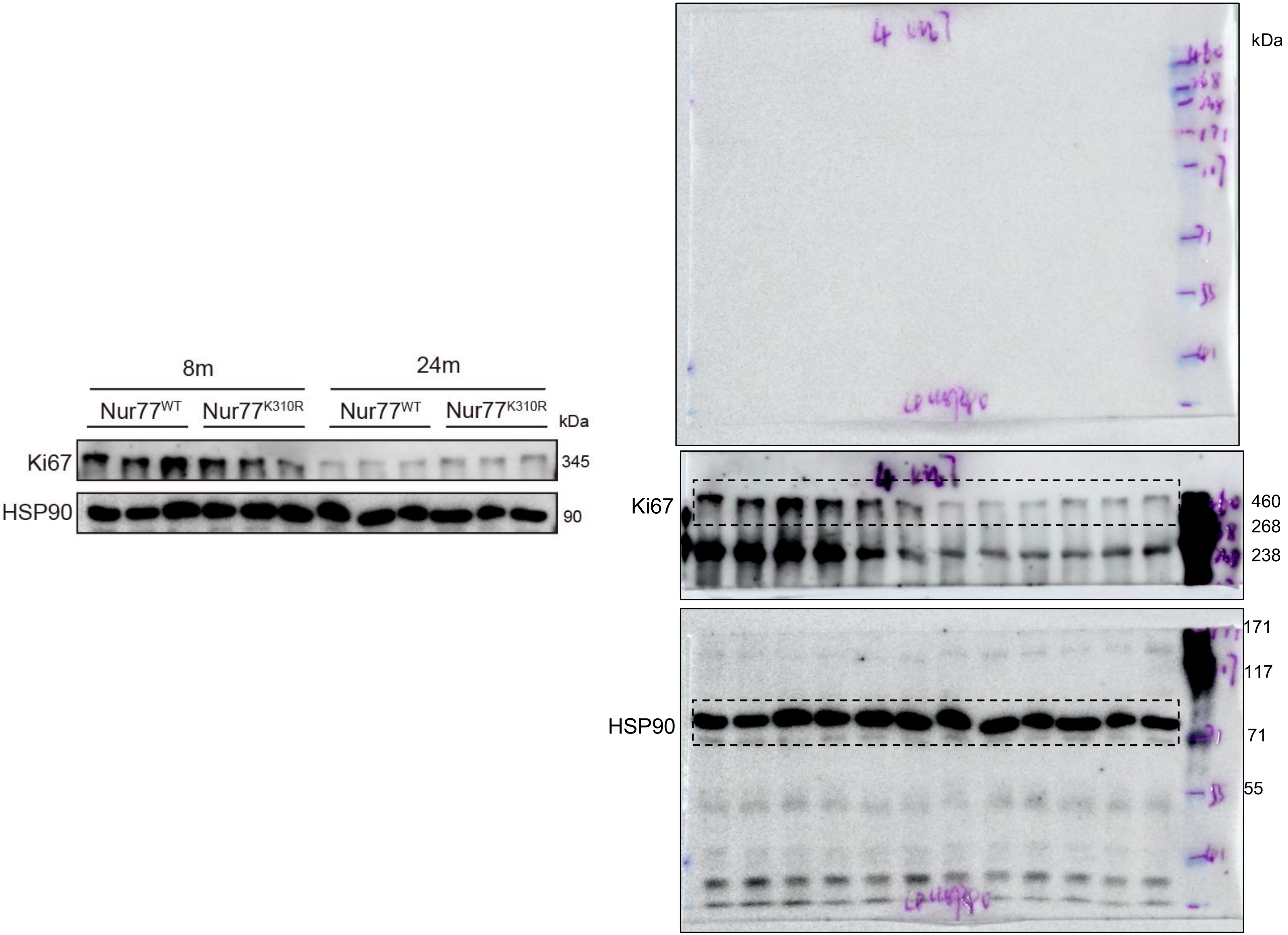

8m
24m
Nur77WT
Nur77K310R
Nur77WT
Nur77K310R
kDa
Ki67
345
HSP90
90
kDa
Ki67
460
268
238
HSP90
171
117
71
55
