# Supplementary material for: The Sirt2–Nur77 axis regulates muscle stem cell quiescence and senescence via epigenetic–metabolic synergy
Source: Cell Death Dis. 2026 Mar 28;17(1):429. doi: 10.1038/s41419-026-08645-w (PMC13153389; doi:10.1038/s41419-026-08645-w)
Supplement: Supplementary file 8 — aj-checklist [file 41419_2026_8645_MOESM8_ESM.pdf]

SPRINGER NATURE

Corresponding Author Name: Difei Wang
Manuscript Number: CDDIS-25-6277

Reporting Summary

Springer Nature wishes to improve the reproducibility of the work that we publish. This checklist is used to ensure good reporting standards and to improve the reproducibility. Please respond completely to all questions relevant to your manuscript. For more information, please read the journal's Guide to Authors.

☑ **Check here to confirm that the following information is available in the Material & Methods section:**

- **The exact sample size (*n*)** for each experimental group/condition, given as a number, not a range
- **A description of the sample collection** allowing the reader to understand whether the samples represent technical or biological replicates (including how many animals, litters, culture, etc.)
- **A statement of how many times the experiment shown was replicated in the laboratory**
- **Definitions of statistical methods and measures**: For small sample sizes (n<5) descriptive statistics are not appropriate, instead plot individual data points
  - Very common tests, such as *t*-test, simple χ^2 tests, Wilcoxon and Mann-Whitney tests, can be unambiguously identified by name only, but more complex techniques should be described in the methods section
  - Are tests one-sided or two-sided?
  - Are there adjustments for multiple comparisons?
  - **Statistical test results**, e.g., ***P* values**
  - Definition of **'center values'** as **median or mean**;
  - Definition of **error bars as s.d. or s.e.m. or c.i.**

Please ensure that the answers to the following questions are reported in the manuscript itself. We encourage you to include a specific subsection in the methods section for statistics, reagents and animal models. Below, provide the page number or section and paragraph number.

Statistics and general methods

Reported in section/paragraph or page #

1. How was the sample size chosen to ensure adequate power to detect a pre-specified effect size? (Give section/paragraph or page #)

For animal studies, include a statement about sample size estimate even if no statistical methods were used.

1. The sample size for animal experiments in this study was not determined by a pre-specified power analysis. Instead, it was selected in accordance with the 3R principles (Replacement, Reduction, and Refinement) to minimize animal use while ensuring scientific rigor. The choice was based on established practices in the field of muscle aging and satellite cell research, with reference to high-impact publications (e.g., PMID: 33116312 in Nature) that commonly use 6 mice per group with balanced sex distribution. This sample size is widely accepted in the literature for detecting biologically relevant effects in similar experimental paradigms. Details regarding animal numbers, grouping, and sex allocation are provided in the figure legends (lines 1037 – 1196) of the manuscript.

2. Describe inclusion/exclusion criteria if samples or animals were excluded from the analysis. Were the criteria pre-established? (Give

2. No animals or samples were excluded from the final analysis in this study. All experimental animals completed the predetermined full protocol. Prior to conducting formal experiments, sufficient pilot studies were performed to optimize and master all technical procedures (including injections and behavioral tests), ensuring that no animal deaths or unintended injuries occurred during formal experiments due to technical errors. All data collected were valid and included in the final statistical analysis. Detailed animal experimental protocols and procedures are described in the "Animal model" section of "Materials and Methods" (lines 637-668).

section/paragraph or page #)

3. If a method of randomization was used to determine how samples/animals were allocated to experimental groups and processed, describe it. (Give section/paragraph or page #)

For animal studies, include a statement about randomization even if no randomization was used.

3. The experimental animals were allocated to groups using a randomized procedure. Specifically, after completion of genotyping, a researcher not involved in subsequent behavioral testing or data analysis used a computer-generated random number table to randomly assign wild-type and knockout mice of the same litter and sex into different experimental groups (lines 643-647).
4.Blinding: Due to the inherently identifiable nature of the experimental interventions, the researchers performing the procedures could not be blinded to group allocation. To minimize assessment bias, blinding was implemented during critical phases of outcome evaluation: histological analysis, image quantification, and related assessments were independently performed by researchers unaware of the experimental groupings. Whenever feasible, the execution of behavioral tests was also conducted by personnel not involved in animal allocation. (lines 648-654).
5. Yes, all statistical tests employed for every figure in this study were rigorously justified to ensure appropriateness for the respective data types and experimental designs. (lines 800-812).

4. If the investigator was blinded to the group allocation during the experiment and/or when assessing the outcome, state the extent of blinding. (Give section/paragraph or page #)

For animal studies, include a statement about blinding even if no blinding was done.

5. For every figure, are statistical tests justified as appropriate?

Do the data meet the assumptions of the tests (e.g., normal distribution)?

Is there an estimate of variation within each group of data?

Is the variance similar between the groups that are being statistically compared? (Give section/paragraph or page #)

Reagents

6. Report the source of antibodies (vendor and catalog number)

7. Identify the source of cell lines and report if they were recently authenticated (e.g., by STR profiling) and tested for mycoplasma contamination

6. Detailed information for all antibodies used in this study, including the vendor, catalog number, clonality (monoclonal/polyclonal), and host species (e.g., rabbit, mouse), has been systematically provided in Supplementary Table 3 within the Supplementary Information.
7. The detailed source information (including name and vendor/depositary) for all cell lines used in this study is provided in Supplementary Table 3 within the Supplementary Information. Prior to experimental use, all cell lines were authenticated by Short Tandem Repeat (STR) profiling and confirmed to be free of mycoplasma contamination. (lines 696-702).

Animal Models

8. Report species, strain, sex and age of animals

9. For experiments involving live vertebrates, include a statement of compliance with ethical regulations and identify the committee(s) approving the experiments.

8. The species, strain, sex, and initial age of the experimental animals used in this study are specified in the "Animal model" subsection of the "Materials and Methods" section (lines 637-642). The precise age (in weeks) or exact time points at the time of experimentation for each group are detailed in the corresponding figure legends.
9-10. All animal procedures in this study were performed in strict compliance with ethical regulations and were approved by the Animal Ethics Committee of Shengjing Hospital, affiliated with China Medical University (Approval Number: 2023PS1235K). The animal experiments were strictly conducted in accordance with the guidelines and policies of the Animal Center of Shengjing Hospital, China Medical University, and followed the ARRIVE guidelines.This statement is explicitly provided in the "ETHICS STATEMENT" section of the manuscript (lines 1014-1017).

10. We recommend consulting the ARRIVE guidelines (*PLoS Biol.* **8**(6), e1000412,2010) to ensure that other relevant aspects of animal studies are adequately reported.

Human subjects

Reported in section/paragraph or page #

11. Identify the committee(s) approving the study protocol.

12. Include a statement confirming that informed consent was obtained from all subjects.

13. For publication of patient photos, include a statement confirming that consent to publish was obtained.

14. Report the clinical trial registration number (at ClinicalTrials.gov or equivalent).

11-16.This study is based on animal experiments and molecular investigations, and does not involve any human subjects. Therefore, the requirements regarding human research ethics (Items 11-16) are not applicable to this manuscript.

15. For phase II and III randomized controlled trials, please refer to the CONSORT statement and submit the CONSORT checklist with your submission.

16. For tumor marker prognostic studies, we recommend that you follow the REMARK reporting guidelines.

Data deposition

Reported in section/paragraph or page #

17. Provide accession codes for deposited data. Data deposition in a public repository is mandatory for:
    a. Protein, DNA and RNA sequences
    b. Macromolecular structures
    c. Crystallographic data for small molecules
    d. Microarray data

17. The primary data generated in this study (e.g., raw Western blot images, quantified statistical data) are included as figures or datasets within the main manuscript and supplementary materials. This study did not generate data types that require mandatory deposition in public repositories (e.g., high-throughput sequencing data, protein structures). Therefore, this item is not applicable.

Deposition is strongly recommended for many other datasets for which structured public repositories exist; more details on our data policy are available in the Guide to Authors. We encourage the provision of other source data in supplementary information or in unstructured repositories such as Figshare and Dryad. We encourage publication of Data Descriptors (see Scientific Data) to maximize data reuse.

18. If computer code was used to generate results that are central to the paper's conclusions, include a statement in the Methods section under "**Code availability**" to indicate whether and how the code can be accessed. Include version information as necessary and any restrictions on availability.

18. No custom or specific computer code was used to generate the central conclusions of this study. All statistical analyses were performed using standard commercial software (e.g., SPSS 26.0 and GraphPad Prism), and all image processing involved routine operations with widely available software (e.g., ImageJ). Therefore, this item regarding "Code availability" is not applicable.
